# Chemoenzymatic Formation of Oxa-Terpenoids by Sesqui- and Diterpene Synthase-Mediated Biotransformations with 9-Oxy-FPP Ether Derivatives

**DOI:** 10.1021/acs.biochem.4c00589

**Published:** 2024-12-28

**Authors:** Henry Struwe, Trang Nguyen, Svenja Schwörer, Jörn Droste, Hanke Spinck, Andreas Kirschning

**Affiliations:** † Institute of Organic Chemistry, Leibniz University Hannover, Schneiderberg 1B, Hannover 30167, Germany; ‡ Uppsala Biomedical Center (BMC), Uppsala University, Husargatan 3, Uppsala 752 37, Sweden

## Abstract

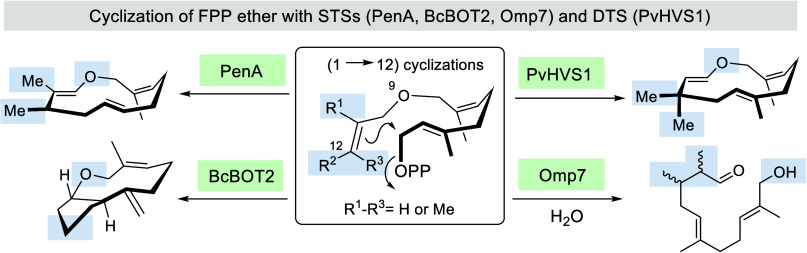

Farnesyl pyrophosphate derivatives bearing an additional oxygen atom at position 5 proved to be very suitable for expanding the substrate promiscuity of sesquiterpene synthases (STSs) and the formation of new oxygenated terpenoids. Insertion of an oxygen atom in position 9, however, caused larger restraints that led to restricted acceptance by STSs. In order to reduce some of the proposed restrictions, two FPP-ether derivatives with altered substitution pattern around the terminal olefinic double bond were designed. These showed improved promiscuity toward different STSs. Four new cyclized terpenoids with an embedded ether group were isolated and characterized. In the case of two cyclic enol ethers, also the corresponding “hydrolysis” products, linear hydroxyaldehydes, were isolated. Interestingly, all cyclization products originate from an initial 1 → 12 cyclization unprecedented when native farnesyl pyrophosphate serves as a substrate. We found that the most suitable FPP derivative with an additional oxygen at position 9 does not carry any methyl group on the terminal alkene, which likely reduces steric congestion when the preferred conformation for cyclization is adopted in the active site.

## Introduction

Terpenes constitute the largest class of secondary metabolites which are biosynthesized from common linear C_5_ precursors (dimethylallyl pyrophosphate (DMAPP) and isopentenyl pyrophosphate (IPP)) either via the mevalonate pathway or via the DXP pathway.^1^ Iterative elongation can lead to geranyl pyrophosphate, the precursor of monoterpenes, farnesyl pyrophosphate, the precursor of sesquiterpenes or geranylgeranyl pyrophosphate, the precursor of diterpenes, just to name the most common terpene classes. Terpene synthases (TSs) are responsible to induce cyclization cascades by activating the pyrophosphate moiety in the presence of the cofactor Mg^2+^.^2^ Consequently, sesquiterpene synthases (STSs) transform the linear farnesyl pyrophosphate (FPP, **1**) into complex, commonly enantiomerically pure oligocyclic systems which are enzymatically further modified, typically by late stage oxidation.^3^ In recent years, the substrate promiscuity of these synthases has been investigated in greater detail.^4^ Allemann and coworkers employed monofluorinated FPP-derivatives as substrates for germacrene A synthase (GAS) as well as for (*S*)-germacrene D synthase (GDS) and found a remarkable promiscuity of these STSs, commonly yielding new fluorinated germacrene A and D derivatives.^5^

**Scheme 1:**
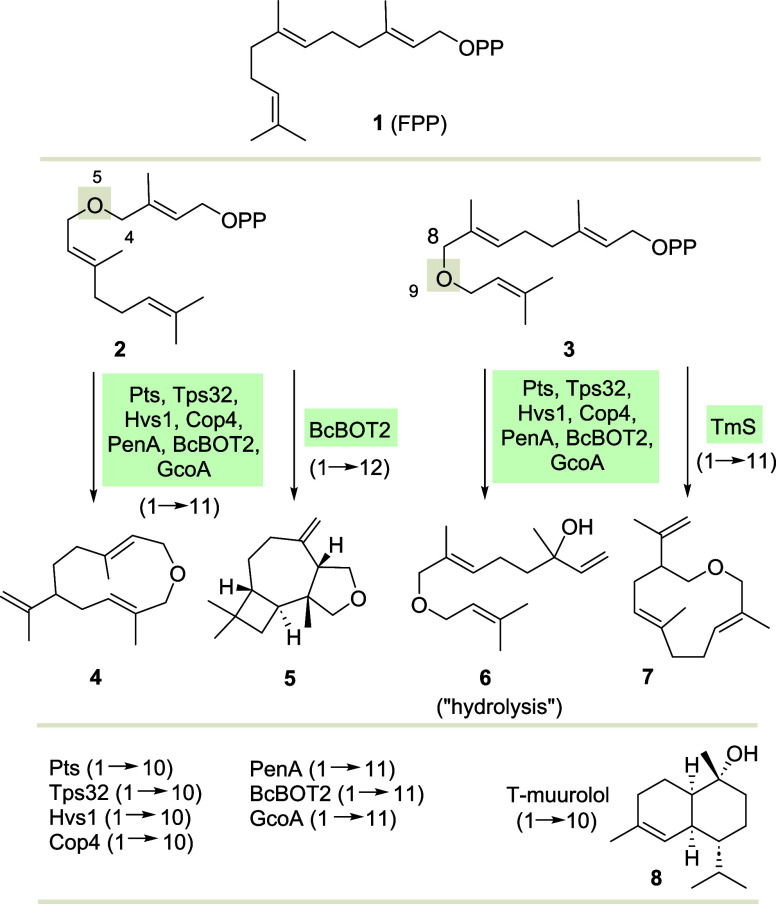
Structures of FPP **1**, and FPP Ether **2** and **3**; Previous Studies on the Promiscuity of Different STSs towards **2** and **3** and Sesquiterpene T-muurolol **8** (OPP is the Abbreviation for Diphosphate Which Is Commonly Employed as Trisammonium Salt Here)

In previous studies on the substrate promiscuity of STSs we investigated the possibility of using FPP derivatives as substrates, which bear an additional heteroatom such as oxygen or sulfur thereby leading to the elongation of the carbon backbone by one atom and in the case of oxy-FPP ether derivatives oxa-terpenoids are at hand. We found that terpenoids **4** and **5** are formed when the oxygen is located at position 5.^6^ When the oxygen atom is inserted at position 9 in FPP **1** we experienced that the resulting ether derivative **3** is less well accepted by STSs (Scheme 1). In fact, only the bacterial STS (+)-T-muurolol synthase (TmS) yielded the cyclization product **7** that had formed after a 1 → 11 ring closure. With FPP **1** TmS usually yields T-muurolol **8** that stems from an initial a 1 → 10 cyclization. All other STSs tested only provided the “hydrolysis” product **6**, which is either formed by the aqueous medium or enzymatically in which pyrophosphate activation does not lead to any cyclization event. Obviously, the introduction of an extra atom located near the aliphatic terminus of FPP hampers the facile adaption of a preferred conformation and hence does not allow initial cyclization.

This is in contrast to the observation that a one atom elongation at position 5 does not have this effect. In the present work, we more closely study this issue from the perspective of the substrate by altering the substitution pattern around the terminal olefinic double bond in FPP ether **3**. The extra oxygen atom stays at position 9.

We employed sesquiterpene synthases that are listed in our original publication on the substrate promiscuity of these enzymes. Plant-derived STSs included the patchoulol synthase (Pts) from *Pogostemon cablin*,^7^ viridiflorene synthase (Tps32) found in *Solanum lycopersicum*,^8^ 11-hydroxy vulgarisane synthase (PvHVS1) from *Prunella vulgaris*(9) and vetispiradiene synthase (Hvs1), originating from *Hyoscyamus muticus* were included.^10^ Furthermore, we utilized the bacterial caryolan-1-ol synthase (GcoA) from *Streptomyces griseus*,^11^ and (+)-T-muurolol synthase (TmS) from *Roseiflexus castenholzii*.^12^ The pentalenene synthase (PenA) is obtained from *Streptomyces exfoliatus* UC5319^13^ while protoilludene synthase (Omp7) present in various basidiomycetes including *Omphalotus olearius* furnishes the tricyclic sesquiterpene Δ6-protoilludene.^14^ In addition, two fungal STSs presilphiperfolan-8-β-ol synthase (BcBOT2) found in *Botrytis cinerea*(15) and cubebol synthase (Cop4) that originates from *Coprinus cinereus* were chosen.^16^

## Results and Discussion

### Chemical Synthesis

**Scheme 2:**
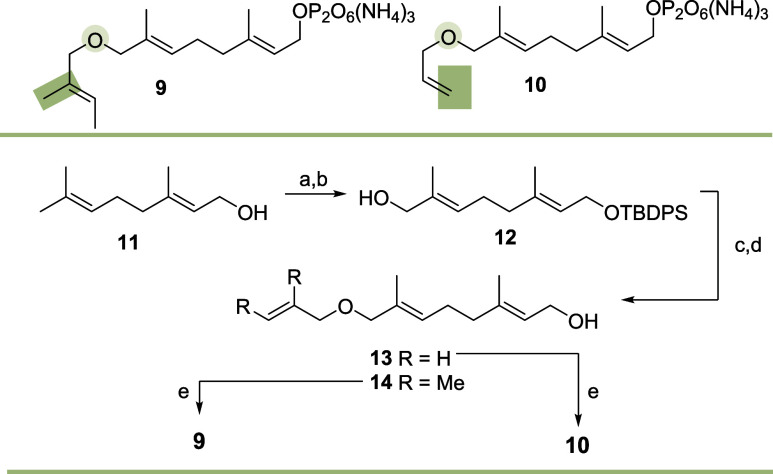
Syntheses of New FPP Derivatives **9** and **10** Starting from Geraniol (**11**) via Alcohol **12** Conditions: (a) imidazole, TBDPSCl, *N,N*-DMF, 0 to rt (97%); (b) SeO_2_, salicylic acid, water, *t*-BuOOH, CH_2_Cl_2_, 0 °C to rt, then NaBH_4_, MeOH, 0 °C (31%); (c), (d) **12** → **13**: NaH, allyl bromide, THF, 0 °C to rt (82%) then TBAF, THF, 0 °C to rt (93%); (c), (d) **12**→ **14**: NaH, tiglyl bromide, THF, 0 °C to rt, then TBAF, THF, 0 °C to rt (32% o2s); (e) for **13** NCS, DMS, CH_2_Cl_2_, 0 °C then ((*n*-Bu)_4_N)_3_P_2_O_7_H, MeCN, rt (82%) and for **14** NCS, DMS, CH_2_Cl_2_, −50 °C to −40 °C to 0 °C to rt, then ((*n*-Bu)_4_N)_3_P_2_O_7_H, MeCN, rt (quant.).

In the present work we add two new 9-oxy-FPP ether derivatives **9** and **10** to the substrate portfolio for STSs developed in our group so far.^17^ Structurally, these two FPP derivatives differ from ether **3** by modifications around the olefinic double bond at the aliphatic terminus. Their syntheses are briefly depicted in Scheme 2.

The syntheses of FPP derivatives **9** and **10** commences with the formation of intermediate alcohol **12** from geraniol (**11**).^6^ At this point the synthesis branches as different allyl bromides are employed in the Williamson ether synthesis to follow. For accessing ether alcohol **13** allyl bromide and for **14** (*E*)-1-bromo-2-methylbut-2-ene (tiglyl bromide) are used as electrophiles. After removal of the silyl protection the two alcohols **13** and **14** could be collected. Finally, these were transformed to the corresponding FPP derivatives **9** and **10,** respectively, following Poulter’s protocol.^18^

### Biotransformations and Structure Elucidation

With three different 9-oxy-FPP derivatives in hand, we treated these with several STSs as well as with the diterpene synthase (DTS) PvHVS1. These synthases were obtained as previously described.^6,17^ To our delight all derivatives where accepted and transformed by at least one synthase from the given list mentioned above (the outcome of all biotransformation experiments is found in Figures S4, S18 and S25). After evaluating the GC–MS data, the STSs BcBOT2, Omp7 and Tri5 and for the FPP derivative **3** PvHVS1 showed the most promising results. The decision to start semipreparative approaches was based not only on the quantities of new products formed, determined by semiquantitative evaluation of the GC–MS results, but also on the assessment of how easy it would be to purify individual products, i.e., whether mixed fractions could be avoided. Here, retention times in particular were included into the evaluation.

**Scheme 3:**
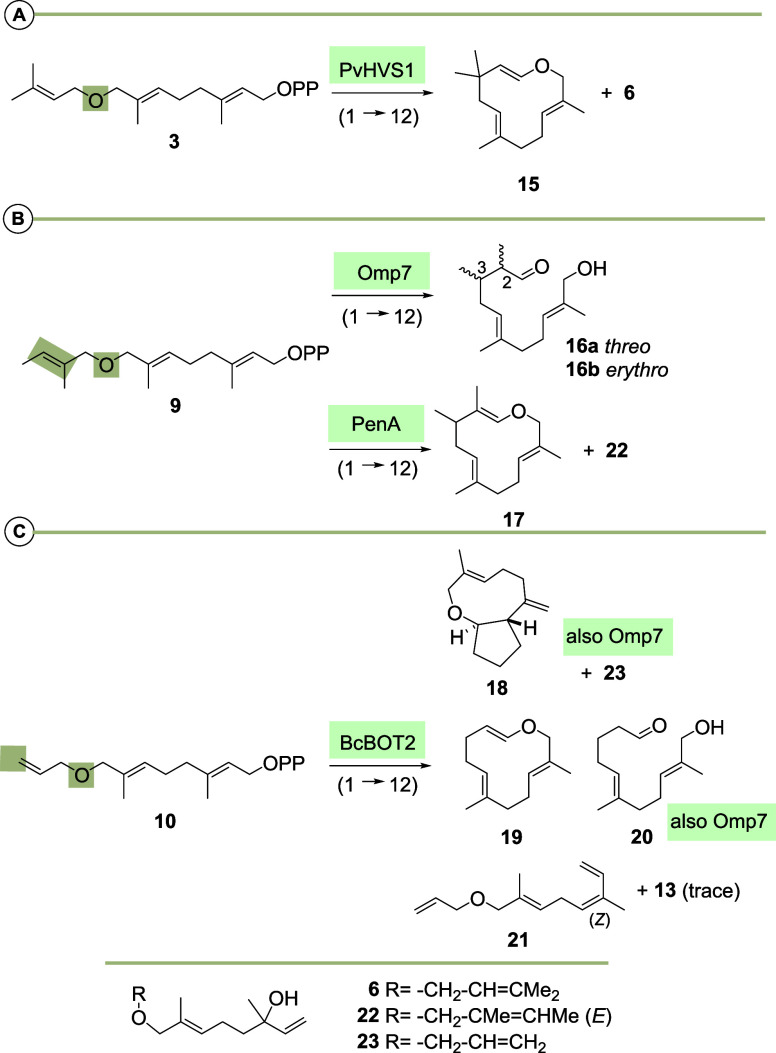
On the Formation of New Oxygenated Terpenoids by Different STSs: (A) Transformation with **3** Using PvHVS1; (B) Transformation with **9** Using Omp7 and PenA; (C) Transformation with **10** Using BcBOT2

The semipreparative biotransformation of ether derivative **3** (138 mg) with the DTS PvHVS1 (13.3 mg/mL, 257 μL) yielded macrocyclic enol ether **15** along with allyl alcohol **6** as byproduct (Scheme 3). FPP derivative **9** was well accepted by two STSs, namely Omp7 and PenA. With Omp7 (2.58 mg/mL, 0.97 mL and 2.86 mg/mL, 0.87 mL) two diastereomeric aldehydes **16a/b** (3 mg) were isolated while when PenA (11.2 mg/mL, 223 μL) was treated with **9** (34 mg for Omp7, 38 mg for PenA) the “hydrolysis” product **22** as well as the macrocyclic enol ether **17** (<1 mg) were isolated and characterized. The fact that two dia-steroemeric aldehydes **16a** and **16b** are formed suggests that **16a/b** are likely the “hydrolysis” products of enol ether **17**. This process does not proceed with lack of stereocontrol so that two stereogenic centers around the methyl branching points at C2 and C3 are created. The largest spectrum of new products was found with FPP ether derivative **10** (146 mg) when BcBOT2 (4.01 mg/mL, 5 mL) served as biocatalyst (case C, Scheme 3). The biotransformation was very effective and overall six products **18**–**21** and the “hydrolysis” products **13** and **23** could be isolated in sufficient amounts to achieve full characterization. While products **19** and **20** fit into the pattern of cyclizations observed oxy-FPP ether derivative **3** and **9**, respectively (see cases A and B, Scheme 3), product **18** bearing a cyclopentane ring annulated to a cyclic 9-membered ether represents a structurally unique terpenoid.

The structure elucidation of new biotransformation products mainly relied on ^1^H, ^13^C, DEPT135 and multiple 2D-NMR spectroscopic experiments. Structural determination of macrocyclic ethers **15**, **17** and **19** were rather straightforward. The olefinic protons of the enol ethers resonance at rather high field (δ= 4.79 + 5.63, 5.35, and 4.75 + 5.51 ppm for **15**, **17** and **19**). The corresponding ^13^C NMR signals for the enol ether moiety were also indicative with δ-values between 131.5 and 147.1 ppm. The configuration of the newly formed olefinic double bond was determined to be *E* for all enol ether macrocycles. This was confirmed by the absence of ^1^H–^1^H NOESY correlations between the olefinic protons of the 1,2-disubstituted alkene in **15** and **19**, respectively, or between the allylic methyl group and the olefinic proton in **17**.

The structure elucidation of aldehydes **16a**, **16b** and **20** commences from the aldehyde terminus and allowed the assignment of the whole carbon backbone by conducting ^1^H–^1^H COSY or ^1^H–^13^C HMBC NMR experiments. For aldehydes **16a** and **16b** the absolute configuration of the two stereogenic centers and the relative orientation of the two methyl groups could not be directly assigned. However, comparison of NMR spectra with literature-known aliphatic α,β-dimethyl branched aldehydes allowed assigning a *threo*/*erythro* ratio of 1.65:1.^19^

**Figure 1:**
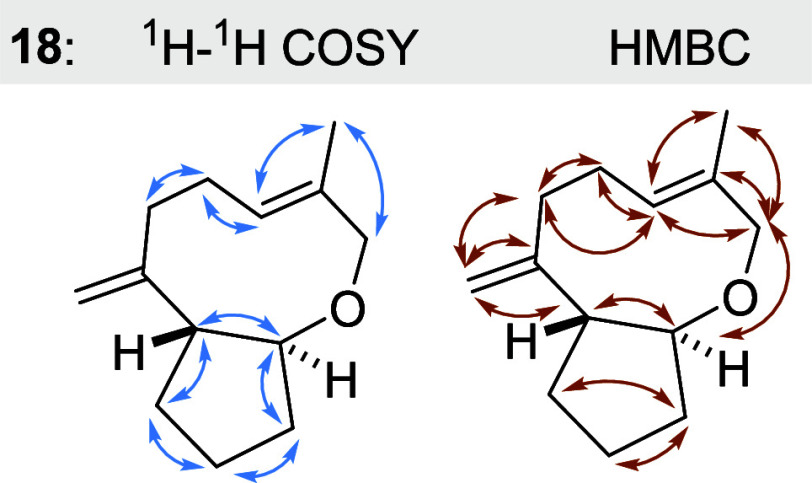
^1^H–^1^H COSY (blue) and HMBC (orange) correlations for bicyclic ether **18**. Noteworthy, a NOESY correlation between the two protons at stereogenic centers was not found; we therefore defined the relative orientation of the two protons to be *anti.*

For bicyclic terpenoid **18** the three methylene groups of the cyclo pentane ring served as starting point for the structure elucidation (Figure 1). The signals are well separated from the other methylene groups in the ^1^H NMR spectrum and are in the neighborhood of two methine moieties. The hydrogen atoms of the two CH groups at the sites of annulation were found to be *anti*-orientated as judged from NOESY correlation experiments. A more detailed description of the structure elucidation for all new terpenoids as well as the “hydrolysis” products is found in the Supporting Information.

### Mechanistic Considerations

**Scheme 4:**
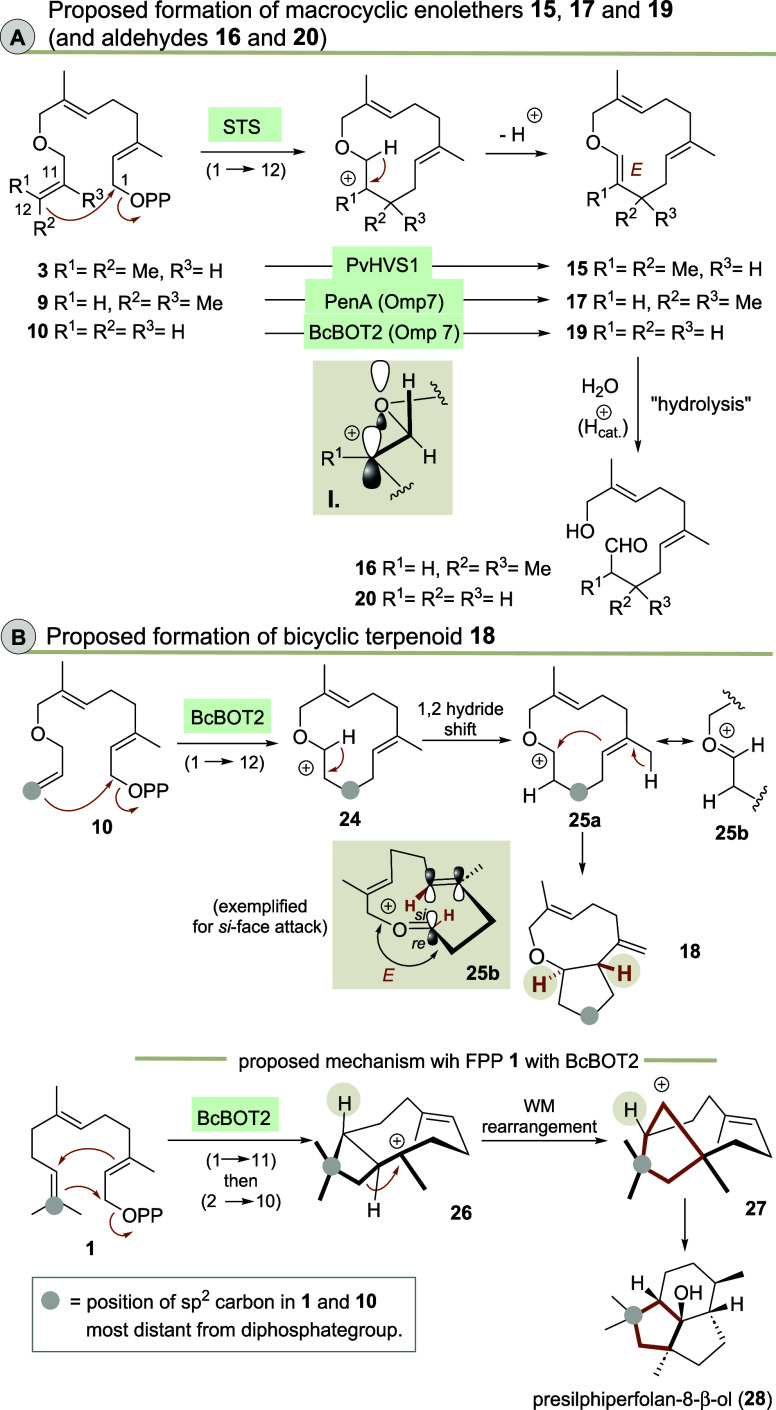
(A) Mechanistic Considerations on the Formation of Enol Ethers **15**, **17** and **19** and Aldehydes **16** and **20**, Respectively; (B) Proposed Mechanism on the Formation of Bicyclic Terpenoid **18** and Differences in Positioning of the Cyclopentane Moieties in **18** and Presilphiperfolan-8-β-ol (**28**), the Main Product Formed from **1** with BcBOT2

Mechanistically, the formation of macrocyclic enol ethers **15**, **17** and **19** can be explained by an initial 1 → 12 cyclization after activation of the diphosphate moiety and removal of a proton of the ether methylene group (Scheme 4).

As only (*E*)-configured enol ethers were formed one can propose a conformation that is based on stereoelectronic considerations. The hydroxy-aldehydes **16** and **20** can be regarded as “hydrolysis” products of the corresponding enol ethers which is initiated by protons or Mg^2+^ in an aqueous environment. This can either have happened in the active site of the STS or after release of the enol ether. As aldehydes were already present in the pentane fraction after extraction, it is very likely that their formation does not happen during purification. The formation of the bicyclic terpenoid **18** by BcBOT2 from the FPP derivative **10** can again be explained by a 1 → 12 cyclization, in which the cation **24** is transformed by a 1,2-hydride shift to the highly stabilized oxocarbenium ion **25a**/**b**. A transannulation reaction and final deprotonation provide the cyclopentane ring and the exocyclic methylene group and hence terpenoid **18**. In order to achieve transannulation the oxocarbeniom ion and the alkene moiety need to adopt a fixed conformation and the oxocarbenium ion needs to be (*E*)-configured. The absolute configuration of the newly formed stereogenic center is governed by the facial orientation of the two functional groups. For clarity reasons, the *si*-attack is arbitrarily chosen and depicted in Scheme 4B. Presilphiperfolan-8-β-ol (**28**) is the main product that is formed from FPP **1** by BcBOT2 via an initial 1 → 11 cyclization. It contains two cyclopentane rings that are annulated to a cyclohexane ring. The first cyclopentane ring is created by a ring-expanding Wagner–Meerwein rearrangement (MW rearrangement), which differs from the proposed cyclopentane formation to terpenoid **18**. Here, a 1,2-hydride shift occurs, which is probably favored by the additional oxygen atom that can perfectly stabilize the new carbocation. Essentially, selected terpene synthases including the diterpene synthase PvHVS1 are able to accept FPP derivatives with an additional atom at the aliphatic end of the prenyl pyrophosphates. All these TSs initiate cyclization between positions 1 and 12, unlike with natural FPP **1**.

### Biotransformations with BcBOT2 Mutants

We have noted before, that the substrate promiscuity toward unnatural FPP derivatives, is very pronounced for the STS BcBOT2 and this observation is also confirmed in this work. In recent work we generated a series of mutants of BcBOT2 based on a computational model.^20^ Therefore, we complemented the present study with a series of transformations using selected mutants. In the previous study, we identified aromatic amino acids Trp_118_, Phe_138_ and Tyr_211_ as part of the active pocket of BcBOT2, which affect the outcome of biotransformations with FPP **1** in such a way that transformation products are formed that structurally significantly differ from **28**.

**Scheme 5:**
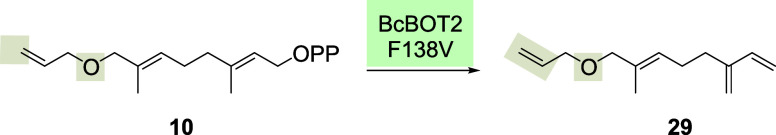
Biotransformation of FPP Ether Derivative **10** with the F138V Mutant of BcBOT2

**Figure 2:**
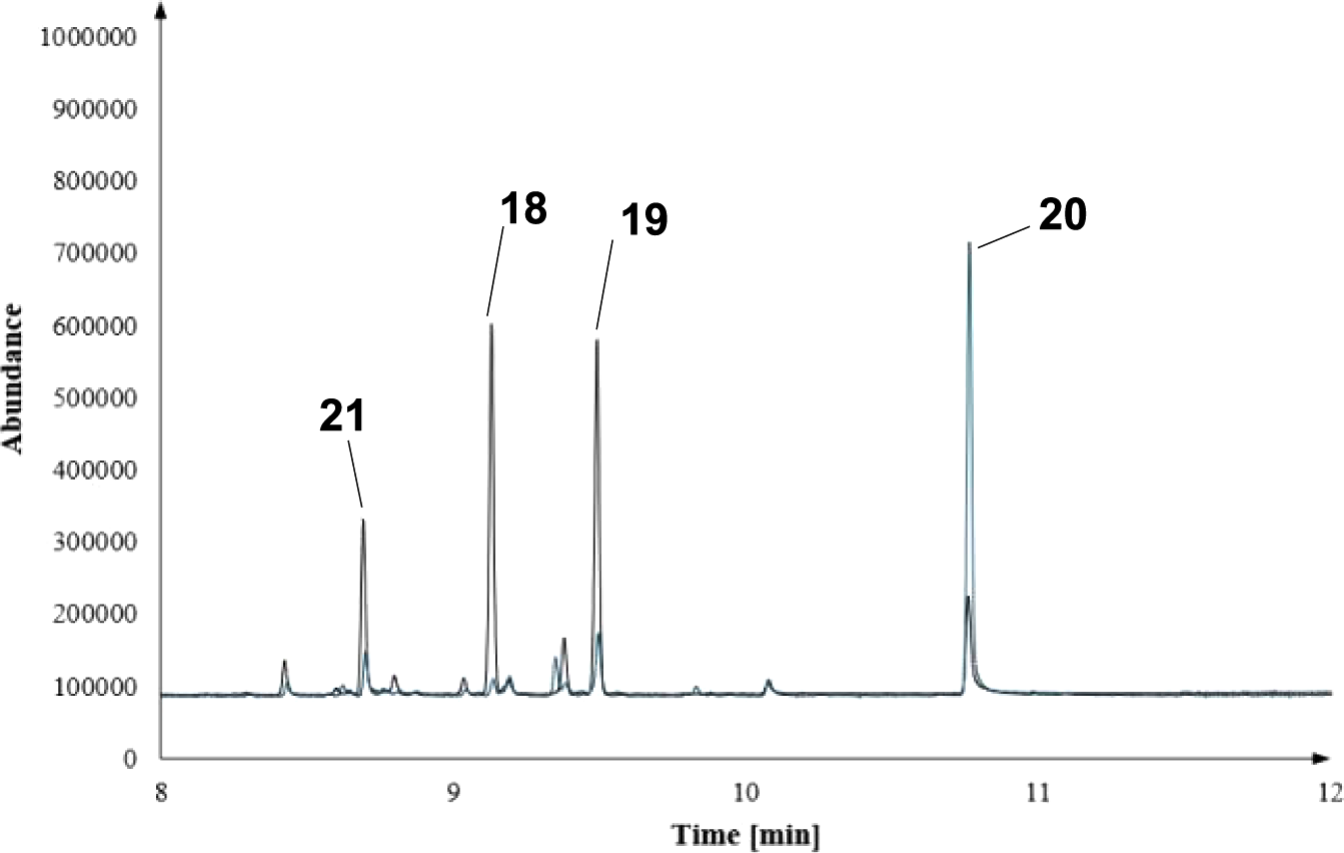
Zoom-in of an overlay for the biotransformation of FPP derivative **10** with BcBOT2 WT (black) or the Y211S mutant (blue).

**Figure 3:**
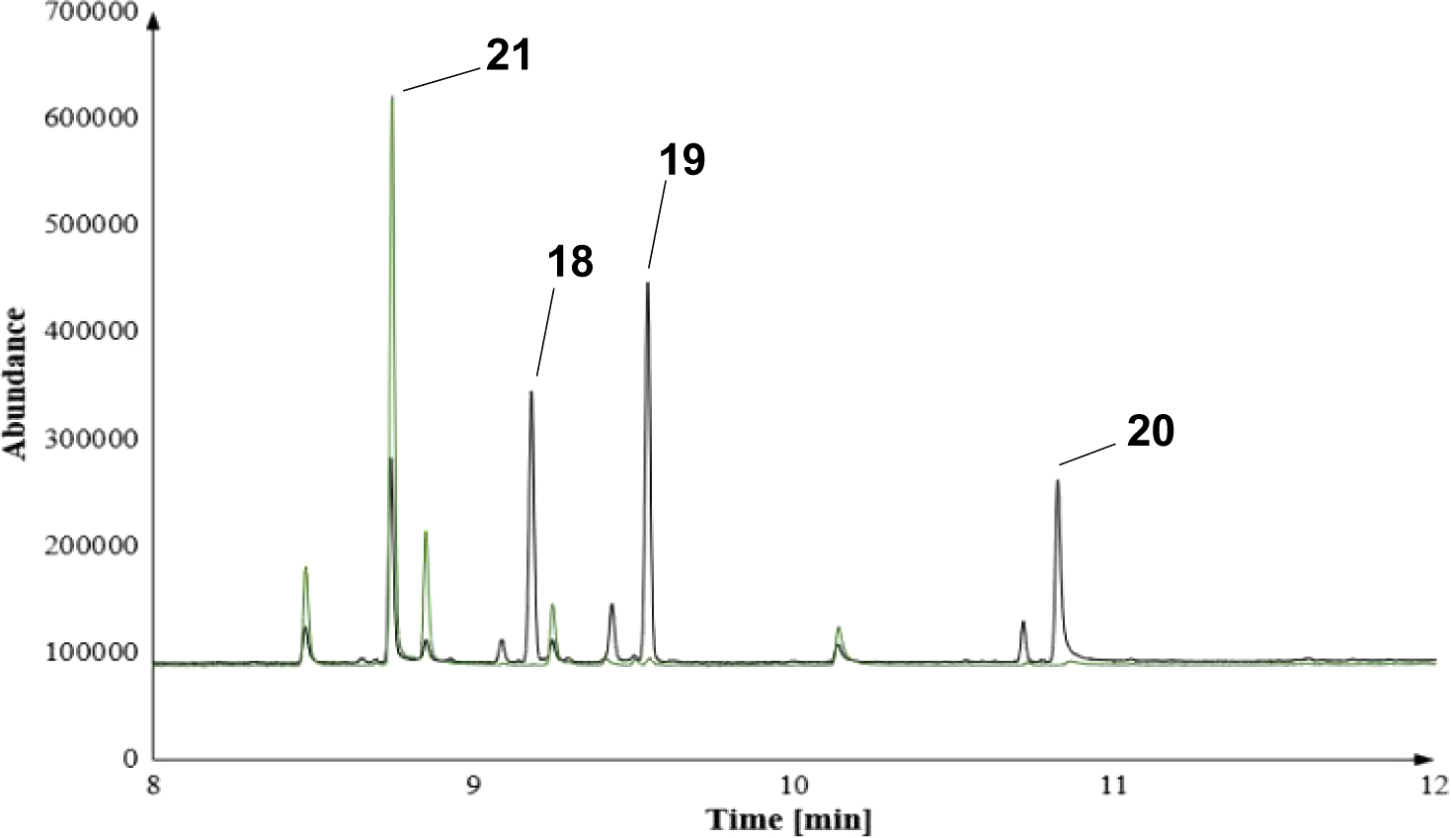
Zoom-in of an overlay for the biotransformation of FPP derivative **10** with BcBOT2 WT (black) or the W118Q mutant (green).

**Figure 4:**
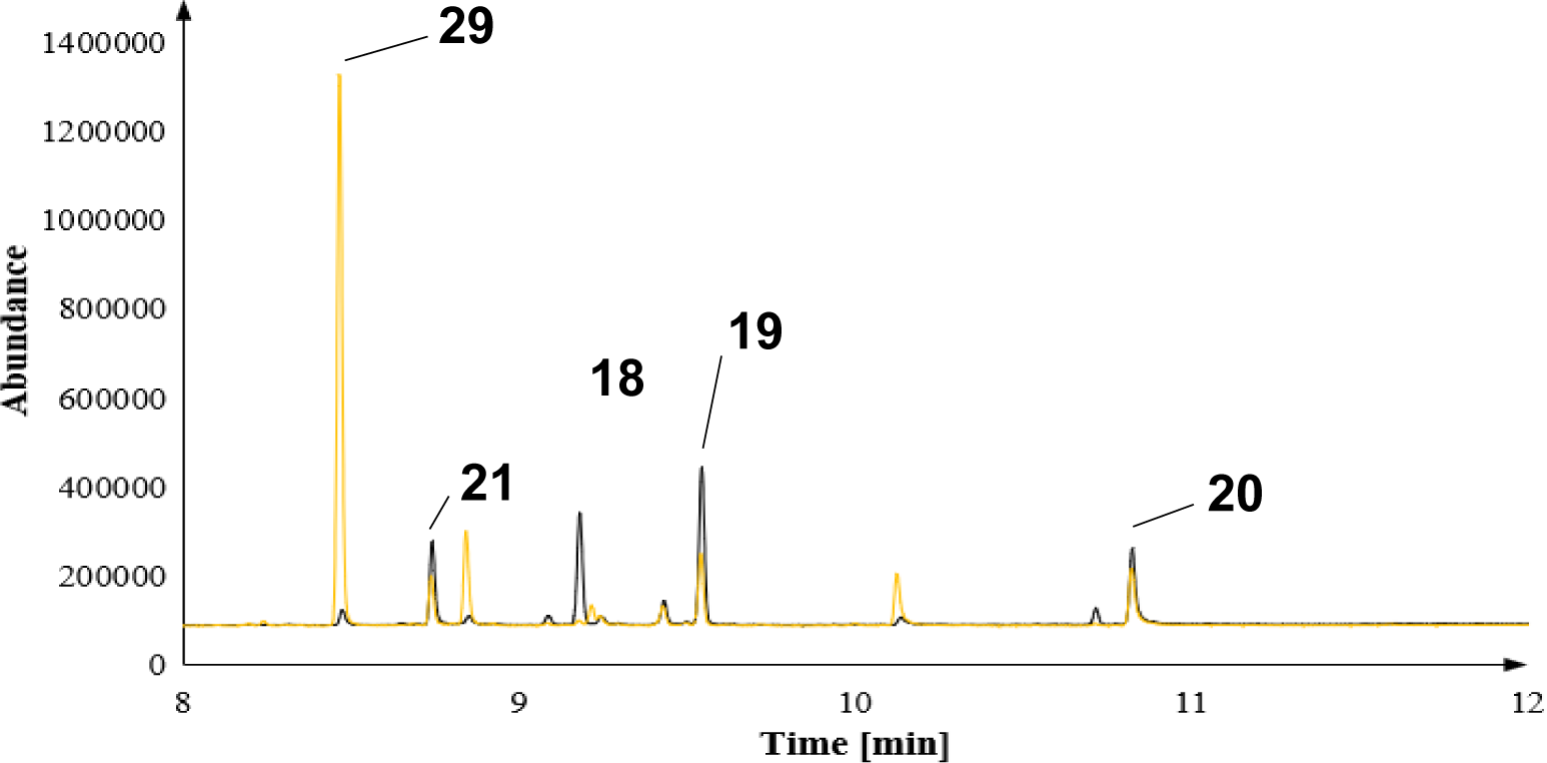
Zoom-in of an overlay for the biotransformation of FPP derivative **10** with BcBOT2 WT (black) or the F138V mutant (orange).

We screened the acceptance of these mutants for the FPP ether derivative **10** (see also Supporting Information) and the most significant outcome of these investigations are presented in Figures 2–4. As a general trend, we observed new product patterns, especially with regard to altered ratios of the individual products compared to wild-type BcBOT2. In the case of the F138V mutant (Figure 4), we were able to isolate triene **29** when FPP derivative **10** served as substrate (Scheme 5).

In fact, this product is also present in minute amounts (as judged by GC–MS) in the product mixture of wild-type BcBOT2. The mutation Y211S favors the formation of **20** (Figure 2), while the mutant W118Q leads to the preferential formation of the noncyclized product **21** (Figure 3). However, we could not detect the formation of new biotransformation products in this screening study.

## Conclusions

The present study expands the repertoire of FPP derivatives that can be accepted by different STSs and that are converted to new terpenoids. It is known that the insertion of an additional oxygen atom at position 9 of the backbone of FPP leads to poorer acceptance by STSs than when the oxygen atom is inserted at position 5. By changing the methylation pattern at the terminal alkene, we achieved improved acceptance of FPP ethers with an oxygen function at position 9, generating a total of four new ether-bearing cyclic terpenoids.

It appears that the elongation of the chain at the aliphatic end of FPP leads to difficulties in the correct folding of the substrate inside the active pocket of STSs. This can be compensated for if the two geminal methyl groups are omitted or shifted. The present work thus extends the possibility of expanding the terpenome by using unnatural FPP derivatives.

## Materials and Methods

### Microbiological Methods: Procedure A—Heterologous Protein Expression and Cell Lysis *via* Ultrasound

In order to cultivate the *E. coli* BL21 (DE3) cells, carrying the required plasmids, a seed culture (50 μL) was incubated with kanamycin (50 mg/mL, 3 μL) in LB-media (3 mL) for approximately 4.5 h at 37 °C and 200 rpm. Alternatively, a seed culture (5 μL) can be incubated with kanamycin (50 mg/mL, 5 μL) in LB-Media (5 mL) at 37 °C and 180 rpm o/n. From this preculture (1 mL) a main culture was created by incubation with kanamycin (50 mg/mL) in 2-TY media (50 mL) at 37 °C and 200 rpm until the culture reached an OD_600_ value of a 0.4 to 0.8. To initiate the protein overexpression IPTG (1 M, 25 or 50 μL) was added to the culture that was stirred at 16 °C and 180 rpm for approximately 22 h. After centrifugation, the cell pellets were stored at −20 °C or used immediately for cell lysis. Cells were resuspended in lysis buffer (20 mL) at 0 °C and lysed by ultrasonication (10 min, 45% amplitude, 4 s ultrasound to 6 s pause). The resulting solution was centrifuged (4 °C, 20 min, 10000 *g*) to give the crude enzyme solution. Immobilized metal-affinity chromatography: for conditioning the column was rinsed with water (10× the column volume) and lysis buffer (5× column volume). The lysate was loaded onto the column (2×) and eluted with Ni-NTA buffers (5 mL each) with increasing imidazole concentrations (25 mM, 50 mM, 100 mM, 250 mM, 500 mM). During this time the solutions were cooled at 0 °C. The fractions were analyzed using a qualitative Brentford assay and those fractions containing protein were united and concentrated by centrifugation (4 °C, 4500 rpm). Buffer exchange: to perform the buffer exchange the column was rinsed with water (10× column volume) and HEPES buffer (5× column volume). The protein solutions were loaded onto the column and eluted with HEPES buffer (5 mL). After centrifugation (4 °C, 4500 rpm) the solutions can be used or stored as a mixture of water and glycerol (1/1) between −70 °C and −80 °C. Concentration measurement: concentrations were determined by measuring the absorption (λ = 280 nm) of the purified protein solutions, using the extinction coefficient for reduced cysteine side chains. *In-vitro* biotransformation (analytical scale): screening for new biotransformation products was performed in a reaction scale of 500 μL containing the corresponding enzyme (50 μg), the FPP-derivatives **9** + **10** (1.5 μL, 50 mM) and a MgCl_2_ solution (1.25 μL, 2 M). In parallel also negative (without FPP derivative or in the absence of enzymes) as well as positive control experiments were performed (using FPP (**1**)) under analogous conditions. Reactions using FPP derivatives were carried out in HEPES buffer (pH = 7.5) at 37 °C and 100 rpm o/n. Positive controls using FPP (**1**) were performed at 30 °C and 100 rpm. In order to extract the products *n*-hexane was added and the phases were separated by centrifugation (3000 rpm, 6 min, 4 °C). The hexane extract was used for GC–MS analysis.

### Procedure B—Heterologous Protein Expression and Cell Lysis *via* Ultrasound

In order to cultivate the *E. coli* BL21 (DE3) cells, carrying the required plasmids, a seed culture (5 μL) was incubated with kanamycin (50 mg/mL, 5 μL) in LB-media (5 mL) at 37 °C and 200 rpm o/n. From this preculture (1 mL) a main culture was created by incubation with kanamycin (50 mg/mL, 50 μL) in 2-TY media (50 mL) at 37 °C and 200 rpm until the culture reached an OD_600_ value of 0.4 to 0.8. To initiate the protein overexpression IPTG (1 M, 50 μL) was added to the culture that was stirred at 16 °C and 180 rpm for approximately 22 h. After centrifugation, the cell pellets were stored at −20 °C or used immediately for cell lysis. Cells were resuspended in lysis buffer (15 mL) at 0 °C and lysed by ultrasonication (10 min, 45% amplitude, 4 s ultrasound to 6 s pause). The resulting solution was centrifuged (4 °C, 20 min, 10000 *g*) to give the crude enzyme solution. Immobilized metal-affinity chromatography: for conditioning the column was rinsed with water (10× the column volume) and lysis buffer (5× column volume). The lysate was loaded onto the column (2×) and eluted with Ni-NTA buffers (5 mL each) with two different imidazole concentrations (25 mM, 250 mM). During this time the solutions were cooled at 0 °C. The fractions were analyzed using a qualitative Brentford assay and those fractions containing protein were united and concentrated by centrifugation (4 °C, 4500 rpm). Buffer exchange: to perform the buffer exchange the column was rinsed with water (10× column volume) and HEPES buffer (5× column volume). The protein solutions were loaded onto the column and eluted with HEPES buffer (5 mL). After centrifugation (4 °C, 4500 rpm) the solutions can be used or stored as a mixture of water and glycerol (1/1) between −70 °C and −80 °C. Concentration measurement: concentrations were determined by measuring the absorption (λ = 280 nm) of the purified protein solutions, using the extinction coefficient for reduced cysteine side chains. *In-vitro* biotransformation (analytical scale): screening for new biotransformation products was performed in a reaction scale of 500 μL containing the corresponding enzyme (50 μg), the FPP-derivative **3** (1.5 μL, 50 mM) and a MgCl_2_ solution (1.25 μL, 2 M). In parallel also negative (without FPP derivative or in the absence of enzymes) as well as positive control experiments were performed (using FPP (**1**)) under analogous conditions. Reactions were carried out in HEPES buffer (pH = 7.5) for 30 min at 30 °C. In order to extract the products *n*-hexane was added and the phases were separated by centrifugation (3000 rpm, 8 min, 4 °C). The hexane extract was used for GC–MS analysis.

### Synthetic Procedures

**12** – SeO_2_ (168 mg, 1.51 mmol, 0.12 equiv) was suspended in CH_2_Cl_2_ (2 mL) and cooled to 0 °C. Salicylic acid (176 mg, 1.27 mmol, 0.10 equiv), water (2 drops) and *t*-BuOOH (5–6 M in decane, 9 mL, 45.0 mmol, 3.57 equiv) were added. Then silylether **S1** (4.95 g, 12.6 mmol, 1.00 equiv) dissolved in CH_2_Cl_2_ (2 mL + 6 mL) was added, the mixture was warmed to rt and stirred o/n. Acetone, EtOAc, an aq. sat. NaHCO_3_ solution and brine were added and the phases were separated. The aqueous phase was extracted with EtOAc (3×), the combined organic phases were dried over MgSO_4_·H_2_O, filtered and the solvent was removed *in vacuo*. The residue was taken up in methanol (20 mL) and cooled to 0 °C. NaBH_4_ (587 mg, 15.5 mmol, 1.23 equiv) was carefully added and the reaction stirred at 0 °C for 2 h before adding acetone and EtOAc. The organic phase was washed with an aq. sat. NaHCO_3_ solution and brine. Then it was dried over MgSO_4_·H_2_O, filtered and the solvent was removed *in vacuo*. The crude product was purified by coloumn chromatography (PE:EtOAc, 4:1) and alcohol **12** (1.60 g, 3.92 mmol, 31%) was obtained as a yellow oil. The analytical data are in accordance with those reported.^6^^a 1^H NMR (400 MHz, CDCl_3_): δ = 7.71–7.67 (m, 4H, H_Ar_), 7.44–7.35 (m, 6H, H_Ar_), 5.41–5.36 (m, 2H, H_3_, H_7_), 4.23–4.21 (m, 2H, H_8_), 3.99 (d, *J* = 5.8 Hz, 2H, H_1_), 2.15–2.10 (m, 2H, H_4_), 2.03–2.00 (m, 2H, H_5_), 1.67 (s, 3H, H_9_), 1.45 (s, 3H, H_10_), 1.22 (t, *J* = 6.0 Hz, 1H, H_OH_), 1.04 (s, 3H, H*_t_*_-Bu_) ppm; ^13^C NMR (101 MHz, CDCl_3_): δ = 136.8 (C_6_), 135.8 (C_Ar_), 135.1 (C_2_), 134.2 (C_Ar_), 129.7 (C_Ar_), 127.7 (C_Ar_), 126.0 (C_3_) 124.5 (C_7_), 69.2 (C_1_), 61.3 (C_8_), 39.2 (C_5_), 27.0 (C_*t*-Bu_), 26.0 (C_4_), 19.3 (C_*t*-Bu_), 16.5 (C_10_), 13.9 (C_9_) ppm; HRMS [ESI-MS]: *m*/*z* calcd for C_26_H_36_O_2_NaSi [M + Na]^+^: 431.2382, found: 431.2382.

NaH (60% in mineral oil, 139 mg, 3.48 mmol, 2.11 equiv) was suspended in THF (10 mL) and cooled to 0 °C. Alcohol **12** (676 mg, 1.65 mmol, 1.0 equiv) was dissolved in THF (5 mL + 5 mL) and added to the reaction mixture. After stirring for 1 h at 0 °C allyl bromide (0.29 mL, 405 mg, 3.35 mmol, 2.03 equiv) was added and stirring was continued for additional 10 min. The reaction mixture was warmed to rt and stirring was continued overnight. Then, an additional portion of NaH (60% in mineral oil, 79 mg, 1.98 mmol, 1.20 equiv) was added and the reaction mixture was stirred for 3 days. The reaction was terminated by addition of an aq. sat. NaHCO3 solution, followed by water and Et_2_O. The phases were separated and the aqueous phase was extracted with Et_2_O (3×) and the combined organic extracts were washed with an aq. sat. NaHCO_3_ solution, dried over MgSO_4_·H_2_O, filtered and the solvent was removed under reduced pressure. The crude product was purified by column chromatography (PE:EtOAc, 10:1) to yield ether **S2** (607 mg, 1.35 mmol, 82%) was obtained as a slight yellow oil. R_f_ = 0.56 (PE:EtOAc, 10:1); ^1^H NMR (400 MHz, CDCl_3_): δ = 7.70–7.68 (m, 4H, H_Ar_), 7.44–7.35 (m, 6H, H_Ar_), 5.96–5.86 (m, 1H, H_10_), 5.40–5.37 (m, 2H, H_2_, H_6_), 5.28–5.14 (m, 2H, H_11_), 4.22 (d, *J* = 6.4 Hz, 2H, H1), 3.90 (dt, *J* = 5.6 Hz, 1.4 Hz, 2H, H_9_), 3.85 (s, 3H, H_8_), 2.16–2.10 (m, 2H, H_5_), 2.03–2.00 (m, 2H, H_4_), 1.65 (s, 3H, H_13_), 1.44 (s, 3H, H_12_), 1.04 (s, 9H, H_*t*-Bu_) ppm; ^13^C NMR (101 MHz, CDCl_3_): δ = 136.8 (C_3_), 135.8 (C_Ar_), 135.2 (C_10_), 134.2 (C_7_), 132.4 (C_Ar_), 129.6 (C_Ar_), 128.0 (C_6_), 127.7 (C_Ar_), 124.4 (C_2_), 116.9 (C_11_), 76.4 (C_8_), 70.6 (C_9_), 61.3 (C_1_), 39.2 (C_4_), 27.0 (C_*t*-Bu_), 26.1 (C_5_), 19.3 (C_*t*-Bu_), 16.4 (C_12_), 14.1 (C_13_) ppm; HRMS [ESI-MS]: *m*/*z* calcd for C_29_H_40_O_2_NaSi [M + Na]+: 471.2695, found: 471.2682.

**13** – ether **S2** was dissolved in THF (20 mL) and cooled to 0 °C. TBAF (1 M in THF, 2.00 mL, 2.00 mmol, 1.49 equiv) was added and the reaction stirred at 0 °C for 30 min before stirring at rt o/n. Then water, EtOAc and brine were added and the phases were separated. The aqueous phase was extracted with EtOAc (3×) and the combined organic phases were dried over MgSO_4_·H_2_O, filtered and the solvent was removed *in vacuo*. The crude product was purified by column chromatography (PE:EtOAc, ≈3:1) and alcohol **13** (263 mg, 1.25 mmol, 93%) was obtained as a pale yellow oil. ^1^H NMR (400 MHz, CDCl_3_): δ = 5.97–5.87 (m, 1H, H_10_), 5.43–5.37 (m, 2H, H_2_, H_6_), 5.29–5.15 (m, 2H, H_11_), 4.15 (d, *J* = 6.8 Hz, 2H, H_1_), 3.92 (dt, *J* = 5.6 Hz, 1.2 Hz, 2H, H_9_), 3.85 (s, 2H, H_8_), 2.21–2.15 (m, 2H, H_5_), 2.09–2.06 (m, 2H, H_4_), 1.68 (s, 3H, H_12_), 1.65 (s, 3H, H_13_) ppm; ^13^C NMR (101 MHz, CDCl_3_): δ = 139.4 (C_3_), 135.1 (C_10_), 132.7 (C_7_), 127.7 (C_6_), 123.9 (C_2_), 117.0 (C_11_), 76.3 (C_8_), 70.7 (C_9_), 59.5 (C_1_), 39.2 (C_4_), 26.1 (C_5_), 16.4 (C_12_), 14.1 (C_13_) ppm; HRMS [ESI-MS]: *m*/*z* calcd for C_13_H_22_O_2_Na [M + Na]^+^: 233.1517, found: 233.1510.

**10** – NCS (179 mg, 1.34 mmol, 1.88 equiv) was suspended in CH_2_Cl_2_ (10 mL) and cooled to 0 °C. DMS (0.90 mL, 0.76 g, 1.22 mmol, 1.71 equiv) was added and the reaction stirred at 0 °C for 10 min before adding alcohol **13** (150 mg, 0.71 mmol, 1.00 equiv) dissolved in CH_2_Cl_2_ (5 mL + 5 mL). After stirring for about 45 min at 0 °C, brine and *n*-pentane were added and the phases were separated. The aqueous phase was extracted with *n*-pentane (3×), the combined organic phases were dried over MgSO_4_·H_2_O, filtered and the solvent was carefully removed *in vacuo*. The crude product was purified by coloumn chromatography (*n*-pentane:Et_2_O, 100:1 → 30:1) and the product was used for the next step without further analysis. Tris(tetra-*n*-butylammonium) hydrogen pyrophosphate (1.36 g, 1.51 mmol, 2.12 equiv) was dissolved in MeCN (10 mL) and the chloride was added as a solution in MeCN (5 mL + 5 mL). The reaction stirred at rt o/n before the solvent was removed *in vacuo*. The residue was loaded onto the ion exchange coloumn as described in the General Information. FPP derivative **10** (246 mg, 0.58 mmol, 82%) was obtained as a fluffy solid. ^1^H NMR (400 MHz, CDCl_3_): δ = 5.95 (ddt, *J* = 17.1 Hz, 10.6 Hz, 6.1 Hz, 1H, H_10_), 5.52–5.44 (m, 2H, H_2_, H_6_), 5.34–5.24 (m, 2H, H_11_), 4.47 (t, *J* = 6.7 Hz, 2H, H_1_), 3.97 (dt, *J* = 6.0 Hz, 1.3 Hz, 2H, H_9_), 3.94 (s, 2H, H_8_), 2.24–2.20 (m, 2H, H_5_), 2.16–2.12 (m, 2H, H_4_), 1.72 (s, 3H, H_12_), 1.65 (s, 3H, H_13_) ppm; ^13^C NMR (101 MHz, CDCl_3_): δ = 142.5 (C_3_), 133.9 (C_10_), 131.7 (C_7_), 129.7 (C_6_), 119.9 (d, C_2_), 118.3 (C_11_), 76.0 (C_8_), 70.0 (C_9_), 62.6 (d, C_1_), 38.3 (C_4_), 25.3 (C_5_), 15.5 (C_12_), 13.2 (C_13_) ppm; HRMS [ESI-MS]: *m*/*z* calcd for C_13_H_23_O_8_P_2_ [M-(NH_4_)_3_+H_2_]^−^: 369.0868, found: 369.0872.

**14** – NaH (60% in mineral oil, 113 mg, 2.83 mmol, 2.17 equiv) was suspended in THF (10 mL) and cooled to 0 °C. Alcohol **12** (530 mg, 1.30 mmol, 1.00 equiv) dissolved in THF (5 mL + 5 mL) was added and the deprotonation was stirred for 1 h at 0 °C. Bromide **S4** (296 mg, 1.99 mmol, 1.53 equiv) was added with THF (1 mL) and after additional 10 min at 0 °C, the reaction mixture was warmed to rt. After stirring o/n additional NaH (60% in mineral oil, 66 mg, 0.95 mmol,1.67 mmol, 1.28 equiv) was added and after 3 d at rt, an aq. sat. NH_4_Cl solution, water and Et_2_O were added. The phases were separated and the aqueous phase was extracted with Et_2_O (3×). The combined organic phases were washed with an aq. sat. NaHCO_3_ solution, dried over MgSO_4_·H_2_O, filtered and the solvent was removed *in vacuo*. The crude product was purified by coloumn chromatography (PE:EtOAc, 10:1) to yield the intermediate C_15_ ether with impurities. Therefore the product (256 mg, 0.54 mmol, 1.00 equiv) was dissolved in THF (16 mL) and cooled to 0 °C. TBAF (1 M in THF, 0.81 mL, 0.81 mmol, 1.51 equiv) was added and the reaction stirred for 30 min at 0 °C, before stirring at rt o/n. Water was added and the phases were separated. The aqueous phase was extracted with EtOAc (3×) and the combined organic phases were dried over MgSO_4_·H_2_O, filtered and the solvent was removed *in vacuo*. The crude product was purified by column chromatography (PE:EtOAc, 3:1) and alcohol **14** (100 mg, 0.42 mmol, 78%, 32% o.2.s.) was obtained as a yellow oil. ^1^H NMR (400 MHz, CDCl_3_): δ = 5.50–5.44 (m, 1H, H_11_), 5.43–5.39 (m, 1H, H_2_), 5.39–5.34 (m, 1H, H_6_), 4.14 (d, *J* = 6.8 Hz, 2H, H_1_), 3.77 (m, 4H, H_8_, H_9_), 2.20–2.14 (m, 2H, H_5_), 2.09–2.05 (m, 2H, H_4_), 1.68 (s, 3H, H_13_), 1.64 (s, 6H, H_14_, H_15_), 1.63–1.61 (m, 3H, H_12_) ppm; ^13^C NMR (101 MHz, CDCl_3_): δ = 139.5 (C_3_), 133.2 (C_7_/C_10_), 132.9 (C_7_/C_10_), 127.5 (C_6_), 123.8 (C_2_), 122.5 (C_11_), 75.9 (C_8_/C_9_), 75.8 (C_8_/C_9_), 59.5 (C_1_), 39.2 (C_4_), 26.1 (C_5_), 16.4 (C_13_), 14.1 (C_14_/C_15_), 13.8 (C_14_/C_15_), 13.3 (C_12_) ppm; HRMS [ESI-MS]: *m*/*z* calcd for C_15_H_26_O_2_Na [M + Na]^+^: 261.1831, found: 261.1833.

**9** – NCS (30 mg, 0.22 mmol, 1.88 equiv) was dissolved in CH_2_Cl_2_ (3 mL) and cooled to −50 °C to −40 °C. DMS (20 μL, 17 mg, 0.27 mmol, 2.25 equiv) was added and the reaction stirred at 0 °C for 5 min before being cooled to −50 °C to −40 °C again. Alcohol **14** (29 mg, 0.12 mmol, 1.00 equiv) was dissolved in CH_2_Cl_2_ (1 mL + 1 mL) and added to the reaction. Shortly after the reaction was warmed to 0 °C where it stirred for 1 h before adding additional NCS (44 mg, 0.33 mmol, 2.75 equiv) and DMS (0.05 mL, 42 mg, 0.68 mmol, 5.63 equiv). After 30 min the reaction was warmed to rt and after 15 min brine was added. The mixture was diluted with water and *n*-penante, the phases were separated and the aqueous phase was extracted with *n*-pentane (3×). The combined organic phases were dried over MgSO_4_·H_2_O, filtered and the solvent was removed *in vacuo* (up to 500 mbar). Tris(tetra-*n*-butylammonium) hydrogen pyrophosphate (235 mg, 0.26 mmol, 2.17 equiv) was dissolved in MeCN (3 mL) and the chloride was added as a solution in MeCN (2 mL + 1 mL). The reaction stirred at rt o/n before the solvent was removed *in vacuo*. The residue was loaded onto the ion exchange coloumn as described in the General Information. FPP derivative **9** (67 mg, 0.15 mmol, quant.) was obtained as a white yellow solid. ^1^H NMR (400 MHz, D_2_O): δ = 5.60–5.54 (m, 1H, H_11_), 5.51–5.46 (m, 2H, H_6_, H_2_), 4.48 (dd, *J* = 6.6 Hz, 6.6 Hz, 2H, H_1_), 3.87 (s, 2H, H_8_), 3.85 (s, 2H, H_9_), 2.26–2.21 (m, 2H, H_5_), 2.16–2.12 (m, 2H, H_4_), 1.73 (s, 3H, H_13_), 1.66–1.62 (m, 9H, H_12_, H_14_, H_15_) ppm; ^13^C NMR (151 MHz, D_2_O): δ = 142.4 (C_3_), 132.4 (C_10_), 131.9 (C_7_), 129.6 (C_6_), 124.6 (C_11_), 120.1 (d, C_2_), 75.3 (C_8_), 75.2 (C_9_), 62.4 (d, C_1_), 38.4 (C_4_), 25.4 (C_5_), 15.6 (C_13_), 13.2 (C_14_/C_15_), 13.0 (C_14_/C_15_), 12.5 (C_12_) ppm; HRMS [ESI-MS]: *m*/*z* calcd for C_15_H_27_O_8_P_2_ [*M*-(NH_4_)_3_+H_2_]^−^: 397.1181, found: 397.1175.

**3** – alcohol **S6** (150 mg, 0.63 mmol, 1.00 equiv) was dissolved in THF (6 mL) and cooled to 0 °C. Et_3_N (0.20 mL, 1.47 mmol, 2.33 equiv) and MsCl (0.10 mL, 1.26 mmol, 2.00 equiv) were added dropwise and the reaction stirred at 0 °C for 1 h. Then LiCl (123 mg, 2.89 mmol, 4.60 equiv) was added and the reaction stirred for 45 min at 0 °C and 30 min at rt. Water was added and the phases were separated. The aqueous layer was extracted with PE (3×) and the combined organic phases were washed with brine, dried over MgSO_4_·H_2_O, filtered and the solvent was removed *in vacuo*. The allylic chloride was obtained as a colorless oil and used in the next reaction without further purification. The chloride was dissolved in MeCN (4 mL) and added to a solution of tris(tetra-*n*-butylammonium) hydrogen pyrophosphate (1.12 g, 1.25 mmol, 2.00 equiv) and 3 Å-sieves in MeCN (4 mL). The mixture stirred at rt o/n. The solvent was removed *in vacuo* and the residue was loaded onto the ion exchange coloumn as described in the General Information and FPP derivative **3** (250 mg, 0.56 mmol, 89%) was obtained as a white solid. The analytical data is in accordance with the literature.^6^^a 1^H NMR (400 MHz, D_2_O): δ = 5.55–5.42 (m, 2H, H_2_, H_6_), 5.41–5.33 (m, 1H, H_10_), 4.49 (dd, *J* = 6.5 Hz, 6.5 Hz, 2H, H_1_), 3.96 (d, *J* = 7.4 Hz, 2H, H_9_), 3.92 (s, 2H, H_8_), 2.28–2.19 (m, 2H, H_5_), 2.19–2.12 (m, 2H, H_4_), 1.77 (s, 3H, H_12_/H_15_), 1.74 (s, 3H, H_13_), 1.69 (s, 3H, H_12_/H_15_), 1.66 (s, 3H, H_14_) ppm; ^13^C NMR (101 MHz, D_2_O): δ = 142.8 (C_3_), 140.4 (C_11_), 131.8 (C_7_), 129.7 (C_6_), 119.7 (d, *J* = 7.8 Hz, C_2_), 119.1 (C_10_), 75.7 (C_8_), 65.1 (C_9_), 62.9 (d, *J* = 4.7 Hz, C_1_), 38.3 (C_4_), 25.4 (C_6_), 24.9 (C_12_/C_15_), 17.2 (C_12_/C_15_), 15.5 (C_13_), 13.2 (C_14_) ppm.

### Biotransformations

**18–21** – FPP derivative **10** (146 mg, 0.35 mmol, 1.00 equiv) was dissolved in an aq. NH_4_HCO_3_ (0.05 M, 17.3 mL) solution as a stock solution. The following reaction was performed in four 50 mL batches, the procedure will be described for one 50 mL batch. Tween 20 (10 μL) and PPase (1 μL) were dissolved in HEPES buffer (44.5 mL). The derivative **10** stock solution (500 μL) and BcBOT2 solution (4.01 mg/mL, 624 μL) were added and the reaction was started by adding MgCl_2_ (2 M, 250 μL), while shaking at 100 rpm and 37 °C. Every 30 min more derivative **10** stock solution (500 μL) was added to a total volume of 4 mL. After 120 min a second batch of BcBOT2 solution (624 μL) was added. The reaction then continued o/n at 37 °C and 100 rpm, before separating the phases after the addition of *n*-pentane. The aqueous phase was extracted with *n*-pentane (3×) and the combined organic phases were washed with brine, dried over MgSO_4_·H_2_O and the solvent was removed *in vacuo*. The crude product was purified by column chromatography (*n*-pentane:Et_2_O, 30:1 → 1:1) (isolation of **20**) and nonpolar fractions were collected and purified by multiple coloumn chromatographies (*n*-pentane: Et_2_O, 100:1) until the different compounds were separated and isolated (**18**, **19** and **21**). The NMR samples were prepared by coevaporation with C_6_D_6_ resulting in yields not being determined, as the previously unknown products were considered potentially volatile. Analytical data of **18**: ^1^H NMR (600 MHz, C_6_D_6_): δ = 5.50 (dd, *J* = 9.1 Hz, 1H, H_3_), 4.77 (d, *J* = 5.0 Hz, 2H, H_13_), 4.12 (d, *J* = 10.5 Hz, 1H, H_1_), 3.58–3.56 (m, 2H, H_1_, H_11_), 2.34–2.27 (m, 1H, H_4_), 2.14–2.03 (m, 3H, H_5_, H_7_), 1.97–1.86 (m, 3H, H_4_, H_8_, H_10_), 1.80–1.74 (m, 1H, H_10_), 1.67 (s, 3H, H_12_), 1.66–1.61 (m, 1H, H_9_), 1.55–1.51 (m, 1H, H_9_), 1.35–1.27 (m, 1H, H_8_) ppm; ^13^C NMR (151 MHz, C_6_D_6_): δ = 155.7 (C_6_), 134.5 (C_2_), 130.7 (C_3_), 112.2 (C_13_), 87.6 (C_11_), 78.8 (C_1_), 58.0 (C_7_), 36.3 (C_8_), 35.7 (C_10_), 34.8 (C_5_), 26.6 (C_4_), 24.8 (C_9_), 16.7 (C_12_) ppm; HRMS [GC–MS, CI]: *m*/*z* calcd for C_13_H_20_O [M]^+^: 192.1514, found: 192.1512. Analytical data of **19**: ^1^H NMR (600 MHz, C_6_D_6_): δ = 5.51 (d, *J* = 12.1 Hz, 1H, H_11_), 4.93–4.90 (m, 1H, H_3_), 4.75 (dt, *J* = 12.1 Hz, 7.6 Hz, 1H, H_10_), 4.69 (t, *J* = 6.9 Hz, 1H, H_7_), 3.87 (s, 2H, H_1_), 2.04–2.00 (m, 4H, H_4_, H_5_), 1.92–1.85 (m, 4H, H_8_, H_9_), 1.49 (s, 3H, H_12_), 1.36 (s, 3H, H_13_) ppm; ^13^C NMR (151 MHz, C_6_D_6_): δ = 147.1 (C_11_), 133.8 (C_6_), 131.8 (C_2_), 131.2 (C_3_), 127.2 (C_7_), 109.7 (C_10_), 77.7 (C_1_), 39.8 (C_5_), 27.9 (C_8_), 27.4 (C_9_), 25.1 (C_4_), 15.2 (C_13_), 14.5 (C_12_) ppm; HRMS [GC–MS, EI]: *m*/*z* calcd for C_13_H_20_O [M]^+^: 192.1514, found: 192.1510. Analytical data of **20**: ^1^H NMR (600 MHz, C_6_D_6_): δ = 9.32 (t, *J* = 1.7 Hz, 1H, H_1_), 5.35 (tq, *J* = 10.5 Hz, 1.3 Hz, 1H, H_9_), 5.05–5.02 (m, 1H, H_5_), 3.81 (s, 2H, H_11_), 2.13–2.09 (m, 2H, H_8_), 2.03–2.00 (m, 2H, H_5_), 1.86–1.81 (m, 4H, H_2_, H_4_), 1.56 (s, 3H, H_13_), 1.48 (s, 3H, H_12_), 1.40 (tt, *J* = 7.2 Hz, 7.2 Hz, 2H, H_3_) ppm; ^13^C NMR (151 MHz, C_6_D_6_): δ = 201.1 (C_1_), 135.9 (C_6_), 135.6 (C_10_), 124.9 (C_9_), 124.2 (C_5_), 68.6 (C_11_), 43.2 (C_2_), 39.8 (C_7_), 27.5 (C_4_), 26.3 (C_8_), 22.4 (C_3_), 16.0 (C_12_), 13.7 (C_13_) ppm; HRMS [GC–MS, CI]: *m*/*z* calcd for C_13_H_22_O_2_ [M]^+^: 210.1620, found: 210.1618. Analytical data of **21**: ^1^H NMR (600 MHz, C_6_D_6_): δ = 6.82 (dd, *J* = 17.3 Hz, 10.8 Hz, 1H, H_2_), 5.91–5.82 (m, 1H, H_10_), 5.42 (t, *J* = 6.9 Hz, 2H, H_6_), 5.33 (t, *J* = 7.6 Hz, 1H, H_4_), 5.27–5.24 (m, 1H, H_11_), 5.20–5.17 (d, *J* = 17.2 Hz, 1H, H_1_), 5.06 (d, *J* = 10.9 Hz, 1H, H_1_), 5.04 (m, 1H, H_11_), 3.79–3.75 (m, 4H, H_8_, H_9_), 2.85 (dd, *J* = 7.3 Hz, 7.3 Hz, 2H, H_5_), 1.76 (s, 3H, H_12_), 1.62 (s, 3H, H_13_) ppm; ^13^C NMR (151 MHz, C_6_D_6_): δ = 135.8 (C_10_), 133f.9 (C_2_), 133.2 (C_7_), 132.8 (C_3_), 129.2 (C_4_), 125.7 (C_6_), 115.9 (C_11_), 113.9 (C_1_), 76.1 (C_8_), 70.6 (C_9_), 26.4 (C_5_), 19.9 (C_12_), 14.0 (C_13_) ppm; HRMS [GC–MS, CI]: *m*/*z* calcd for C_13_H_20_O [M]^+^: 192.1514, found: 192.1517.

**13 + 20 + 23** – FPP derivative **10** (86 mg, 0.20 mmol, 1.00 equiv) was dissolved in water (10.9 mL) to prepare a stock solution. The following reaction was performed in five 25 mL batches, the procedure will be described for 25 mL batch. Tween 20 (5 μL) and PPase (0.5 μL) were dissolved in HEPES buffer (22.1 mL). The derivative **10** stock solution (250 μL) and Omp7 solution (3.3 mg/mL, 380 μL) were added and the reaction was started by adding MgCl_2_ (2 M, 125 μL) at 37 °C and 100 rpm. Every 30 min more derivative **10** stock solution (250 μL) was added to a total volume of 4 mL. After 120 min a second batch of Omp7 solution (3.3 mg/mL, 376 μL) was added. The reaction continued o/n at 37 °C and 100 rpm, before separating the phases after adding *n*-pentane. The aqueous phase was extracted with *n*-pentane (3×) and the combined organic phases were washed with brine and were exposed to an ultrasonic bath to enhance the phase separation. After drying over MgSO_4_·H_2_O and filtration, the solvent was very carefully removed *in vacuo*. The crude product was purified by coloumn chromatography (*n-*pentane:Et_2_O, 5:1 → 4:1 → 3:1) and the isolated fractions were coevaporated with C_6_D_6_ for NMR measurements. The oily products were weighed before adding C_6_D_6_, therefore likely containing solvent residues at this point: **23**: 7 mg, **13**: 195 mg, **20**: 8 mg. Spectroscopic data of **23** is used from a different biotransformation using Tri5, resulting in the same product as mentioned above.

Analytical data of **23**: ^1^H NMR (400 MHz, C_6_D_6_): δ = 5.88 (ddt, *J* = 17.2 Hz, 10.5 Hz, 5.2 Hz, 1H, H_10_), 5.72 (dd, *J* = 17.3 Hz, 10.7 Hz, 1H, H_2_), 5.44 (tq, *J* = 10.9 Hz, 1.3 Hz, 1H, H_6_), 5.29 (ddt, *J* = 17.2 Hz, 1.8 Hz, 1.8 Hz, 1H, H_11_), 5.18 (dd, *J* = 17.3 Hz, 1.6 Hz, 1H, H_1_), 5.06 (ddt, *J* = 10.5 Hz, 1.9 Hz, 1.5 Hz, 1H, H_11_), 4.94 (dd, *J* = 10.7 Hz, 1.6 Hz, 1H, H_1_), 3.82 (ddd, *J* = 5.2 Hz, 1.6 Hz, 1.6 Hz, 2H, H_9_), 3.79 (s, 2H, H_8_), 2.17–2.00 (m, 2H, H_5_), 1.65 (s, 3H, H_13_), 1.51–1.39 (m, 2H, H_4_), 1.09 (s, 3H, H_12_) ppm; ^13^C NMR (101 MHz, C_6_D_6_): δ = 145.5 (C_2_), 135.8 (C_10_), 132.8 (C_7_), 128.1 (C_6_), 115.9 (C_11_), 111.5 (C_1_), 76.3 (C_8_), 72.9 (C_3_), 70.6 (C_9_), 42.2 (C_4_), 28.3 (C_12_), 22.7 (C_5_), 14.0 (C_13_) ppm; HRMS [GC–MS, EI]: *m*/*z* calcd for C_13_H_20_O [M-H_2_O]^+^: 192.1514, found: 192.1517.

**16a/b** – FPP derivative **9** (34 mg, 75.7 μmol, 1.00 equiv) was dissolved in an aq. NH_4_HCO_3_ solution (0.05 M, 4.07 mL). This solution was added to a mixture of HEPES buffer (43.9 mL), Tween20 (10 μL) and PPase (1 μL). Omp7 enzyme solution (2.58 mg/mL, 0.97 mL) and then MgCl_2_ solution (2 M, 250 μL) was addded to start the reaction. After 2 h at 100 rpm and 37 °C another batch of Omp7 enzyme solution (2.86 mg/mL, 0.87 mL) was added. After 4 h of total reaction time *n*-pentane (25 mL) was added and the mixture was allowed to shake o/n. After the addition of *n*-pentane, the phases were separated (if problems with the separation occur, ultrasonic baths can help), the aqueous phase was extracted with *n*-pentante (3×) and the combined organic phases were dried over MgSO_4_·H_2_O, filtered and the solvent was carefully removed. The crude product was purified by coloumn chromatography (*n*-pentane:Et_2_O, 1:1) and aldehydes **16a** and **16b** (3 mg) were obtained as a yellowish oil and a mixture of two diastereomers. ^1^H NMR (400 MHz, C_6_D_6_): δ = 9.40 (d, *J* = 1.5 Hz, 1H, H_1b_), 9.39 (d, *J* = 1.4 Hz, 1H, H_1a_), 5.38–5.30 (m, 2H, H_9a_, H_9b_), 5.09–5.03 (m, 2H, H_5a_, H_5b_), 3.85 (s, 2H, H_11b_), 3.80 (s, 2H, H_11a_), 2.14–2.00 (m, 8H, H_2a_, H_7a_, H_7b_, H_8a_, H_8b_), 1.92–1.66 (m, 7H, H_2b_, H_3a_, H_3b_, H_4a_, H_4b_), 1.57 (s, 3H, H_15b_), 1.55 (s, 3H, H_15a_), 1.49 (s, 3H, H_14a_), 1.48 (s, 3H, H_14b_), 0.82 (d, *J* = 7.0 Hz, 3H, H_12b_), 0.80 (d, *J* = 7.0 Hz, 3H, H_12a_), 0.77 (d, *J* = 6.8 Hz, 3H, H_13b_), 0.66 (d, *J* = 6.7 Hz, 3H, H_13a_) ppm; ^13^C NMR (101 MHz, C_6_D_6_): δ = 204.0 (C_1a_), 204.0 (C_1b_), 136.5 (C_6b_), 136.4 (C_6a_), 135.6 (C_10b_), 135.5 (C_a_), 124.8 (C_9a_), 124.8 (C_9b_), 123.4 (C_5a_), 123.2 (C_5b_), 68.6 (C_11b_), 68.6 (C_11a_), 51.0 (C_2b_), 50.1 (C_2a_), 39.9 (C_7a_), 39.9 (C_7b_), 34.8 (C_3b_), 33.6 (C_3a_), 33.4 (C_4a_), 31.8 (C_4b_), 26.3 (C_8a_), 26.1 (C_8b_), 17.9 (C_13b_), 16.1 (C_14a_, C_14b_), 15.6 (C_13a_), 13.7 (C_15b_), 13.7 (C_15a_), 9.7 (C_12b_), 8.5 (C_12a_) ppm; HRMS [GC–MS, CI]: *m*/*z* calcd for C_15_H_27_O_2_ [M + H]^+^: 239.2011, found: 239.2010.

**17 + 22** – FPP derivative **9** (38 mg, 84.6 μmol, 1.00 equiv) was dissolved in an aq. NH_4_HCO_3_ solution (0.05 M, 4.52 mL). The reaction was performed in two 25 mL batches. The following procedure will be described for one 25 mL batch. Tween20 (10 μL) and PPase (0.5 μL) were dissolved in HEPES buffer (22.6 mL). FPP derivative **9** (250 μL) and PenA enzyme solution (11.2 mg/mL, 111 μL) were added and the reaction was started by the addition of MgCl_2_ (2 M, 125 μL). The reaction was stirred at 37 °C and 100 rpm and every 30 min more FPP derivative **9** (250 μL) was added to a total volume of 2 mL. After 2 h another batch of PenA enzyme solution (112 μL) was added. The reaction was then allowed to stirr at 37 °C and 100 rpm o/n, before adding *n*-pentane. The phases were separated and the aqueous phase was extracted with *n*-pentane (3×). The combined organic phases were dried over MgSO_4_·H_2_O, filtered and the solvent was carefully removed *in vacuo*. The crude product was purified by coloumn chromatography (*n-*pentane:Et_2_O, 50:1, 9:1, 3:1, 1:1) and the isolated compounds were prepared by C_6_D_6_ coevaporation for NMR analysis, still showing residues of *n*-pentane and Et_2_O. **22** and **17** were obtained in small amounts of <1 mg. Additionally traces of **16a/b** were isolated, the corresponding data is shown above. Analytical data of **22**: ^1^H NMR (600 MHz, C_6_D_6_): δ = 5.72 (dd, *J* = 17.3 Hz, 10.7 Hz, 1H, H_2_), 5.53–5.47 (m, 2H, H_6_, H_11_), 5.18 (dd, *J* = 17.3 Hz, 1.5 Hz, 1H, H_1_), 4.94 (dd, *J* = 10.7 Hz, 1.5 Hz, 1H, H_1_), 3.81 (s, 2H, H_9_), 3.80 (s, 2H, H_8_), 2.18–2.04 (m, 2H, H_5_), 1.69 (s, 3H, H_14_), 1.66 (s, 3H, H_15_), 1.53 (d, *J* = 6.8 Hz, 3H, H_12_), 1.51–1.40 (m, 2H, H_4_), 1.09 (s, 3H, H_13_) ppm; ^13^C NMR (151 MHz, C_6_D_6_): δ = 145.6 (C_2_), 134.0 (C_10_), 133.2 (C_7_), 127.6 (C_6_), 121.7 (C_11_), 111.6 (C_1_), 76.0 (C_9_), 75.9 (C_8_), 73.0 (C_3_), 42.4 (C_4_), 28.4 (C_13_), 22.9 (C_5_), 14.2 (C_14_), 13.8 (C_15_), 13.3 (C_12_) ppm; HRMS [GC–MS, CI]: *m*/*z* calcd for C_15_H_25_O [M-H_2_O+H]^+^: 221.1905, found: 221.1899. Analytical data of **17**: ^1^H NMR (600 MHz, C_6_D_6_): δ = 5.35 (q, *J* = 1.4 Hz, 1H, H_1_), 4.89 (dd, *J* = 10.2 Hz, 3.8 Hz, 1H, H_9_), 4.79–4.77 (m, 1H, H_5_), 4.14 (d, *J* = 11.3 Hz, 1H, H_11_), 3.65 (d, *J* = 11.2 Hz, 1H, H_11_), 2.22–2.15 (m, 1H, H_8_), 2.10–2.03 (m, 3H, H_3_, H_4_, H_7_), 1.95 (ddd, *J* = 12.6 Hz, 12.6 Hz, 3.9 Hz, 1H, H_7_), 1.87–1.83 (m, 1H, H_8_), 1.80–1.78 (m, 1H, H_4_), 1.54 (s, 3H, H_12_), 1.54 (s, 3H, H_15_), 1.39–1.38 (m, 3H, H_14_), 1.02 (d, *J* = 6.6 Hz, 3H, H_13_) ppm; ^13^C NMR (151 MHz, C_6_D_6_): δ = 141.3 (C_1_), 133.3 (C_6_), 131.7 (C_10_), 131.5 (C_9_), 127.2 (C_5_), 119.7 (C_2_), 78.2 (C_11_), 40.0 (C_7_), 38.2 (C_3_), 33.8 (C_4_), 25.2 (C_8_), 19.4 (C_13_), 15.0 (C_14_), 14.7 (C_15_), 8.7 (C_12_) ppm; HRMS [GC–MS, CI]: *m*/*z* calcd for C_15_H_24_O [M]^+^: 220.1827, found: 220.1825.

**6** – FPP derivative **3** (137 mg, 305 μmol, 1.00 equiv) was dissolved in an aq. NH_4_HCO_3_ solution (14 mM, 21.8 mL). The reaction was performed in five 41 mL batches. The following procedure will be described for one 41 mL batch. Tween20 (8 μL) was dissolved in HEPES_2 buffer (36.1 mL). FPP derivative **3** (500 μL) and PvHVS enzyme solution (24.3 mg/mL, 167.1 μL) were added. The reaction was stirred at 29 °C and 50 rpm and every 30 min more FPP derivative **3** (500 μL, last addition: 400 μL) was added to a total volume of 4.4 mL. After the last addition, PPase (1 μL) was added. The reaction was then allowed to stirr at 29 °C and 50 rpm o/n. The batches were combined and extracted with *n*-pentane (3×). The combined organic phases were dried over MgSO_4_·H_2_O, filtered and the solvent was carefully removed *in vacuo*. The crude product was purified by column chromatography (*n-*pentane:Et_2_O, 100:1, 5:1) and the isolated compound were prepared by C_6_D_6_ coevaporation for NMR analysis. Analytical data of **6**, literature known for CDCl_3_.^6^^a 1^H NMR (600 MHz, C_6_D_6_): δ = 5.72 (dd, *J* = 17.3 Hz, 10.7 Hz, 1H, H_2_), 5.54–5.52 (m, 1H, H_10_), 5.49 (t, *J* = 7.2 Hz, 1H, H_6_), 5.18 (d, *J* = 17.3 Hz, 1H, H_1_), 4.94 (d, *J* = 10.8 Hz, 1H, H_1_), 3.96 (d, *J* = 6.7 Hz, 2H, H_9_), 3.85 (s, 2H, H_8_), 2.16–2.06 (m, 2H, H_5_), 1.70 (s, 3H, H_14_), 1.60 (s, 3H, H_12_/H_15_), 1.52 (s, 3H, H_12_/H_15_), 1.49–1.40 (m, 2H, H_5_), 1.09 (s, 3H, H_13_) ppm; ^13^C NMR (151 MHz, C_6_D_6_): δ = 145.5 (C_2_), 135.3 (C_11_), 133.2 (C_7_), 127.6 (C_6_), 122.8 (C_10_), 111.5 (C_1_), 76.1 (C_8_), 72.9 (C_3_), 66.5 (C_9_), 42.3 (C_4_), 28.3 (C_13_), 25.7 (C_12_/C_15_), 22.8 (C_5_), 18.0 (C_12_/C_15_), 14.1 (C_14_) ppm; HRMS [GC–MS, CI]: *m*/*z* calcd for C_15_H_24_O [M-H_2_O]^+^: 220.1827, found: 220.1830.

**15** – FPP derivative **3** (138 mg, 307 μmol, 1.00 equiv) was dissolved in an aq. NH_4_HCO_3_ solution (14 mM, 21.9 mL). The reaction was performed in six 34 mL batches. The following procedure will be described for one 34 mL batch. Tween20 (7 μL) was dissolved in HEPES_2 buffer (30.2 mL). FPP derivative **3** (500 μL) and PvHVS enzyme solution (13.3 mg/mL, 257 μL) were added. The reaction was stirred at 29 °C and 50 rpm and every 30 min more FPP derivative **3** (500 μL, last addition: 200 μL) was added to a total volume of 3.7 mL. After the last addition, PPase (1 μL) was added. The reaction was then allowed to stirr at 29 °C and 50 rpm o/n. The batches were combined and extracted with *n*-pentane (3×). The combined organic phases were dried over MgSO_4_·H_2_O, filtered and the solvent was carefully removed *in vacuo*. The crude product was purified by column chromatography (*n-*pentane:Et_2_O 400:1, 100:1) and the isolated compound were prepared by C_6_D_6_ coevaporation for NMR analysis. ^1^H NMR (600 MHz, C_6_D_6_): δ = 5.63 (d, *J* = 12.5 Hz, 1H, H_1_), 5.02 (t, *J* = 7.6 Hz, 1H, H_5_), 4.91 (t, *J* = 7.8 Hz, 1H, H_9_), 4.79 (d, *J* = 12.4 Hz, 1H, H_2_), 3.91 (s, 2H, H_11_), 2.06–2.02 (m, 4H, H_7_, H_8_), 1.89 (d, *J* = 7.7 Hz, 2H, H_4_), 1.51 (s, 3H, H_15_), 1.38 (s, 3H, H_14_), 1.02 (s, 6H, H_12_, H_13_) ppm; ^13^C NMR (151 MHz, C_6_D_6_): δ = 143.8 (C_1_), 134.2 (C_6_), 131.1 (C_10_), 131.0 (C_9_), 124.3 (C_5_), 119.9 (C_2_), 76.9 (C_11_), 42.0 (C_4_), 39.9 (C_7_), 35.0 (C_3_), 28.2 (C_12_, C_13_), 25.2 (C_8_), 15.2 (C_14_), 14.5 (C_15_) ppm; HRMS [GC–MS, CI]: *m*/*z* calcd for C_15_H_24_O [M]^+^: 220.1827, found: 220.1826.

**29** – FPP derivative **10** (134 mg, 697 μmol, 1.00 equiv) was dissolved in an aq. NH_4_HCO_3_ solution (0.05 mM, 16 mL). The reaction was performed in four 50 mL batches. The following procedure will be described for one 50 mL batch. Tween20 (10 μL), PPase (1 μL) and MgCl_2_ (2 M, 250 μL) were dissolved in HEPES buffer (45.5 mL). FPP derivative **10** solution (500 μL) and BcBot2 F138 Vsolution (20.7 mg/mL, 121 μL) were added. The reaction was stirred at 37 °C and 100 rpm. Every 30 min more FPP derivative **10** solution (500 μL) was added to a total volume of 4.0 mL. Halfway through the period more enzyme solution (20.7 mg/mL, 121 μL) was added. The reaction was then allowed to stirr at 37 °C and 100 rpm o/n. The batches were combined and extracted with *n*-pentane (3×). The combined organic phases were washed with an aq. sat. NaCl solution, dried over MgSO_4_·H_2_O, filtered and the solvent was carefully removed *in vacuo* and under a stream of N_2_. The crude product (9 mg) was purified by column chromatography (*n-*pentane:Et_2_O 100:1, 45:1) which resulted in a non separable mixture of compounds with one major product. In order to isolate this compound we used a preparative gas chromatography (see General Information). ^1^H NMR (600 MHz, C_6_D_6_): δ = 6.34 (dd, *J* = 17.6 Hz, 10.6 Hz, 1H, H_2_), 5.88 (ddt, *J* = 17.2 Hz, 10.5 Hz, 5.3 Hz, 1H, H_10_), 5.45–5.42 (m, 1H, H_6_), 5.28 (ddt, *J* = 17.2 Hz, 1.8 Hz, 1.8 Hz, 1H, H_11_), 5.17 (d, *J* = 17.5 Hz, 1H, H_1_), 5.07–5.04 (m, 1H, H_11_), 4.97–4.95 (m, 3H, H_1_, H_12_), 3.81 (dt, *J* = 5.2 Hz, 1.5 Hz, 2H, H_9_), 3.79 (s, 2H, H_8_), 2.21 (m, 4H, H_4_, H_5_), 1.61 (s, 3H, H_13_) ppm; ^13^C NMR (151 MHz, C_6_D_6_): δ = 146.2 (C_3_), 139.3 (C_2_), 135.8 (C_10_), 133.2 (C_7_), 127.2 (C_6_), 116.1 (C_12_), 115.9 (C_11_), 113.2 (C_1_), 76.2 (C_8_), 70.5 (C_9_), 31.5 (C_4_), 26.6 (C_5_), 14.0 (C_13_) ppm; HRMS [GC–MS, CI]: *m*/*z* calcd for C_13_H_21_O [M + H]^+^: 193.1592, found: 193.1600.

## Supporting Information Available

Detailed chemical procedures, biotransformations and spectral data as well as copies of NMR spectra (PDF)

## Abbreviations

TBDPS: *tert-*butyldiphenylsilyl
tiglyl: Me-CH=CMe-R (*E*)
NCS: *N*-chloro succinimide
DMS: dimethysulfide
