# Supplementary material for: Chemoenzymatic Formation of Oxa-Terpenoids by Sesqui- and Diterpene Synthase-Mediated Biotransformations with 9-Oxy-FPP Ether Derivatives
Source: Biochemistry. 2024 Dec 28;64(2):498–508. doi: 10.1021/acs.biochem.4c00589 (PMC11756643; doi:10.1021/acs.biochem.4c00589)
Supplement: Supplementary file 1 — bi4c00589_si_001.pdf [file bi4c00589_si_001.pdf]

Supporting Information

Chemoenzymatic formation of oxa-terpenoids by sesqui- and diterpene synthase-mediated biotransformations with 9-oxy-FPP ether derivatives

Henry Struwe[a], Trang Nguyen[a], Svenja Schwörer[a], Jörn Droste[a], Hanke Spinck[a], Andreas Kirschning*[a,b]

[a] *Institute of Organic Chemistry, Leibniz University Hannover, Schneiderberg 1B, 30167 Hannover, Germany*

[b] *Uppsala Biomedical Center (BMC), University Uppsala, Husargatan 3, 752 37 Uppsala, Sweden*

Email: andreas.kirschning@oci.uni-hannover.de; orcid.org/0000-0001-5431-6930

Table of Contents

1. **Materials and methods**

1.1 General information

1.2 Synthesis

1.2.1 Chemical Synthesis

1.2.2 Biotransformations

1.3 Microbiological methods and biotransformations

1.3.1 Procedure A

1.3.2 Procedure B

1.4 GC-MS Data

1.4.1 Positive and Negative controls

1.4.2 Biotransformation using FPP derivative **10**

1.4.3 Biotransformation using FPP derivative **9**

1.4.4 Biotransformation using FPP derivative **3**

1.4.5 Biotransformations using BcBot2 mutants and **10**

1.4.6 Retention index of isolated products

1.5 Structure elucidations

1.5.1 Structure elucidation of **18**

1.5.2 Structure elucidation of **21**

1.5.3 Structure elucidation of **19**

1.5.4 Structure elucidation of **20**

1.5.5 Structure elucidation of **23**

1.5.6 Structure elucidation of **16a** and **16b**

1.5.7 Structure elucidation of **22**

1.5.8 Structure elucidation of **17**

1.5.9 Structure elucidation of **15**

1.5.10 Structure elucidation of **6**

1.5.11 Structure elucidation of **29**

2. **Copies of NMR Spectra**

3. **References (supporting information)**

1. Materials and methods

1.1 General information

The experimental procedures described below, in which water is neither a reactant nor a solvent, were carried out in pre-dried flasks under an inert gas atmosphere. To ensure homogeneity, the reaction mixtures were stirred with a magnetic stirrer. The temperatures given refer to the bath vessels used. Temperatures of -78 °C were achieved with an acetone-dry ice mixture, -41 °C with MeCN-dry ice mixtures and 0 °C with a water-ice mixture. The term room temperature does not refer to a specific value here, but varies with the ambient temperature. Water baths, oil baths, NaCl-ice mixtures or a cryostat were used for different temperatures.

Unless otherwise stated, dry solvents were used for each reaction in which a pre-dried flask was used. Tetrahydrofuran, dichloromethane, N,N-dimethylformamide, acetonitrile, and diethyl ether were obtained as dry solvents from a Braun solvent purification system or as dry solvents from Acros Organics or Thermos Scientific. Tetrahydrofuran was also used when freshly distilled. Deuterated solvents were purchased from Deutero GmbH.

Column chromatography was performed using silica gel obtained from Macherey-Nagel (particle size 40-63 µm). The chromatographic purifications were carried out under adjusted pressures. The solvents/mixtures used are listed in the corresponding experimental procedures. Thin layer chromatography (TLC) was performed with pre-coated TLC sheets ALUGRAM® Xtra SIL G/UV_{254} foil from Macherey-Nagel (layer: 0.20 mm silica gel 60 with fluorescent indicator UV_{254}). Reagents containing vanillin or $KMnO_4$ were used as coloring solutions. In selected cases UV light (λ = 254 nm/ 366 nm) was employed to identify compounds on TLC. The Rf values given are subject to certain inaccuracies due to measurement limitations, but can be used as an initial guide. The volumes given as a total refer to the amount of solvent used for the initial reaction solution and any additional solvent added to rinse the glassware (e.g. solvent (2 mL + 2 mL)).

NMR data (^{1}H, ^{13}C, ^{31}P, DEPT135, 2D-NMR) were recorded on the following spectrometers: Bruker AVANCE I (ν_L(^{1}H)= 400 MHz) equipped with a DUL probe, Bruker AVANCE III HD (ν_L(^{1}H)=400 MHz) equipped with either a PRODIGY BBFO or BBO probe, Bruker AVANCE III HD (ν_L(^{1}H)=500 MHz) equipped with a TCI cryoprobe, and Bruker AVANCE NEO (ν_L(^{1}H)=600 MHz) equipped with DUL cryoprobe. All probes were equipped with z-Gradient coils. The choice of deuterated solvents is given in the respective procedures ($CDCl_3$, C_6D_6, D_2O). The analysis was performed using the Bruker Topspin® software. The residual solvent signal of the deuterated solvents was used to calibrate the chemical shift scale of the NMR spectra. Chemical shifts δ are given in ppm, J coupling constants are given in Hz and were determined manually or with appropriate software functions. The abbreviations used here for multiplicities are s (singlet), d (doublet), t (triplet), q (quartet), qi (quintet), and m (multiplet). When a particular signal could not be unambiguously assigned to a particular position, e.g. because signals overlapped, the indices in question were separated by "/" to indicate that there were several possibilities. Structural assignments were made with additional information from HSQC, COSY, HMBC and 1D-NOE/NOESY experiments

HR-ESI-MS (Tof) analysis was performed with Aliance 2695 HPLC (Waters) coupled to a LCT premier (Waters) with a lock spray dual ion ESI source. GC-EI-MS (quadrupole) was measured on a 6890 GC (Agilent) / 5973 MSD (Hewlett Packard) (column: Optima WAX) and a 7890B GC (Agilent) / 5977B MSD (Agilent) (column: Optima5HT). HR-CI-MS (Tof) was performed with a 6890 GC + (Hewlett Packard) coupled to GCT Premier (Waters) equipped with a CI ion source. The GC systems were additionally equipped with a FI detector.

Compound isolation on a preparative GC was performed with GC system HP6890 Series Plus coupled to a HP7683 Series Injector and a Gerstel PFC with cooling traps at −3 °C (Thermos Scientific A40). The stationary phase is a Zebron ZB1: 30 m, 0.53 mm, 3.00 µm. Temperature program: Starting temp. 100 °C; 20 °C/min for 3 min to 160 °C; 4 °C/min for 13.75 min to 215 °C holding for 1.5 min, 40 °C/min for 1.375 min to 280 °C.

Ion exchange of tetra-*n*-butylammonium to ammonium cations was performed using DOWEX®50WX8 resin as column material. For this purpose, the resin was rinsed with an aqueous HCl solution (3 M) until the eluent was acidic (pH paper). It was then rinsed with H_2O to a near neutral pH value, with NH_4OH (6%) to an alkaline pH, and finally with ion exchange buffer (980 mL H_2O, 20 mL *i*-PrOH, 2 g NH_4HCO_3) to a pH of 8 to 9. The residues collected from the reactions were then uploaded onto the column and eluted with ion exchange buffer. The product-containing fractions, analyzed by $KMnO_4$-TLC staining, were combined and the solvent was removed in vacuo. The residue was diluted in an aqueous NH_4HCO_3 solution (0.05 M) and freeze-dried. To remove the inorganic ammonium pyrophosphate salt, the product was dissolved in an aqueous NH_4HCO_3 solution (0.05 M, 2 mL) and mixed with MeCN/*i*-PrOH (1/1, 8 mL). After centrifugation (5000 rpm, 10 min, 4 °C), the liquid was collected and the procedure was repeated. After removal of the solvent in vacuo, the residue was dissolved in an aqueous NH_4HCO_3 solution (0.05 M) and freeze-dried. The product was stored at temperatures between −70 °C and −80 °C. The diphosphate salts were analyzed by ^{1}H-, ^{13}C- and by ^{31}P-NMR spectroscopy. HRMS measurements and determination of R_f values could sometimes not be performed on these salts.

Experiments with living microorganisms were performed in either S1 or S2 laboratories. Sterile work was performed using a Thermos Scientific laminar flow cabinet (type 2020). Optical density (OD600) was measured at 600 nm using a photometer from FoodALYT. Cell lysis was performed using a SONOPULS ultrasonic homogenizer from Bandelin (Procedure A) and Sonopuls GM2070 from Bandelin (Procedure B). IMAC was performed with column material from Macherey-Nagel GmbH & Co. KG containing Ni ions, such as Ni-NTA. Buffer exchange was performed using a column from GE Healthcare AB (V= 8.3 mL). A Merck KGaA filter unit with an exclusion limit of 30000 Da was used to constrict protein solutions. The extinction coefficient for protein concentration measurement was determined using ProtParam (ExPASy). UV/Vis spectroscopy was performed at 280 nm using a DeNovix spectrophotometer (type: DS-11+). GC-MS analyses for in vitro biotransformations were performed using an Agilent 5977B GC/MSD with 7890B GC system and an Optima 5HT - 0.25 µm, carrier gas: He, column volume: 30 m x 250 µm x 0.25 µm, injection volume 1 µL. Retention indices (RI) were determined in comparison to *n*-alkanes (C7 to C30). Differences in measurement dates of a sample and *n*-alkanes were within a short time period but not necessarily performed on the same day.

Composition of buffer solutions and culture media

In order to set pH value for the buffer solutions, aqueous NaOH and HCl solutions were used.

Lysis buffer:	Tris·HCl (40 mM), NaCl (100 mM)
Ni-NTA buffer (x M):	Tris·HCl (40 mM), NaCl (100 mM), Imidazol (x M)
HEPES buffer:	HEPES (50 mM), DTT (5 mM), pH =7.5
HEPES_2 buffer:	HEPES (50 mM), DTT (5 mM), NaCl (50 mM), $MgCl_2$ (10 mM) pH =7.5
LB media:	0.50% (w/v) yeast extract (Duchefa Biochemie)
	1.00% (w/v) trypton (Duchefa Biochemie)
	0.50% (w/v) NaCl (Roth or VWR)
2-TY media:	1.00% (w/v) yeast extract (Duchefa Biochemie)
	1.60% (w/v) tryptone (Duchefa Biochemie)
	0.50% (w/v) NaCl (Roth or VWR)

Information and characterization of BcBot2

The gene for BcBot2 used in this work was synthesized by GENEWIZ LLC as a construct with pUC57 and are optimized for protein expression in *E.coli*. The gene sequence can be used from a gene bank for the accession number Q6WP50.[15]

Information and characterization of PvHVS

The gene for PvHVS used in this work was synthesized by GenScript Biotech(Netherlands)B.V. as a constructs in pET28a(+) and pET28a(+)-TEV. The gene sequence can be used from a gene bank for the accession number AZB50511.[9]

Information and characterization of Omp7

The gene for Omp7 used in this work was synthesized by GENEWIZ LLC as a construct with pUC57 and are optimized for protein expression in *E.coli*. The gene sequence can be used from a gene bank for the accession number MUStwsD_GLEAN_10003831.[14b]

Information and characterization of PenA

The gene for PenA used in this work was synthesized by GENEWIZ LLC as a construct with pUC57 and are optimized for protein expression in *E.coli*. The gene sequence can be used from a gene bank for the accession number AAA19131.[13a]

Information and characterization of BcBot2 mutants

Information about the forward and reverse primers can be found in our previous publication, where the same mutants have already been used.[(20)]

1.2 Synthesis

1.2.1 Chemical Synthesis

Silylether S1 [6a]

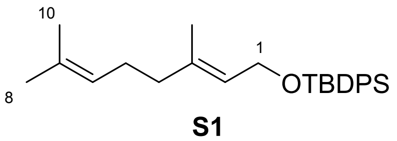

S1
$C_{26}H_{36}OSi$
392.66

Geraniol (**11**) (2.01 g, 13.0 mmol, 1.00 eq.) was dissolved in *N*,*N*-DMF (6 mL) and imidazole (1.96 g, 28.8 mmol, 2.21 eq.) was added. The solution was cooled to 0 °C and TBDPSCl (3.7 mL, 3.92 g, 14.3 mmol, 1.10 eq.) was added. After stirring for 10 min at 0 °C, the reaction was warmed to rt. An additional hour later, water was added and the mixture was diluted with EtOAc. The phases were separated, the aqueous phase was extracted with EtOAc (3x) and the combined organic phases were washed with an aq. sat. $NaHCO_3$ solution and brine. The combined organic phases were dried over $MgSO_4·H_2O$, filtered and the solvent was removed *in vacuo*. The crude product was purified by column chromatography (PE:EtOAc, 9:1) and silylether **S1** (4.95 g, 12.6 mmol, 97%) was obtained as a colourless to pale yellow oil. The analytical data is in accordance to the literature. [6a]

R_f = 0.78 (PE:EtOAc, 10:1); ^{1}H NMR (400 MHz, $CDCl_3$): δ = 7.72 – 7.69 (m, 4H, H_{Ar}), 7.44 – 7.35 (m, 6H, H_{Ar}), 5.38 (tq, J = 9.4 Hz, 1.2 Hz, 1H, H_2), 5.12 – 5.08 (m, 1H, H_6), 4.23 (d, J = 6.2 Hz, H_1), 2.10 – 2.04 (m, 2H, H_5), 2.00 – 1.96 (m, 2H, H_4), 1.69 (s, 3H, H_8/H_{10}), 1.61 (s, 3H, H_8/H_{10}), 1.44 (s, 3H, H_9), 1.05 (s, 9H, $H_{t\text{-Bu}}$) ppm; ^{13}C NMR (101 MHz, $CDCl_3$): δ = 137.2 (C_3), 135.8 (C_{Ar}), 134.3 (C_{Ar}), 131.7 (C_7),129.6 (C_{Ar}), 127.7 (C_{Ar}), 124.3 (C_2/C_6), 124.2 (C_6/C_2), 61.3 (C_1), 39.6 (C_4), 27.0 ($C_{t\text{-Bu}}$), 26.5 (C_5), 25.8 (C_8/C_{10}), 19.3 ($C_{t\text{-Bu}}$), 17.9 (C_8/C_{10}), 16.5 (C_9) ppm; HRMS [GC-MS, EI]: *m/z* calcd for $C_{26}H_{36}OSi$ $[M]^+$: 392.2535, found: 392.2519.

Alcohol 12 [6a]

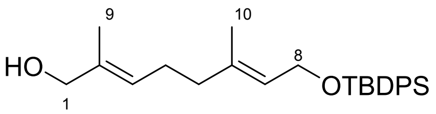

12
$C_{26}H_{36}O_2Si$
408.66

SeO_2 (168 mg, 1.51 mmol, 0.12 eq.) was suspended in CH_2Cl_2 (2 mL) and cooled to 0 °C. Salicylic acid (176 mg, 1.27 mmol, 0.10 eq.), water (2 drops) and *t*-BuOOH (5 – 6 M in decane, 9 mL, 45.0 mmol, 3.57 eq.) were added. Then silylether **S1** (4.95 g, 12.6 mmol, 1.00 eq.) dissolved in CH_2Cl_2 (2 mL + 6 mL) was added, the mixture was warmed to rt and stirred o/n. Acetone, EtOAc, an aq. sat. $NaHCO_3$ solution and brine were added and the phases were separated. The aqueous phase was extracted with EtOAc (3x), the combined organic phases were dried over $MgSO_4·H_2O$, filtered and the solvent was removed *in vacuo*. The residue was taken up in Methanol (20 mL) and cooled to 0 °C. $NaBH_4$ (587 mg, 15.5 mmol, 1.23 eq.) was carefully added and the reaction stirred at 0 °C for 2 h before adding acetone and EtOAc. The organic phase was washed with an aq. sat. $NaHCO_3$ solution and brine. Then it was dried over $MgSO_4·H_2O$, filtered and the solvent was removed *in vacuo*. The crude product was purified by coloumn chromatography (PE:EtOAc, 4:1) and alcohol **12** (1.60 g, 3.92 mmol, 31%) was obtained as a yellow oil. The analytical data is in accordance to the literature. [6a]

R_f = 0.48 (PE:EtOAc, 2:1); ^{1}H NMR (400 MHz, $CDCl_3$): δ = 7.71 – 7.67 (m, 4H, H_{Ar}), 7.44 – 7.35 (m, 6H, H_{Ar}), 5.41 – 5.36 (m, 2H, H_3, H_7), 4.23 – 4.21 (m, 2H, H_8), 3.99 (d, J = 5.8 Hz, 2H, H_1), 2.15 – 2.10 (m, 2H, H_4), 2.03 – 2.00 (m, 2H, H_5), 1.67 (s, 3H, H_9), 1.45 (s, 3H, H_{10}), 1.22 (t, J = 6.0 Hz, 1H, H_{OH}), 1.04 (s, 3H, $H_{t\text{-Bu}}$) ppm; ^{13}C NMR (101 MHz, $CDCl_3$): δ = 136.8 (C_6), 135.8 (C_{Ar}), 135.1 (C_2), 134.2 (C_{Ar}), 129.7 (C_{Ar}), 127.7 (C_{Ar}), 126.0 (C_3) 124.5 (C_7), 69.2 (C_1), 61.3 (C_8), 39.2 (C_5), 27.0 ($C_{t\text{-Bu}}$), 26.0 (C_4), 19.3 ($C_{t\text{-Bu}}$), 16.5 (C_{10}), 13.9 (C_9) ppm; HRMS [ESI-MS]: *m/z* calcd for $C_{26}H_{36}O_2NaSi$ $[M+Na]^+$: 431.2382, found: 431.2382.

Ether S2

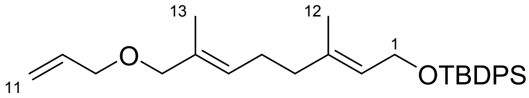

S2
$C_{29}H_{40}O_2Si$
448.72

NaH (60% in mineral oil, 139 mg, 3.48 mmol, 2.11 eq.) was suspensed in THF (10 mL) and cooled to 0 °C. Alcohol **12** (676 mg, 1.65 mmol, 1.00 eq.) was dissolved in THF (5 mL + 5 mL) and added to the reaction. After stirring 1 h at 0 °C allyl bromide (0.29 mL, 405 mg, 3.35 mmol, 2.03 eq.) was added and after further 10 min. The reaction was warmed to rt and stirred o/n, before adding more NaH (60% in mineral oil, 79 mg, 1.98 mmol, 1.20 eq.) and stirring for additional 3 days. An aq. sat. $NaHCO_3$ solution, water and Et_2O were added and the phases were separated. The aqueous phase was extracted with Et_2O (3x) and the combined organic phases were washed with an aq. sat. $NaHCO_3$ solution, dried over $MgSO_4{\cdot}H_2O$, filtered and the solvent was removed *in vacuo*. The crude product was purified by column chromatography (PE:EtOAc, 10:1) and ether **S2** (607 mg, 1.35 mmol, 82%) was obtained as a yellowish oil.

R_f = 0.56 (PE:EtOAc, 10:1); ^{1}H NMR (400 MHz, $CDCl_3$): δ = 7.70 – 7.68 (m, 4H, H_{Ar}), 7.44 – 7.35 (m, 6H, H_{Ar}), 5.96 – 5.86 (m, 1H, H_{10}), 5.40 – 5.37 (m, 2H, H_2, H_6), 5.28 – 5.14 (m, 2H, H_{11}), 4.22 (d, J = 6.4 Hz, 2H, H_1), 3.90 (dt, J = 5.6 Hz, 1.4 Hz, 2H, H_9), 3.85 (s, 3H, H_8), 2.16 – 2.10 (m, 2H, H_5), 2.03 – 2.00 (m, 2H, H_4), 1.65 (s, 3H, H_{13}), 1.44 (s, 3H, H_{12}), 1.04 (s, 9H, $H_{t\text{-Bu}}$) ppm; ^{13}C NMR (101 MHz, $CDCl_3$): δ = 136.8 (C_3), 135.8 (C_{Ar}), 135.2 (C_{10}), 134.2 (C_7), 132.4 (C_{Ar}), 129.6 (C_{Ar}), 128.0 (C_6), 127.7 (C_{Ar}), 124.4 (C_2), 116.9 (C_{11}), 76.4 (C_8), 70.6 (C_9), 61.3 (C_1), 39.2 (C_4), 27.0 ($C_{t\text{-Bu}}$), 26.1 (C_5), 19.3 ($C_{t\text{-Bu}}$), 16.4 (C_{12}), 14.1 (C_{13}) ppm; HRMS [ESI-MS]: *m/z* calcd for $C_{29}H_{40}O_2NaSi$ $[M+Na]^+$: 471.2695, found: 471.2682.

Alcohol 13

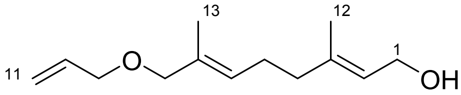

13
$C_{13}H_{22}O_2$
210.32

Ether **S2** was dissolved in THF (20 mL) and cooled to 0 °C. TBAF (1 M in THF, 2.00 mL, 2.00 mmol, 1.49 eq.) was added and the reaction stirred at 0 °C for 30 min before stirring at rt o/n. Then water, EtOAc and brine were added and the phases were separated. The aqueous phase was extracted with EtOAc (3x) and the combined organic phases were dried over $MgSO_4{\cdot}H_2O$, filtered and the solvent was removed *in vacuo*. The crude product was purified by column chromatography (PE:EtOAc, ≈3:1) and Alcohol **13** (263 mg, 1.25 mmol, 93%) was obtained as a pale yellow oil.

R_f = 0.33 (PE:EtOAc, 2:1); ^{1}H NMR (400 MHz, $CDCl_3$): δ = 5.97 – 5.87 (m, 1H, H_{10}), 5.43 – 5.37 (m, 2H, H_2, H_6), 5.29 – 5.15 (m, 2H, H_{11}), 4.15 (d, J = 6.8 Hz, 2H, H_1), 3.92 (dt, J = 5.6 Hz, 1.2 Hz, 2H, H_9), 3.85 (s, 2H, H_8), 2.21 – 2.15 (m, 2H, H_5), 2.09 – 2.06 (m, 2H, H_4), 1.68 (s, 3H, H_{12}), 1.65 (s, 3H, H_{13}) ppm; ^{13}C NMR (101 MHz, $CDCl_3$): δ = 139.4 (C_3), 135.1 (C_{10}), 132.7 (C_7), 127.7 (C_6), 123.9 (C_2), 117.0 (C_{11}), 76.3 (C_8), 70.7 (C_9), 59.5 (C_1), 39.2 (C_4), 26.1 (C_5), 16.4 (C_{12}), 14.1 (C_{13}) ppm; HRMS [ESI-MS]: *m/z* calcd for $C_{13}H_{22}O_2Na$ $[M+Na]^+$: 233.1517, found: 233.1510.

FPP derivative 10

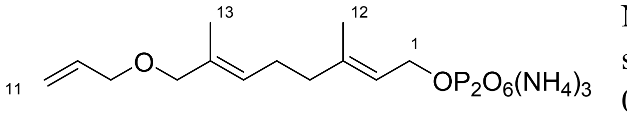

10
$C_{13}H_{33}N_3O_8P_2$
421.37

NCS (179 mg, 1.34 mmol, 1.88 eq.) was suspended in CH_2Cl_2 (10 mL) and cooled to 0 °C. DMS (0.90 mL, 0.76 g, 1.22 mmol, 1.71 eq.) was added and the reaction stirred at 0 °C for 10 min before adding alcohol **13** (150 mg, 0.71 mmol, 1.00 eq.) dissolved in CH_2Cl_2 (5 mL + 5 mL). After stirring for about 45 min at 0 °C, brine and *n*-pentane were added and the phases were separated. The aqueous phase was extracted with *n*-pentane (3 x), the combined organic phases were dried over MgSO4·H_2O, filtered and the solvent was carefully removed *in vacuo*. The crude product was purified by coloumn chromatography (*n*-pentane:Et_2O, 100:1 → 30:1) and the product was used for the next step without further analysis. Tris(tetra-*n*-butylammonium) hydrogen pyrophosphate (1.36 g, 1.51 mmol, 2.12 eq.) was dissolved in MeCN (10 mL) and the chloride was added as a solution in MeCN (5 mL + 5 mL). The reaction stirred at rt o/n before the solvent was removed *in vacuo*. The residue was loaded onto the ion exchange coloumn as described in the General Information. FPP derivative **10** (246 mg, 0.58 mmol, 82%) was obtained as a fluffy solid.

^{1}H NMR (400 MHz, $CDCl_3$): δ = 5.95 (ddt, J = 17.1 Hz, 10.6 Hz, 6.1 Hz, 1H, H_{10}), 5.52 – 5.44 (m, 2H, H_2, H_6), 5.34 – 5.24 (m, 2H, H_{11}), 4.47 (t, J = 6.7 Hz, 2H, H_1), 3.97 (dt, J = 6.0 Hz, 1.3 Hz, 2H, H_9), 3.94 (s, 2H, H_8), 2.24 – 2.20 (m, 2H, H_5), 2.16 – 2.12 (m, 2H, H_4), 1.72 (s, 3H, H_{12}), 1.65 (s, 3H, H_{13}) ppm; ^{13}C NMR (101 MHz, $CDCl_3$): δ = 142.5 (C_3), 133.9 (C_{10}), 131.7 (C_7), 129.7 (C_6), 119.9 (d, C_2), 118.3 (C_{11}), 76.0 (C_8), 70.0 (C_9), 62.6 (d, C_1), 38.3 (C_4), 25.3 (C_5), 15.5 (C_{12}), 13.2 (C_{13}) ppm; ^{31}P NMR (162 MHz, D_2O): δ = −7.3 (d, J = 20.8 Hz), −10.1 (d, J = 20.0 Hz) ppm; HRMS [ESI-MS]: *m/z* calcd for $C_{13}H_{23}O_8P_2$ $[M-(NH_4)_3+H_2]^-$: 369.0868, found: 369.0872.

Alcohol S3 [S1]

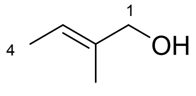

S3
$C_5H_{10}O$
86.13

LAH (10.8 g, 115 mmol, 4.08 eq.) was suspended in Et_2O (80 mL) and cooled to 0 °C. Tiglic acid (2.83 g, 28.2 mmol, 1.00 eq.) was dissolved in Et_2O (10 mL + 10 mL) and slowly added to the reaction. After stirring at 0 °C for 5 min, the reaction was warmed to rt and stirred for 2.5 h. An aq. sat. K,Na-tartrate solution and Et_2O were carefully added at 0 °C and the mixture stirred at rt o/n to enhance the phase separation. The phases were separated and the aqueous phase was

extracted with Et_2O (3x). The combined organic phases were washed with an aq. K,Na-tartrate solution, dried over $MgSO_4 \cdot H_2O$, filtered and the solvent was removed *in vacuo*. The crude product was purified by column chromatography (*n*-pentane:Et_2O, 2:1) and the solvents were carefully removed (pressure up to 300 mbar). Alcohol **S3** (1.47 g, 17.0 mmol, 60%) was obtained as a colourless oil. The ^{1}H-NMR data is in accordance with the literature.[S1,S2]

^{1}H NMR (400 MHz, $CDCl_3$): δ = 5.52 – 5.46 (m, 1H, H_3), 4.00 (d, J = 4.9 Hz, 2H, H_1), 1.67 (s, 3H, H_5), 1.62 (d, J = 6.7 Hz, 3H, H_4), 1.33 (t, J = 5.5 Hz, 1H, H_{OH}) ppm; ^{13}C NMR (101 MHz, $CDCl_3$): δ = 135.6 (C_2), 120.8 (C_3), 69.2 (C_1), 13.5 (C_4/C_5), 13.2 (C_4/C_5) ppm.

Bromide S4 [S2]

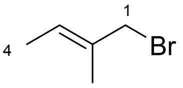

S4
C_5H_9Br
149.03

Alcohol **S3** (371 mg, 4.31 mmol, 1.00 eq.) dissolved in Et_2O (10 mL) was cooled to 0 °C. PBr_3 (0.30 mL, 856 mg, 3.16 mmol, 0.73 eq.) was added and the reaction stirred at 0 °C for 1 h before adding an aq. sat. $NaHCO_3$ solution. The phases were separated, the aqueous phase was extracted with Et_2O (1x) and the combined organic phases were dried over $MgSO_4 \cdot H_2O$, filtered and the solvent was removed *in vacuo* (pressure up to 300 mbar). Bromide **S4** (302 mg, 2.03 mmol, 47%) was obtained as a colourless/yellowish oil with Et_2O residues. The ^{1}H-NMR data is in accordance with the literature.[S2]

^{1}H NMR (400 MHz, $CDCl_3$): δ = 5.69 (q, J = 6.7 Hz, 1H, H_3), 3.98 (s, 2H, H_1), 1.76 – 1.75 (m, 3H, H_5), 1.63 (d, J = 6.8 Hz, 3H, H_4) ppm; ^{13}C NMR (101 MHz, $CDCl_3$): δ = 132.9 (C_2), 126.0 (C_3), 42.0 (C_1), 14.5 (C_4/C_5), 14.0 (C_2/C_3) ppm.

Alcohol 14

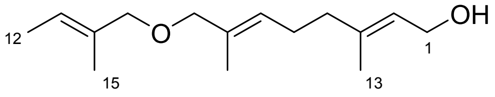

14
$C_{15}H_{26}O_2$
238.37

NaH (60% in mineral oil, 113 mg, 2.83 mmol, 2.17 eq.) was suspended in THF (10 mL) and cooled to 0 °C. Alcohol **12** (530 mg, 1.30 mmol, 1.00 eq.) dissolved in THF (5 ml + 5 mL) was added and the deprotonation was stirred for 1 h at 0 °C. Bromide **S4** (296 mg, 1.99 mmol, 1.53 eq.) was added with THF (1 mL) and after additional 10 min at 0 °C, the reaction mixture was warmed to rt. After stirring o/n additional NaH (60% in mineral oil, 66 mg, 0.95 mmol,1.67 mmol, 1.28 eq.) was added and after 3 d at rt, an aq. sat. NH_4Cl solution, water and Et_2O were added. The phases were separated and the aqueous phase was extracted with Et_2O (3x). The combined organic phases were washed with an aq. sat. $NaHCO_3$ solution, dried over $MgSO_4 \cdot H_2O$, filtered and the solvent was removed *in vacuo*. The crude product was purified by coloumn chromatography (PE:EtOAc, 10:1) to yield the intermediate C_{15} ether with impurities. Therefore the product (256 mg, 0.54 mmol, 1.00 eq.) was dissolved in THF (16 mL) and colled to 0 °C. TBAF (1 M in THF, 0.81 mL, 0.81 mmol, 1.51 eq.) was added and the reaction stirred for 30 min at 0 °C, before stirring at rt o/n. Water was added and the phases were separated. The aqueous phase was extracted with EtOAc (3x) and the combined organic phases were dried over $MgSO_4 \cdot H_2O$, filtered and the solvent was removed *in vacuo*. The crude product was purified by column

chromatography (PE:EtOAc, 3:1) and alcohol **14** (100 mg, 0.42 mmol, 78%, 32% o.2.s.) was obtained as a yellow oil.

R_f = 0.48 (*n*-pentane:Et_2O, 1:1); ^1H NMR (400 MHz, $CDCl_3$): δ = 5.50 – 5.44 (m, 1H, H_{11}), 5.43 – 5.39 (m, 1H, H_2), 5.39 – 5.34 (m, 1H, H_6), 4.14 (d, J = 6.8 Hz, 2H, H_1), 3.77 (m, 4H, H_8, H_9), 2.20 – 2.14 (m, 2H, H_5), 2.09 – 2.05 (m, 2H, H_4), 1.68 (s, 3H, H_{13}), 1.64 (s, 6H, H_{14}, H_{15}), 1.63 – 1.61 (m, 3H, H_{12}) ppm; ^{13}C NMR (101 MHz, $CDCl_3$): δ = 139.5 (C_3), 133.2 (C_7/C_{10}), 132.9 (C_7/C_{10}), 127.5 (C_6), 123.8 (C_2), 122.5 (C_{11}), 75.9 (C_8/C_9), 75.8 (C_8/C_9), 59.5 (C_1), 39.2 (C_4), 26.1 (C_5), 16.4 (C_{13}), 14.1 (C_{14}/C_{15}), 13.8 (C_{14}/C_{15}), 13.3 (C_{12}) ppm; HRMS [ESI-MS]: *m/z* calcd for $C_{15}H_{26}O_2Na$ $[M+Na]^+$: 261.1831, found: 261.1833.

FPP derivative 9

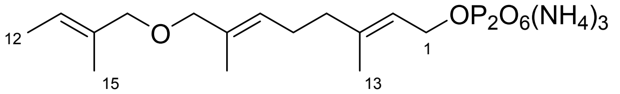

9
$C_{15}H_{37}N_3O_8P_2$
449.42

NCS (30 mg, 0.22 mmol, 1.88 eq.) was dissolved in CH_2Cl_2 (3 mL) and cooled to −50 °C to −40 °C. DMS (20 µL, 17 mg, 0.27 mmol, 2.25 eq.) was added and the reaction witrred at 0 °C for 5 min before being cooled to −50 °C to −40 °C again. Alcohol **14** (29 mg, 0.12 mmol, 1.00 eq.) was dissolved in CH_2Cl_2 (1 mL + 1 mL) and added to the reaction. Shortly after the reaction was warmed to 0 °C where it stirred for 1 h before adding additional NCS (44 mg, 0.33 mmol, 2.75 eq.) and DMS (0.05 mL, 42 mg, 0.68 mmol, 5.63 eq.). After 30 min the reaction was warmed to rt and after 15 min brine was added. The mixture was diluted with water and *n*-penante, the phases were separated and the aqueous phase was extracted with *n*-pentane (3x). The combined organic phases were dried over $MgSO_4{\cdot}H_2O$, filtered and the solvent was removed *in vacuo* (up to 500 mbar). Tris(tetra-*n*-butylammonium) hydrogen pyrophosphate (235 mg, 0.26 mmol, 2.17 eq.) was dissolved in MeCN (3 mL) and the chloride was added as a solution in MeCN (2 mL + 1 mL). The reaction stirred at rt o/n before the solvent was removed *in vacuo*. The residue was loaded onto the ion exchange coloumn as described in the General Information. FPP derivative **9** (67 mg, 0.15 mmol, quant.) was obtained as a white yellow solid.

^1H NMR (400 MHz, D_2O): δ = 5.60 – 5.54 (m, 1H, H_{11}), 5.51 – 5.46 (m, 2H, H_6, H_2), 4.48 (dd, J = 6.6 Hz, 6.6 Hz, 2H, H_1), 3.87 (s, 2H, H_8), 3.85 (s, 2H, H_9), 2.26 – 2.21 (m, 2H, H_5), 2.16 – 2.12 (m, 2H, H_4), 1.73 (s, 3H, H_{13}), 1.66 – 1.62 (m, 9H, H_{12}, H_{14}, H_{15}) ppm; ^{13}C NMR (151 MHz, D_2O): δ = 142.4 (C_3), 132.4 (C_{10}), 131.9 (C_7), 129.6 (C_6), 124.6 (C_{11}), 120.1 (d, C_2), 75.3 (C_8), 75.2 (C_9), 62.4 (d, C_1), 38.4 (C_4), 25.4 (C_5), 15.6 (C_{13}), 13.2 (C_{14}/C_{15}), 13.0 (C_{14}/C_{15}), 12.5 (C_{12}) ppm; ^{31}P NMR (162 MHz, D_2O): δ = −6.4 (d, J = 22.2 Hz), −10.1 (d, J = 22.2 Hz) ppm; HRMS [ESI-MS]: *m/z* calcd for $C_{15}H_{27}O_8P_2$ $[M-(NH_4)_3+H_2]^-$: 397.1181, found: 397.1175.

Ether S5 [6a]

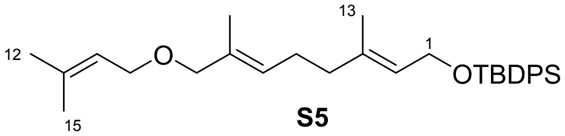

S5
$C_{29}H_{40}O_2Si$
448.72

NaH (90%, 131 mg, 4.89 mmol, 2.00 eq.) was suspended in THF (2 mL) at 0 °C and alcohol **12** (1.00 g, 2.45 mmol, 1.00 eq.) in THF (2 mL) was added. The mixture was stirred for 1 h and was allowed to warm to rt, where it stirred for another 20 min. Then TBAI (27 mg, 0.73 mmol, 0.30 eq.) and 3,3-dimethylallyl bromide (0.37 mL, 3.18 mmol, 1.30 eq.) were added at rt. The resulting mixture was stirred at rt overnight. A sat. aq. NH_4Cl solution was added, the phases were separated and the aqueous layer was extracted with Et_2O (3x). The combined organic phases were washed with brine, dried over $MgSO_4{\cdot}H_2O$, filtered and the solvent was removed *in vacuo*. The crude product was purified by column chromatography (PE 100% → PE:EtOAc, 25:1) and ether **S5** (1.04 g, 2.19 mmol, 89%) was obtained as a yellowish oil. The analytical data is in accordance with the literature. [6a]

R_f = 0.29 (PE 100%); ^{1}H NMR (400 MHz, $CDCl_3$): δ = 7.73 – 76.5 (m, 4H, H_{Ar}), 7.46 – 7.33 (m, 6H, H_{Ar}), 5.42 – 5.31 (m, 3H, H_2, H_6, H_{10}), 4.21 (d, J = 6.3 Hz, 2H, H_1), 3.87 (d, J = 7.0 Hz, 2H, H_9), 3.82 (s, 2H, H_8), 2.13 (m, 2H, H_5), 2.01 (m, 2H, H_4), 1.74 (s, 3H, H_{12}/H_{15}), 1.65 (s, 6H, H_{12}/H_{15}, H_{14}), 1.44 (s, 3H, H_{13}), 1.04 (s, 9H, $H_{t\text{-Bu}}$) ppm; ^{13}C NMR (101 MHz, $CDCl_3$): δ = 136.9 (C_3), 136.8 (C_{11}), 135.8 (C_{Ar}), 134.2 (C_{Ar}), 132.7 (C_7), 129.6 (C_{Ar}), 127.9 (C_6), 127.7 (C_{Ar}), 124.4 (C_2), 121.5 (C_{10}), 76.3 (C_8), 66.0 (C_9), 61.3 (C_1), 39.2 (C_4), 27.0 ($C_{t\text{-Bu}}$), 26.2 (C_5), 26.0 (C_{12}/C_{15}), 19.3 ($C_{t\text{-Bu}}$), 18.2 (C_{12}/C_{15}), 16.4 (C_{13}), 14.1 (C_9) ppm.

Alcohol S6 [6a]

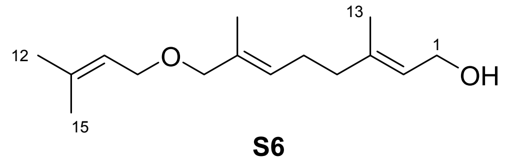

S6
$C_{15}H_{26}O_2$
238.37

Ether **S5** (1.04 g, 2.18 mmol, 1.00 eq.) was dissolved in THF (23 mL) and cooled to 0 °C. TBAF (1 M in THF, 6.52 mL, 6.52 mmol, 3.00 eq.) was added and the reaction stirred at rt o/n. An aq. sat. NH_4Cl solution was added and the phases were separated. The aqueous layer was extracted with EtOAc (3x), the combined organic phases were washed with brine and dried over $MgSO_4{\cdot}H_2O$, filtered and the solvent was removed *in vacuo*. The crude product was purified by column chromatography (PE:EtOAc, 4:1) and alcohol **S6** (430 mg, 1.80 mmol, 78%) was obtained as a yellowish oil. The analytical data is in accordance with the literature. [6a]

R_f = 0.20 (PE:EtOAc, 5:1); ^{1}H NMR (400 MHz, $CDCl_3$): δ = 5.47 – 5.29 (m, 3H, H_2, H_6, H_{10}), 4.14 (d, J = 6.9 Hz, 2H, H_1), 3.88 (d, J = 6.9 Hz, 2H, H_9), 3.82 (s, 2H, H_8), 2.21 – 2.12 (m, 2H, H_5), 2.10 – 2.03 (m, 2H, H_4), 1.74 (s, 3H, H_{12}/H_{15}), 1.68 (s, 3H, H_{13}), 1.66 (s, 3H, H_{12}/H_{15}), 1.65 (s, 3H, H_{14}) ppm; ^{13}C NMR (101 MHz, $CDCl_3$): δ = 139.5 (C_3), 136.9 (C_{11}), 132.9 (C_7), 127.6 (C_6), 123.8 (C_2), 121.4 (C_{10}), 76.3 (C_8), 66.1 (C_9), 59.5 (C_1), 39.2 (C_4), 26.1 (C_5), 25.9 (C_{12}/C_{15}), 18.1 (C_{12}/C_{15}), 16.4 (C_{13}), 14.1 (C_{14}) ppm.

FPP Derivative 3 [6a]

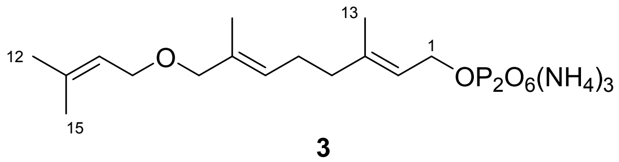

3
$C_{15}H_{37}N_3O_8P_2$
449.42

Alcohol **S6** (150 mg, 0.63 mmol, 1.00 eq.) was dissolved in THF (6 mL) and cooled to 0 °C. Et_3N (0.20 mL, 1.47 mmol, 2.33 eq.) and MsCl (0.10 mL, 1.26 mmol, 2.00 eq.) were added dropwise and the reaction stirred at 0 °C for 1 h. Then LiCl (123 mg, 2.89 mmol, 4.60 eq.) was added and the reaction stirred for 45 min at 0 °C and 30 min at rt. Water was added and the phases were separated. The aqueous layer was extracted with PE (3x) and the combined organic phases were washed with brine, dried over $MgSO_4 \cdot H_2O$, filtered and the solvent was removed *in vacuo*. The allylic chloride was obtained as a colourless oil and used in the next reaction without further purification. The chloride was dissolved in MeCN (4 mL) and added to a solution of Tris(tetra-*n*-butylammonium) hydrogen pyrophosphate (1.12 g, 1.25 mmol, 2.00 eq.) and 3Å-sieves in MeCN (4 mL). The mixture stirred at rt o/n. The solvent was removed *in vacuo* and the residue was loaded onto the ion exchange coloumn as described in the General Information and FPP derivative **3** (250 mg, 0.56 mmol, 89%) was obtained as a white solid. The analytical data is in accordance with the literature. [6a]

^{1}H NMR (400 MHz, D_2O): δ = 5.55 – 5.42 (m, 2H, H_2, H_6), 5.41 – 5.33 (m, 1H, H_{10}), 4.49 (dd, J = 6.5 Hz, 6.5 Hz, 2H, H_1), 3.96 (d, J = 7.4 Hz, 2H, H_9), 3.92 (s, 2H, H_8), 2.28 – 2.19 (m, 2H, H_5), 2.19 – 2.12 (m, 2H, H_4), 1.77 (s, 3H, H_{12}/H_{15}), 1.74 (s, 3H, H_{13}), 1.69 (s, 3H, H_{12}/H_{15}), 1.66 (s, 3H, H_{14}) ppm; ^{13}C NMR (101 MHz, D_2O): δ = 142.8 (C_3), 140.4 (C_{11}), 131.8 (C_7), 129.7 (C_6), 119.7 (d, J = 7.8 Hz, C_2), 119.1 (C_{10}), 75.7 (C_8), 65.1 (C_9), 62.9 (d, J = 4.7 Hz, C_1), 38.3 (C_4), 25.4 (C_6), 24.9 (C_{12}/C_{15}), 17.2 (C_{12}/C_{15}), 15.5 (C_{13}), 13.2 (C_{14}) ppm; ^{31}P NMR (162 MHz, D_2O): δ = −9.9, −10.3 ppm;

1.2.2 Biotransformations

Biotransformation products 18 - 21

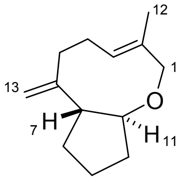

18
$C_{13}H_{20}O$
192.3020

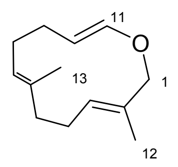

19
$C_{13}H_{20}O$
192.3020

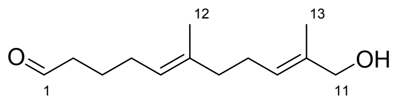

20
$C_{13}H_{20}O$
192.3020

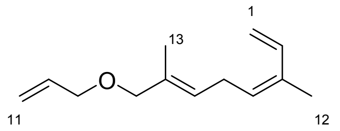

21
$C_{13}H_{20}O$
192.3020

FPP derivative **10** (146 mg, 0.35 mmol, 1.00 eq.) was dissolved in an aq. NH_4HCO_3 (0.05 M, 17.3 mL) solution as a stock solution. The following reaction was performed in four 50 mL batches, the procedure will be described for one 50 mL batch. Tween 20® (10 µL) and PPase (1 µL) were dissolved in HEPES buffer (44.5 mL). The derivative **10** stock solution (500 µL) and BcBot2 solution (4.01 mg/mL, 624 µL) were added and the reaction was started by adding $MgCl_2$ (2 M, 250 µL), while shaking at 100 rpm and 37 °C. Every 30 min more derivative **10** stock solution (500 µL) was added to a total volume of 4 mL. After 120 min a second batch of BcBot2 solution (624 µL) was added. The reaction then continued o/n at 37 °C and 100 rpm, before separating the phases after the addition of *n*-pentane. The aqueous phase was extracted with *n*-pentane (3x) and the combined organic phases were washed with brine, dried over $MgSO_4 \cdot H_2O$ and the solvent was removed *in vacuo*. The crude product was purified by column

chromatography (*n*-pentane:Et_2O, 30:1 → 1:1) (isolation of **20**) and non-polar fractions were collected and purified by multiple coloumn chromatographies (*n*-pentane : Et_2O, 100:1) until the different compounds were separated and isolated (**18**, **19** and **21**). The NMR samples were prepared by co-evaporation with C_6D_6 resulting in yields not being determined, as the previously unknown products were considered potentially volatile.

Analytical data of **18**

R_f = 0.80 (PE:EtOAc, 8:1); ^{1}H NMR (600 MHz, C_6D_6): δ = 5.50 (dd, J = 9.1 Hz, 1H, H_3), 4.77 (d, J = 5.0 Hz, 2H, H_{13}), 4.12 (d, J = 10.5 Hz, 1H, H_1), 3.58 – 3.56 (m, 2H, H_1, H_{11}), 2.34 – 2.27 (m, 1H, H_4), 2.14 – 2.03 (m, 3H, H_5, H_7), 1.97 – 1.86 (m, 3H, H_4, H_8, H_{10}), 1.80 – 1.74 (m, 1H, H_{10}), 1.67 (s, 3H, H_{12}), 1.66 – 1.61 (m, 1H, H_9), 1.55 – 1.51 (m, 1H, H_9), 1.35 – 1.27 (m, 1H, H_8) ppm; ^{13}C NMR (151 MHz, C_6D_6): δ = 155.7 (C_6), 134.5 (C_2), 130.7 (C_3), 112.2 (C_{13}), 87.6 (C_{11}), 78.8 (C_1), 58.0 (C_7), 36.3 (C_8), 35.7 (C_{10}), 34.8 (C_5), 26.6 (C_4), 24.8 (C_9), 16.7 (C_{12}) ppm; HRMS [GC-MS, CI]: *m/z* calcd for $C_{13}H_{20}O$ $[M]^+$: 192.1514, found: 192.1512.

Analytical data of **19**

R_f = 0.87 (PE:EtOAc, 8:1); ^{1}H NMR (600 MHz, C_6D_6): δ = 5.51 (d, J = 12.1 Hz, 1H, H_{11}), 4.93 – 4.90 (m, 1H, H_3), 4.75 (dt, J = 12.1 Hz, 7.6 Hz, 1H, H_{10}), 4.69 (t, J = 6.9 Hz, 1H, H_7), 3.87 (s, 2H, H_1), 2.04 – 2.00 (m, 4H, H_4, H_5), 1.92 – 1.85 (m, 4H, H_8, H_9), 1.49 (s, 3H, H_{12}), 1.36 (s, 3H, H_{13}) ppm; ^{13}C NMR (151 MHz, C_6D_6): δ = 147.1 (C_{11}), 133.8 (C_6), 131.8 (C_2), 131.2 (C_3), 127.2 (C_7), 109.7 (C_{10}), 77.7 (C_1), 39.8 (C_5), 27.9 (C_8), 27.4 (C_9), 25.1 (C_4), 15.2 (C_{13}), 14.5 (C_{12}) ppm; HRMS [GC-MS, EI]: *m/z* calcd for $C_{13}H_{20}O$ $[M]^+$: 192.1514, found: 192.1510.

Analytical data of **20**

R_f = 0.39 (PE:EtOAc, 2:1); ^{1}H NMR (600 MHz, C_6D_6): δ = 9.32 (t, J = 1.7 Hz, 1H, H_1), 5.35 (tq, J = 10.5 Hz, 1.3 Hz, 1H, H_9), 5.05 – 5.02 (m, 1H, H_5), 3.81 (s, 2H, H_{11}), 2.13 – 2.09 (m, 2H, H_8), 2.03 – 2.00 (m, 2H, H_5), 1.86 – 1.81 (m, 4H, H_2, H_4), 1.56 (s, 3H, H_{13}), 1.48 (s, 3H, H_{12}), 1.40 (tt, J = 7.2 Hz, 7.2 Hz, 2H, H_3) ppm; ^{13}C NMR (151 MHz, C_6D_6): δ = 201.1 (C_1), 135.9 (C_6), 135.6 (C_{10}), 124.9 (C_9), 124.2 (C_5), 68.6 (C_{11}), 43.2 (C_2), 39.8 (C_7), 27.5 (C_4), 26.3 (C_8), 22.4 (C_3), 16.0 (C_{12}), 13.7 (C_{13}) ppm; HRMS [GC-MS, CI]: *m/z* calcd for $C_{13}H_{22}O_2$ $[M]^+$: 210.1620, found: 210.1618.

Analytical data of **21**

R_f = 0.87 (PE:EtOAc, 8:1); ^{1}H NMR (600 MHz, C_6D_6): δ = 6.82 (dd, J = 17.3 Hz, 10.8 Hz, 1H, H_2), 5.91 – 5.82 (m, 1H, H_{10}), 5.42 (t, J = 6.9 Hz, 2H, H_6), 5.33 (t, J = 7.6 Hz, 1H, H_4), 5.27 – 5.24 (m, 1H, H_{11}), 5.20 – 5.17 (d, J = 17.2 Hz, 1H, H_1), 5.06 (d, J = 10.9 Hz, 1H, H_1), 5.04 (m, 1H, H_{11}), 3.79 – 3.75 (m, 4H, H_8, H_9), 2.85 (dd, J = 7.3 Hz, 7.3 Hz, 2H, H_5), 1.76 (s, 3H, H_{12}), 1.62 (s, 3H, H_{13}) ppm; ^{13}C NMR (151 MHz, C_6D_6): δ = 135.8 (C_{10}), 133f.9 (C_2), 133.2 (C_7), 132.8 (C_3), 129.2 (C_4), 125.7 (C_6), 115.9 (C_{11}), 113.9 (C_1), 76.1 (C_8), 70.6 (C_9), 26.4 (C_5), 19.9 (C_{12}), 14.0 (C_{13}) ppm; HRMS [GC-MS, CI]: *m/z* calcd for $C_{13}H_{20}O$ $[M]^+$: 192.1514, found: 192.1517.

Biotransformation products 13 + 20 + 23

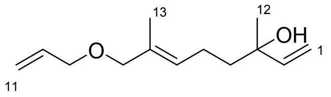

23
$C_{13}H_{22}O_2$
210.32

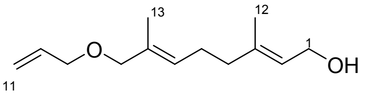

13
$C_{13}H_{22}O_2$
210.32

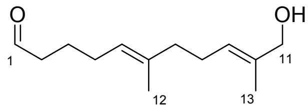

20
$C_{13}H_{22}O_2$
210.32

FPP derivative **10** (86 mg, 0.20 mmol, 1.00 eq.) was dissolved in water (10.9 mL) to prepare a stock solution. The following reaction was performed in five 25 mL batches, the procedure will be described for 25 mL batch. Tween 20® (5 µL) and PPase (0.5 µL) were dissolved in HEPES buffer (22.1 mL). The derivative **10** stock solution (250 µL) and Omp7 solution (3.3 mg/mL, 380 µL) were added and the reaction was started by adding $MgCl_2$ (2 M, 125 µL) at 37 °C and 100 rpm. Every 30 min more derivative **10** stock solution (250 µL) was added to a total volume of 4 mL. After 120 min a seconf batch of Omp7 solution (3.3 mg/mL, 376 µL) was added. The reaction continued o/n at 37 °C and 100 rpm, before seperating the phases after adding *n*-pentane. The aqueous phase was extracted with *n*-pentane (3x) and the combined organic phases were washed with brine and were exposed to an ultrasonic bath to enhance the phase separation. After drying over $MgSO_4 \cdot H_2O$ and filtration, the solvent was very carefully removed *in vacuo*. The crude product was purified by coloumn chromatography (*n*-pentane:Et_2O, 5:1 → 4:1 → 3:1) and the isolated fractions were coevaporated with C_6D_6 for NMR measurements. The oily products were weighed before adding C_6D_6, therefore likely containing solvent residues at this point: **23:** 7 mg, **13**: 195 mg, **20**: 8 mg. Spectroscopic data of **23** is used from a different biotransformation using Tri5, resulting in the same product as mentioned above.

Analytical data of **23**

R_f = 0.54 (PE:EtOAc, 5:1); ^{1}H NMR (400 MHz, C_6D_6): δ = 5.88 (ddt, J = 17.2 Hz, 10.5 Hz, 5.2 Hz, 1H, H_{10}), 5.72 (dd, J = 17.3 Hz, 10.7 Hz, 1H, H_2), 5.44 (tq, J = 10.9 Hz, 1.3 Hz, 1H, H_6), 5.29 (ddt, J = 17.2 Hz, 1.8 Hz, 1.8 Hz, 1H, H_{11}), 5.18 (dd, J = 17.3 Hz, 1.6 Hz, 1H, H_1), 5.06 (ddt, J = 10.5 Hz, 1.9 Hz, 1.5 Hz, 1H, H_{11}), 4.94 (dd, J = 10.7 Hz, 1.6 Hz, 1H, H_1), 3.82 (ddd, J = 5.2 Hz, 1.6 Hz, 1.6 Hz, 2H, H_9), 3.79 (s, 2H, H_8), 2.17 – 2.00 (m, 2H, H_5), 1.65 (s, 3H, H_{13}), 1.51 – 1.39 (m, 2H, H_4), 1.09 (s, 3H, H_{12}) ppm; ^{13}C NMR (101 MHz, C_6D_6): δ = 145.5 (C_2), 135.8 (C_{10}), 132.8 (C_7), 128.1 (C_6), 115.9 (C_{11}), 111.5 (C_1), 76.3 (C_8), 72.9 (C_3), 70.6 (C_9), 42.2 (C_4), 28.3 (C_{12}), 22.7 (C_5), 14.0 (C_{13}) ppm; HRMS [GC-MS, EI]: *m/z* calcd for $C_{13}H_{20}O$ $[M-H_2O]^+$: 192.1514, found: 192.1517.

The analytical data for **13** and **20** is already described above.

Biotransformation products 16a and 16b

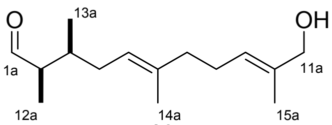

16a
$C_{15}H_{26}O_2$
238.37

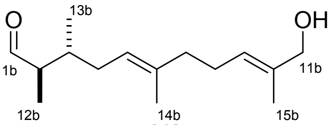

16b
$C_{15}H_{26}O_2$
238.37

FPP derivative **9** (34 mg, 75.7 µmol, 1.00 eq.) was dissolved in an aq. NH_4HCO_3 solution (0.05 M, 4.07 mL). This solution was added to a mixture of HEPES buffer (43.9 mL), Tween20® (10 µL) and PPase (1 µL). Omp7 enzyme solution (2.58 mg/mL, 0.97 mL) and then $MgCl_2$ solution (2 M, 250 µL) was addded to start the reaction. After 2 h at 100 rpm and 37 °C another batch of Omp7 enzyme solution (2.86 mg/mL, 0.87 mL) was added. After 4 h of total reaction time *n*-pentane (25 mL) was added and the mixture was allowed to shake o/n. After the addition of *n*-pentane, the phases were separated (if problems with the separation occur, ultrasonic baths can help), the aqueous phase was extracted with *n*-pentante (3x) and the combined organic phases were dried over $MgSO_4{\cdot}H_2O$, filtered and the solvent was carefully removed. The crude product was purified by coloumn chromatography (*n*-pentane:Et_2O, 1:1) and aldehydes **16a** and **16b** (3 mg) were obtained as a yellowish oil and a mixture of two diastereomers.

Comparing the NMR data of our sample to literature known simplified aldehyds bearing either *threo*- or *erythro* orientated methylgroups in the α- and β-position of an aldehyde, we postulate a ratio from **16a**:**16b** of *threo*:*erythro,* 1.65:1.[19] A more detailed analysis can be seen below in the corresponding section of the SI.

R_f = 0.69 (PE:EtOAc, 1:1); ^{1}H NMR (400 MHz, C_6D_6): δ = 9.40 (d, J = 1.5 Hz, 1H, H_{1b}), 9.39 (d, J = 1.4 Hz, 1H, H_{1a}), 5.38 – 5.30 (m, 2H, H_{9a}, H_{9b}), 5.09 – 5.03 (m, 2H, H_{5a}, H_{5b}), 3.85 (s, 2H, H_{11b}), 3.80 (s, 2H, H_{11a}), 2.14 – 2.00 (m, 8H, H_{2a}, H_{7a}, H_{7b}, H_{8a}, H_{8b}), 1.92 – 1.66 (m, 7H, H_{2b}, H_{3a}, H_{3b}, H_{4a}, H_{4b}), 1.57 (s, 3H, H_{15b}), 1.55 (s, 3H, H_{15a}), 1.49 (s, 3H, H_{14a}), 1.48 (s, 3H, H_{14b}), 0.82 (d, J = 7.0 Hz, 3H, H_{12b}), 0.80 (d, J = 7.0 Hz, 3H, H_{12a}), 0.77 (d, J = 6.8 Hz, 3H, H_{13b}), 0.66 (d, J = 6.7 Hz, 3H, H_{13a}) ppm; ^{13}C NMR (101 MHz, C_6D_6): δ = 204.0 (C_{1a}), 204.0 (C_{1b}), 136.5 (C_{6b}), 136.4 (C_{6a}), 135.6 (C_{10b}), 135.5 (C_a), 124.8 (C_{9a}), 124.8 (C_{9b}), 123.4 (C_{5a}), 123.2 (C_{5b}), 68.6 (C_{11b}), 68.6 (C_{11a}), 51.0 (C_{2b}), 50.1 (C_{2a}), 39.9 (C_{7a}), 39.9 (C_{7b}), 34.8 (C_{3b}), 33.6 (C_{3a}), 33.4 (C_{4a}), 31.8 (C_{4b}), 26.3 (C_{8a}), 26.1 (C_{8b}), 17.9 (C_{13b}), 16.1 (C_{14a}, C_{14b}), 15.6 (C_{13a}), 13.7 (C_{15b}), 13.7 (C_{15a}), 9.7 (C_{12b}), 8.5 (C_{12a}) ppm; HRMS [GC-MS, CI]: *m/z* calcd for $C_{15}H_{27}O_2$ $[M+H]^+$: 239.2011, found: 239.2010.

Biotransformation product 22 and 17

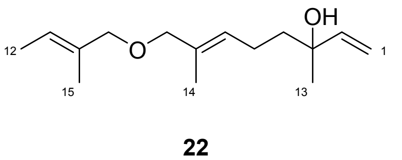

22
$C_{15}H_{26}O_2$
238.37

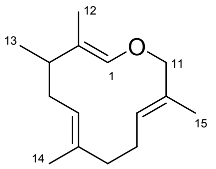

17
$C_{15}H_{24}O$
220.36

FPP derivative **9** (38 mg, 84.6 µmol, 1.00 eq.) was dissolved in an aq. NH_4HCO_3 solution (0.05 M, 4.52 mL). The reaction was performed in two 25 mL batches. The following procedure will be described for one 25 mL batch. Tween20® (10µL) and PPase (0.5 µL) were dissolved in HEPES buffer (22.6 mL). FPP derivative **9** (250 µL) and PenA enzyme solution (11.2 mg/mL, 111 µL) were added and the reaction was started by the addition of $MgCl_2$ (2 M, 125 µL). The reaction was stirred at 37 °C and 100 rpm and every 30 min more FPP derivative **9** (250 µL)

was added to a total volume of 2 mL. After 2 h another batch of PenA enzyme solution (112 µL) was added. The reaction was then allowed to stirr at 37 °C and 100 rpm o/n, before adding *n*-pentane. The phases were separated and the aqueous phase was extracted with *n*-pentane (3x). The combined organic phases were dried over $MgSO_4{\cdot}H_2O$, filtered and the solvent was carefully removed *in vacuo*. The crude product was purified by coloumn chromatography (*n*-pentane:Et_2O, 50:1, 9:1, 3:1, 1:1) and the isolated compounds were prepared by C_6D_6 coevaporation for NMR analysis, still showing residues of *n*-pentane and Et_2O. **22** and **17** were obtained in small amounts of <1 mg. Additionally traces of **16a/b** were isolated, the corresponding data is shown above.

Analytical data of **22**

R_f = 0.63 (PE:EtOAc, 3:1); ^{1}H NMR (600 MHz, C_6D_6): δ = 5.72 (dd, J = 17.3 Hz, 10.7 Hz, 1H, H_2), 5.53 – 5.47 (m, 2H, H_6, H_{11}), 5.18 (dd, J = 17.3 Hz, 1.5 Hz, 1H, H_1), 4.94 (dd, J = 10.7 Hz, 1.5 Hz, 1H, H_1), 3.81 (s, 2H, H_9), 3.80 (s, 2H, H_8), 2.18 – 2.04 (m, 2H, H_5), 1.69 (s, 3H, H_{14}), 1.66 (s, 3H, H_{15}), 1.53 (d, J = 6.8 Hz, 3H, H_{12}), 1.51 – 1.40 (m, 2H, H_4), 1.09 (s, 3H, H_{13}) ppm; ^{13}C NMR (151 MHz, C_6D_6): δ = 145.6 (C_2), 134.0 (C_{10}), 133.2 (C_7), 127.6 (C_6), 121.7 (C_{11}), 111.6 (C_1), 76.0 (C_9), 75.9 (C_8), 73.0 (C_3), 42.4 (C_4), 28.4 (C_{13}), 22.9 (C_5), 14.2 (C_{14}), 13.8 (C_{15}), 13.3 (C_{12}) ppm; HRMS [GC-MS, CI]: *m/z* calcd for $C_{15}H_{25}O$ $[M-H_2O+H]^+$: 221.1905, found: 221.1899.

Analytical data of **17**

R_f = 0.51 (PE:EtOAc, 50:1); ^{1}H NMR (600 MHz, C_6D_6): δ = 5.35 (q, J = 1.4 Hz, 1H, H_1), 4.89 (dd, J = 10.2 Hz, 3.8 Hz, 1H, H_9), 4.79 – 4.77 (m, 1H, H_5), 4.14 (d, J = 11.3 Hz, 1H, H_{11}), 3.65 (d, J = 11.2 Hz, 1H, H_{11}), 2.22 – 2.15 (m, 1H, H_8), 2.10 – 2.03 (m, 3H, H_3, H_4, H_7), 1.95 (ddd, J = 12.6 Hz, 12.6 Hz, 3.9 Hz, 1H, H_7), 1.87 – 1.83 (m, 1H, H_8), 1.80 – 1.78 (m, 1H, H_4), 1.54 (s, 3H, H_{12}), 1.54 (s, 3H, H_{15}), 1.39 – 1.38 (m, 3H, H_{14}), 1.02 (d, J = 6.6 Hz, 3H, H_{13}) ppm; ^{13}C NMR (151 MHz, C_6D_6): δ = 141.3 (C_1), 133.3 (C_6), 131.7 (C_{10}), 131.5 (C_9), 127.2 (C_5), 119.7 (C_2), 78.2 (C_{11}), 40.0 (C_7), 38.2 (C_3), 33.8 (C_4), 25.2 (C_8), 19.4 (C_{13}), 15.0 (C_{14}), 14.7 (C_{15}), 8.7 (C_{12}) ppm; HRMS [GC-MS, CI]: *m/z* calcd for $C_{15}H_{24}O$ $[M]^+$: 220.1827, found: 220.1825.

Biotransformation product 6

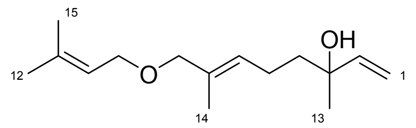

6
$C_{15}H_{26}O_2$
238.37

FPP derivative **3** (137 mg, 305 µmol, 1.00 eq.) was dissolved in an aq. NH_4HCO_3 solution (14 mM, 21.8 mL). The reaction was performed in five 41 mL batches. The following procedure will be described for one 41 mL batch. Tween20® (8 µL) was dissolved in HEPES_2 buffer (36.1 mL). FPP derivative **3** (500 µL) and PvHVS enzyme solution (24.3 mg/mL, 167.1 µL) were added. The reaction was stirred at 29 °C and 50 rpm and every 30 min more

FPP derivative **3** (500 µL, last addition: 400 µL) was added to a total volume of 4.4 mL. After the last addition, PPase (1 µL) was added. The reaction was then allowed to stirr at 29 °C and 50 rpm o/n. The batches were combined and extracted with *n*-pentane (3x). The combined organic phases were dried over $MgSO_4{\cdot}H_2O$, filtered and the solvent was carefully removed *in vacuo*. The crude product was purified by column chromatography (*n*-pentane:Et_2O, 100:1, 5:1) and the isolated compound were prepared by C_6D_6 coevaporation for NMR analysis.

Analytical data of **6**, literature known for $CDCl_3$.[6a]

R_f = 0.22 (*n*-pentane:Et_2O, 4:1); ^{1}H NMR (600 MHz, C_6D_6): δ = 5.72 (dd, *J* = 17.3 Hz, 10.7 Hz, 1H, H_2), 5.54 – 5.52 (m, 1H, H_{10}), 5.49 (t, *J* = 7.2 Hz, 1H, H_6), 5.18 (d, *J* = 17.3Hz, 1H, H_1), 4.94 (d, *J* = 10.8 Hz, 1H, H_1), 3.96 (d, *J* = 6.7 Hz, 2H, H_9), 3.85 (s, 2H, H_8), 2.16 – 2.06 (m, 2H, H_5), 1.70 (s, 3H, H_{14}), 1.60 (s, 3H, H_{12}/H_{15}), 1.52 (s, 3H, H_{12}/H_{15}), 1.49 – 1.40 (m, 2H, H_5), 1.09 (s, 3H, H_{13}) ppm; ^{13}C NMR (151 MHz, C_6D_6): δ = 145.5 (C_2), 135.3 (C_{11}), 133.2 (C_7), 127.6 (C_6), 122.8 (C_{10}), 111.5 (C_1), 76.1 (C_8), 72.9 (C_3), 66.5 (C_9), 42.3 (C_4), 28.3 (C_{13}), 25.7 (C_{12}/C_{15}), 22.8 (C_5), 18.0 (C_{12}/C_{15}), 14.1 (C_{14}) ppm; HRMS [GC-MS, CI]: *m/z* calcd for $C_{15}H_{24}O$ $[M-H_2O]^+$: 220.1827, found: 220.1830.

Biotransformation product 15

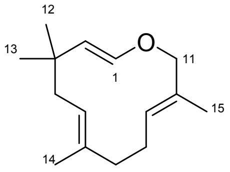

15
$C_{15}H_{24}O$
220.36

FPP derivative **3** (138 mg, 307 µmol, 1.00 eq.) was dissolved in an aq. NH_4HCO_3 solution (14 mM, 21.9 mL). The reaction was performed in six 34 mL batches. The following procedure will be described for one 34 mL batch. Tween20® (7 µL) was dissolved in HEPES_2 buffer (30.2 mL). FPP derivative **3** (500 µL) and PvHVS enzyme solution (13.3 mg/mL, 257 µL) were added. The reaction was stirred at 29 °C and 50 rpm and every 30 min more FPP derivative **3** (500 µL, last addition: 200 µL) was added to a total volume of 3.7 mL. After the last addition, PPase (1 µL) was added. The reaction was then allowed to stirr at 29 °C and 50 rpm o/n. The batches were combined and extracted with *n*-pentane (3x). The combined organic phases were dried over $MgSO_4{\cdot}H_2O$, filtered and the solvent was carefully removed *in vacuo*. The crude product was purified by column chromatography (*n*-pentane:Et_2O 400:1, 100:1) and the isolated compound were prepared by C_6D_6 coevaporation for NMR analysis.

R_f = 0.32 (*n*-pentane:Et_2O, 100:1); ^{1}H NMR (600 MHz, C_6D_6): δ = 5.63 (d, *J* = 12.5 Hz, 1H, H_1), 5.02 (t, *J* = 7.6 Hz, 1H, H_5), 4.91 (t, *J* = 7.8 Hz, 1H, H_9), 4.79 (d, *J* = 12.4 Hz, 1H, H_2), 3.91 (s, 2H, H_{11}), 2.06 – 2.02 (m, 4H, H_7, H_8), 1.89 (d, *J* = 7.7 Hz, 2H, H_4), 1.51 (s, 3H, H_{15}), 1.38 (s, 3H, H_{14}), 1.02 (s, 6H, H_{12}, H_{13}) ppm; ^{13}C NMR (151 MHz, C_6D_6): δ = 143.8 (C_1), 134.2 (C_6), 131.1 (C_{10}), 131.0 (C_9), 124.3 (C_5), 119.9 (C_2), 76.9 (C_{11}), 42.0 (C_4), 39.9 (C_7), 35.0 (C_3),

28.2 (C_{12}, C_{13}), 25.2 (C_8), 15.2 (C_{14}), 14.5 (C_{15}) ppm; HRMS [GC-MS, CI]: *m/z* calcd for $C_{15}H_{24}O$ $[M]^+$: 220.1827, found: 220.1826.

Biotransformation product 29

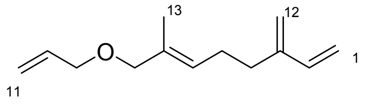

29
$C_{13}H_{20}O$
192.3020

FPP derivative **10** (134 mg, 697 µmol, 1.00 eq.) was dissolved in an aq. NH_4HCO_3 solution (0.05 mM, 16 mL). The reaction was performed in four 50 mL batches. The following procedure will be described for one 50 mL batch. Tween20® (10 µL), PPase (1µL) and $MgCl_2$ (2 M, 250 µL) were dissolved in HEPES buffer (45.5 mL). FPP derivative **10** solution (500 µL) and BcBot2 F138V solution (20.7 mg/mL, 121 µL) were added. The reaction was stirred at 37 °C and 100 rpm. Every 30 min more FPP derivative **10** solution (500 µL) was added to a total volume of 4.0 mL. Halfway through the period more enzyme solution (20.7 mg/mL, 121 µL) was added. The reaction was then allowed to stirr at 37 °C and 100 rpm o/n. The batches were combined and extracted with *n*-pentane (3x). The combined organic phases were washed with an aq. sat. NaCl solution, dried over $MgSO_4{\cdot}H_2O$, filtered and the solvent was carefully removed *in vacuo* and under a stream of N_2. The crude product (9 mg) was purified by column chromatography (*n*-pentane:Et_2O 100:1, 45:1) which resulted in a non seperable mixture of compounds with one major product. In order to isolate this compound we used a preparative gas chromatography (see general information).

R_f = 0.17 (PE:EtOAc, 100:1); ^1H NMR (600 MHz, C_6D_6): δ = 6.34 (dd, J = 17.6 Hz, 10.6 Hz, 1H, H_2), 5.88 (ddt, J = 17.2 Hz, 10.5 Hz, 5.3 Hz, 1H, H_{10}), 5.45 – 5.42 (m, 1H, H_6), 5.28 (ddt, J = 17.2 Hz, 1.8 Hz, 1.8 Hz, 1H, H_{11}), 5.17 (d, J = 17.5 Hz, 1H, H_1), 5.07 – 5.04 (m, 1H, H_{11}), 4.97 – 4.95 (m, 3H, H_1, H_{12}), 3.81 (dt, J = 5.2 Hz, 1.5 Hz, 2H, H_9), 3.79 (s, 2H, H_8), 2.21 (m, 4H, H_4, H_5), 1.61 (s, 3H, H_{13}) ppm; ^{13}C NMR (151 MHz, C_6D_6): δ = 146.2 (C_3), 139.3 (C_2), 135.8 (C_{10}), 133.2 (C_7), 127.2 (C_6), 116.1 (C_{12}), 115.9 (C_{11}), 113.2 (C_1), 76.2 (C_8), 70.5 (C_9), 31.5 (C_4), 26.6 (C_5), 14.0 (C_{13}) ppm; HRMS [GC-MS, CI]: *m/z* calcd for $C_{13}H_{21}O$ $[M+H]^+$: 193.1592, found: 193.1600.

1.3 Microbiological methods and biotransformations

1.3.1 Procedure A

Heterologous protein expression and cell lysis *via* ultrasound

In order to cultivate the *E.coli* BL21 (DE3) cells, carrying the required plasmids, a seed culture (50 µL) was incubated with kanamycin (50 mg/mL, 3 µL) in LB-media (3 mL) for approximately 4.5 h at 37 °C and 200 rpm. Alternatively, a seed culture (5 µL) can be incubated with kanamycin (50 mg/mL, 5 µL) in LB-Media (5 mL) at 37 °C and 180 rpm o/n. From this

pre-culture (1 mL) a main culture was created by incubation with kanamycin (50 mg/mL) in 2-TY media (50 mL) at 37 °C and 200 rpm until the culture reached an OD_{600} value of a 0.4 to 0.8. To initiate the protein overexpression IPTG (1 M, 25 µL or 50 µL) was added to the culture that was stirred at 16 °C and 180 rpm for approximately 22 h. After centrifugation, the cell pellets were stored at −20 °C or used immediately for cell lysis. Cells were resuspended in lysis buffer (20 mL) at 0 °C and lysed by ultrasonication (10 min, 45% amplitude, 4 s ultrasound to 6 s pause). The resulting solution was centrifuged (4 °C, 20 min, 10000 g) to give the crude enzyme solution.

Immobilized metal-affinity chromatography

For conditioning the column was rinsed with water (10x the column volume) and lysis buffer (5x column volume). The lysate was loaded onto the column (2x) and eluted with Ni-NTA buffers (5 mL each) with increasing imidazole concentrations (25 mM, 50 mM, 100 mM, 250 mM, 500 mM). During this time the solutions were cooled at 0 °C. The fractions were analysed using a qualitative Brentford assay and those fractions containing protein were united and concentrated by centrifugation (4 °C, 4500 rpm).

Buffer exchange

To perform the buffer exchange the column was rinsed with water (10x column volume) and HEPES buffer (5x column Volume). The protein solutions were loaded onto the column and eluted with HEPES buffer (5 mL). After centrifugation (4 °C, 4500 rpm) the solutions can be used or stored as a mixture of water and glycerol (1/1) between −70 °C and −80 °C.

Concentration measurement

Concentrations were determined by measuring the absorption ($\lambda = 280$ nm) of the purified protein solutions, using the extinction coefficient for reduced cysteine side chains.

***In-vitro* biotransformation (analytical scale)**

Screening for new biotransformation products was performed in a reaction scale of 500 µL containing the corresponding enzyme (50 µg), the FPP-derivatives **9** + **10** (1.5 µL, 50 mM) and a $MgCl_2$ solution (1.25 µL, 2 M). In parallel also negative (without FPP derivative or in the absence of enzymes) as well as positive control experiments were performed (using FPP (**1**)) under analogous conditions. Reactions using FPP derivatives were carried out in HEPES buffer (pH = 7.5) at 37 °C and 100 rpm o/n. Positive controls using FPP (**1**) were performed at 30 °C and 100 rpm. In order to extract the products *n*-hexane was added and the phases were separated by centrifugation (3000 rpm, 6 min, 4 °C). The hexane extract was used for GC-MS analysis.

1.3.2 Procedure B

Heterologous protein expression and cell lysis *via* ultrasound

In order to cultivate the *E.coli* BL21 (DE3) cells, carrying the required plasmids, a seed culture (5 µL) was incubated with kanamycin (50 mg/mL, 5 µL) in LB-media (5 mL) at 37 °C and 200 rpm o/n. From this pre-culture (1 mL) a main culture was created by incubation with kanamycin (50 mg/mL, 50 µL) in 2-TY media (50 mL) at 37 °C and 200 rpm until the culture

reached an OD_{600} value of a 0.4 to 0.8. To initiate the protein overexpression IPTG (1 M, 50 μL) was added to the culture that was stirred at 16 °C and 180 rpm for approximately 22 h. After centrifugation, the cell pellets were stored at −20 °C or used immediately for cell lysis. Cells were resuspended in lysis buffer (15 mL) at 0 °C and lysed by ultrasonication (10 min, 45% amplitude, 4 s ultrasound to 6 s pause). The resulting solution was centrifuged (4 °C, 20 min, 10000 g) to give the crude enzyme solution.

Immobilized metal-affinity chromatography

For conditioning the column was rinsed with water (10x the column volume) and lysis buffer (5x column volume). The lysate was loaded onto the column (2x) and eluted with Ni-NTA buffers (5 mL each) with two different imidazole concentrations (25 mM, 250 mM). During this time the solutions were cooled at 0 °C. The fractions were analysed using a qualitative Brentford assay and those fractions containing protein were united and concentrated by centrifugation (4 °C, 4500 rpm).

Buffer exchange

To perform the buffer exchange the column was rinsed with water (10x column volume) and HEPES buffer (5x column Volume). The protein solutions were loaded onto the column and eluted with HEPES buffer (5 mL). After centrifugation (4 °C, 4500 rpm) the solutions can be used or stored as a mixture of water and glycerol (1/1) between −70 °C and −80 °C.

Concentration measurement

Concentrations were determined by measuring the absorption ($\lambda = 280$ nm) of the purified protein solutions, using the extinction coefficient for reduced cysteine side chains.

***In-vitro* biotransformation (analytical scale)**

Screening for new biotransformation products was performed in a reaction scale of 500 μL containing the corresponding enzyme (50 μg), the FPP-derivative **3** (1.5 μL, 50 mM) and a $MgCl_2$ solution (1.25 μL, 2 M). In parallel also negative (without FPP derivative or in the absence of enzymes) as well as positive control experiments were performed (using FPP (**1**)) under analogous conditions. Reactions were carried out in HEPES buffer (pH = 7.5) for 30 min at 30 °C. In order to extract the products *n*-hexane was added and the phases were separated by centrifugation (3000 rpm, 8 min, 4 °C). The hexane extract was used for GC-MS analysis.

1.4 GC-MS Data

1.4.1 Positive and Negative Controls

Before screening derivatives for new terpenoids, the qualitative enzyme activity was investigated by incubation of each enzyme with FPP **1** (see Figure S **1**). To see whether the potential new compounds are specific products obtained by the combination of a FPP derivative and the respective enzymes, negative controls using no FPP or a derivative were screened (see Figure S **2**). In the same manner every FPP derivative was tested without enzyme (Figure S **3**).

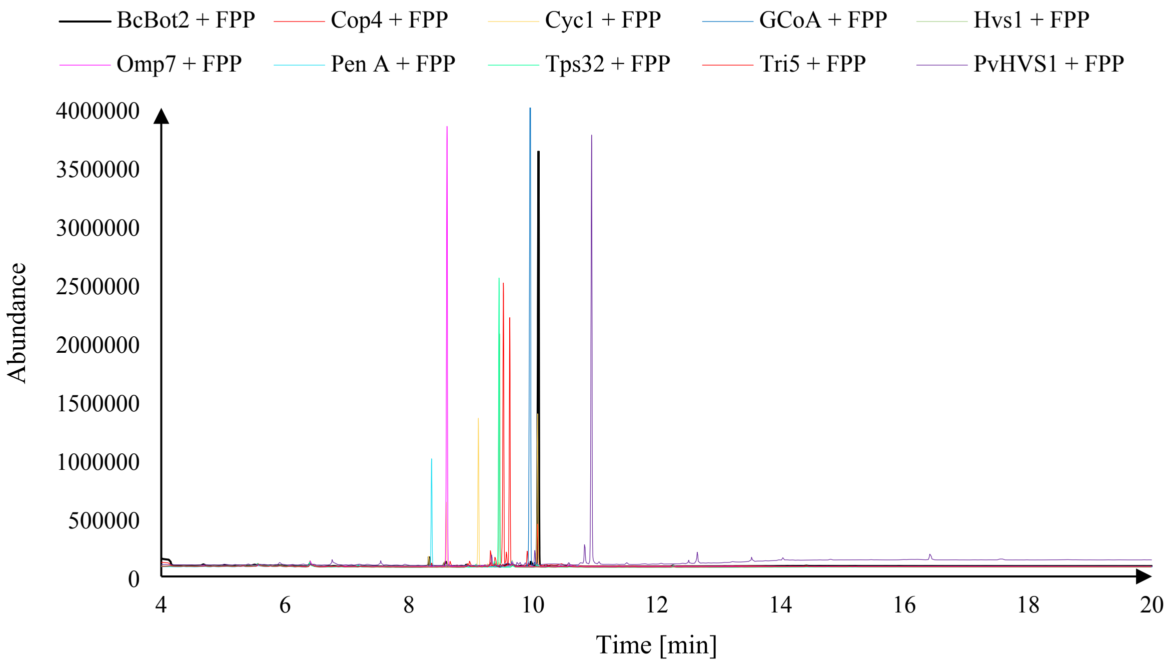

Figure S **1**. Overlay of all chromatograms for FPP (**1**) and the respective enzymes.

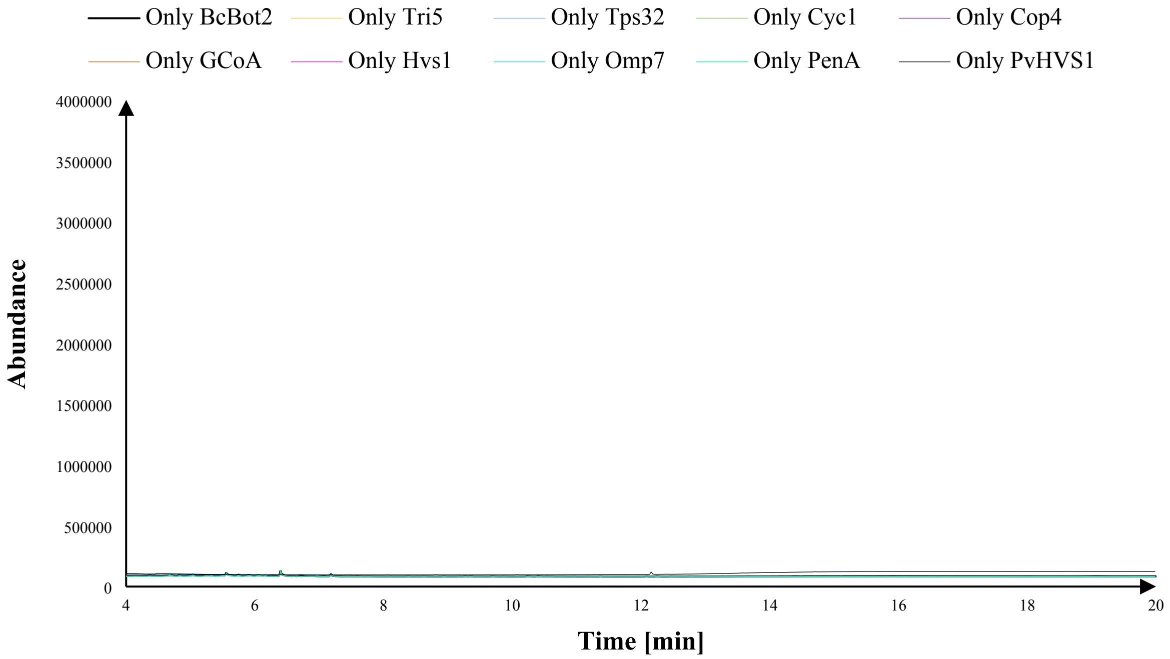

Figure S **2**. Overlay of all chromatograms using nine differents STCs and no substrates.

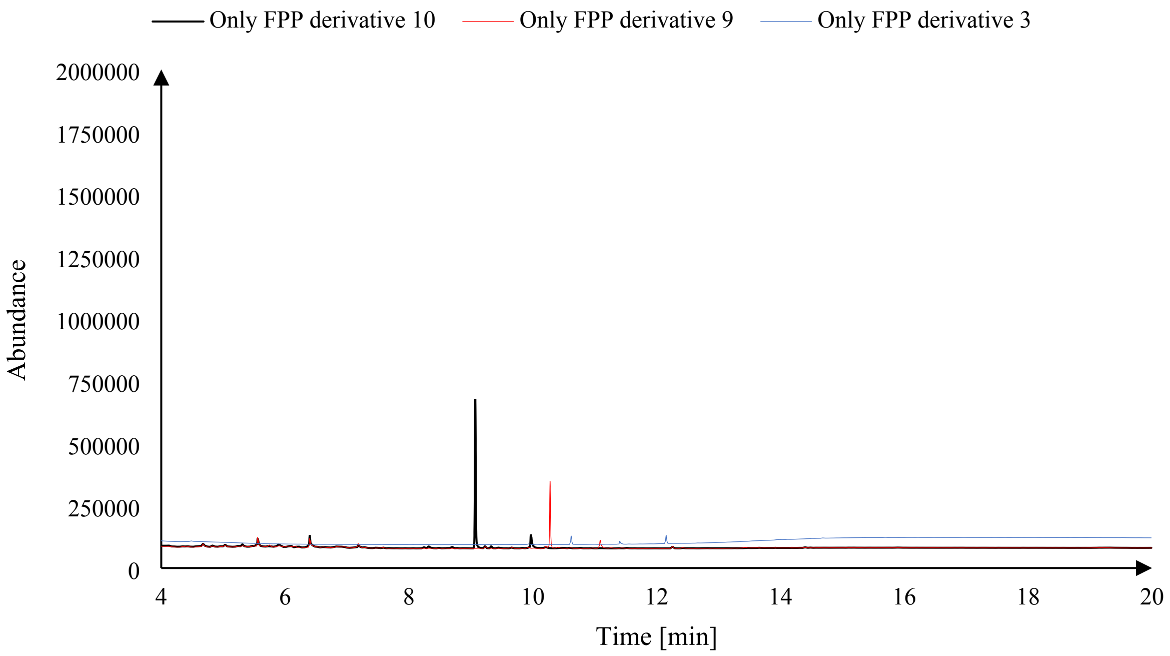

Figure S **3**. Overlay of chromatograms for FPP derivatives **3** + **9** + **10** without enzymes.

1.4.2 Biotransformations Using FPP Derivative 10

For FPP derivative **10** nine different sesquiterpene synthases were tested. An overlay of resulting chromatograms is shown below (see Figure S **4**). The most promising results were observed for Tri5, Omp7, BcBot2. Those three enzymes were therefore selected for up-scaling and product isolation. The corresponding GC-MS data will be displayed below.

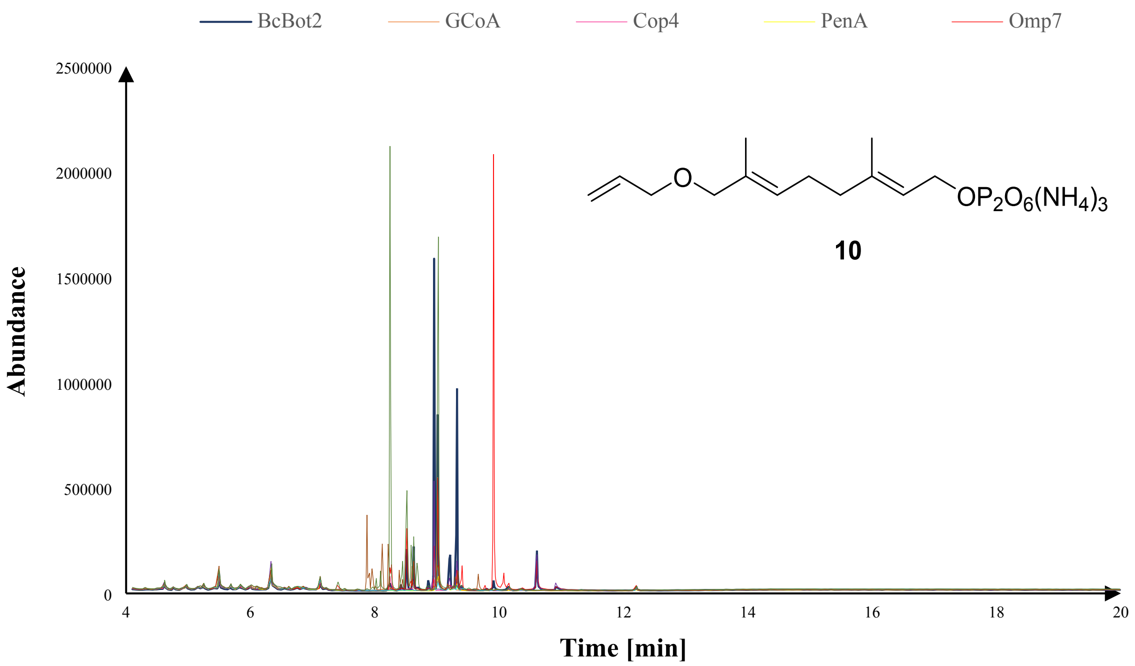

Figure S **4**. Overlay of chromatograms using nine differents STCs and FPP derivative **10**.

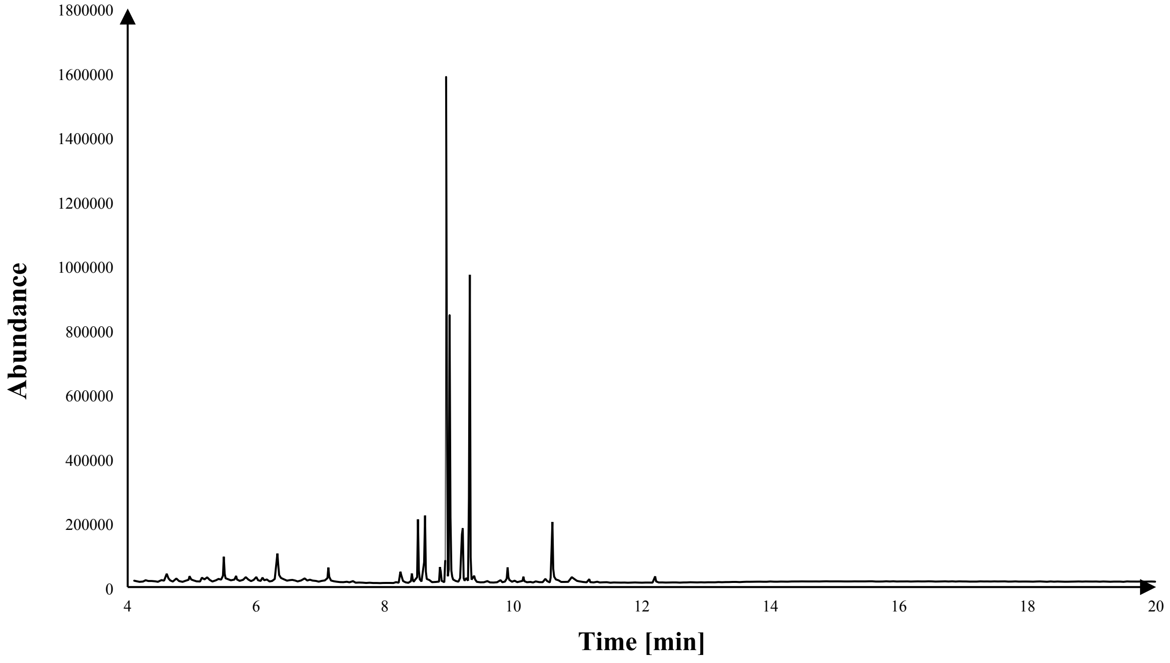

Figure S **5.** Chromatogram of the STC BcBot2 and FPP derivative **10**.

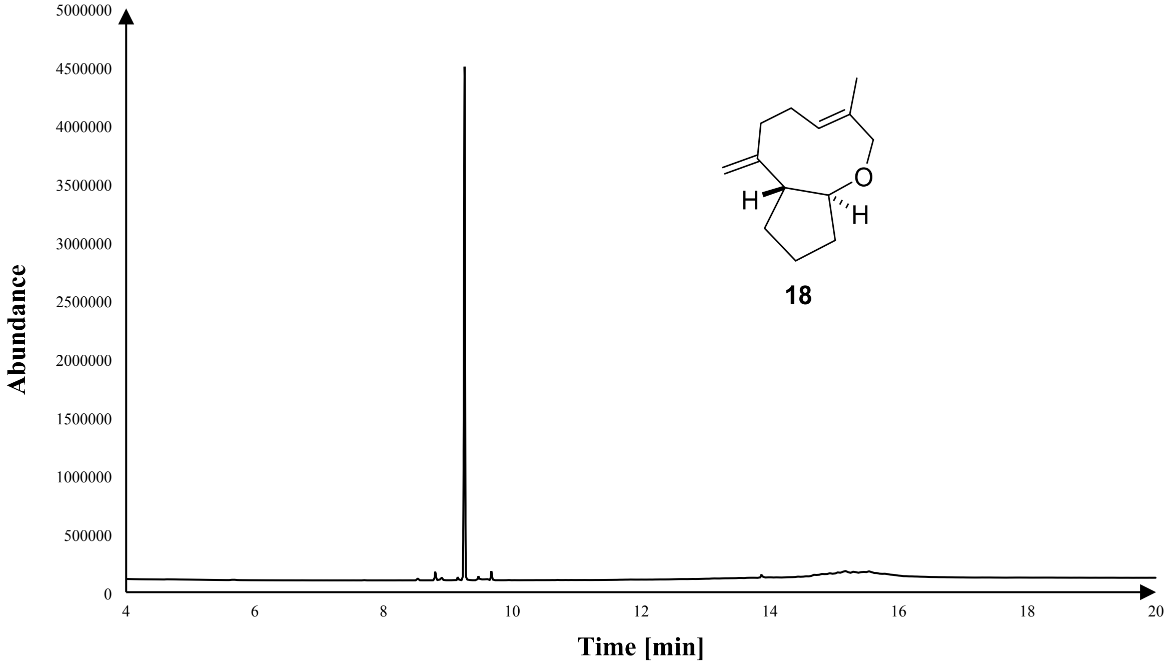

Figure S **6**. Chromatogram of isolated compound **18**.

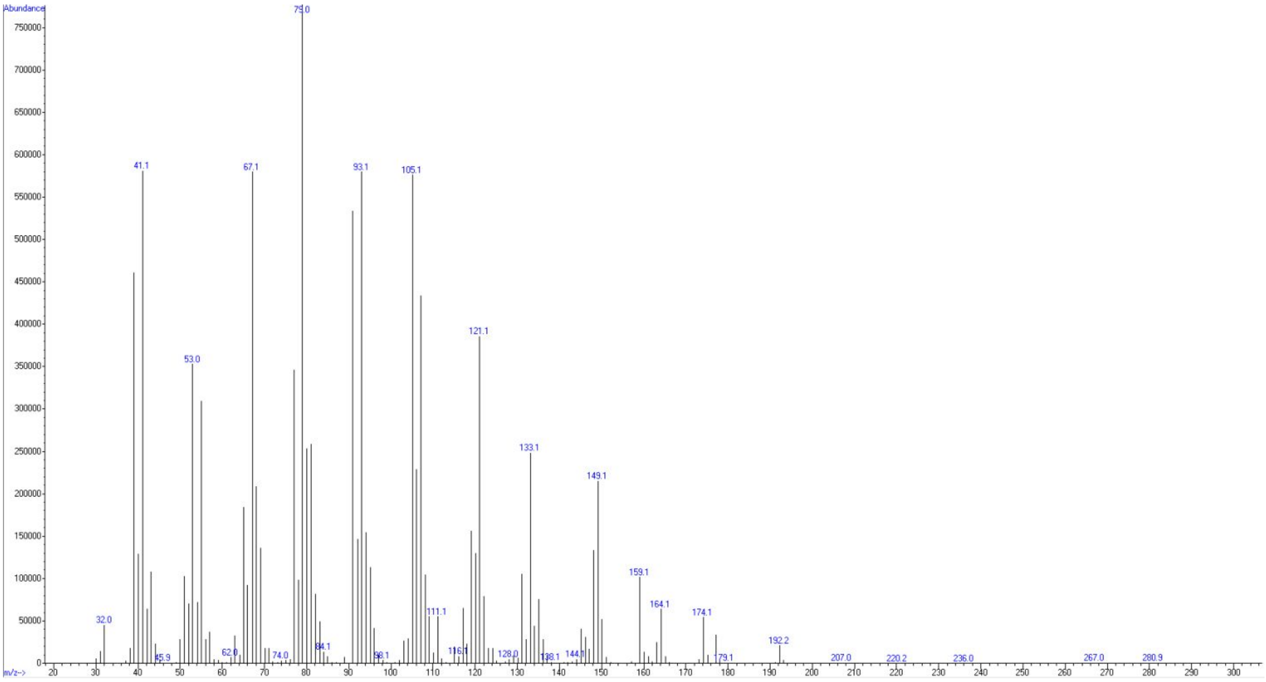

Figure S **7**. MS-data of compound **18**.

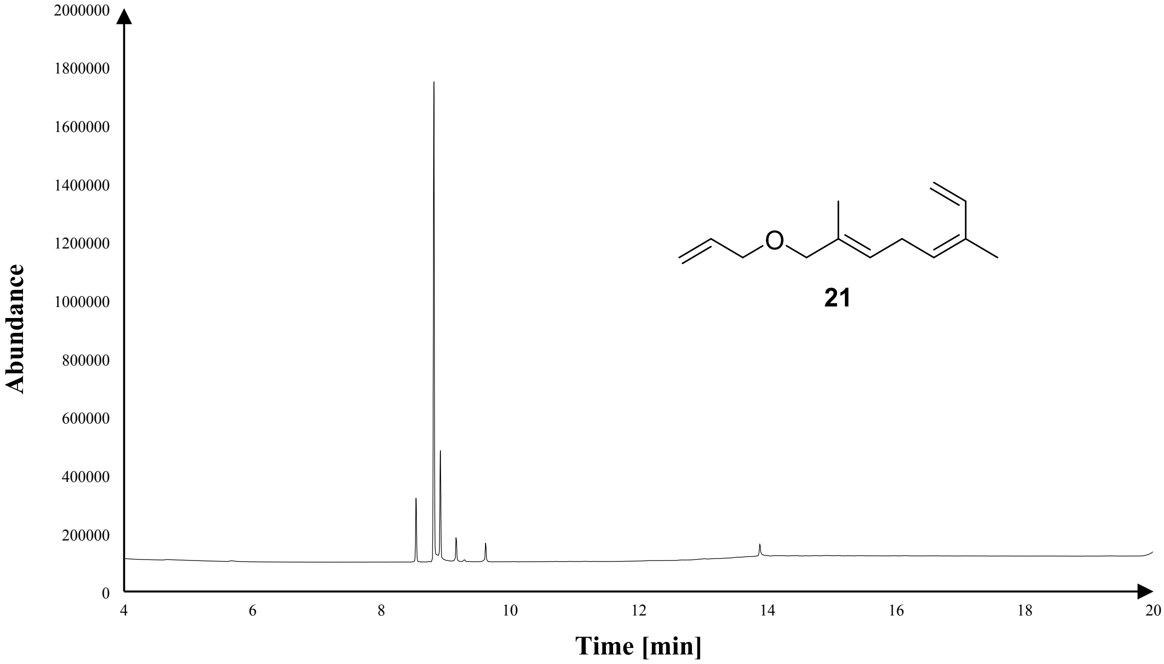

Figure S **8**. Chromatogram of isolated compound **21**.

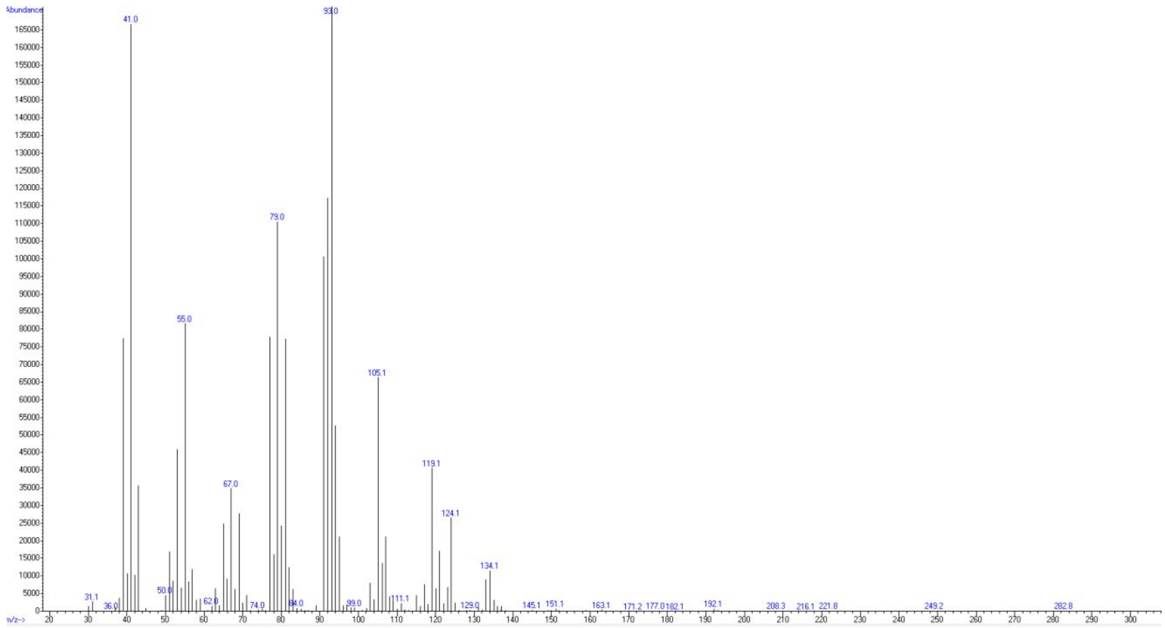

Figure S **9**. MS-data of compound **21**.

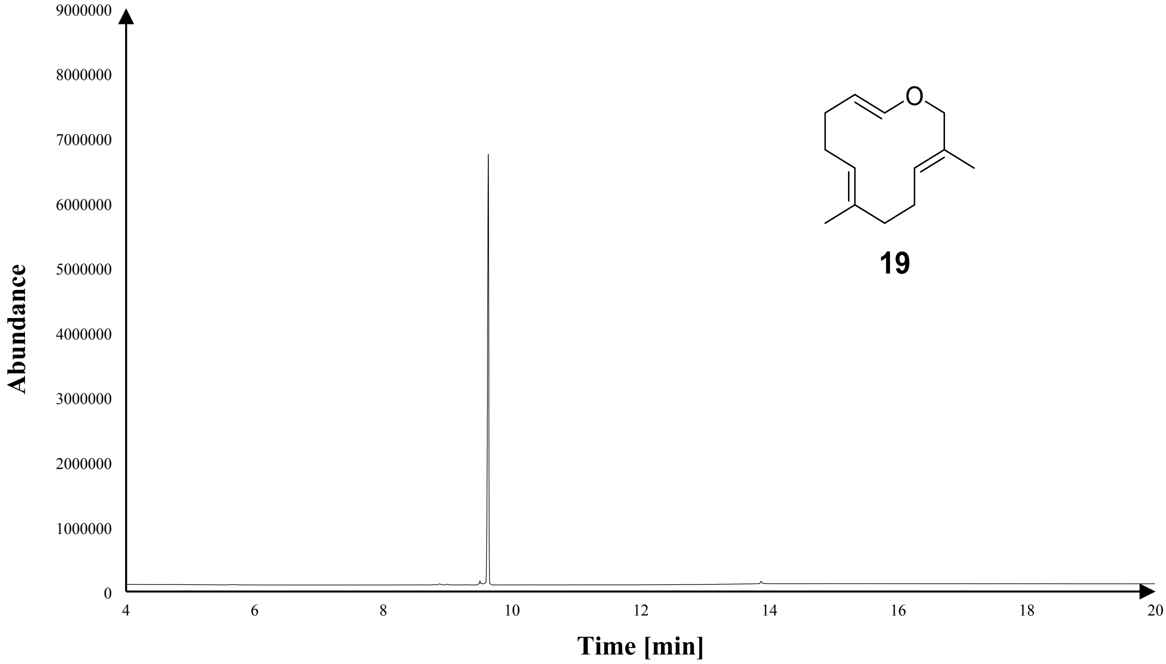

Figure S **10**. Chromatogram of isolated compound **19**.

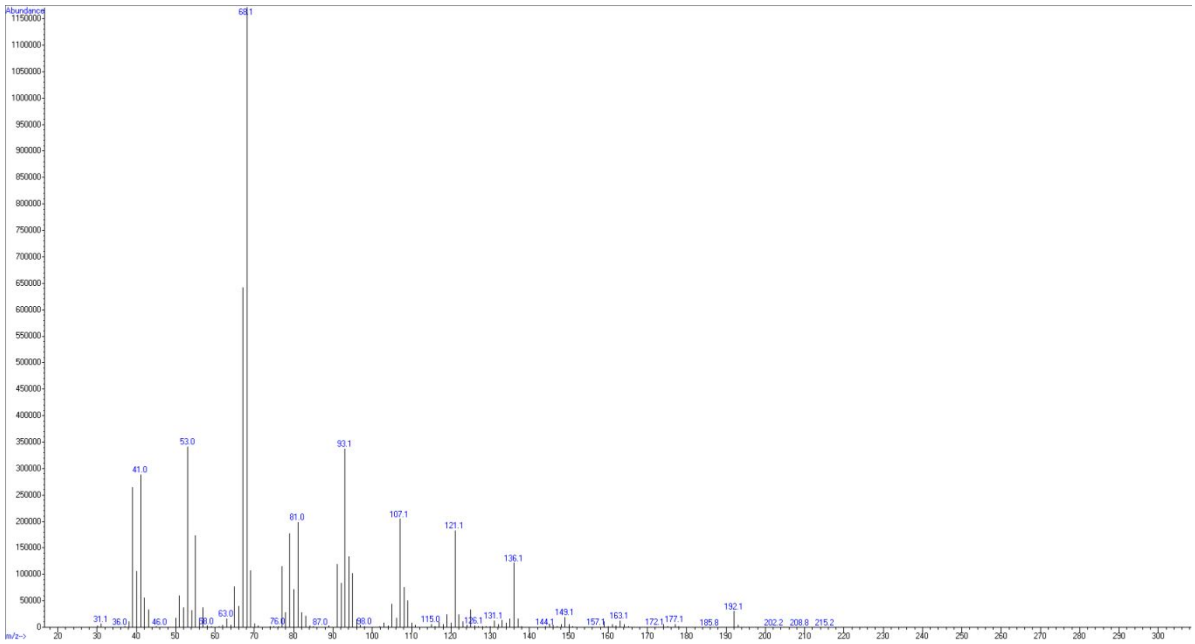

Figure S **11**. MS-data of compound **19**.

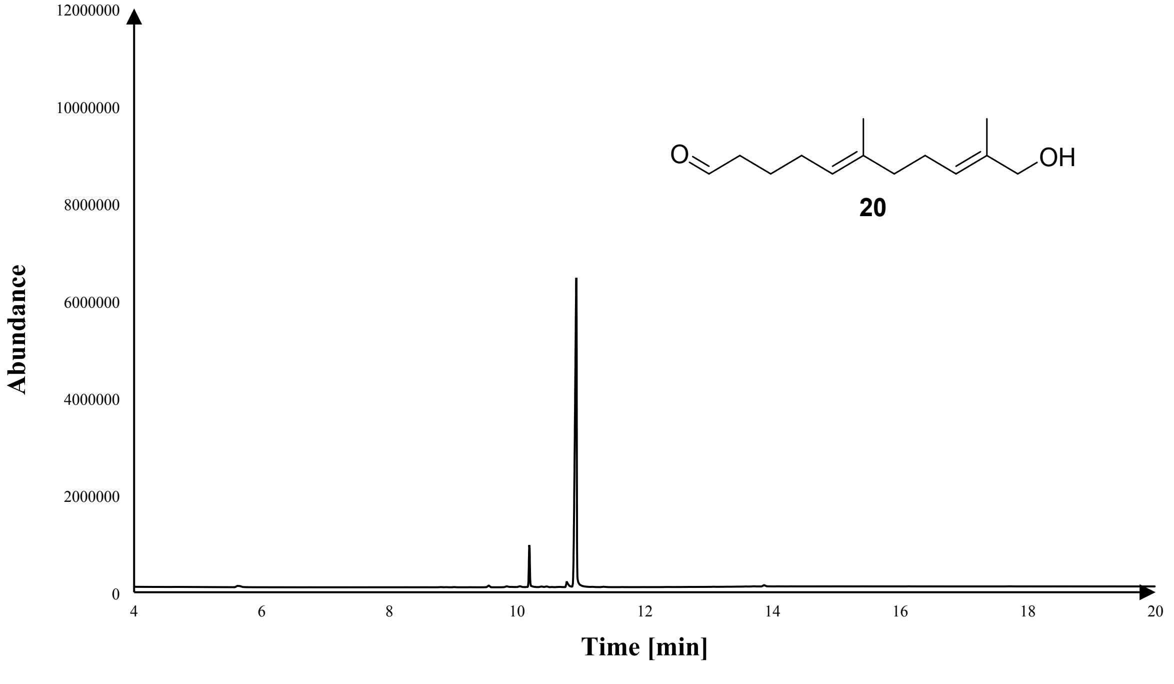

Figure S **12**. Chromatogram of isolated compound **20**.

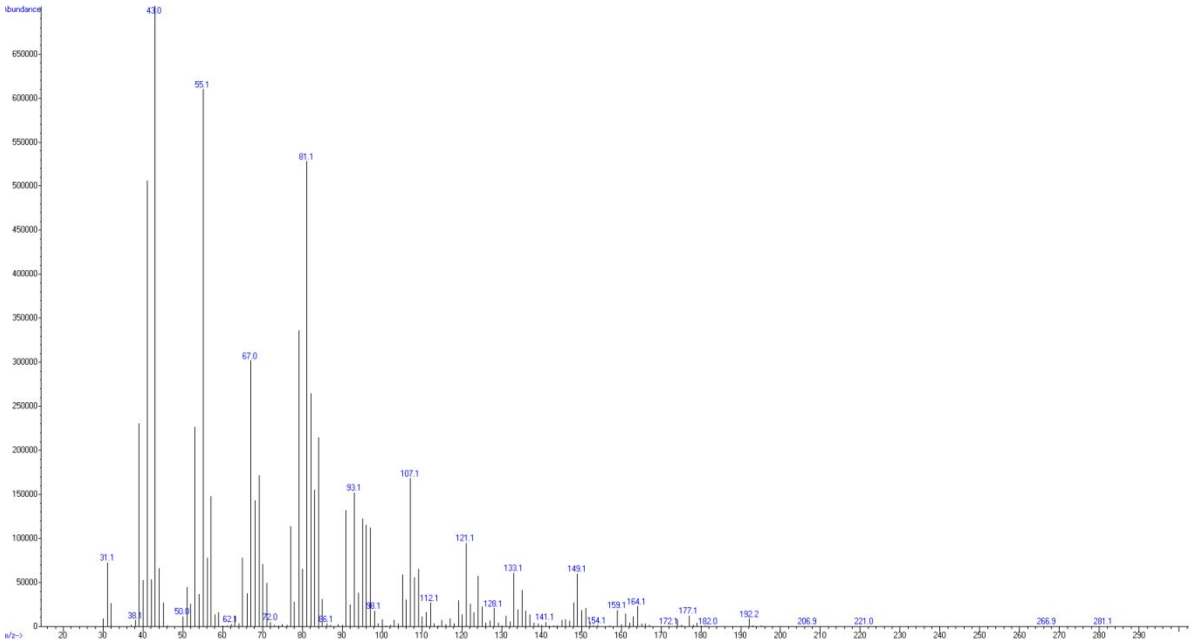

Figure S **13**. MS-data of compound **20**.

Despite promising results using Tri5 in the analytical *in vitro* scale, the up-scaling led to the production of the tertiary Alcohol **23** who is also produced in the negative control using only $MgCl_2$ and no enzymes, while the production of the compound at 8.2 min decreased to a minimum. A product isolation was therefore not possible. Since also Omp7 is producing aldehyde **20** as the main poduct, no further cyclization products were obtained.

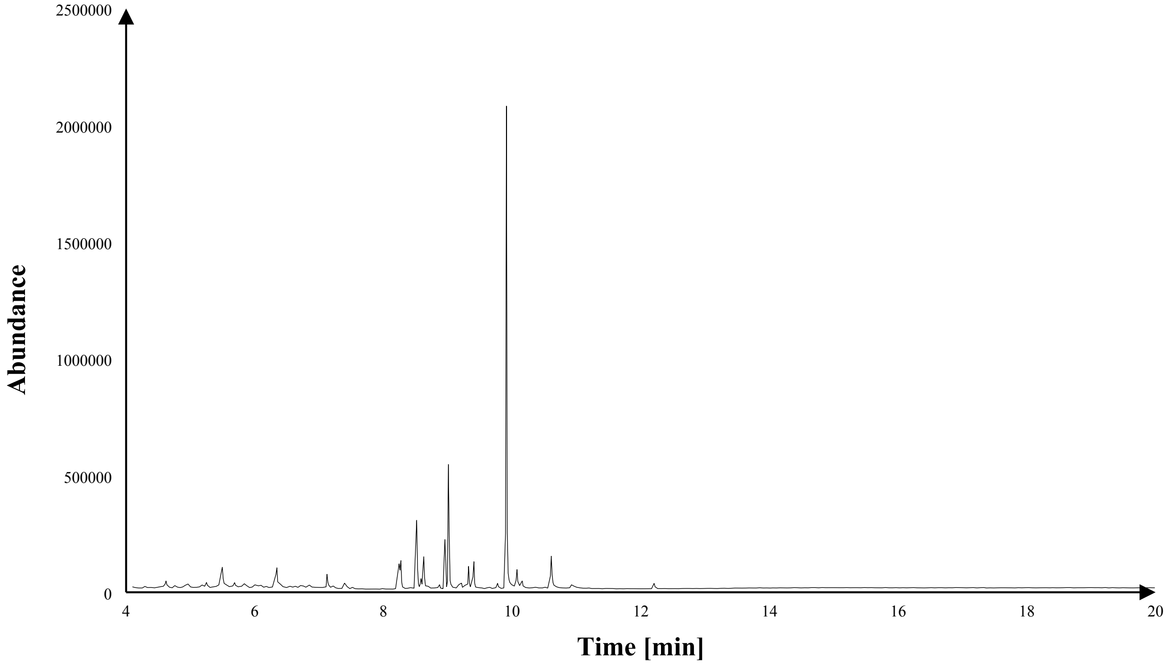

Figure S **14**. Chromatogram of the STC Omp7 and FPP derivative **10**.

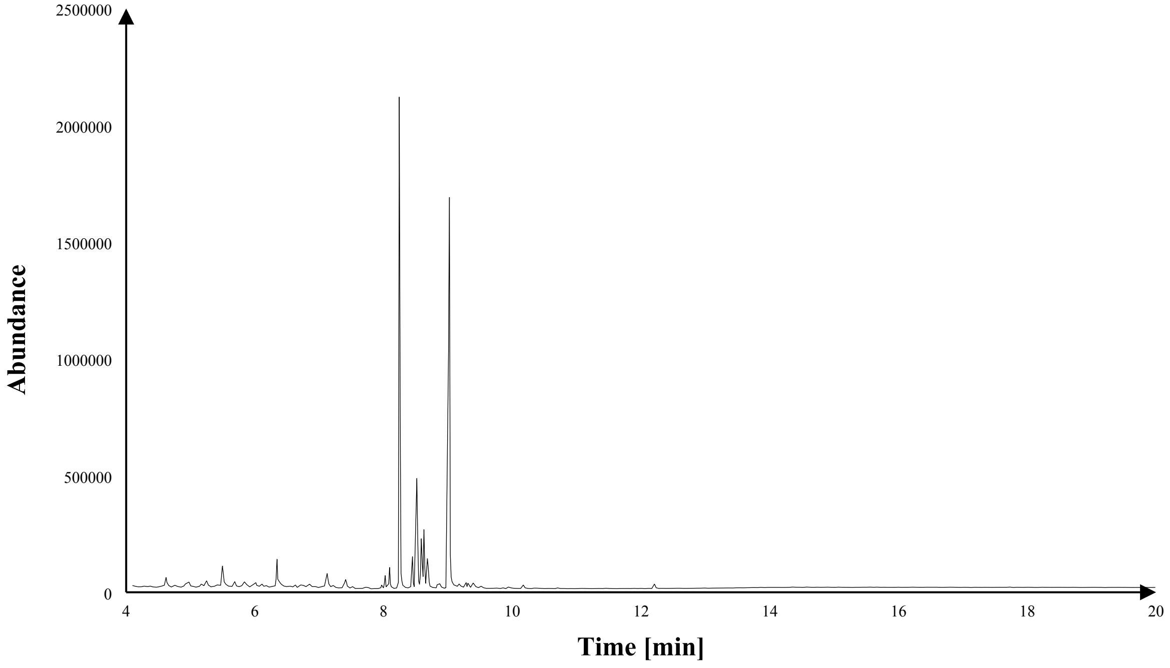

Figure S **15**. Chromatogram of the STC Tri5 and FPP derivative **10**.

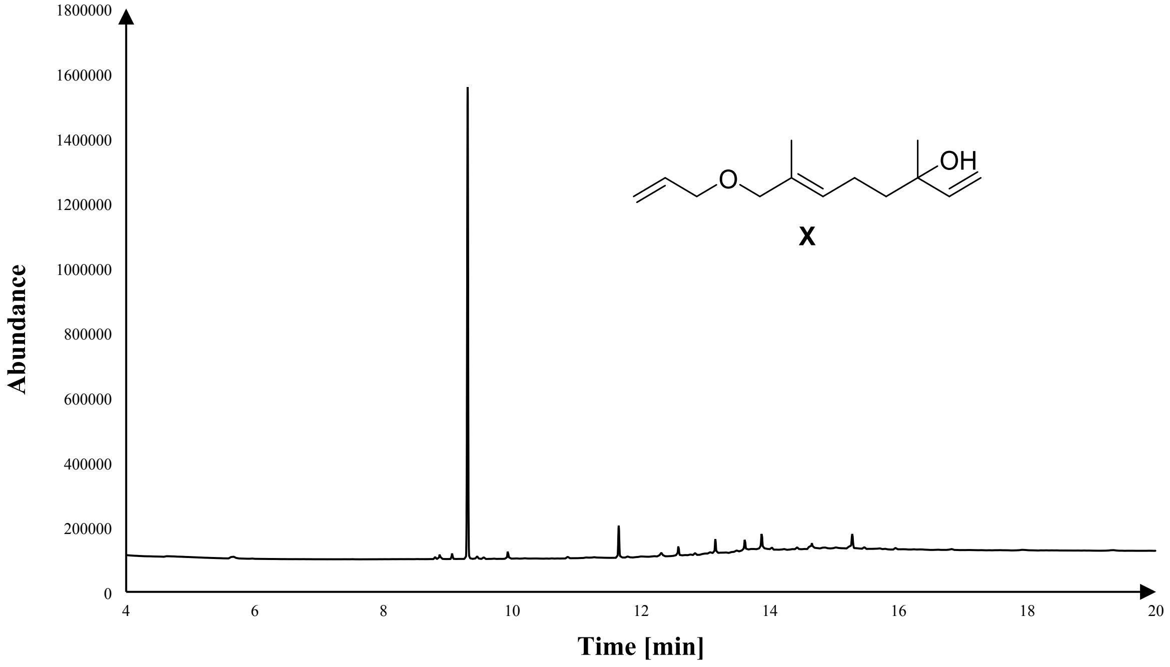

Figure S **16**. Chromatogram of isolated compound **23**.

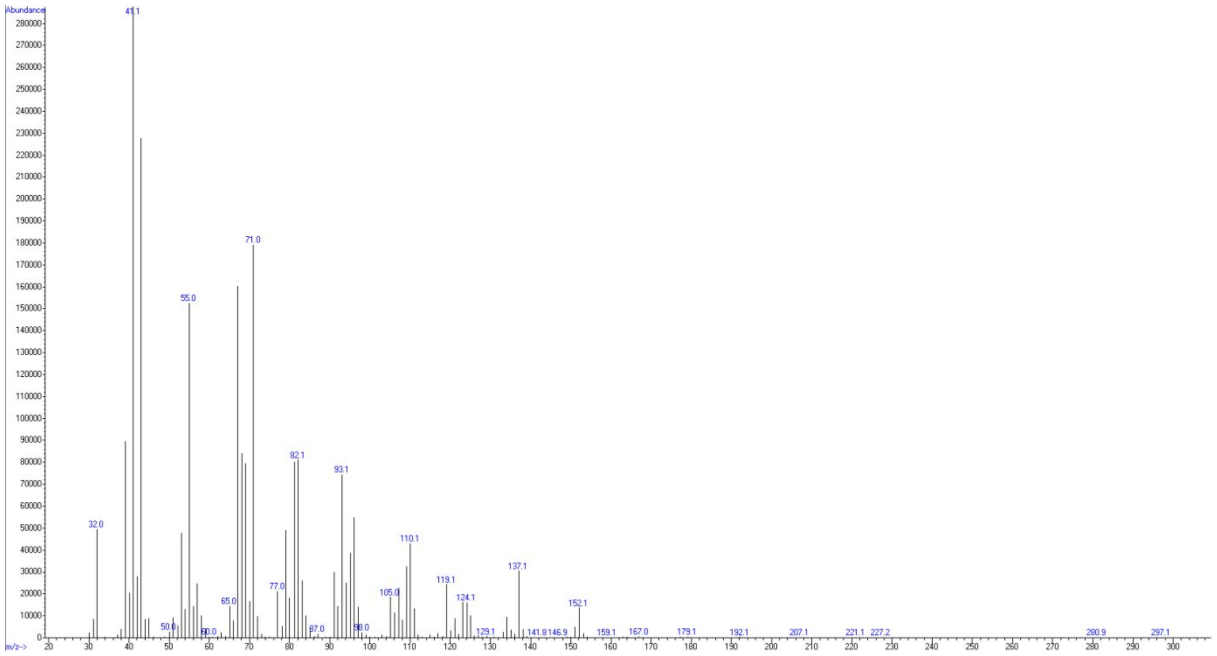

Figure S **17**. MS-data of compound **23**.

1.4.3 Biotransformations using FPP Derivative 9

For FPP derivative **9** nine different sesquiterpene synthases were tested. An overlay of resulting chromatograms is shown below (see Figure S **18**). The most promising results were observed for PenA and Omp7. These two enzymes were therefore selected for up-scaling and product isolation. The corresponding GC-MS data will be displayed below.

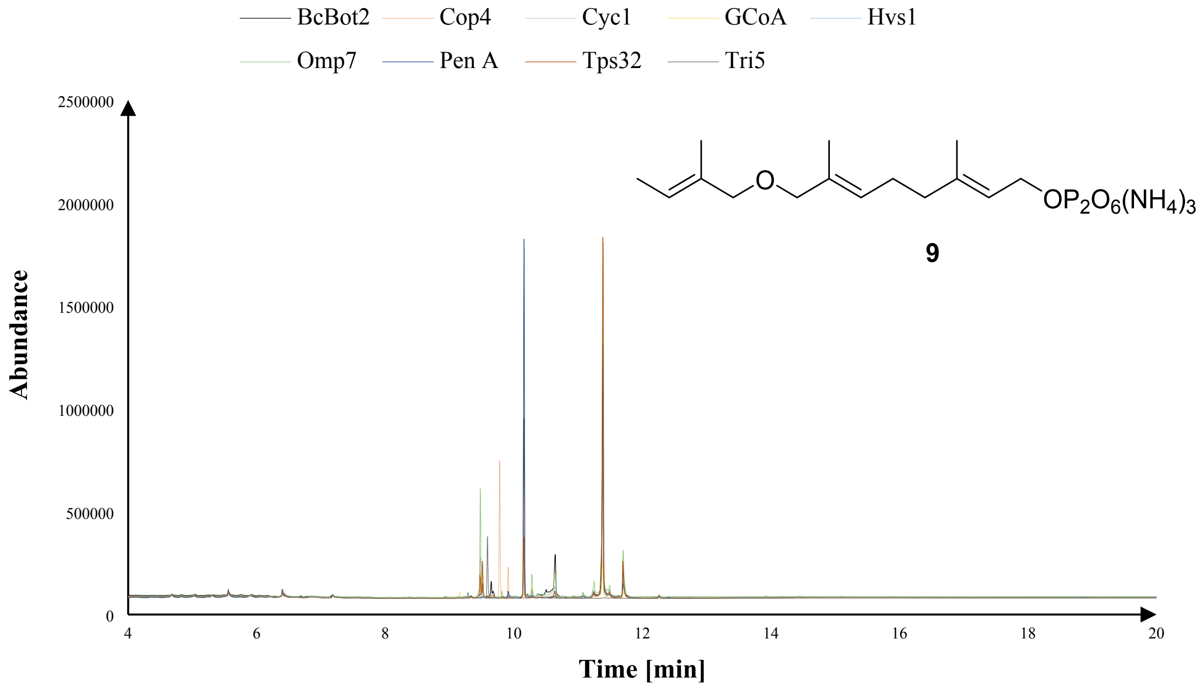

Figure S **18**. Overlay of all chromatograms using nine differents STCs and FPP derivative **9**.

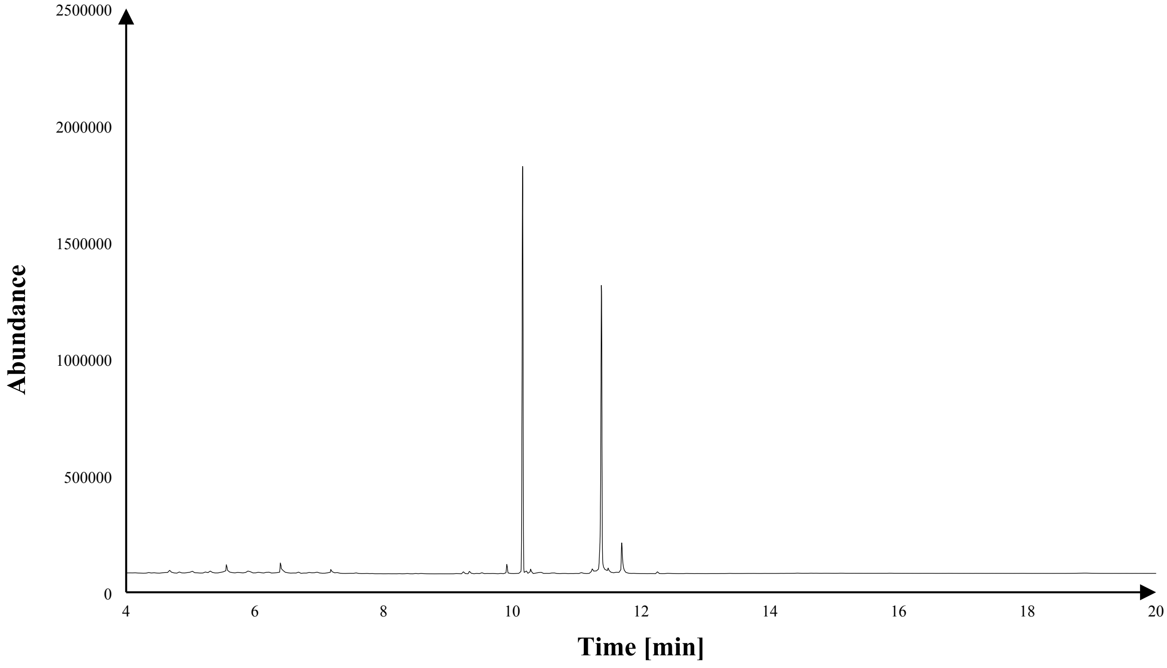

Scheme **19**. Chromatogram of the STC PenA and FPP derivative **9**.

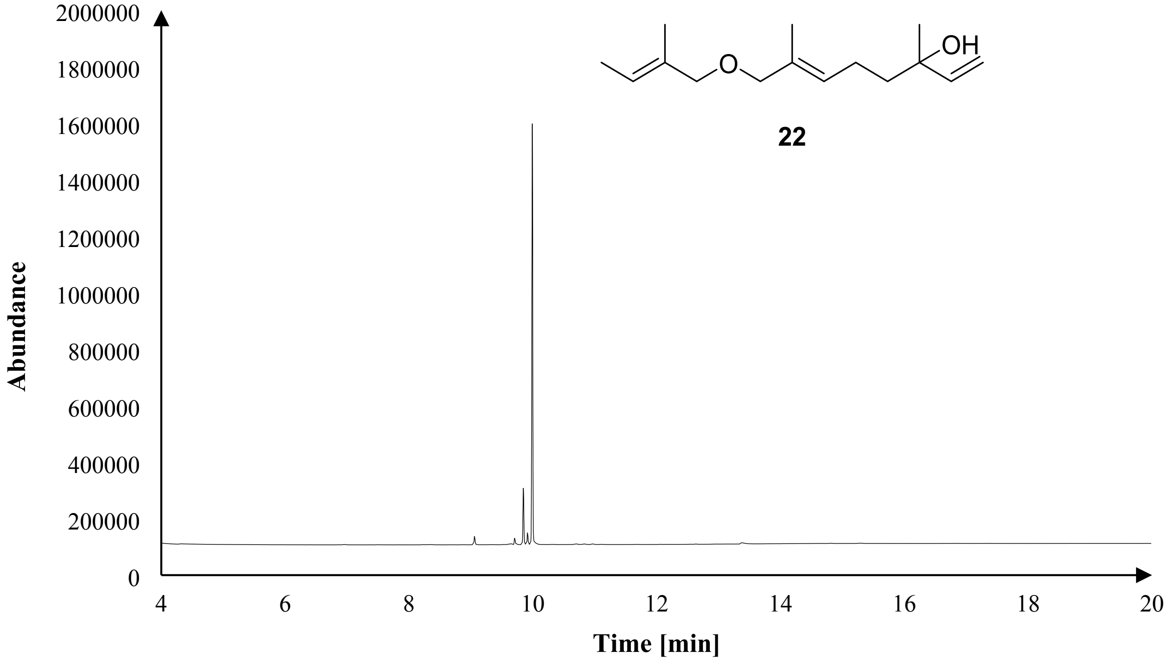

Figure S **20**. Chromatogram of isolated compound **22**.

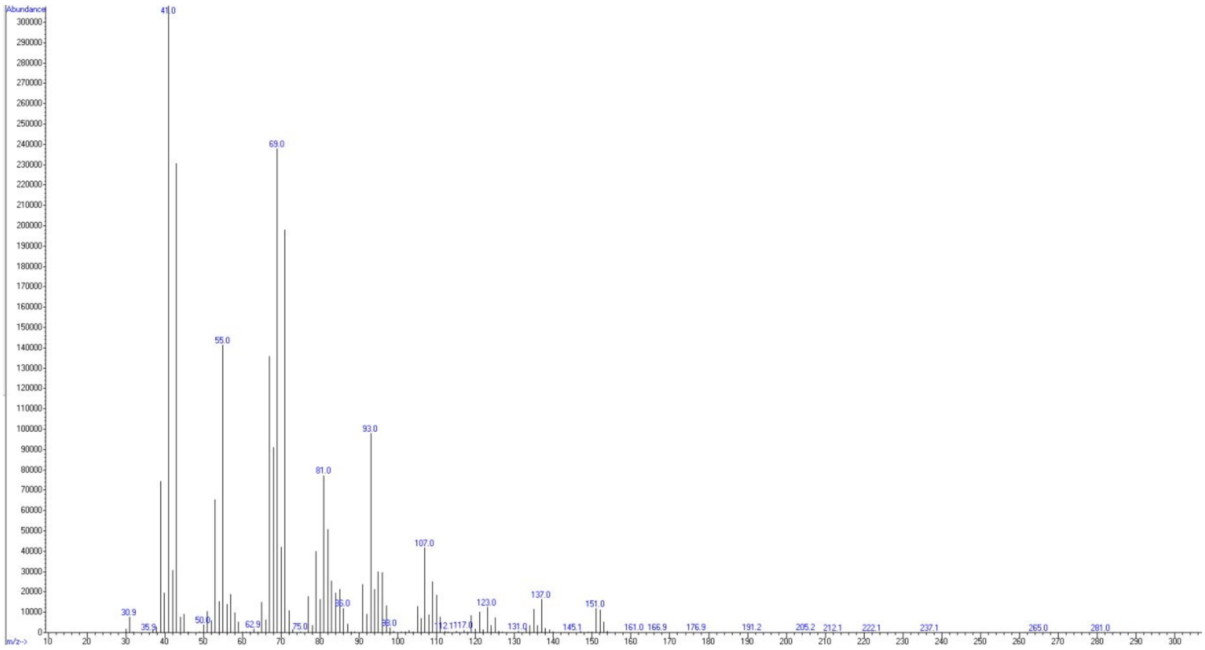

Figure S **21**. MS-data of compound **22**.

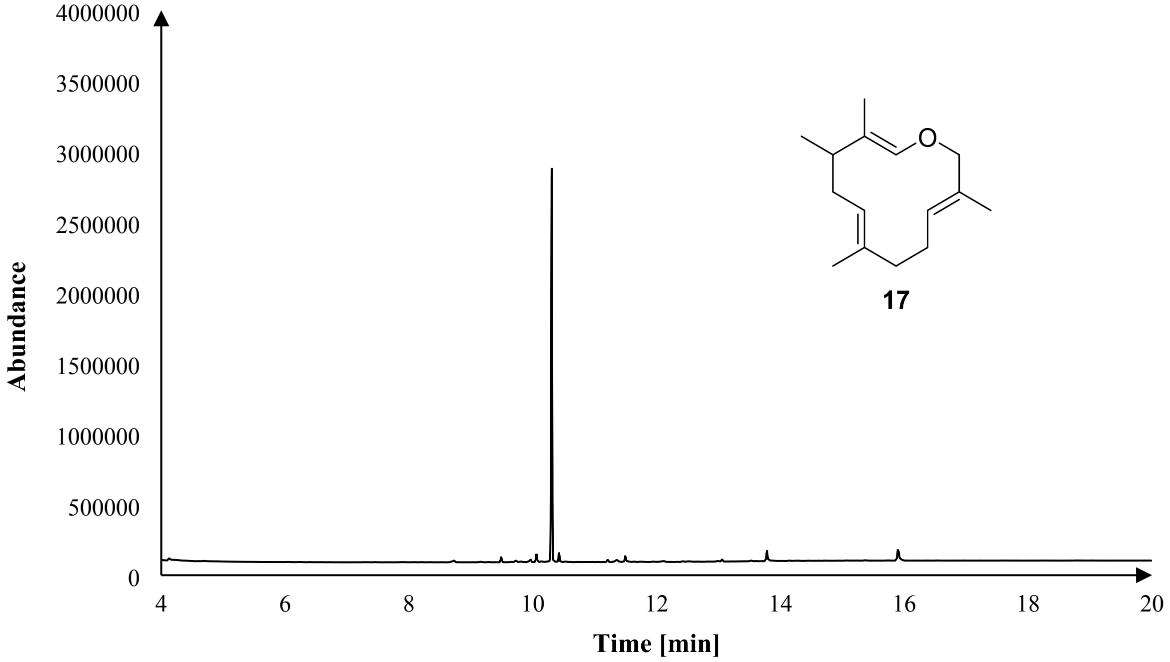

Figure S **22**. Chromatogram of isolated compound **17**. Measurement was performed after several months which leads to different retention times.

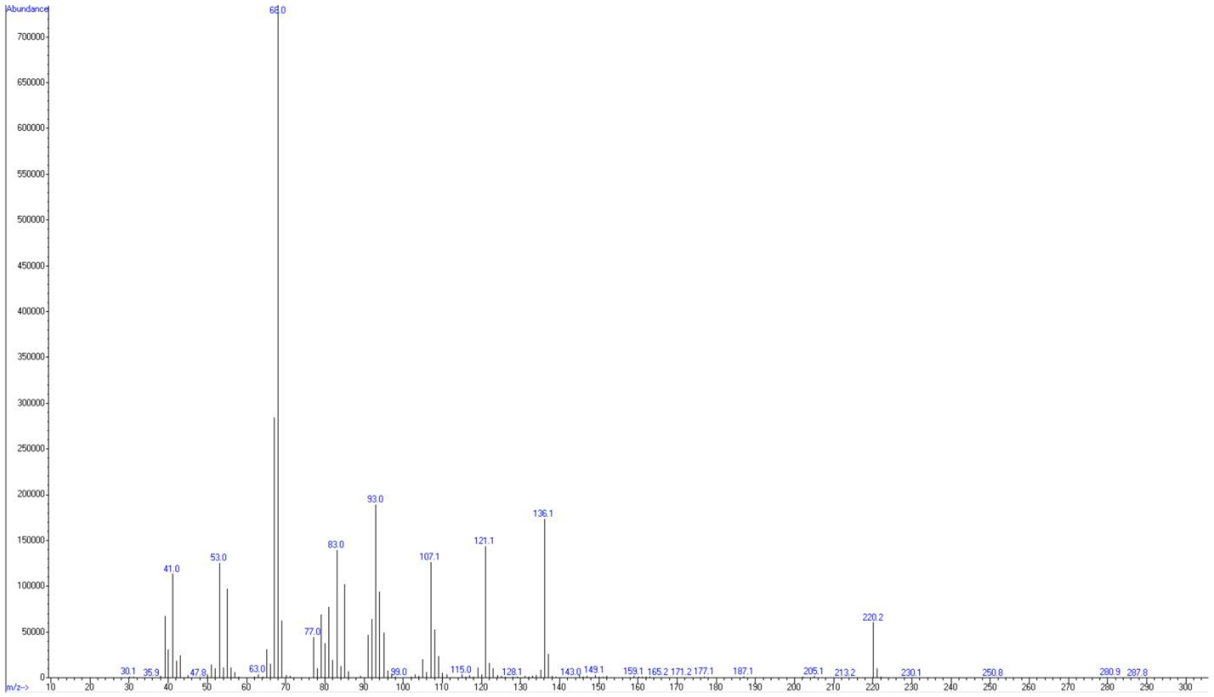

Figure S **23**. MS-data of compound **17**.

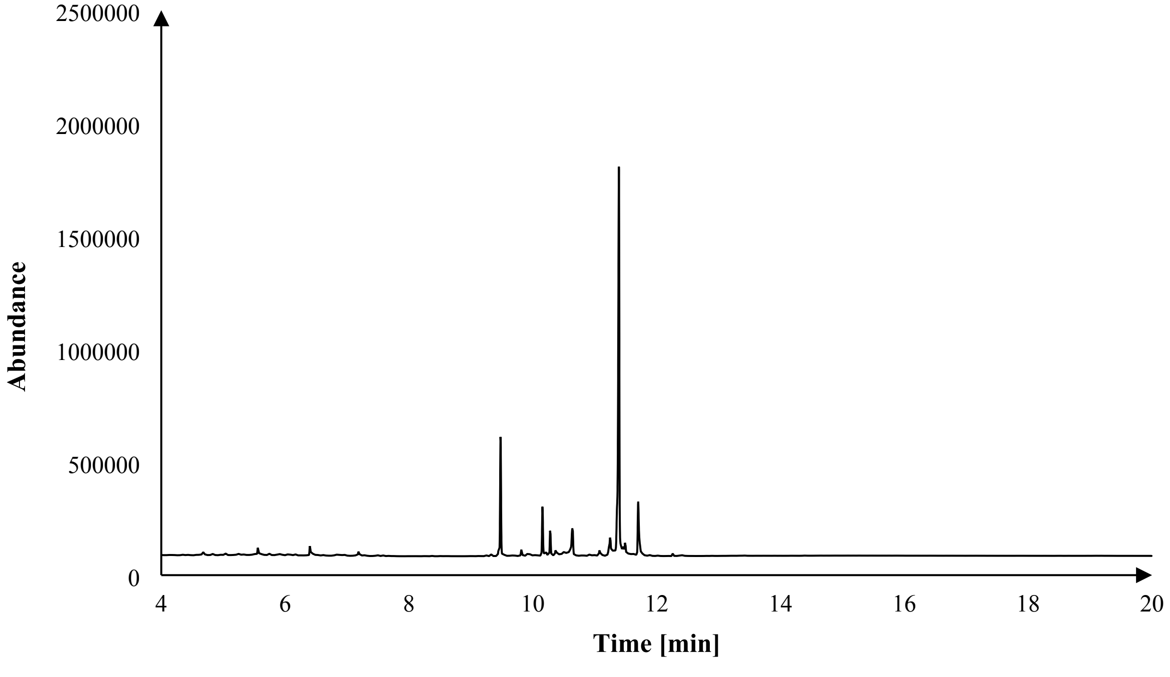

Figure S **24**. Chromatogram of the STC Omp7 and FPP derivative **9**.

1.4.4 Biotransformations using FPP Derivative 3

For FPP derivative **3** a screening with different STCs was already performed in the past, therefore only the biotransformation using PvHVS will be shown here.[6a]

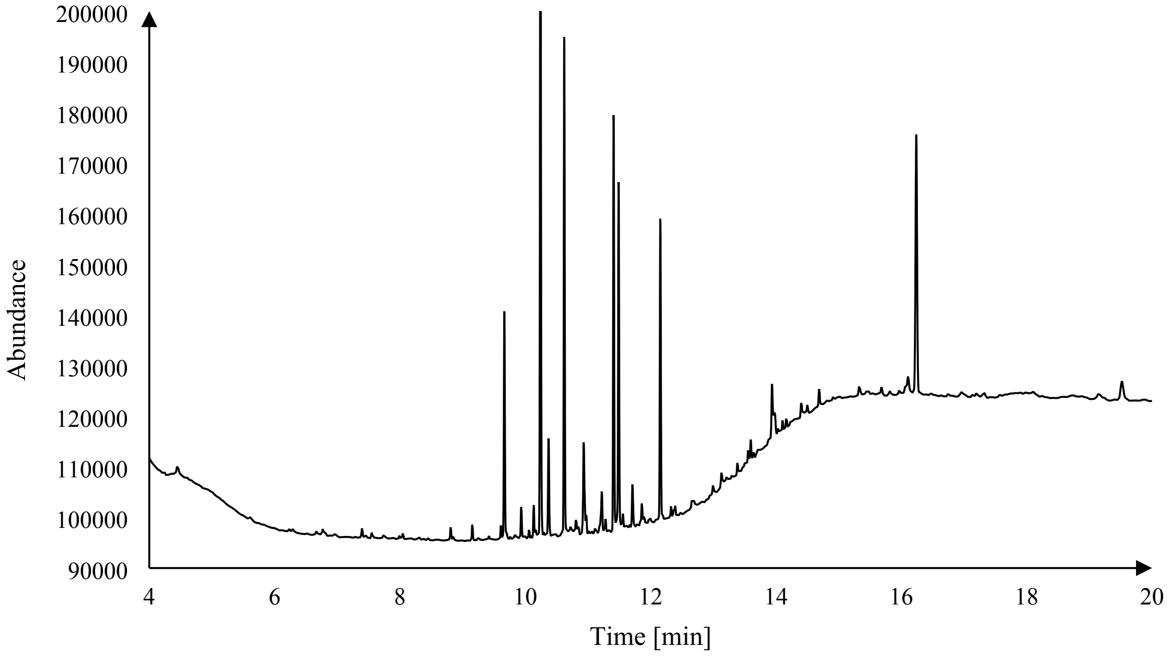

Figure S **25**. Chromatogram of PvHVS and FPP derivative **3**.

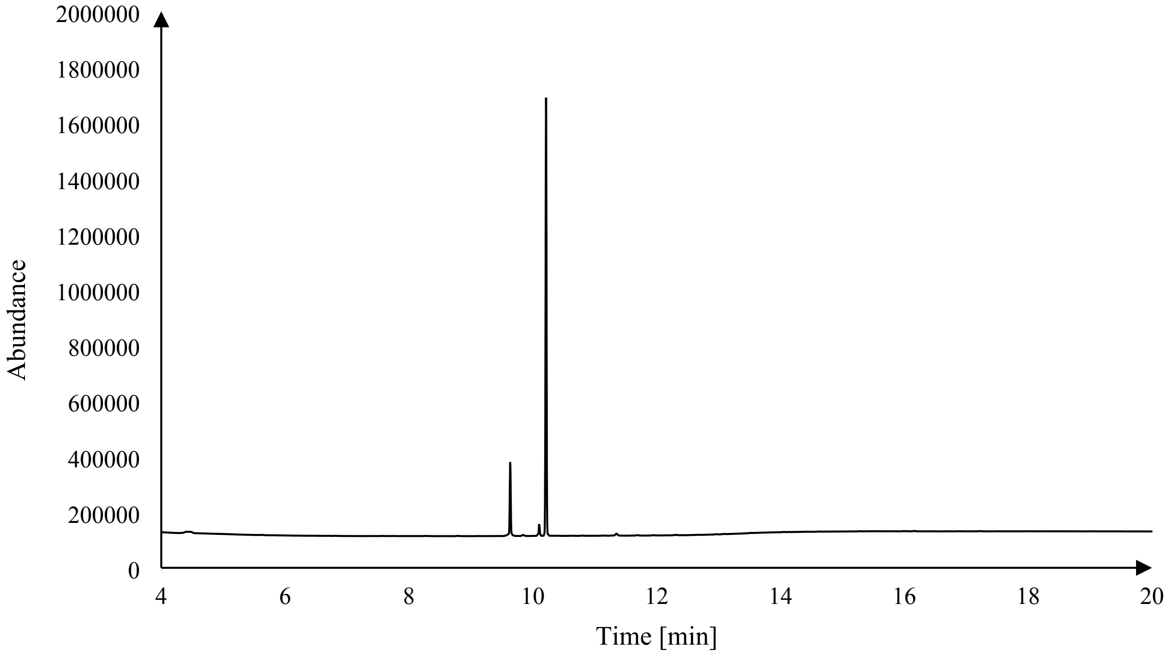

Figure S **26**. Chromatogram of isolated compound **15**.

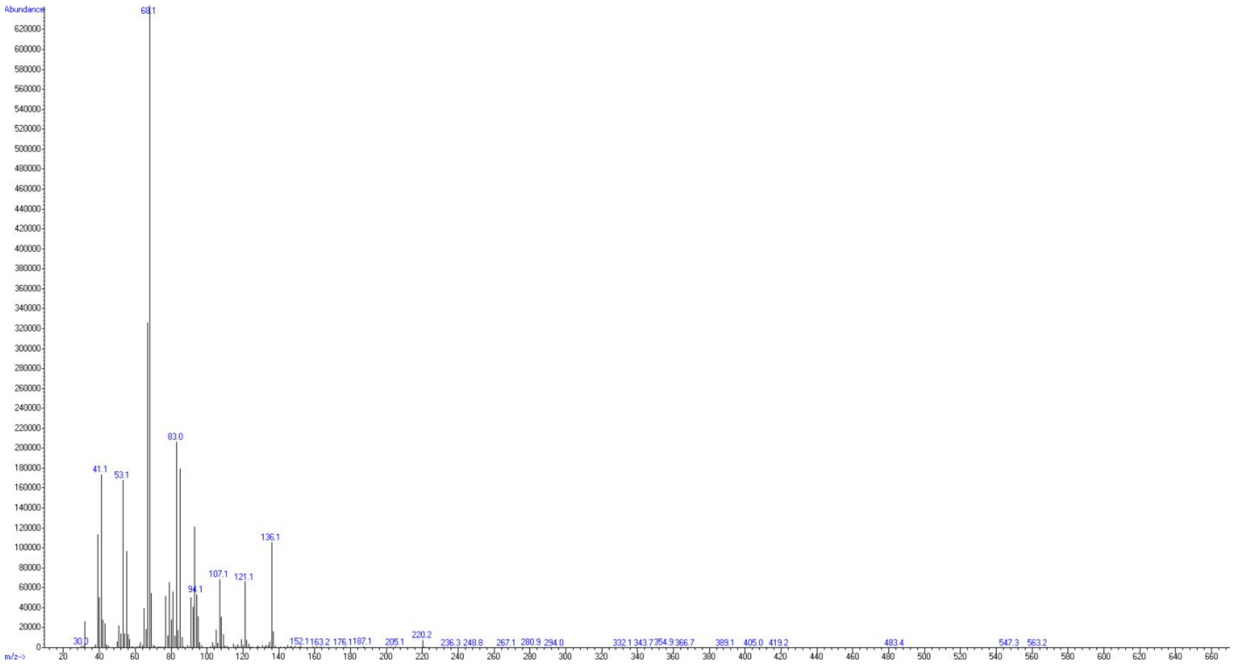

Figure S **27**. MS-data of compound **15**.

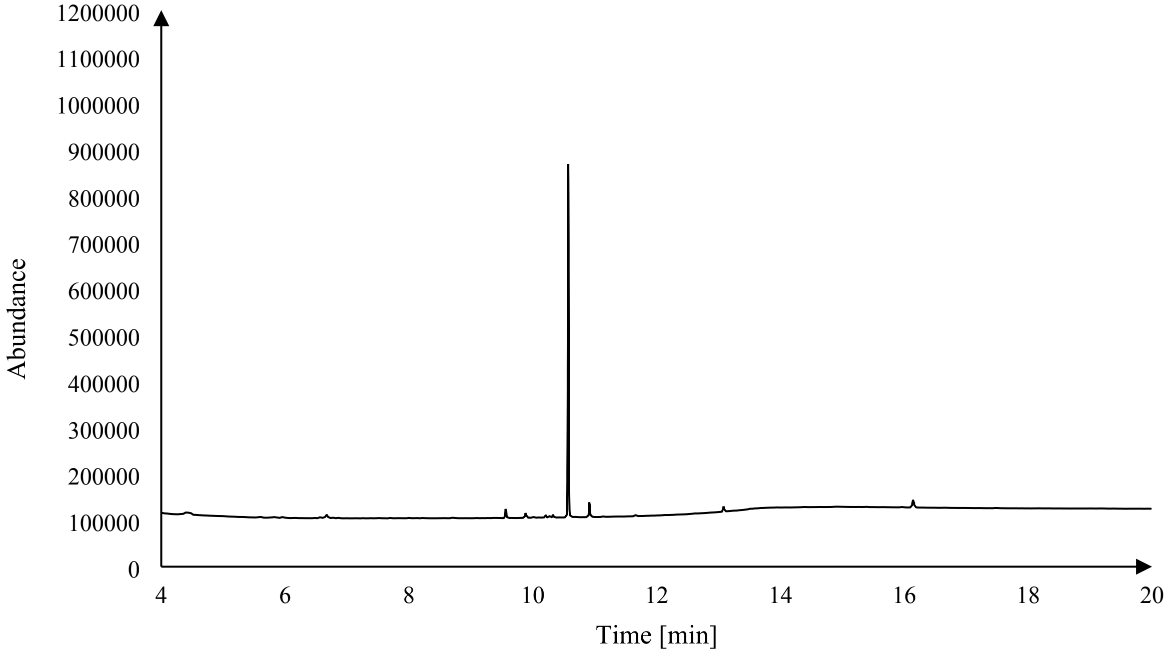

Figure S **28**. Chromatogram of isolated compound **6**.

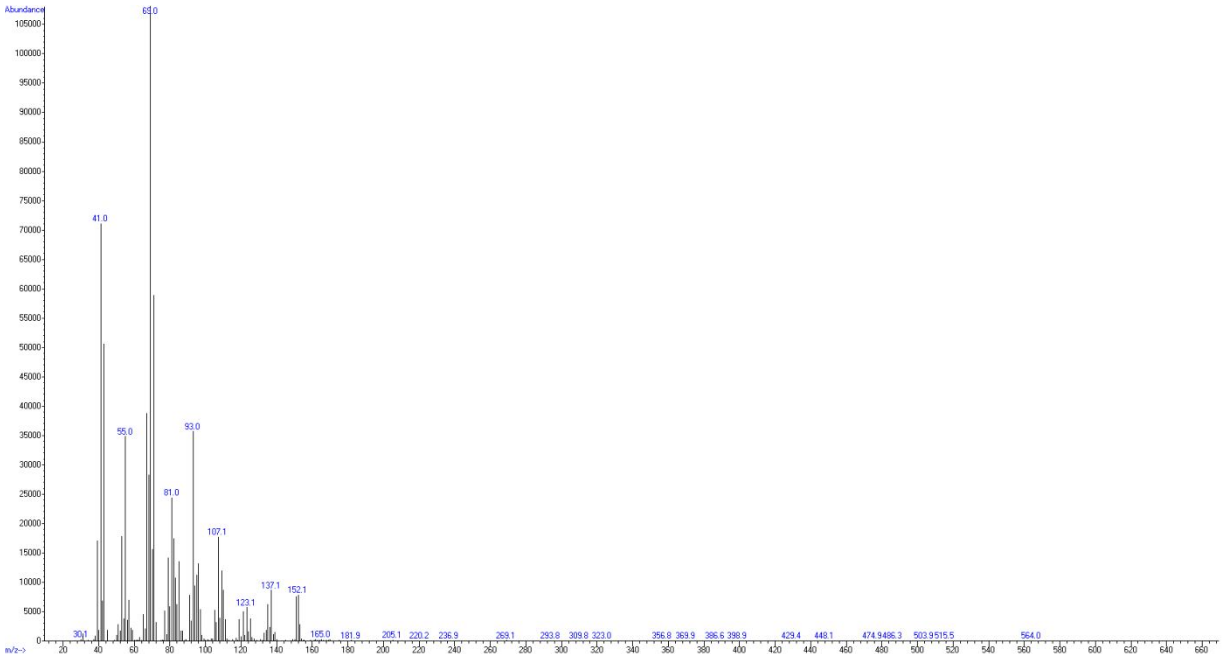

Figure S **29**. MS-data of compound **6**.

1.4.5 Biotransformations using BcBot2 mutants and 10

In a biotransformation using the Y211S mutant and FPP derivative **10** a shift towards **20** was observed.

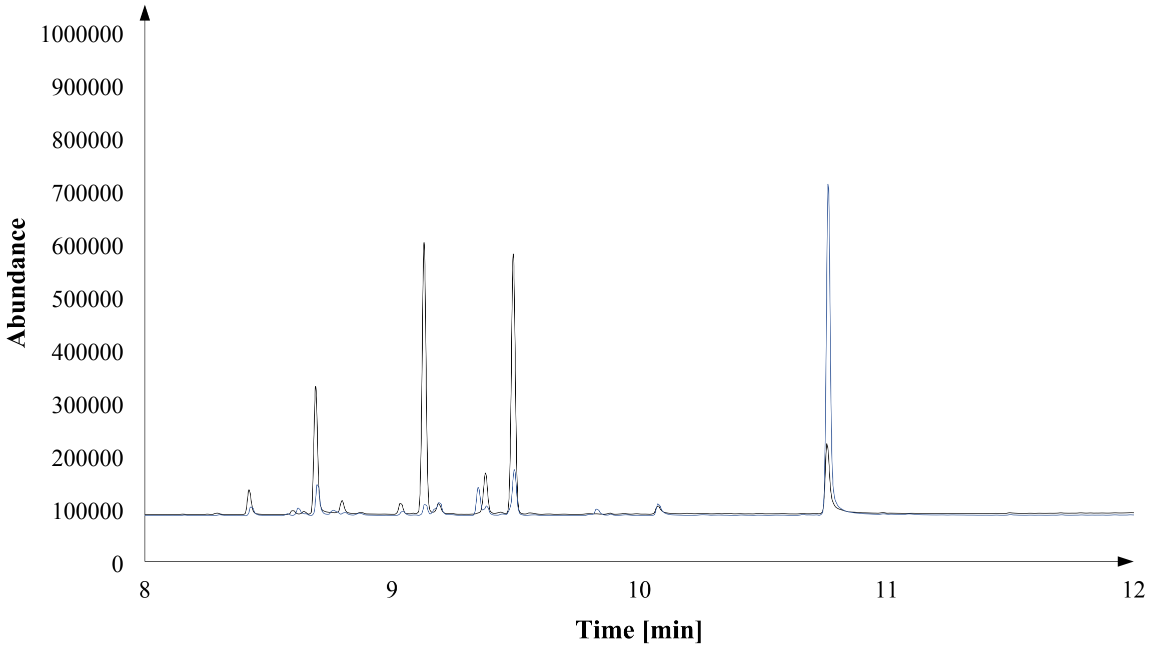

Figure S **30**. Chromatograms of biotransformations using **10** and BcBot2 WT (**black**) or Y211S mutant (**blue**).

In a biotransformation using the W118Q mutant and FPP derivative **10** a shift towards **21** was observed.

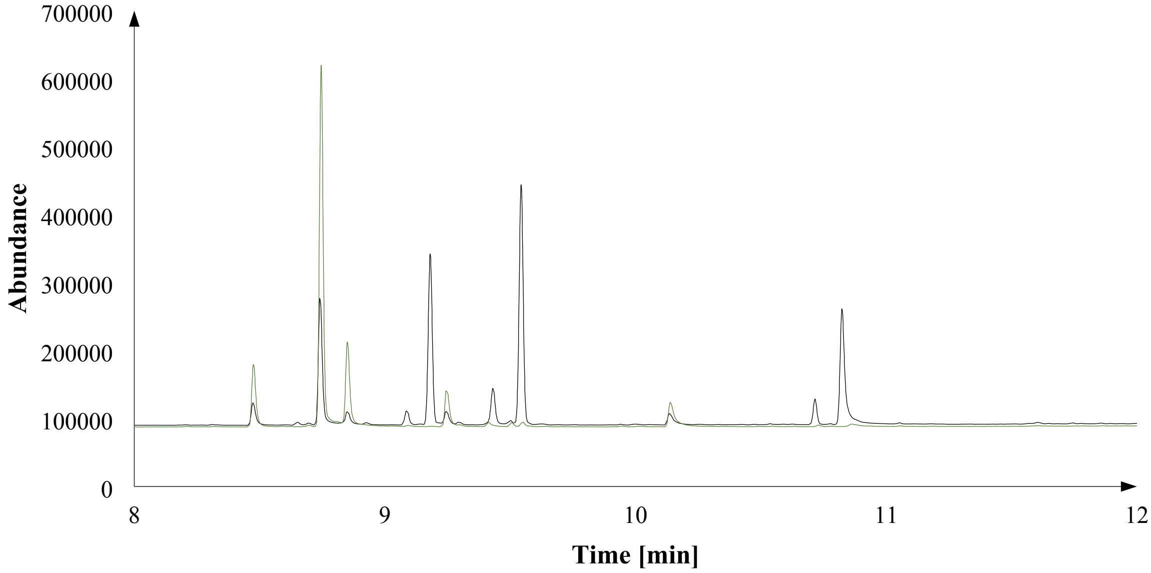

Figure S **31**. Chromatograms of biotransformations using **10** and BcBot2 WT (**black**) or W118Q mutant (**green**).

In a biotransformation using the F138V variant the formation of a former minor side product, which could not be isolated and elucidated, was now favoured. Once the isolation was performed using the F138V mutant, the new side product was elucidated as **29**.

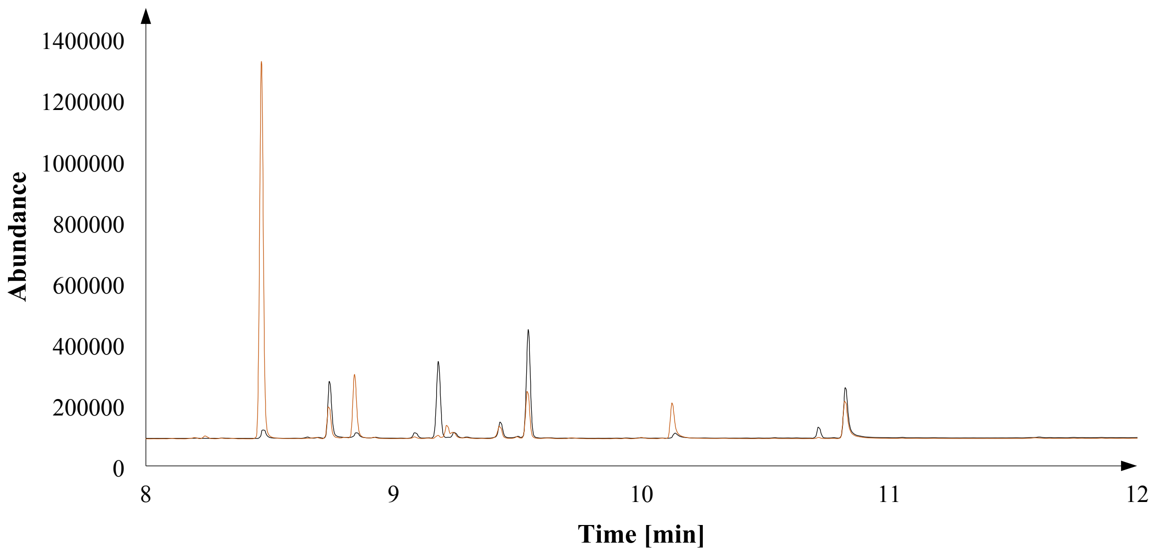

Figure S **32**. Chromatograms of biotransformations using **10** and BcBot2 WT (**black**) or F138V mutant (**orange**).

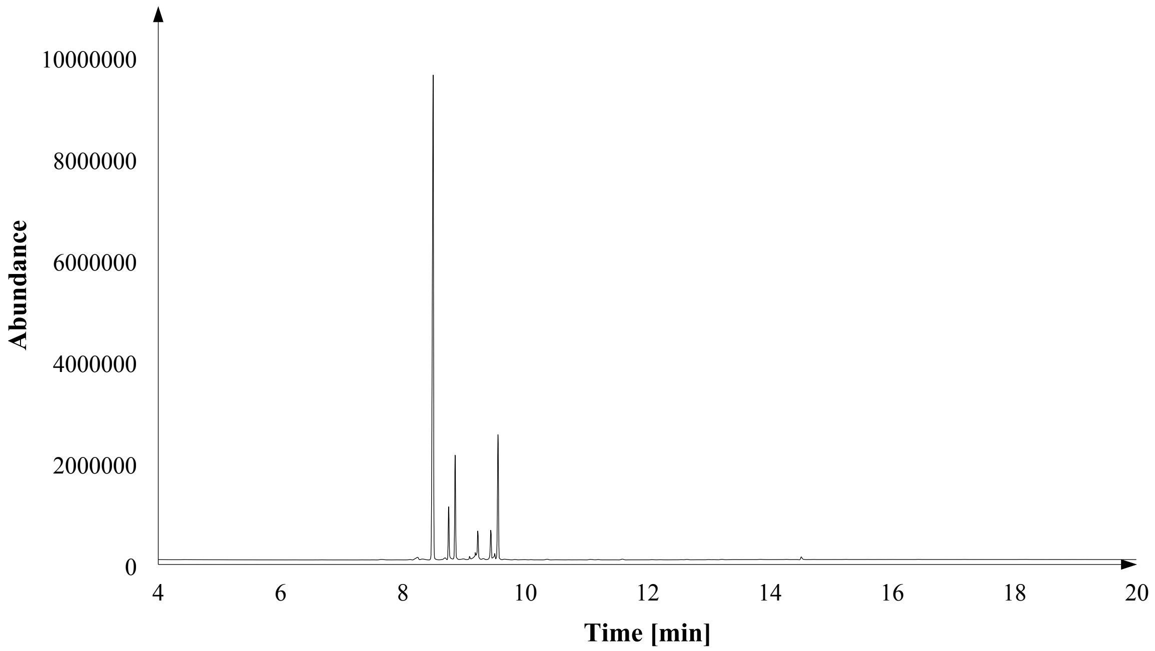

Figure S **33**. Chromatograms of non-seperable biotransformation products after silica coloumn using F138V mutant and FPP derivative **10**.

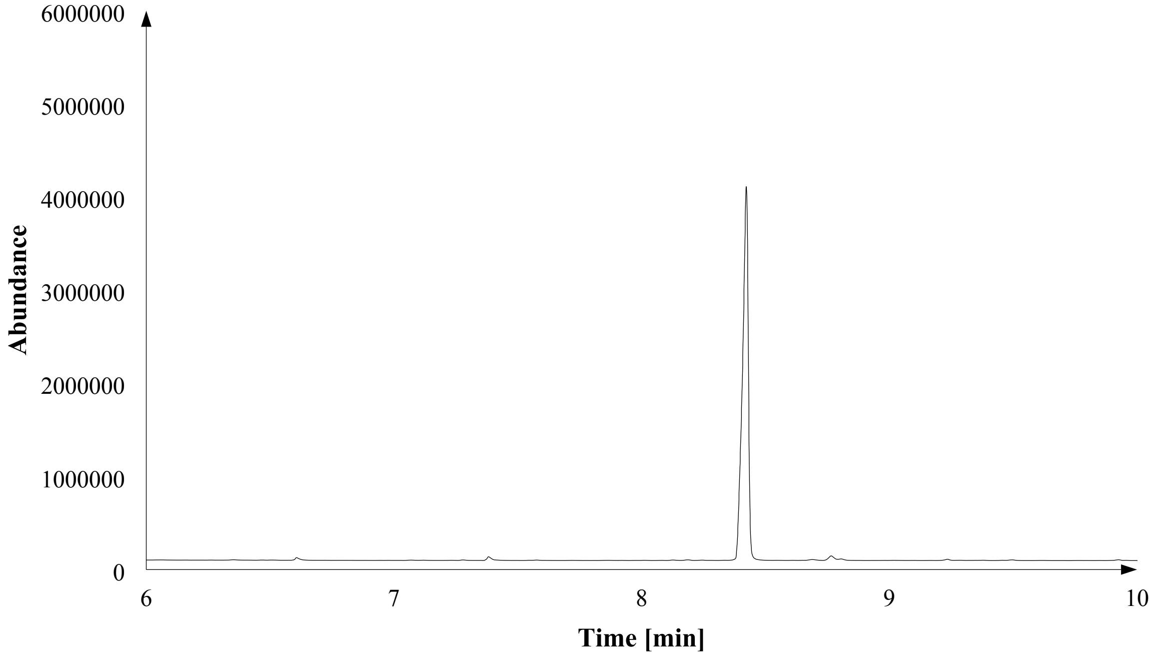

Figure S **34**. Zoom-In of desired product chromatogram after preparative GC.

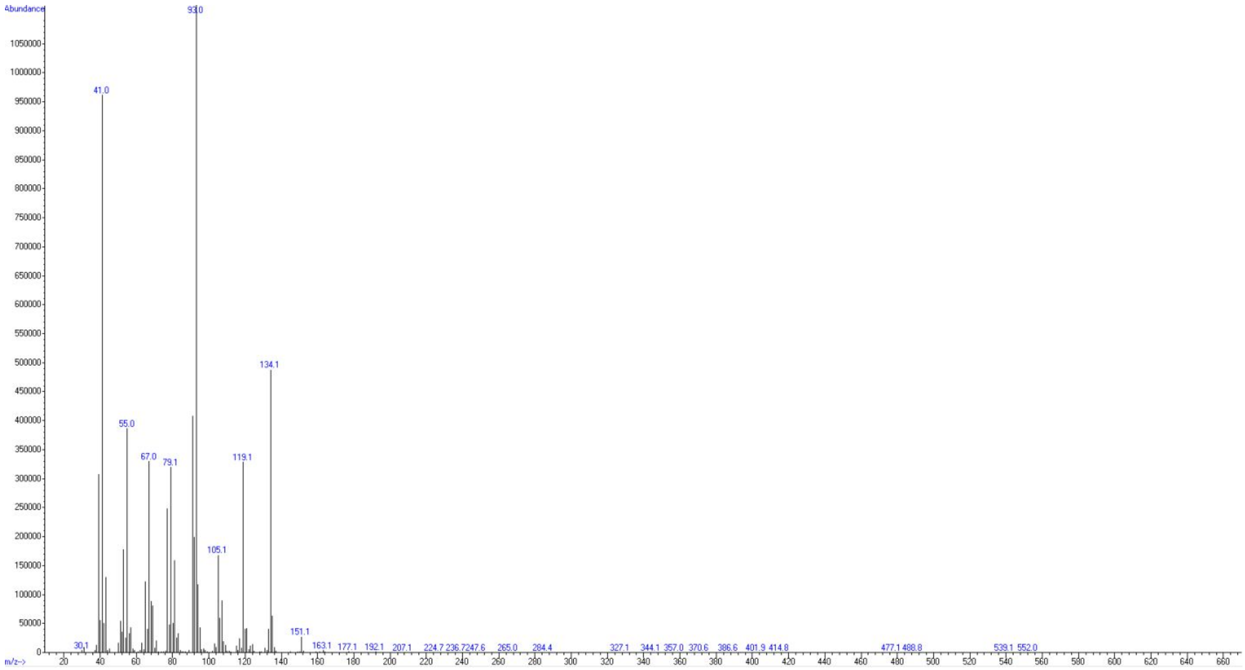

Figure S **35**. MS-data of compound **29**.

1.4.6 Retention indices of isolated products

Table S **1**. Retention index of isolated products.

Biotransformation product	Retention index
**6**	1692
**15**	1626
**16**	1890
**17**	1586
**18**	1471
**19**	1531
**20**	1757
**21**	1401
**22**	1578
**23**	1484
**29**	1358

1.5Structure elucidations

1.5.1 Structure elucidation of 18

Copies of ^{1}H-NMR and ^{13}C spectra of all biotransformation products are found in section 2 (Copies of NMR spectra).

Table S **2**. ^{1}H NMR signals and their corresponding ^{13}C NMR signals for compound **18** as analysed with the support of ^{1}H-^{13}C HSQC and ^{13}C{^{1}H} DEPT135 experiments. The quaternary carbon atoms are listed at the bottom.

δ (^{1}H)/ppm	δ (^{13}C)/ppm	DEPT135/HSQC phase
5.50	130.7	CH/CH_3
4.77	112.2	CH_2
4.12	78.8	CH_2
3.58 – 3.56	78.8 + 87.6	CH_2 + CH/CH_3
2.34 – 2.27	26.6	CH_2
2.14 – 2.03	58.0 + 34.8	CH/CH_3 + CH_2
1.97 – 1.86	35.7 + 36.3 + 26.6	CH_2 + CH_2 + CH_2
1.80 – 1.74	35.7	CH_2
1.67	16.7	CH/CH_3
1.66 – 1.61	24.8	CH_2
1.55 – 1.51	24.8	CH_2
1.32 – 1.28	36.3	CH_2
	155.7	C_q
	134.5	C_q

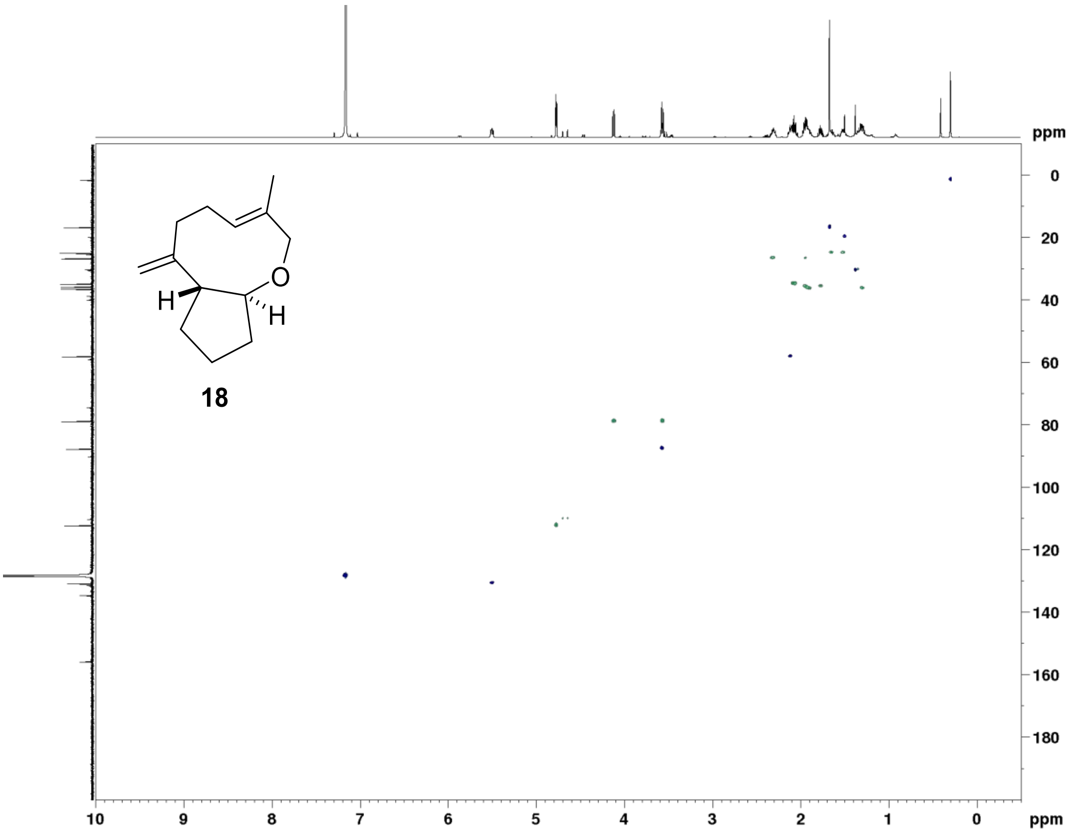

Figure S **36.** ^{1}H-^{13}C HSQC NMR spectrum of compound **18** in C_6D_6 (pos. phase = blue (CH/CH_3), neg. phase = green (CH_2)).

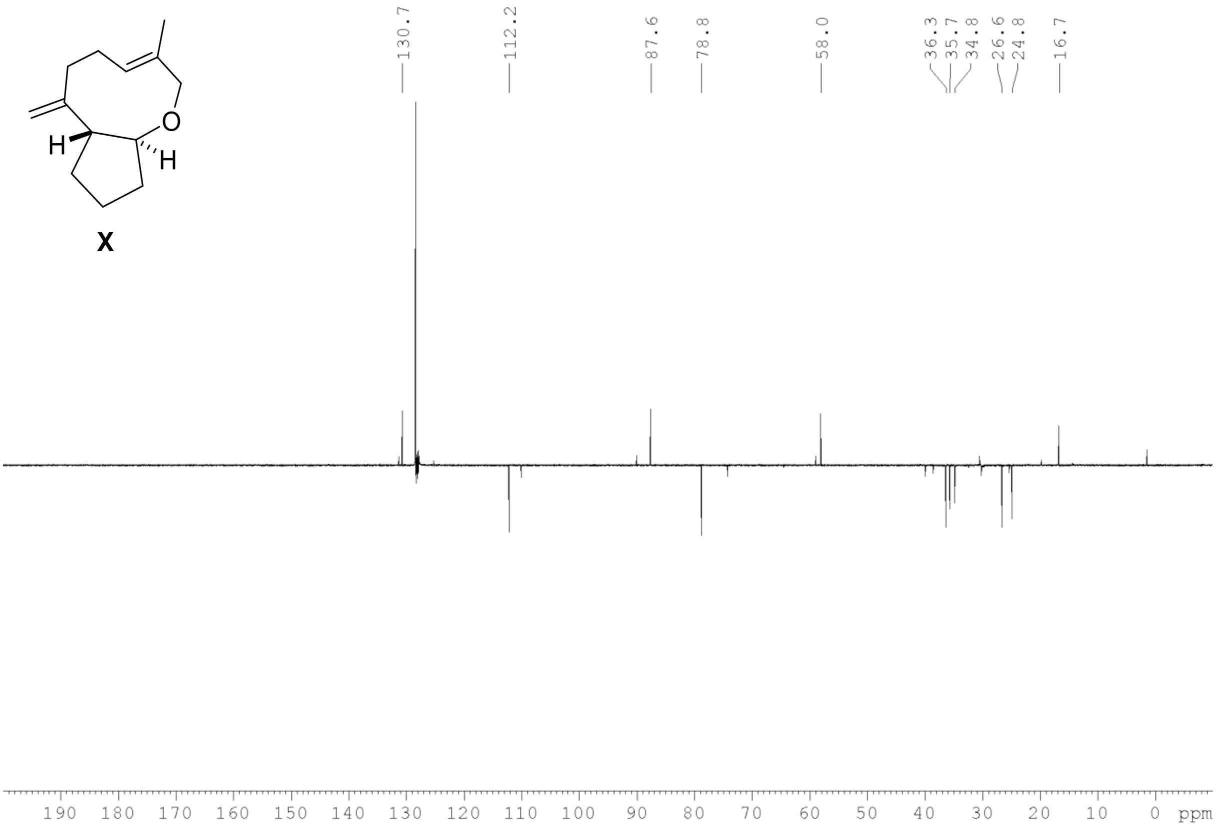

Figure S **37.** $^{13}C\{^{1}H\}$ DEPT135 NMR spectrum of compound **18** in C_6D_6.

Table S **3**. ^{1}H NMR signals and the corresponding ^{1}H-^{1}H COSY correlations for compound **18**. Signals with weak intensities are given in parentheses.

δ (^{1}H)/ppm	COSY correlations
5.50	2.34 – 2.27 + 1.97 – 1.86, 1.67
4.77	-
4.12	3.58 – 3.56
3.58 – 3.56	1.67, 1.80 – 1.74, 1.97 – 1.86, (2.14 – 2.03), 4.12
2.34 – 2.27	5.50, 2.14 – 2.03, 1.97 – 1.86
2.14 – 2.03	3.58 – 3.56, (2.34 – 2.27), 1.97 – 1.86
1.97 – 1.86	5.50, 3.58 – 3.56, 2.34 – 2.27, 2.14 – 2.03, 1.80 – 1.74, 1.32 – 1.28
1.80 – 1.74	1.97 – 1.86, 3.58 – 3.56,
1.67	5.50, 3.58 – 3.56
1.66 – 1.61	1.97 – 1.86, 1.80 – 1.74, 1.55 – 1.51, 1.32 – 1.28
1.55 – 1.51	1.97 – 1.86, (1.80 – 1.74), 1.66 – 1.61, (1.32 – 1.28)
1.32 – 1.28	1.55 – 1.51, 1.66 – 1.61, 1.97 – 1.86, 2.14 – 2.03

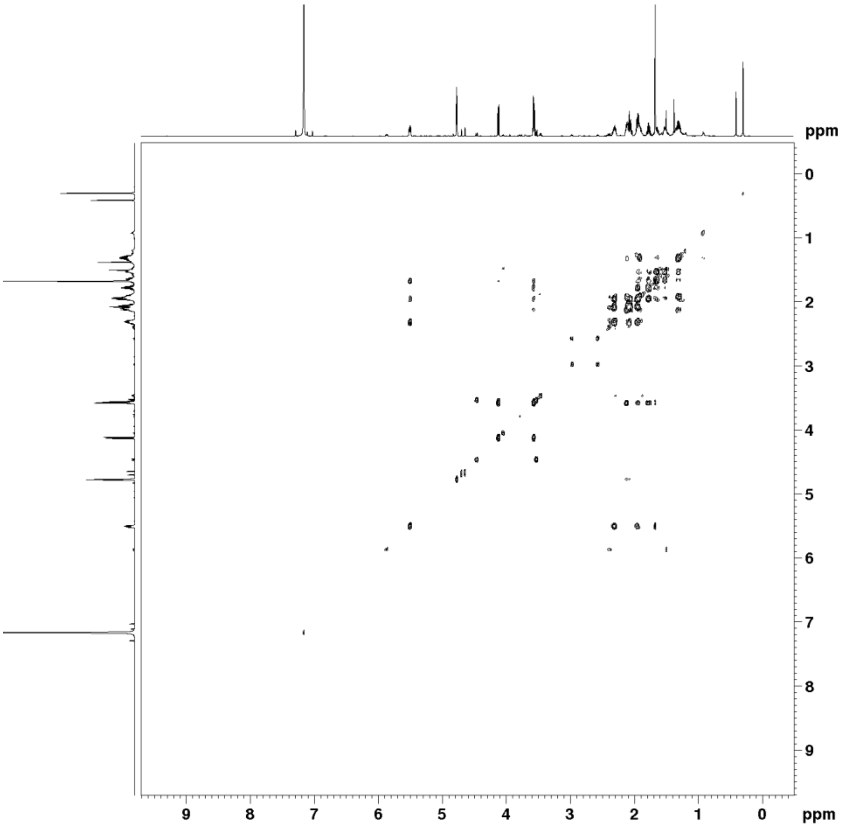

Figure S **38.** ^{1}H-^{1}H COSY NMR spectrum of compound **18** in C_6D_6.

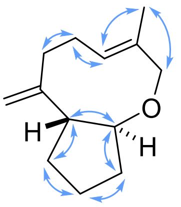

18

Figure S **39**. Key ^{1}H-^{1}H COSY NMR correlations of **18** as indicated by arrows.

Table S **4**. Selected correlations between ^{13}C NMR signals and neighbouring ^{1}H NMR signals as collected from the ^{1}H-^{13}C HMBC spectrum of compound **18**. Note: Signals with weak intensities are given in parentheses.

δ (^{1}H)/ppm	*δ* (^{13}C)/ppm
5.50	78.8, 26.6, 16.7 (34.8)
4.77	155.7, 58.0, 34.8, (26.6)
4.12	134.5, 130.7, 87.6, 16.7
3.58 – 3.56	130.7, 87.6, 16.7
2.34 – 2.27	-
2.14 – 2.03	155.7, 130.7, 112.2, 58.0, 26.6
1.97 – 1.86	134.5, 130.7, 34.8
1.80 – 1.74	(58.0), (36.3), (24.8)
1.67	134.5, 130.7, 78.8
1.66 – 1.61	-
1.55 – 1.51	-
1.32 – 1.28	155.7, 58.0, (24.8)

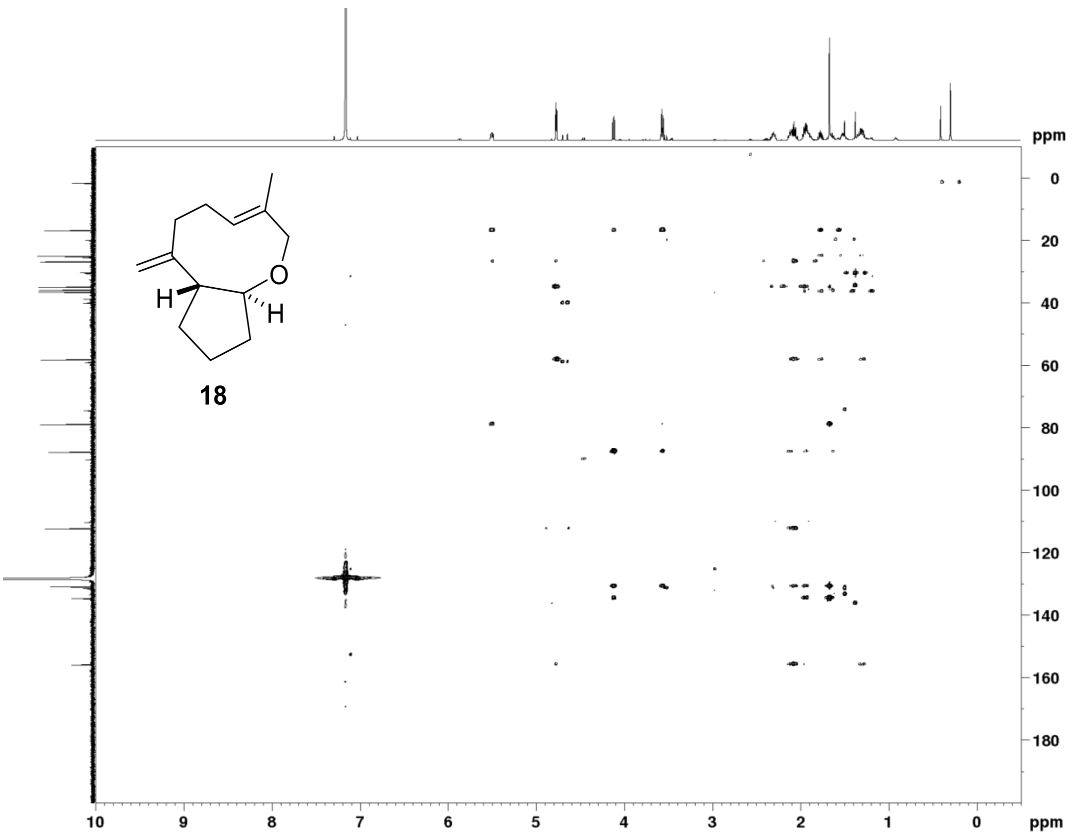

Figure S **40.** ¹H-¹³C HMBC NMR spectrum of compound **18** in C_6D_6.

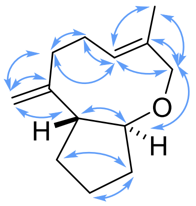

18

Figure S **41.** Key ¹H-¹³C HMBC NMR correlations of **18** as indicated by arrows.

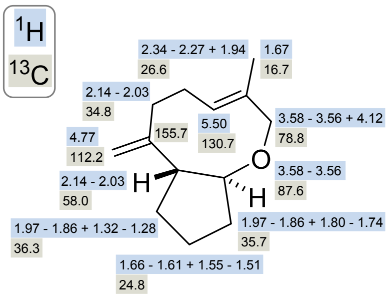

18

Since no NOESY correlation can be observed between proton signals 3.58 – 3.56 ppm and 2.14 – 2.03 ppm, the bridgehead protons will likely be *anti* orientated.

Figure S **42.** Full assignment of NMR spectra for **18**.

1.5.2 Structure elucidation of 21

Table S **5**. ^{1}H NMR signals and their corresponding ^{13}C NMR signals for compound **21** as analyzed with the support of ^{1}H-^{13}C HSQC and $^{13}C\{^{1}H\}$ DEPT135 experiments. The quaternary carbon atoms are listed at the bottom.

δ (^{1}H)/ppm	δ (^{13}C)/ppm	DEPT135/HSQC phase
6.82	133.9	CH/CH_3
5.91 – 5.82	135.8	CH/CH_3
5.42	125.7	CH/CH_3
5.33	129.2	CH/CH_3
5.27 – 5.24	115.9	CH_2
5.20 – 5.17	113.9	CH_2
5.06	113.9	CH_2
5.04	115.9	CH_2
3.78	70.6	CH_2
3.75	76.1	CH_2
2.85	26.4	CH_2
1.76	19.9	CH/CH_3
1.62	14.0	CH/CH_3
	133.2	C_q
	132.8	C_q

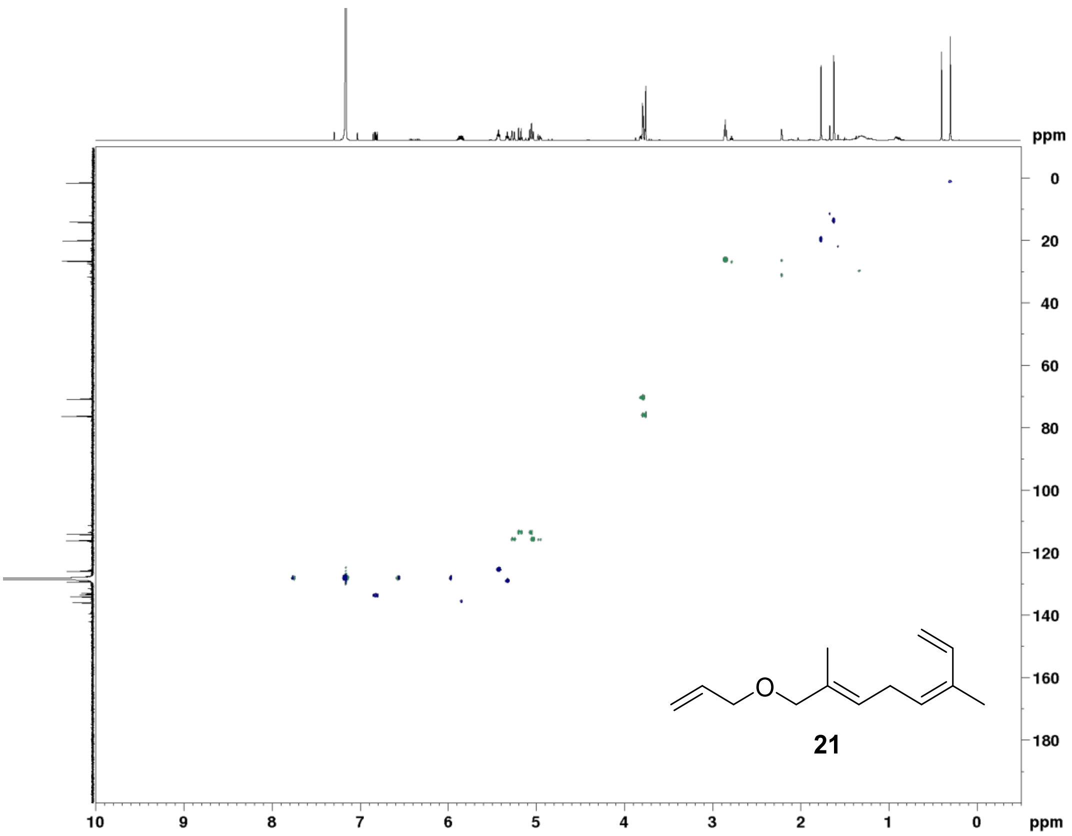

Figure S **43.** ^{1}H-^{13}C HSQC NMR spectrum of compound **21** in C_6D_6 (pos. phase = blue (CH/CH_3), neg. phase = green (CH_2)).

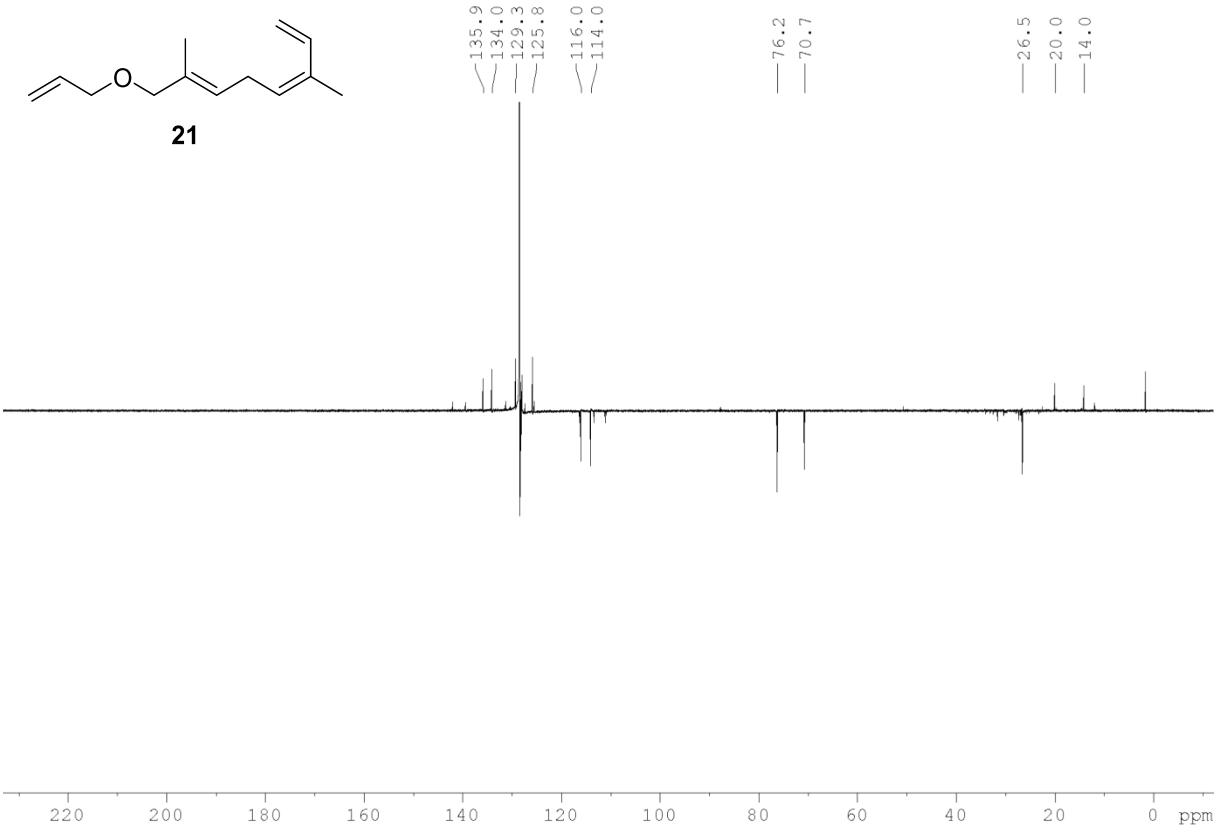

Figure S **44.** ^{13}C{^{1}H} DEPT135 NMR spectrum of compound **21** in C_6D_6.

Table S **6.** ^{1}H NMR signals and the corresponding ^{1}H-^{1}H COSY correlations for compound **21**.

δ (^{1}H)/ppm	COSY correlations
6.82	5.20 – 5.17, 5.06
5.91 – 5.82	5.27 – 5.24, 5.04, 3.78,
5.42	3.75, 2.85, 1.62
5.33	2.85, 1.76
5.27 – 5.24	3.78, 5.04, 5.91 – 5.28
5.20 – 5.17	6.82, 5.06
5.06	6.82, 5.20 – 5.17
5.04	5.91 – 5.82, 5.27 – 5.24, 3.78
3.78	5.91 – 5.82, 5.27 – 5.24, 5.04
3.75	5.42, 2.85, 1.62
2.85	5.42, 5.33, 3.75, 1.76, 1.62
1.76	5.33, 2.85
1.62	5.42, 3.75, 2.85

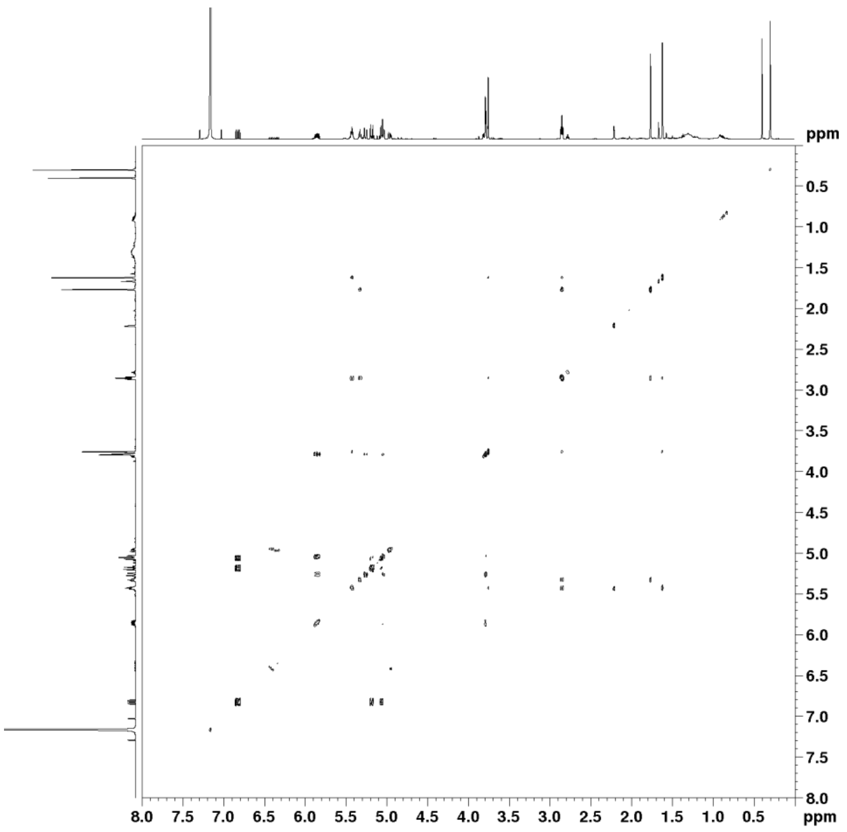

Figure S **45**. ^{1}H-^{1}H COSY NMR spectrum of compound **21** in C_6D_6.

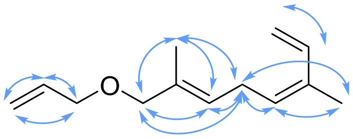

21

Figure S **46**. Key ^{1}H-^{1}H COSY NMR correlations of **21** as indicated by arrows.

Table S **7**. Selected correlations between ^{13}C NMR signals and neighbouring ^{1}H NMR signals as collected from the ^{1}H-^{13}C HMBC spectrum of compound **21**.

δ (^{1}H)/ppm	δ (^{13}C)/ppm
6.82	132.8, 129.2, 19.9
5.91 – 5.82	70.6
5.42	129.2, 76.1, 26.4, 14.0
5.33	133.9, 125.7, 26.4, 19.9
5.27 – 5.24	70.6
5.20 – 5.17	132.8
5.06	132.8
5.04	70.6
3.78	135.8, 115.9, 76.1
3.75	133.2, 125.7, 70.6, 14.0
2.85	133.2, 129.2, 125.7
1.76	132.8, 129.2
1.62	133.2, 125.7, 76.1

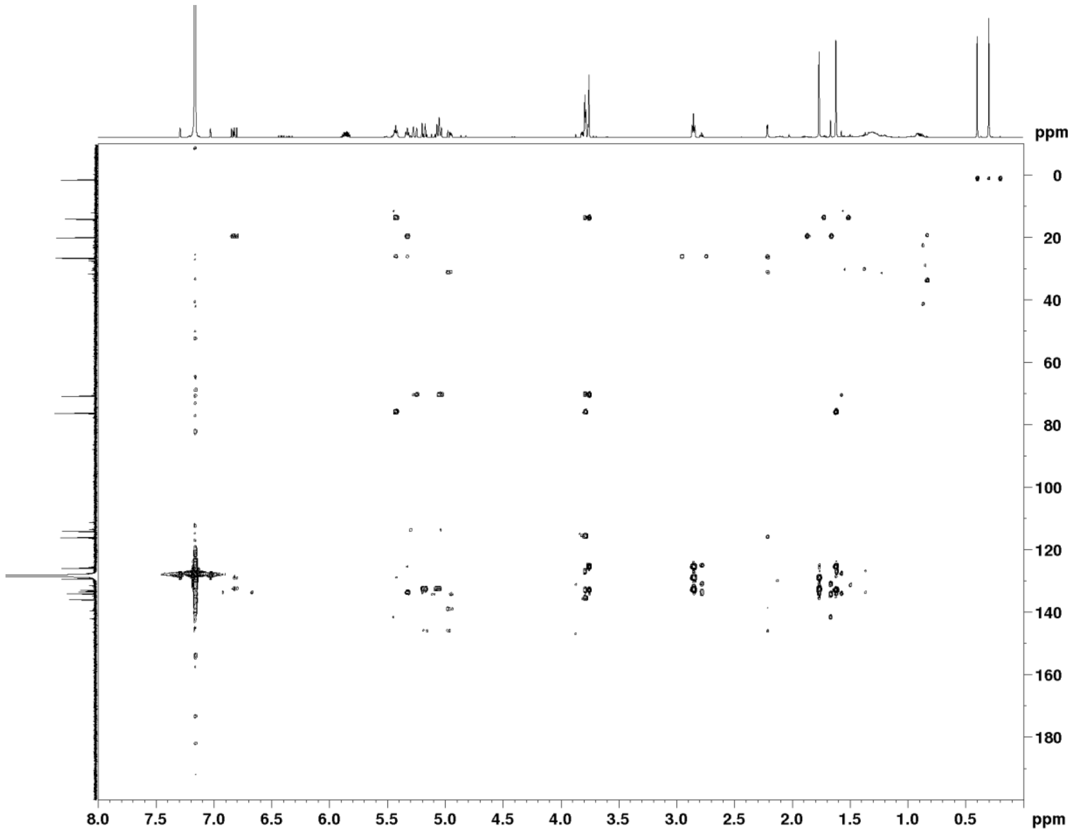

Figure S **47**. ^{1}H-^{13}C HMBC NMR spectrum of compound **21** in C_6D_6.

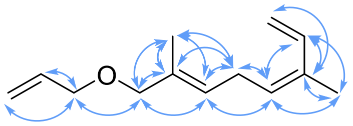

21

Figure S **48**. Key ^{1}H-^{13}C HMBC NMR correlations of **21** as indicated by arrows.

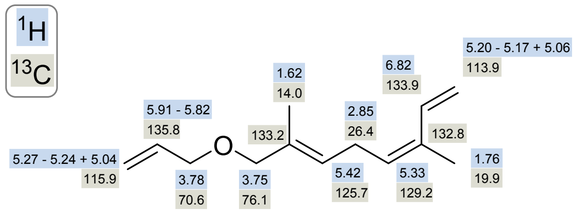

21

The NOESY spectra of **21** shows a correlation of 5.33 and 1.76, as well as a correlation between 6.82 and 2.85. We therefore believe that the main product carries a *Z* configurated double bond, formed in the deprotonation process.

Figure S **49**. Full assignment of NMR spectra for **21**.

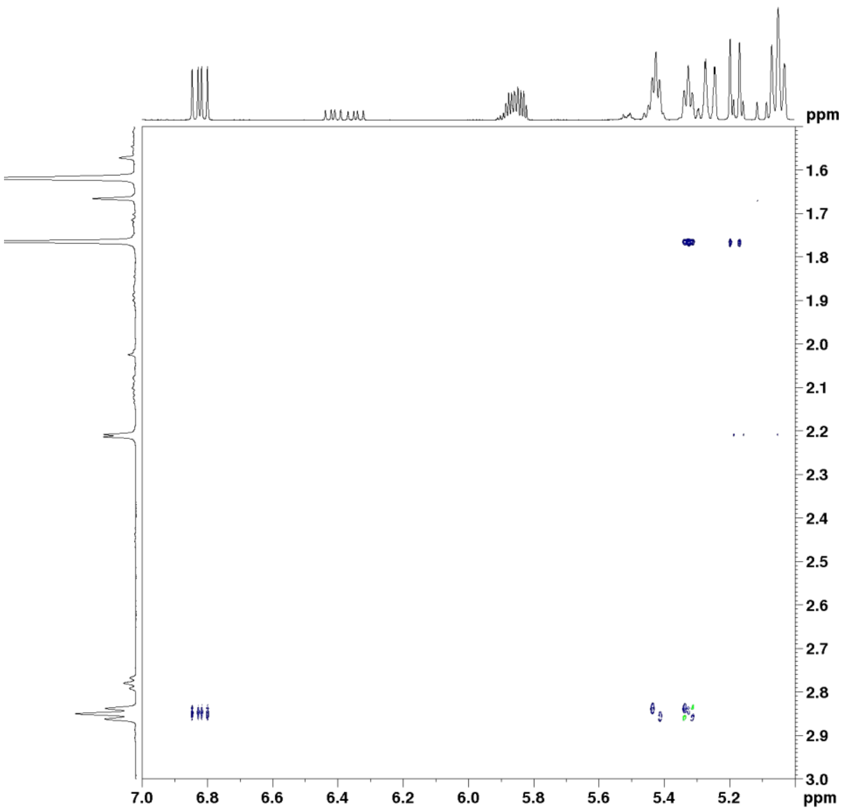

Figure S **50**. NOESY NMR spectrum of compound **21** in C_6D_6.

Based on a rough NMR analysis of the two more abundant side products, these could be double bond isomers showing an *E* isomer (C_3=C_4, as indicated by the signal at 2.78 ppm) as well as a 1,1-disubstituted double bond (C_3=C_{12}). Since the biotransformation using the BcBot2 F138V mutant allowed isolation of one side product, we identified one to be the compound with a 1,1-disubstituted double bond (see below).

1.5.3 Structure elucidation of 19

Table S **8**. ^{1}H NMR signals and their corresponding ^{13}C NMR signals for compound **19** as analysed with the support of ^{1}H-^{13}C HSQC and ^{13}C{^{1}H} DEPT135 experiments. The quaternary carbon atoms are listed at the bottom.

δ (^{1}H)/ppm	δ (^{13}C)/ppm	DEPT135/HSQC phase
5.51	147.1	CH/CH_3
4.93 – 4.90	131.2	CH/CH_3
4.75	109.7	CH/CH_3
4.69	127.2	CH/CH_3
3.87	77.7	CH_2
2.04 – 2.00	39.8, 25.1	CH_2, CH_2
1.92 – 1.85	27.9, 27.4	CH_2, CH_2
1.49	14.5	CH/CH_3
1.36	15.2	CH/CH_3
	133.8	C_q
	131.8	C_q

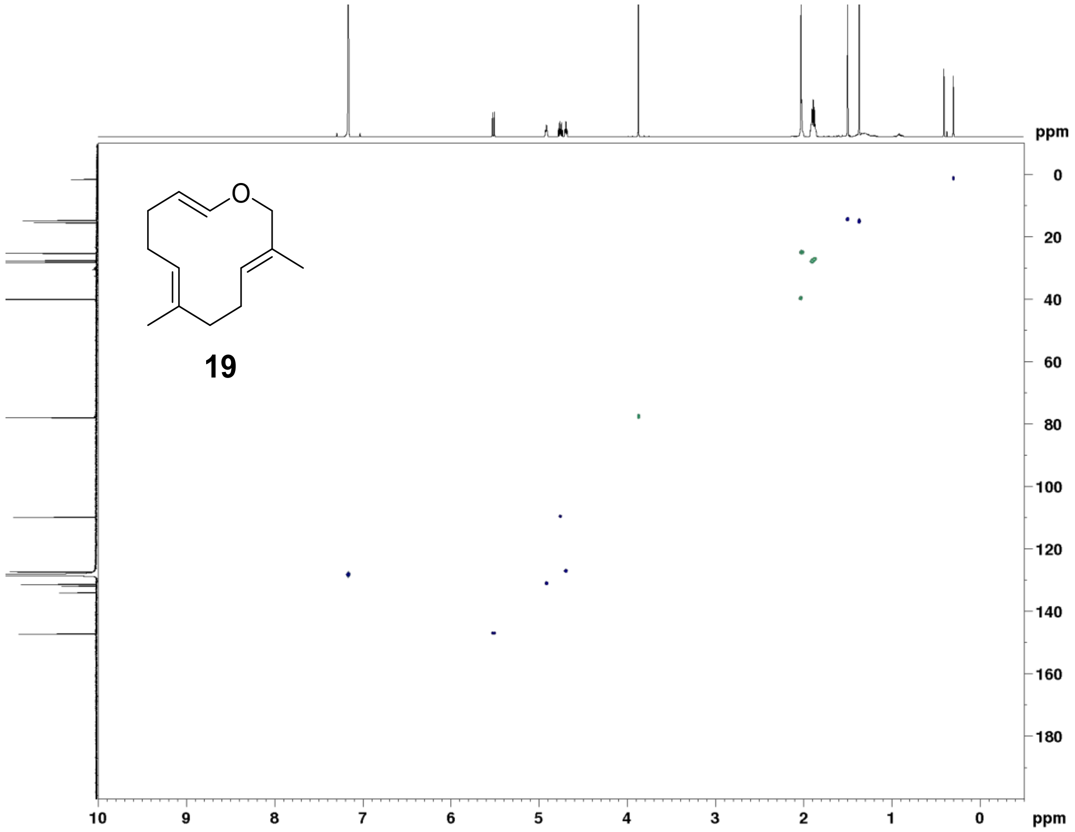

Figure S **51.** ^{1}H-^{13}C HSQC NMR spectrum of compound **19** in C_6D_6 (pos. phase = blue (CH/CH_3), neg. phase = green (CH_2)).

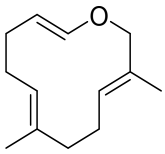

19

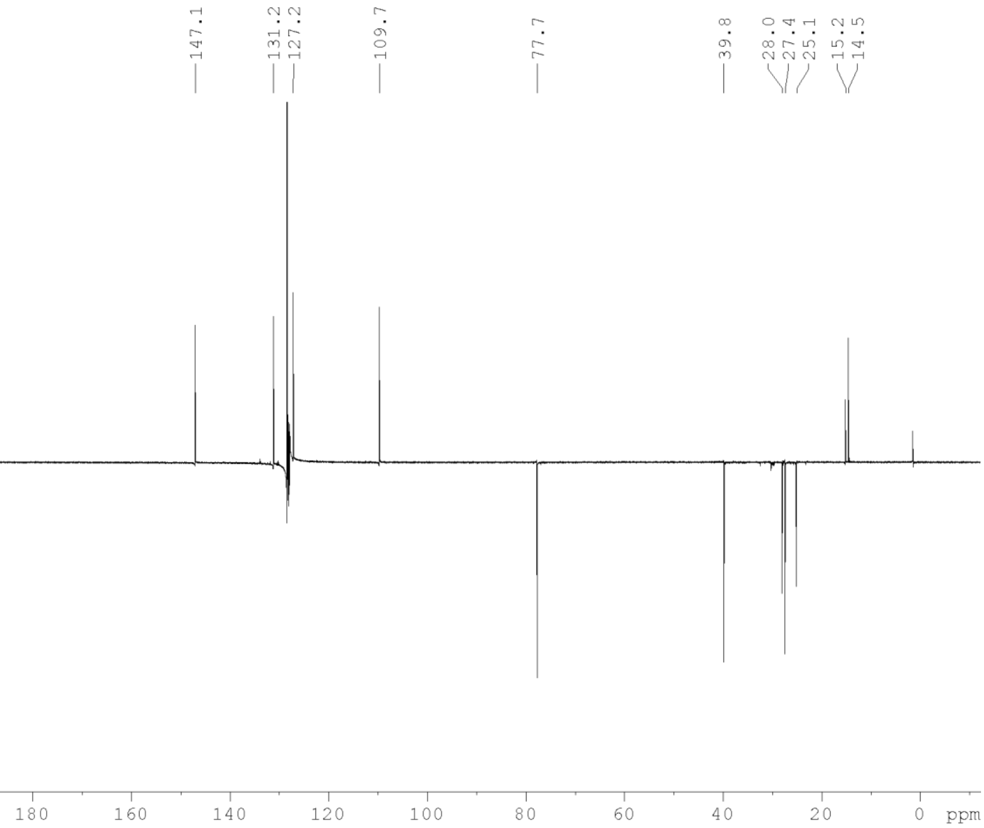

Figure S **52.** $^{13}C\{^{1}H\}$ DEPT135 NMR spectrum of compound **19** in C_6D_6.

Table S **9**. ^{1}H NMR signals and the corresponding ^{1}H-^{1}H COSY correlations for compound **19**. Signals with weak intensities are given in parentheses.

δ (^{1}H)/ppm	COSY correlations
5.51	4.75, 1.92 – 1.85
4.93 – 4.90	3.87, 2.04 – 2.00, 1.49
4.75	5.51, 1.92 – 1.85
4.69	2.04 – 2.00, 1.92 – 1.85, 1.36
3.87	4.93 – 4.90, 2.04 – 2.00
2.04 – 2.00	4.93 – 4.90, 3.87, 1.49
1.92 – 1.85	(5.51), 4.75, 4.69, (2.04 – 2.00), 1.36
1.49	4.93 – 4.90, 2.04 – 2.00
1.36	4.69, 2.04 – 2.00, 1.92 – 1.85

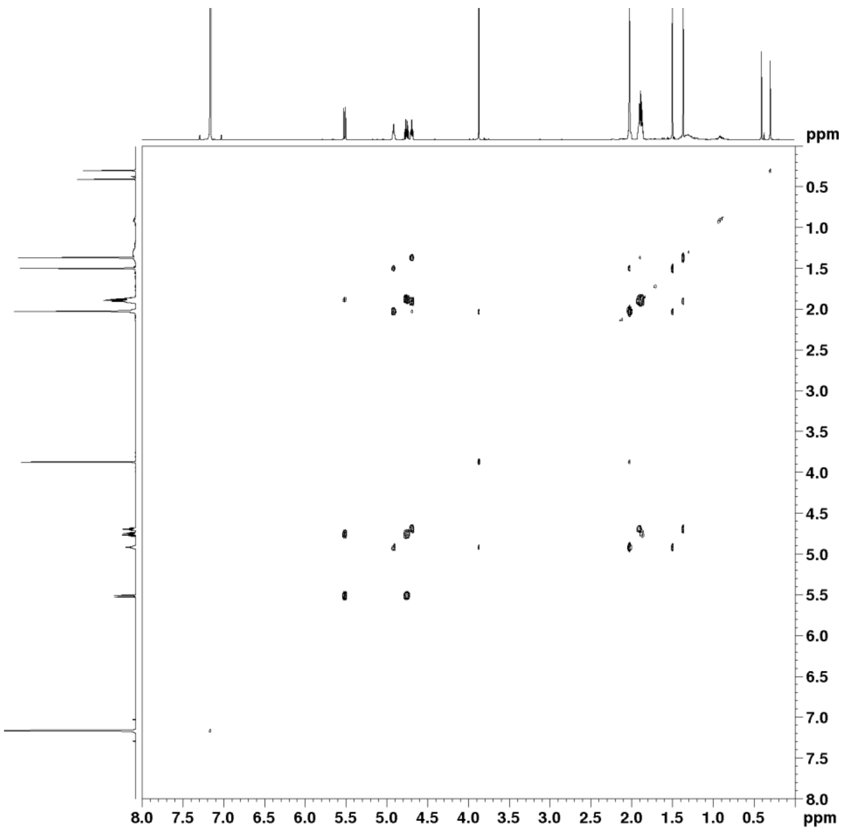

Figure S **53**. ^1H-^1H COSY NMR spectrum of compound **19** in C_6D_6.

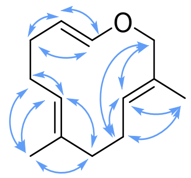

19

Figure S **54**. Key ^1H-^1H COSY NMR correlations of **19** as indicated by arrows.

Analyzing the COSY spectra, especially the long range ^5J coupling of the ether group to the allylic CH_2 group is noteworthy.

Table S **10**. Selected correlations between ^{13}C NMR signals and neighbouring ^1H NMR signals as collected from the ^1H-^{13}C HMBC spectrum of compound **19**.

δ (^1H)/ppm	δ (^{13}C)/ppm
5.51	109.7, 77.7, 27.4
4.93 – 4.90	77.7, 39.8, 25.1, 14.5
4.75	147.1, 27.4
4.69	39.8, 27.9, 15.2
3.87	147.1, 131.2, 14.5
2.04 – 2.00	133.8, 131.8, 131.2, 127.2, 39.8, 25.1, 15.2
1.92 – 1.85	147.1, 133.8, 127.2, 109.7, 27.9, 27.4
1.49	132.8, 131.2, 77.7

δ (^{1}H)/ppm	δ (^{13}C)/ppm
1.36	133.8, 127.2, 39.8

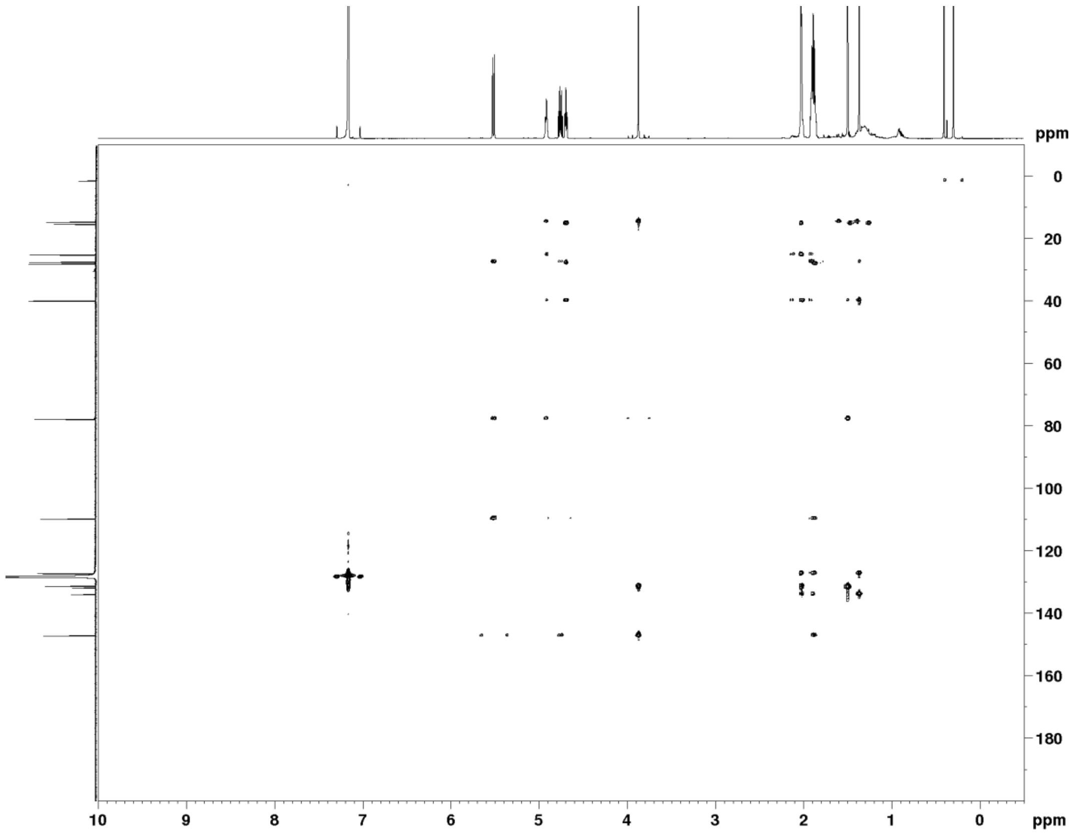

Figure S **55**. ^{1}H-^{13}C HMBC NMR spectrum of compound **19** in C_6D_6.

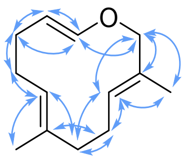

19

Figure S **56**. Key ^{1}H-^{13}C HMBC NMR correlations of **19** as indicated by arrows.

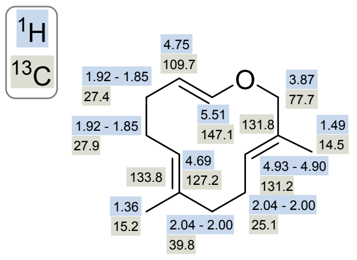

19

Since only a dispersive signal between 4.75 ppm and 5.51 ppm can be observed in the NOESY spectra, which is probably based on ^{3}J couplings, the new formed double bond is proposed to be *anti* orientated.

Figure S **57**. Full assignment of NMR spectra for **19**.

1.5.4 Structure elucidation of 20

Table S **11**. ^{1}H NMR signals and their corresponding ^{13}C NMR signals for compound **20** as analysed with the support of ^{1}H-^{13}C HSQC and ^{13}C{^{1}H} DEPT135 experiments. The quaternary carbon atoms are listed at the bottom.

δ (^{1}H)/ppm	δ (^{13}C)/ppm	DEPT135/HSQC phase
9.32	201.1	CH/CH_3
5.35	124.9	CH/CH_3
5.05 – 5.02	124.2	CH/CH_3
3.81	68.6	CH_2
2.13 – 2.09	26.3	CH_2
2.03 – 2.00	39.8	CH_2
1.86 – 1.81	43.2, 27.5	CH_2
1.56	13.7	CH/CH_3
1.48	16.0	CH/CH_3
1.40	22.4	CH_2
	135.9	C_q
	135.6	C_q

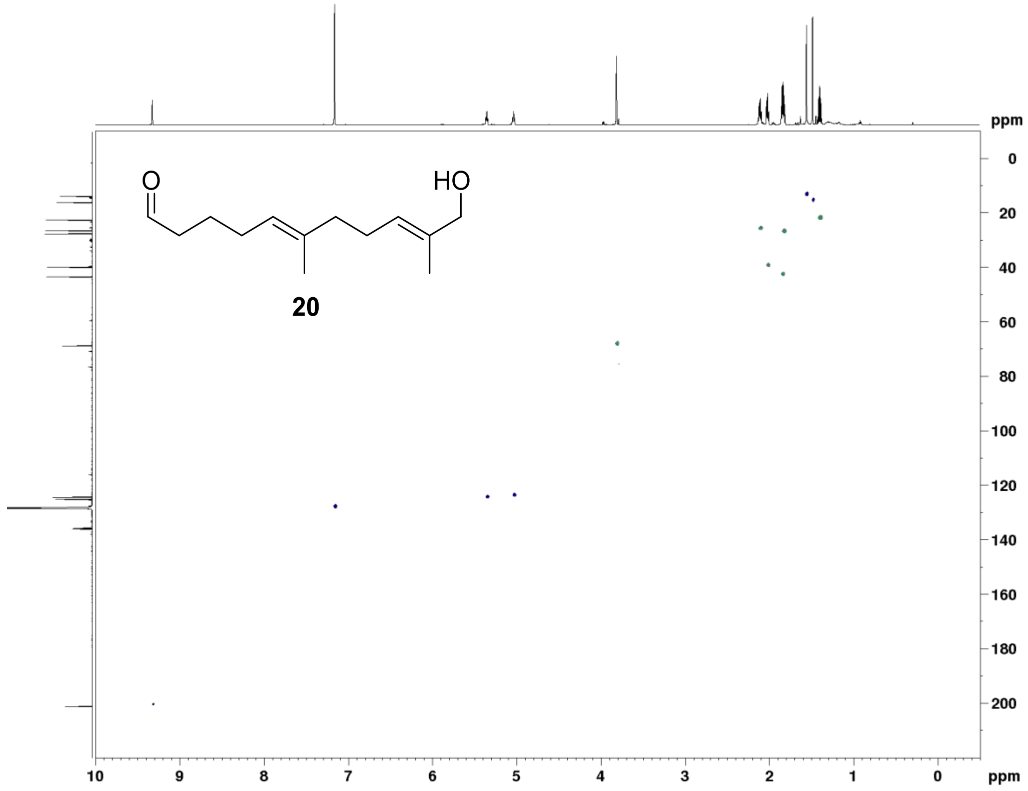

Figure S **58.** ^{1}H-^{13}C HSQC NMR spectrum of compound **20** in C_6D_6 (pos. phase = blue (CH/CH_3), neg. phase = green (CH_2)).

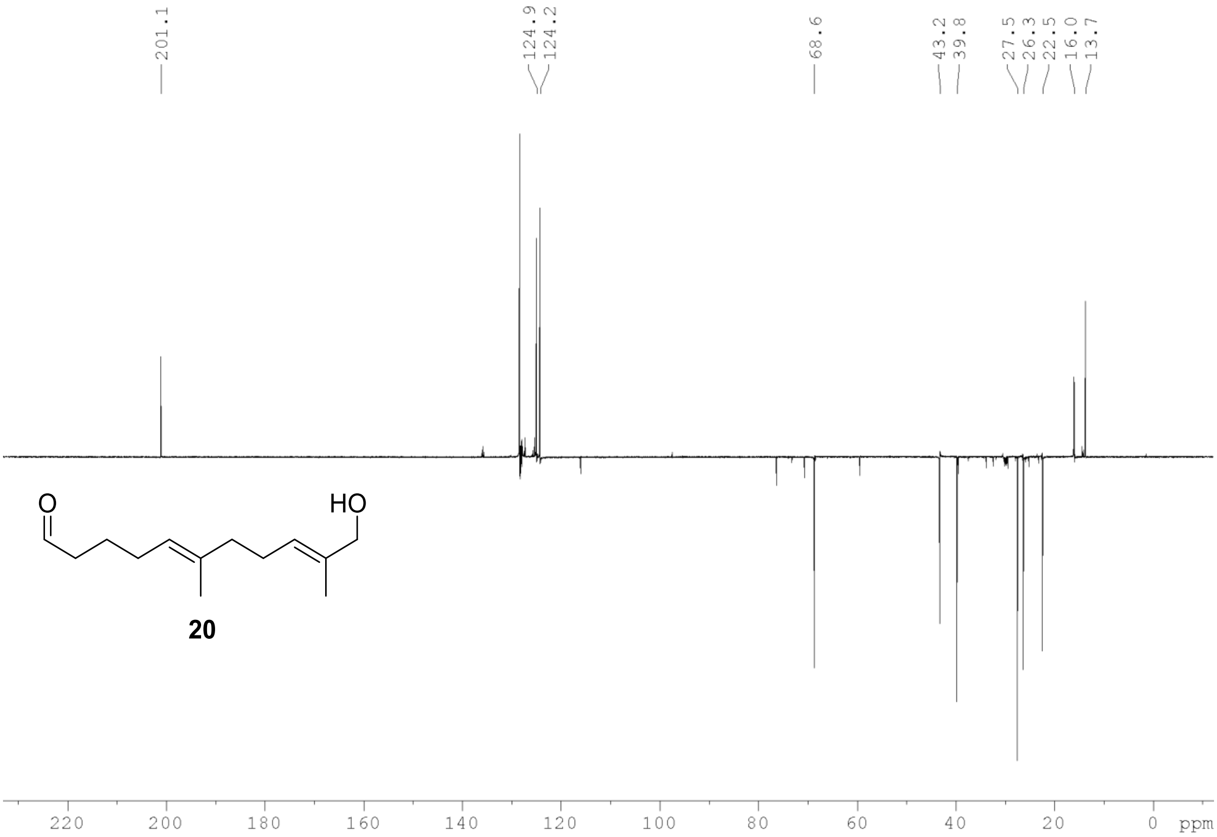

Figure S **59.** $^{13}C\{^{1}H\}$ DEPT135 NMR spectrum of compound **20** in C_6D_6.

Table S **12.** ^{1}H NMR signals and the corresponding ^{1}H-^{1}H COSY correlations for compound **20**. Signals with weak intensities are given in parentheses.

δ (^{1}H)/ppm	COSY correlations
9.32	1.86 – 1.81
5.35	3.81, 2.13 – 2.09, 1.56
5.05 – 5.02	(2.03 – 2.00), 1.86 – 1.81, 1.48
3.81	5.35, 2.13 – 2.09, 1.56
2.13 – 2.09	5.35, 3.81, 2.03 – 2.00, 1.56
2.03 – 2.00	2.13 – 2.09
1.86 – 1.81	9.32, 5.05 – 5.02, 1.40
1.56	5.35, 3.81, 2.13 – 2.09
1.48	5.05 – 5.02, 1.86 – 1.81
1.40	1.86 – 1.81

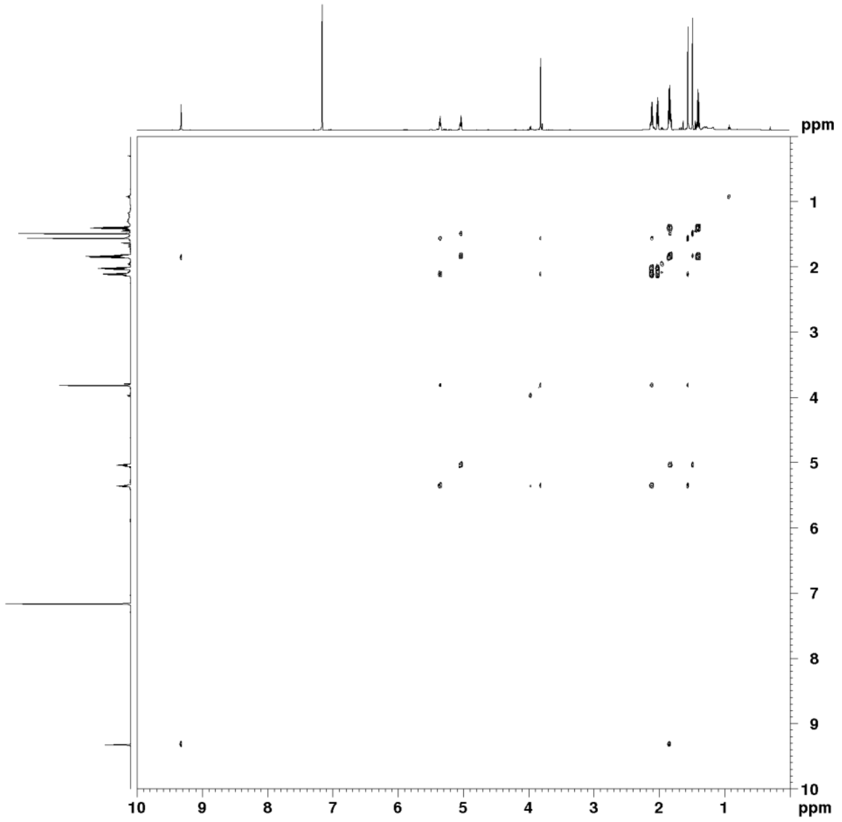

Figure S **60**. ^{1}H-^{1}H COSY NMR spectrum of compound **20** in C_6D_6.

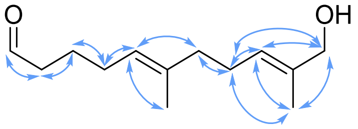

20

Figure S **61**. Key ^{1}H-^{1}H COSY NMR correlations of **20** as indicated by arrows.

Table S **13**. Selected correlations between ^{13}C NMR signals and neighbouring ^{1}H NMR signals as collected from the ^{1}H-^{13}C HMBC spectrum of compound **20**.

δ (^{1}H)/ppm	δ (^{13}C)/ppm
9.32	43.2, 22.4
5.35	68.6, 39.8, 26.3, 13.7
5.05 – 5.02	39.8, 27.5, 22.4, 16.0
3.81	135.6, 124.9, 13.7
2.13 – 2.09	135.6, 124.9, 39.8
2.03 – 2.00	135.9, 124.9, 124.2, 26.3, 16.0
1.86 – 1.81	201.1, 135.9, 124.2, 43.2, 27.5, 22.4
1.56	135.6, 124.9, 68.6
1.48	135.9, 124.2, 39.8

δ (^{1}H)/ppm	δ (^{13}C)/ppm
1.40	201.1, 124.2, 43.2, 27.5

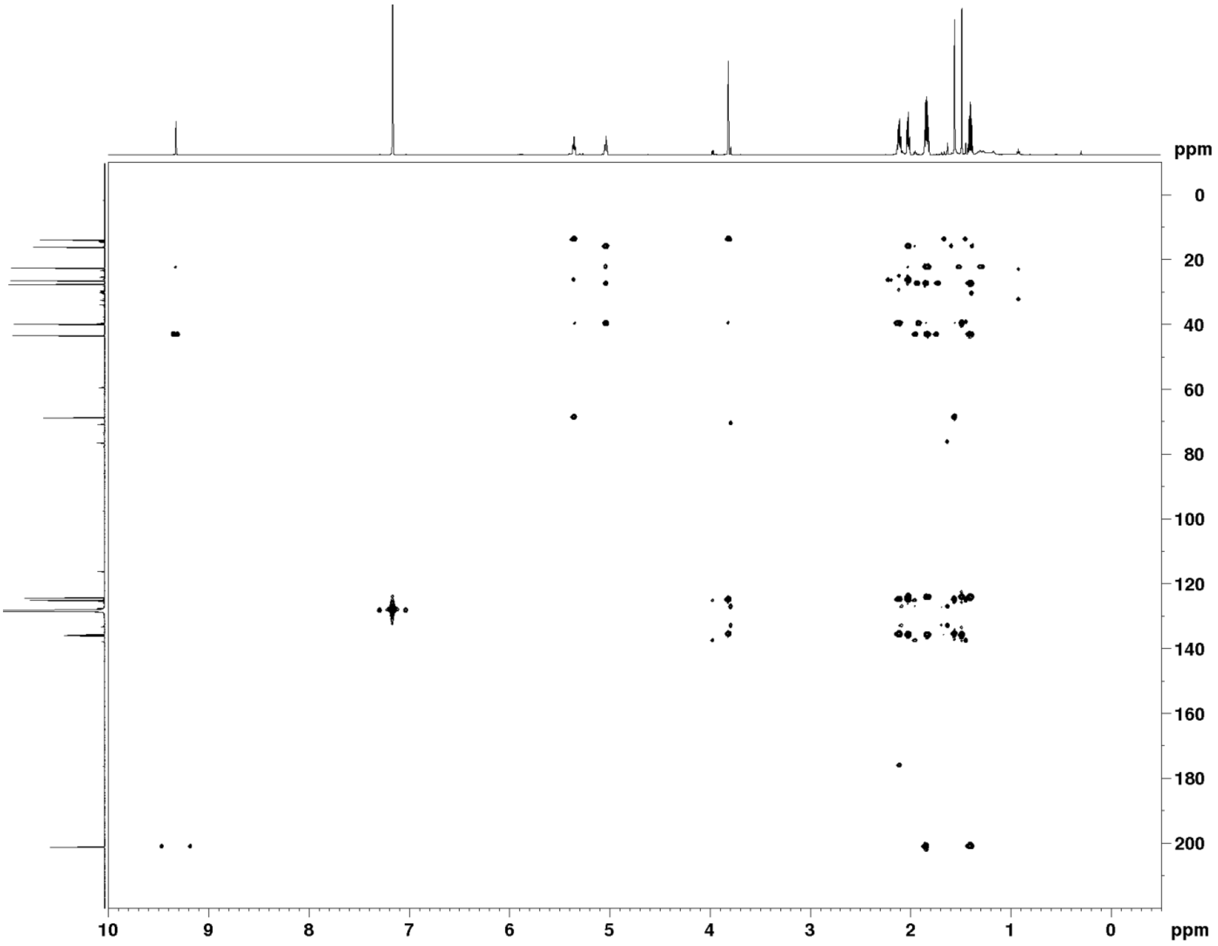

Figure S **62**. ^{1}H-^{13}C HMBC NMR spectrum of compound **20** in C_6D_6.

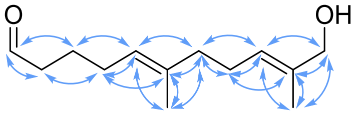

20

Figure S **63**. Key ^{1}H-^{13}C HMBC NMR correlations of **20** as indicated by arrows.

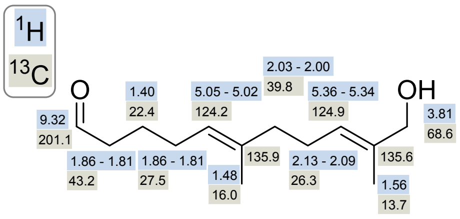

20

Figure S **64**. Full assignment of NMR spectra for **20**.

1.5.5 Structure elucidation of 23

Table S **14**. ^{1}H NMR signals and their corresponding ^{13}C NMR signals for compound **23** as analysed with the support of ^{1}H-^{13}C HSQC experiments. The quaternary carbon atoms are listed at the bottom.

δ (^{1}H)/ppm	δ (^{13}C)/ppm	HSQC phase
5.88	135.8	CH/CH_3
5.72	145.5	CH/CH_3
5.44	128.1	CH/CH_3
5.29	115.9	CH_2
5.18	111.5	CH_2
5.06	115.9	CH_2
4.94	111.5	CH_2
3.82	70.6	CH_2
3.79	76.3	CH_2
2.17 – 2.00	22.7	CH_2
1.65	14.0	CH/CH_3
1.51 – 1.39	42.2	CH_2
1.09	28.3	CH/CH_3
	132.8	C_q
	72.8	C_q

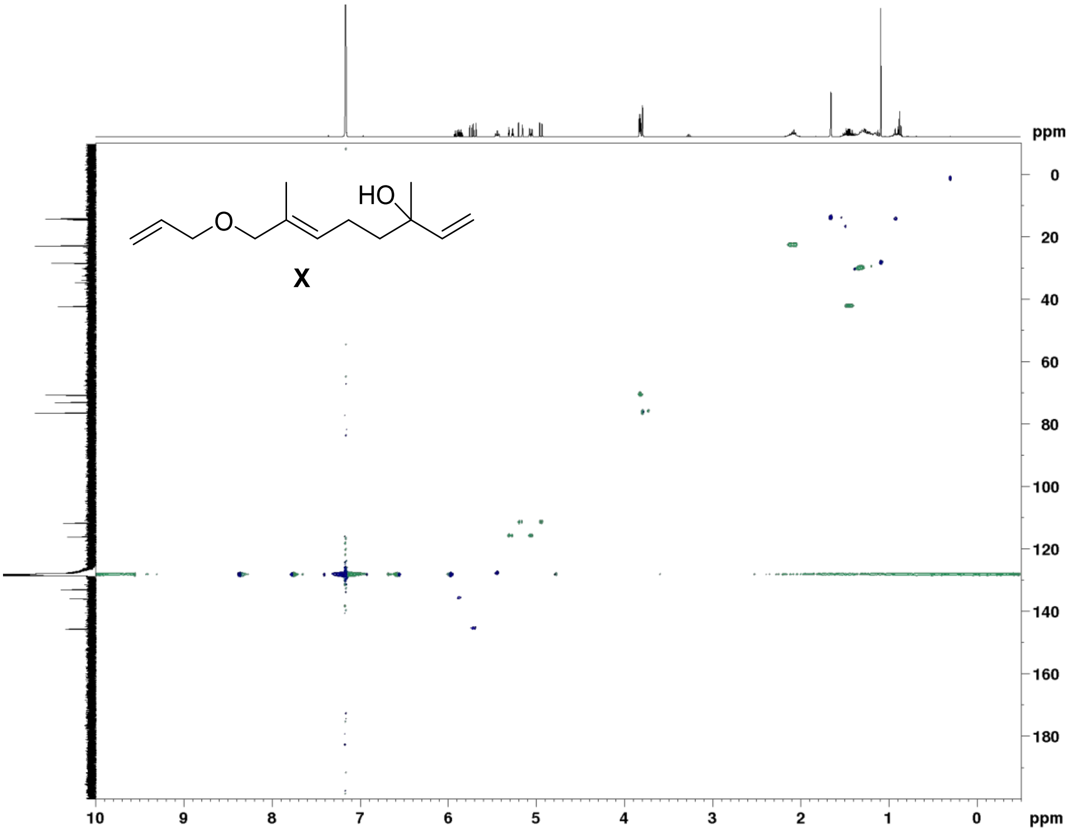

Figure S **65.** ^{1}H-^{13}C HSQC NMR spectrum of compound **23** in C_6D_6 (pos. phase = blue (CH/CH_3), neg. phase = green (CH_2)).

Table S **15**. ^{1}H NMR signals and the corresponding ^{1}H-^{1}H COSY correlations for compound **23**.

δ (^{1}H)/ppm	COSY correlations
5.88	5.29, 5.06, 3.82
5.72	5.18, 4.94
5.44	3.79, 2.17 – 2.00, 1.65
5.29	5.88, 5.06, 3.82
5.18	5.72, 4.94
5.06	5.88, 5.29, 3.82
4.94	5.72, 5.18
3.82	5.88, 5.29, 5.06
3.79	5.44, 1.65
2.17 – 2.00	5.44, 1.51 – 1.39
1.65	5.44, 3.79
1.51 – 1.39	2.17 – 2.00

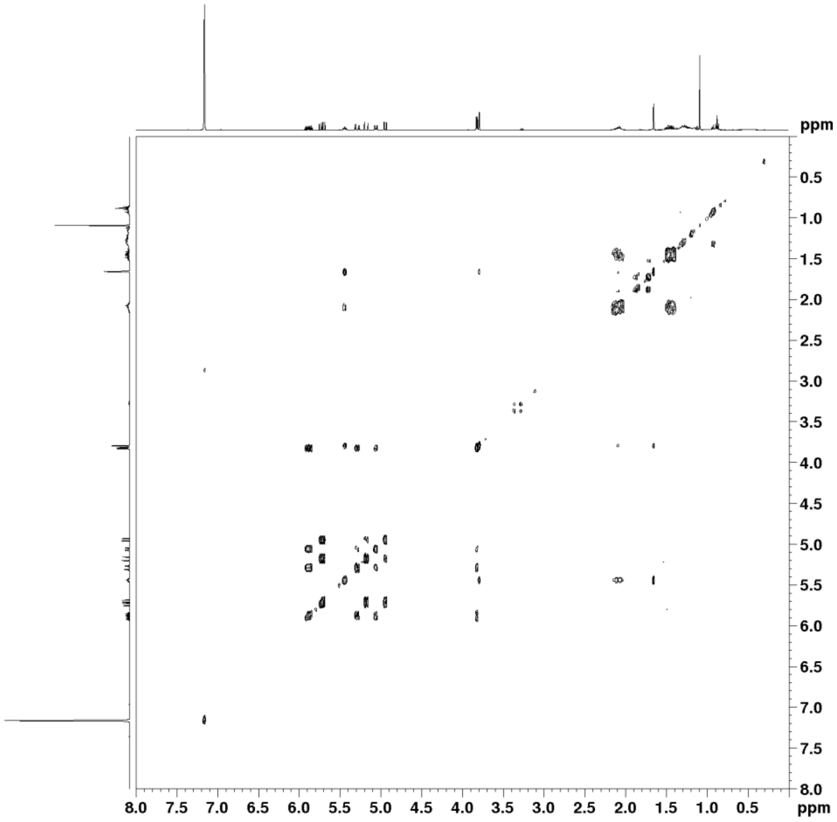

Figure S **66**. ^{1}H-^{1}H COSY NMR spectrum of compound **23** in C_6D_6.

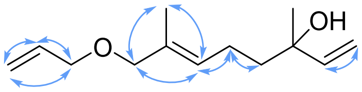

23

Figure S **67**. Key ^{1}H-^{1}H COSY NMR correlations of **23** as indicated by arrows.

Table S **16.** Selected correlations between ^{13}C NMR signals and neighbouring ^{1}H NMR signals as collected from the ^{1}H-^{13}C HMBC spectrum of compound **23**. Note: Signals with weak intensities are given in parentheses.

δ (^{1}H)/ppm	δ (^{13}C)/ppm
5.88	-
5.72	72.8
5.44	76.3, 14.0
5.29	70.6
5.18	72.8
5.06	70.6
4.94	72.8
3.82	135.8, 115.9, 76.3
3.79	132.8, 128.1, 70.6, 14.0

δ (^{1}H)/ppm	δ (^{13}C)/ppm
2.17 – 2.00	(132.8), 128.1, 42.2
1.65	132.8, 128.1, 76.3,
1.51 – 1.39	-
1.09	145.5, 72.8, 42.2

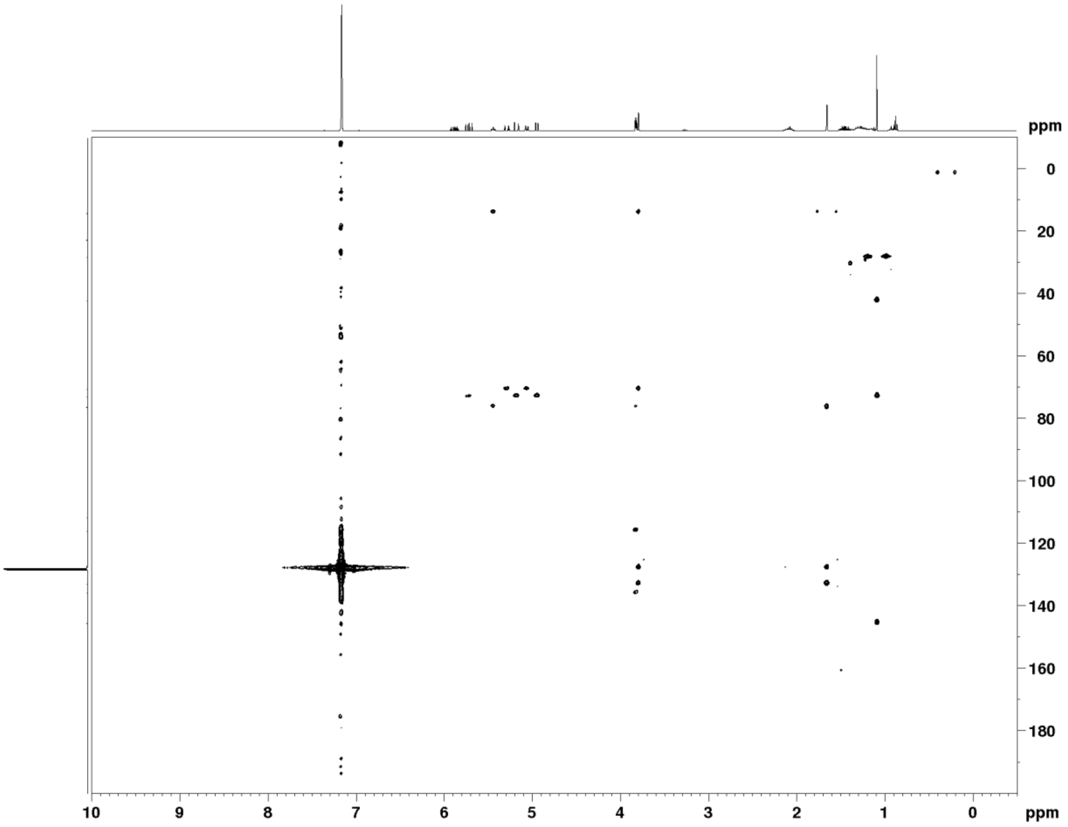

Figure S **68**. ^{1}H-^{13}C HMBC NMR spectrum of compound **23** in C_6D_6.

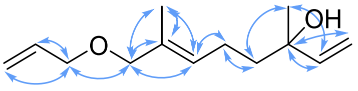

23

Figure S **69**. Key ^{1}H-^{13}C HMBC NMR correlations of **23** as indicated by arrows.

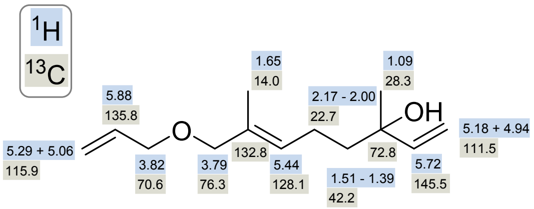

23

Figure S **70**. Full assignment of NMR spectra for **23**.

1.5.6 Structure elucidation of 16a and 16b

Table S **17**. ^{1}H NMR signals and their corresponding ^{13}C NMR signals for compound **16a** and **16b** as analysed with the support of ^{1}H-^{13}C HSQC experiments. The quaternary carbon atoms are listed at the bottom.

δ (^{1}H)/ppm	δ (^{13}C)/ppm	DEPT135/HSQC phase
9.40	204.0	CH/CH_3
9.39	204.0	CH/CH_3
5.38 – 5.30	124.8, 124.8	CH/CH_3, CH/CH_3
5.09 – 5.03	124.3, 123.2	CH/CH_3, CH/CH_3
3.85	68.6	CH_2
3.80	68.6	CH_2
2.14 – 2.00	50.1, 39.9, 39.9, 26.3, 26.1	CH/CH_3, CH_2, CH_2, CH_2, CH_2
1.92 – 1.66	51.0, 34.8 33.6, 33.4, 31.8	CH/CH_3, CH/CH_3, CH/CH_3, CH_2, CH_2
1.57	13.7	CH/CH_3
1.55	13.7	CH/CH_3
1.49	16.1	CH/CH_3
1.48	16.1	CH/CH_3
0.82	9.7	CH/CH_3
0.80	8.5	CH/CH_3
0.77	17.9	CH/CH_3
0.66	15.6	CH/CH_3
	136.5	C_q
	136.4	C_q
	135.6	C_q
	135.5	C_q

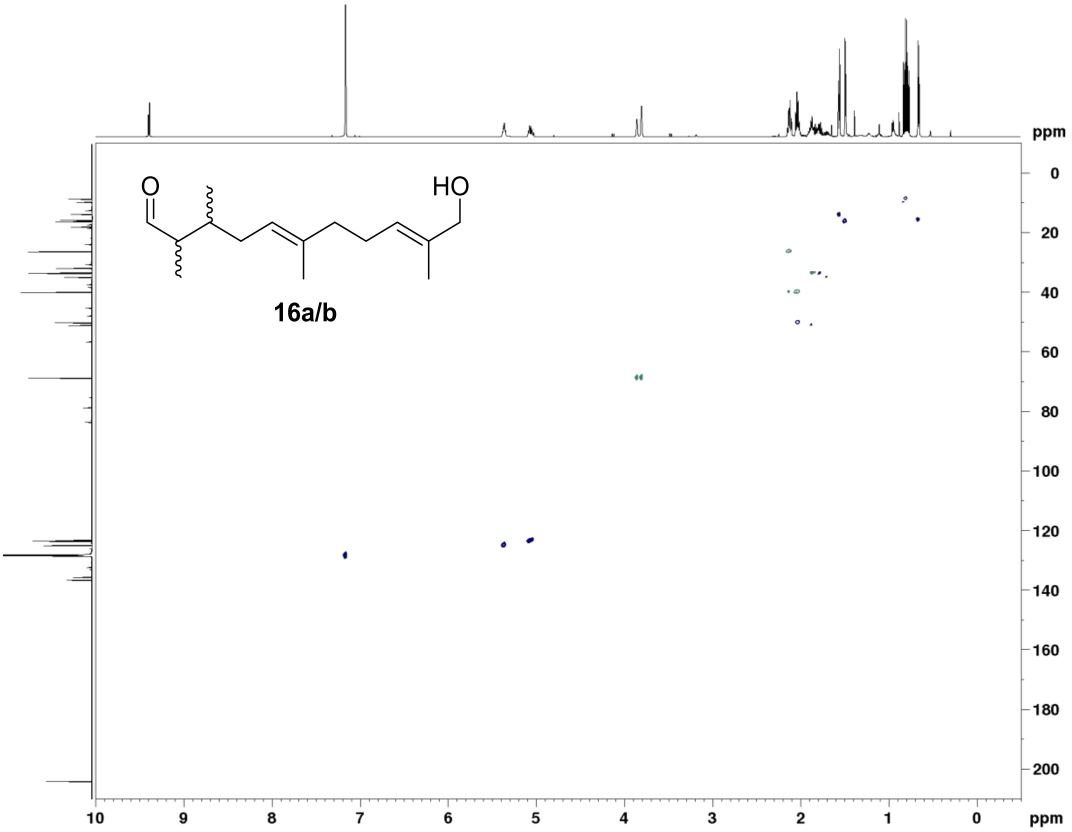

Figure S **71.** ^{1}H-^{13}C HSQC NMR spectrum of compound **16a/b** in C_6D_6 (pos. phase = blue (CH/CH_3), neg. phase = green (CH_2)).

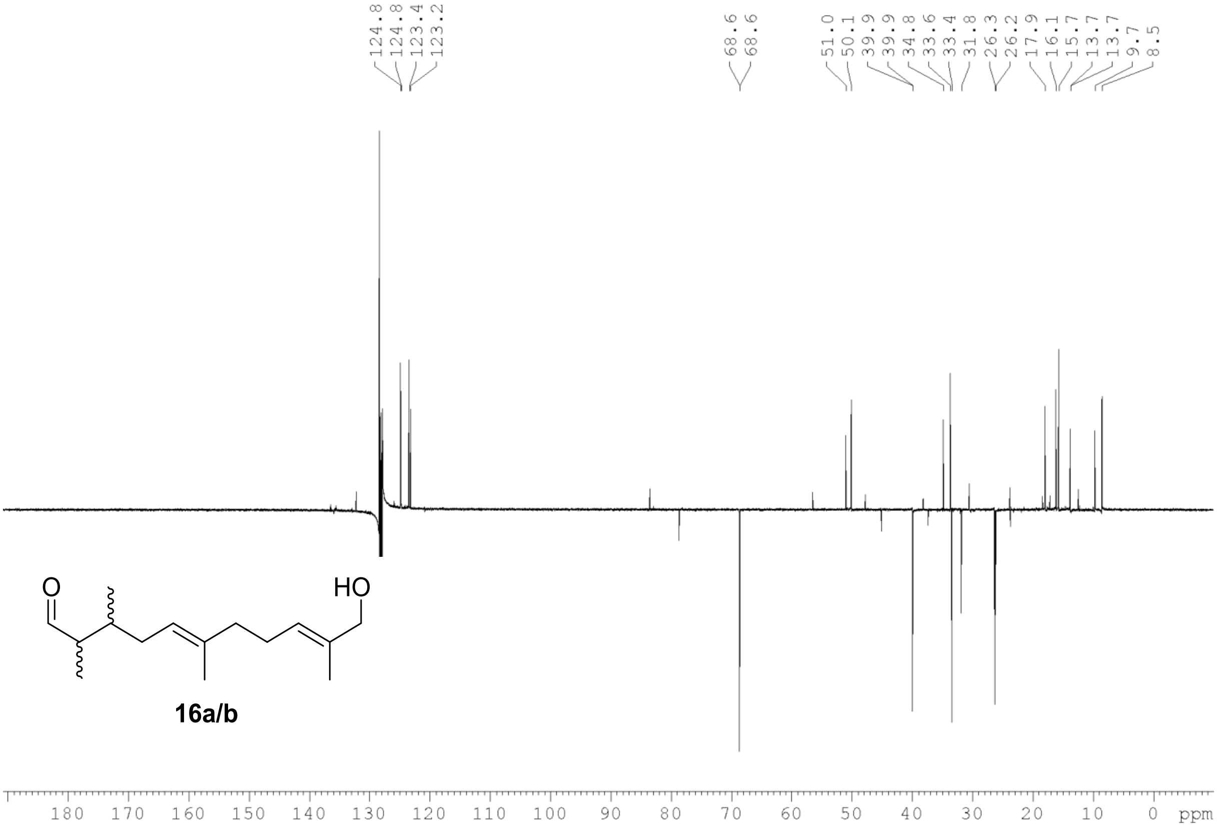

Figure S **72.** $^{13}C\{^{1}H\}$ DEPT135 NMR spectrum of compound **16a/b** in C_6D_6.

Table S **18**. ^{1}H NMR signals and the corresponding ^{1}H-^{1}H COSY correlations for compound **16a/b**.

δ (^{1}H)/ppm	COSY correlations
9.40	1.92 – 1.66
9.39	2.14 – 2.00
5.38 – 5.30	3.85, 3.80, 2.14 – 2.00, 1.57, 1.55
5.09 – 5.03	2.14 – 2.00, 1.92 – 1.66, 1.49, 1.48
3.85	5.38 – 5.30, 2.14 – 2.00, 1.57
3.80	5.38 – 5.30, 2.14 – 2.00, 1.55
2.14 – 2.00	5.38 – 5.30, 0.80
1.92 – 1.66	5.09 – 5.03, 0.82, 0.77, 0.66
1.57	5.38 – 5.30
1.55	5.38 – 5.30
1.49	5.09 – 5.03
1.48	5.09 – 5.03
0.82	1.92 – 1.66
0.80	2.14 – 2.00
0.77	1.92 – 1.66
0.66	1.92 – 1.66

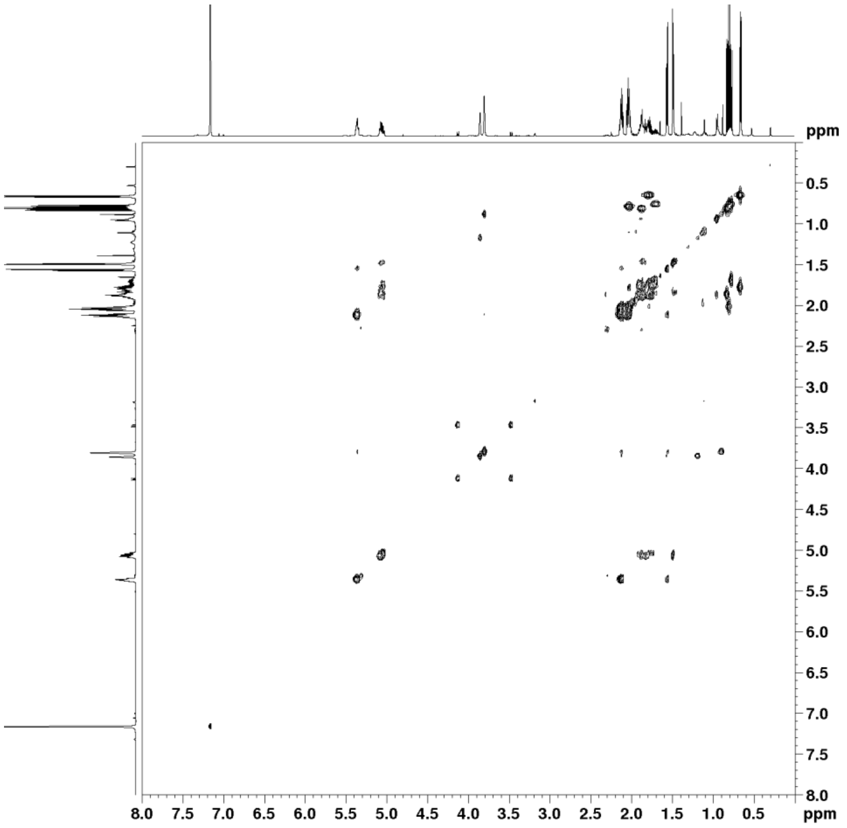

Figure S **73**. ^{1}H-^{1}H COSY NMR spectrum of compound **16a/b** in C_6D_6.

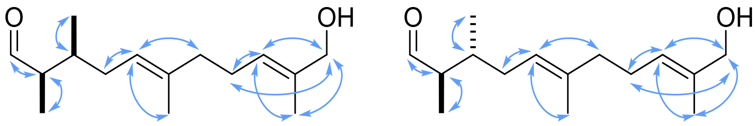

16a **16b**

Figure S **74**. Key ^{1}H-^{1}H COSY NMR correlations of **16a** and **16b** as indicated by arrows.

Table S **19**. Selected correlations between ^{13}C NMR signals and neighbouring ^{1}H NMR signals as collected from the ^{1}H-^{13}C HMBC spectrum of compounds **16a** and **16b**. Note: Ambiguous signals are marked by an "x".

δ (^{1}H)/ppm	δ (^{13}C)/ppm
9.40	51.0, 9.7
9.39	50.1, 8.5
5.38 – 5.30	68.6, 39.9, 26.3, 26.1, 13.7
5.09 – 5.03	31.8, 16.1, 33.x, 34.8, 39.9
3.85	135.x, 124.8, 13.7
3.80	135.x, 124.8, 13.7
2.14 – 2.00	204.0, 135.x, 124.8, 39.9, 16.1,
1.92 – 1.66	204.0, 136.x, 123.x, 50.1, 34.8, 33.x,
1.57	135.x, 124.8, 68.6
1.55	135.x, 124.8, 68.6
1.49	136.x, 123.x, 39.9
1.48	136.x, 123.x, 39.9
0.82	204.0, 51.0, 34.8
0.80	204.0, 50.1, 33.6,
0.77	51.0, 34.8, 31.8
0.66	50.1, 33.x

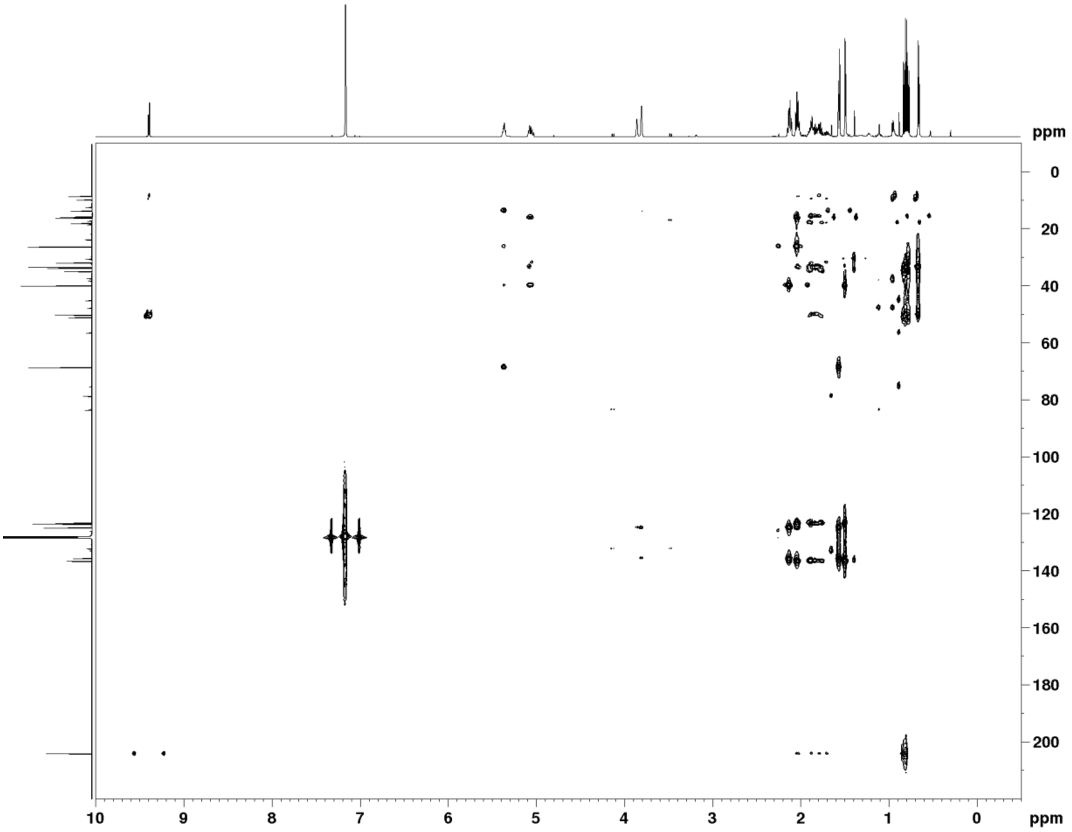

Figure S **75**. ^{1}H-^{13}C HMBC NMR spectrum of compound **16a/b** in C_6D_6.

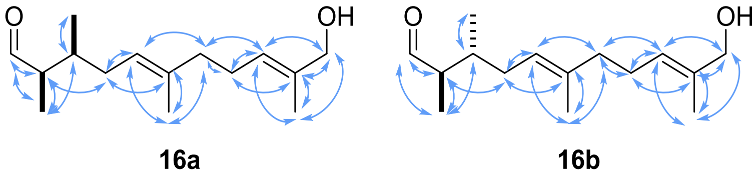

Figure S **76**. Key ^{1}H-^{13}C HMBC NMR correlations of **16a** and **16b** as indicated by arrows.

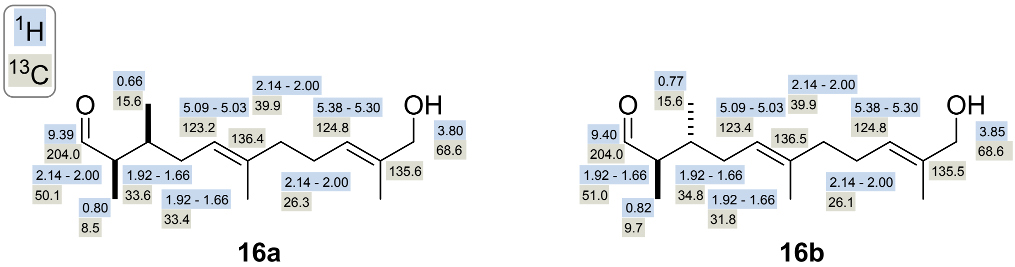

Figure S **77**. Full assignment of NMR spectra for **16a** and **16b**.

1.5.7 Structure elucidation of 22

During the structure elucidation the sample still contained *n*-pentane and Et_2O, which were removed by co-evaporation afterwards for a clean set of ^{1}H and ^{13}C NMR spectra.

Table S **20**. ^{1}H NMR signals and their corresponding ^{13}C NMR signals for compound **22** as analysed with the support of ^{1}H-^{13}C HSQC experiments. The quaternary carbon atoms are listed at the bottom.

δ (^{1}H)/ppm	δ (^{13}C)/ppm	DEPT135/HSQC phase
5.72	145.6	CH/CH$_3$
5.53 – 5.47	127.6, 121.7	CH/CH$_3$, CH/CH$_3$
5.18	111.6	CH$_2$
4.94	111.6	CH$_2$
3.81	76.0	CH$_2$
3.80	75.9	CH$_2$
2.18 – 2.04	22.9	CH$_2$
1.69	14.2	CH/CH$_3$
1.66	13.8	CH/CH$_3$
1.53	13.3	CH/CH$_3$
1.51 – 1.40	42.4	CH$_2$
1.09	28.4	CH/CH$_3$
	134.0	C$_q$
	133.2	C$_q$
	73.0	C$_q$

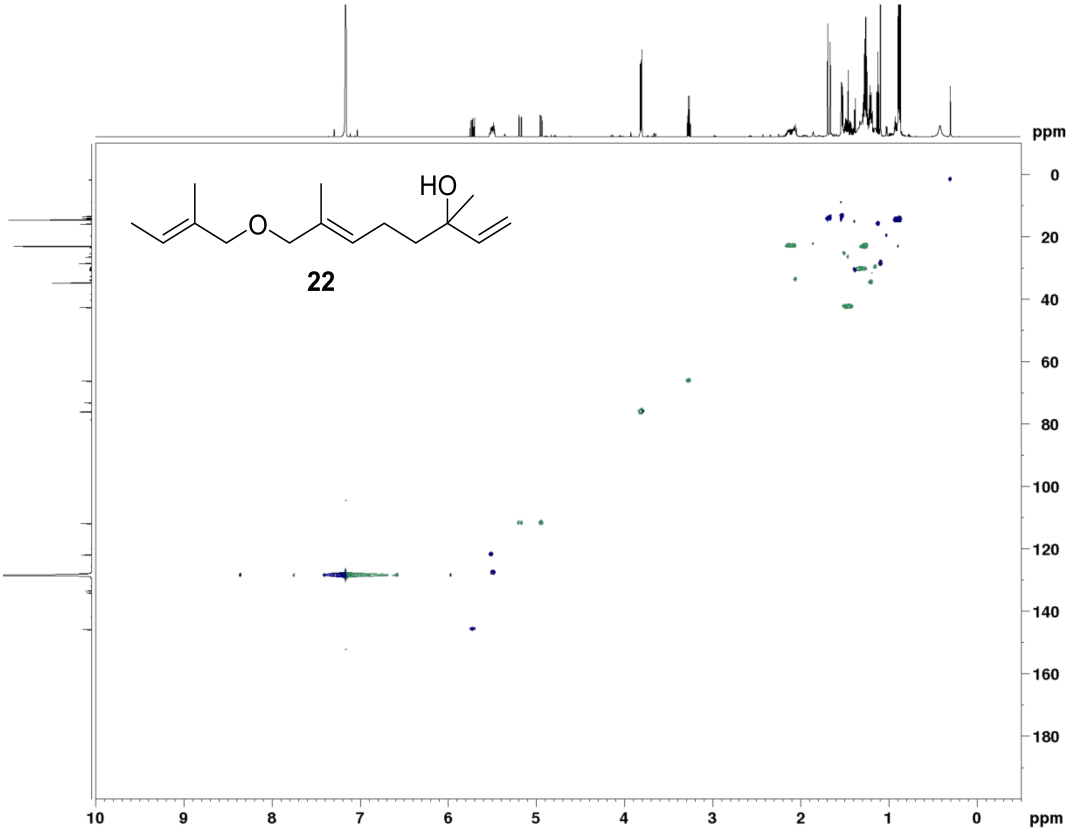

Figure S **78.** ^{1}H-^{13}C HSQC NMR spectrum of compound **22** in C_6D_6 (pos. phase = blue (CH/CH_3), neg. phase = green (CH_2)).

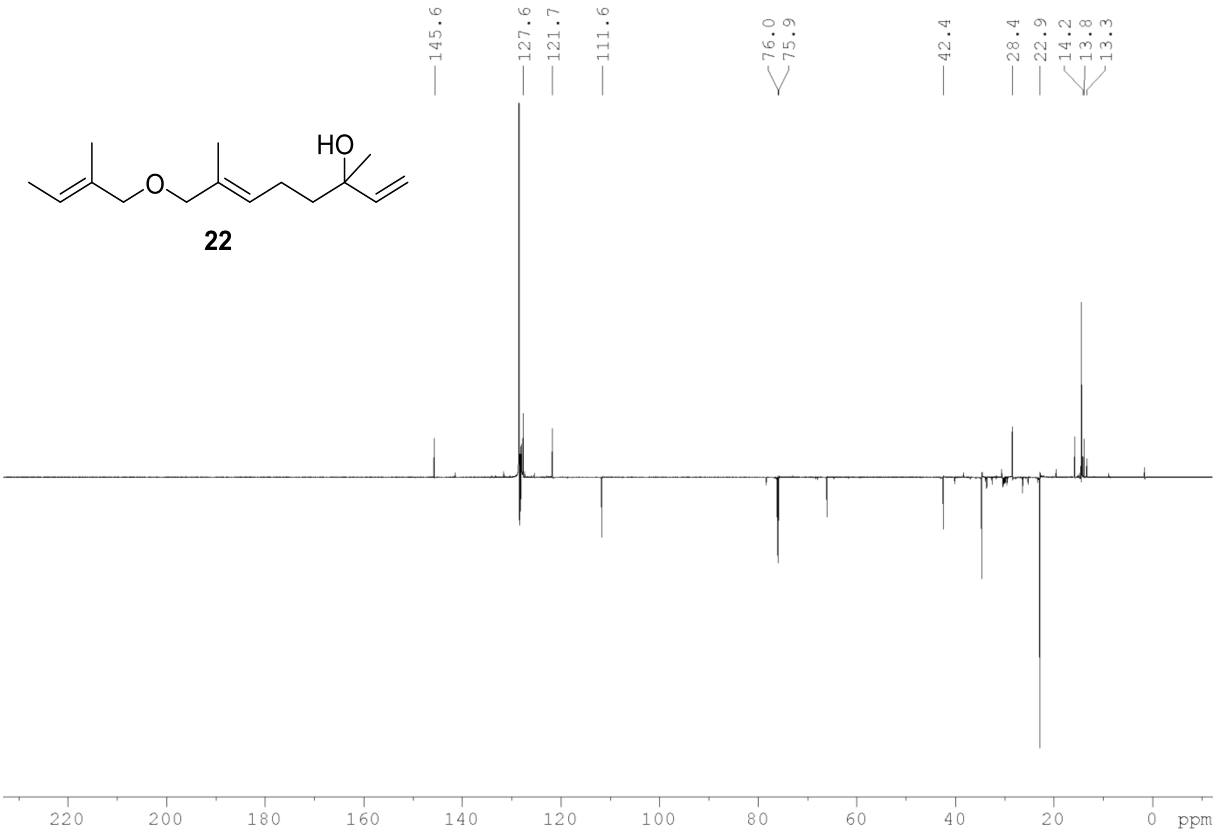

Figure S **79.** $^{13}C\{^1H\}$ DEPT135 NMR spectrum of compound **22** in C_6D_6.

Table S **21.** ^{1}H NMR signals and the corresponding ^{1}H-^{1}H COSY correlations for compound **22**.

δ (^{1}H)/ppm	COSY correlations
5.72	5.18, 4.94
5.53 – 5.47	3.81, 3.80, 2.18 – 2.04, 1.69, 1.66, 1.53
5.18	5.72, 4.94
4.94	5.72, 5.18
3.81	5.53 – 5.47, 1.66, 1.53
3.80	5.53 – 5.47, 1.69
2.18 – 2.04	5.53 – 5.47, 1.51 – 1.40
1.69	5.53 – 5.47, 3.80
1.66	5.53 – 5.47, 3.81, 1.53
1.53	5.53 – 5.47, 3.81, 1.66
1.51 – 1.40	2.18 – 2.04
1.09	-

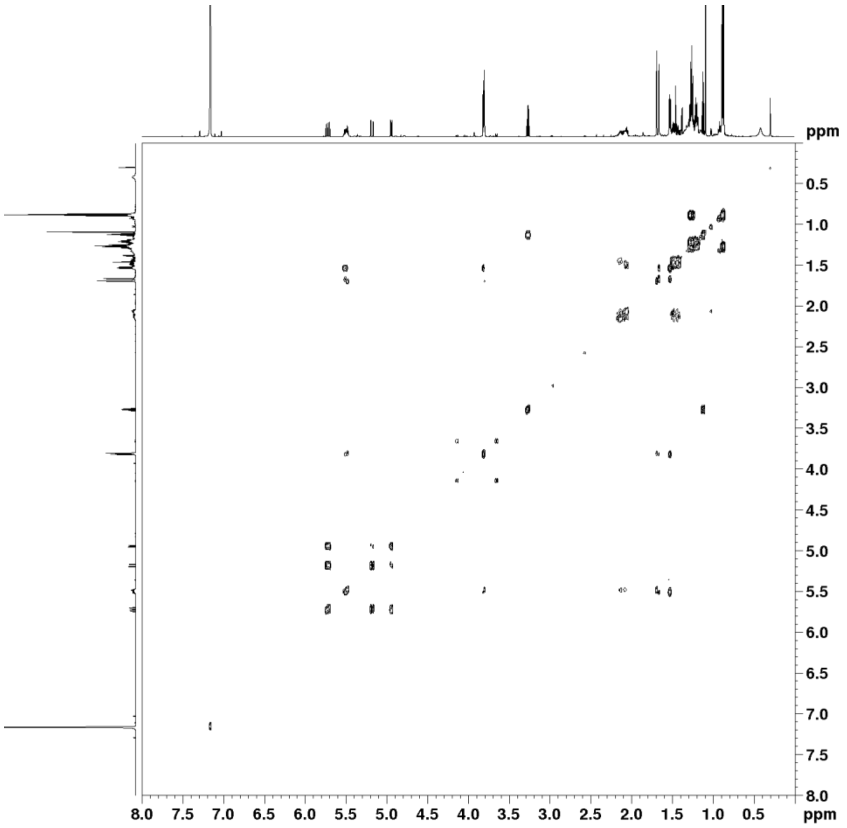

Figure S **80**. ^{1}H-^{1}H COSY NMR spectrum of compound **22** in C_6D_6.

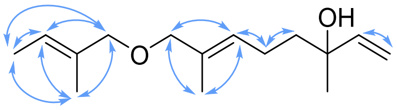

22

Figure S **81**. Key ^{1}H-^{1}H COSY NMR correlations of **22** as indicated by arrows.

Table S **22.** Selected correlations between ^{13}C NMR signals and neighbouring ^{1}H NMR signals as collected from the ^{1}H-^{13}C HMBC spectrum of compounds **22**. Note: Ambiguous signals are marked with an "x".

δ (^{1}H)/ppm	δ (^{13}C)/ppm
5.72	73.0
5.53 – 5.47	76.0, 75.9, 42.4, 22.9, 14.2, 13.x
5.18	145.6
4.94	-
3.81	134.0, 121.7, 75.9, 13.8,
3.80	133.2, 127.6, 76.0, 14.2
2.18 – 2.04	133.2, 127.6, 42.4
1.69	133.2, 127.6, 75.9
1.66	134.0, 121.7, 76.0

δ (^{1}H)/ppm	δ (^{13}C)/ppm
1.53	134.0, 121.7
1.51 – 1.40	-
1.09	145.6, 73.0, 42.4

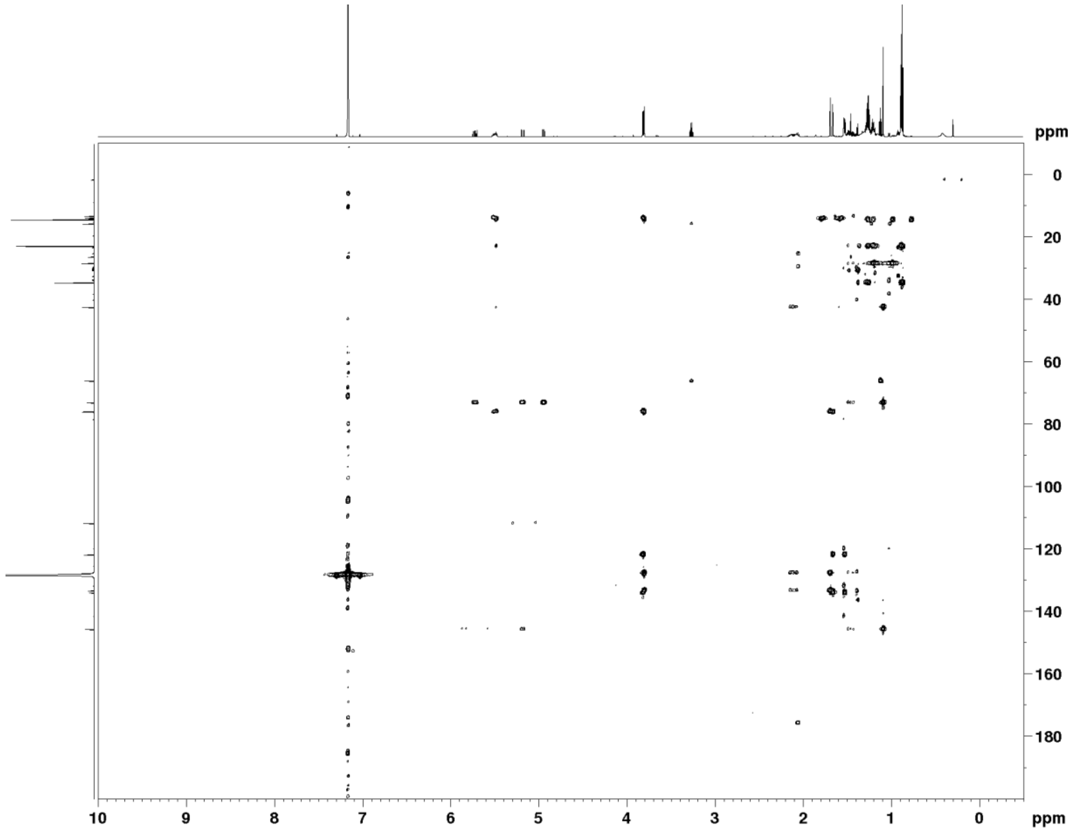

Figure S **82**. ^{1}H-^{13}C HMBC NMR spectrum of compound **22** in C_6D_6.

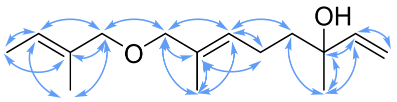

22

Figure S **83**. Key ^{1}H-^{13}C HMBC NMR correlations of **22** as indicated by arrows.

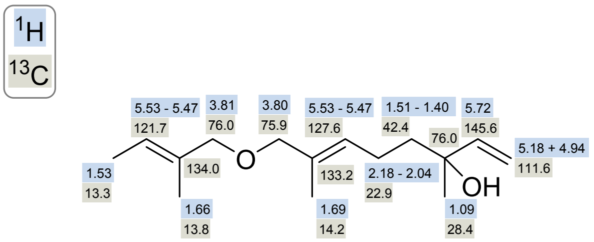

22

Figure S **84**. Full assignment of NMR spectra for **22**.

1.5.8 Structure elucidation of 17

During the structure elucidation the sample still contained *n*-pentane and Et_2O, which were removed by coevaporation afterwards for a clean set of ^{1}H and ^{13}C NMR spectras.

Table S **23**. ^{1}H NMR signals and their corresponding ^{13}C NMR signals for compound **17** as analysed with the support of ^{1}H-^{13}C HSQC experiments. The quaternary carbon atoms are listed at the bottom.

δ (^{1}H)/ppm	δ (^{13}C)/ppm	DEPT135/HSQC phase
5.35	141.3	CH/CH_3
4.89	131.5	CH/CH_3
4.79 – 4.77	127.2	CH/CH_3
4.14	78.2	CH_2
3.65	78.2	CH_2
2.22 – 2.15	25.2	CH_2
2.10 – 2.03	38.2, 33.8, 40.0	CH/CH_3, CH_2, CH_2
1.95	40.0	CH_2
1.87 – 1.83	25.2	CH_2
1.80 – 1.78	33.8	CH_2
1.54	8.7	CH/CH_3
1.54	14.7	CH/CH_3
1.39 – 1.38	15.0	CH/CH_3
1.02	19.4	CH/CH_3
	133.3	C_q
	131.7	C_q
	119.7	C_q

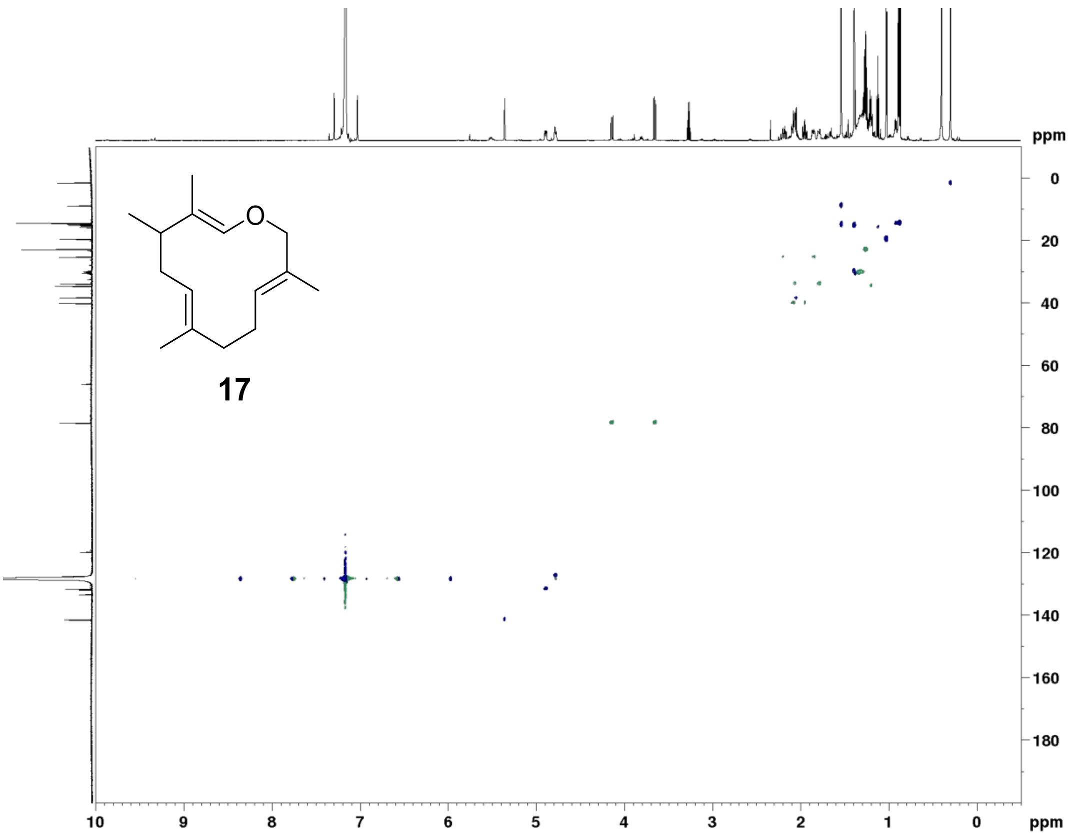

Figure S **85.** ^{1}H-^{13}C HSQC NMR spectrum of compound **17** in C_6D_6 (pos. phase = blue (CH/CH_3), neg. phase = green (CH_2)).

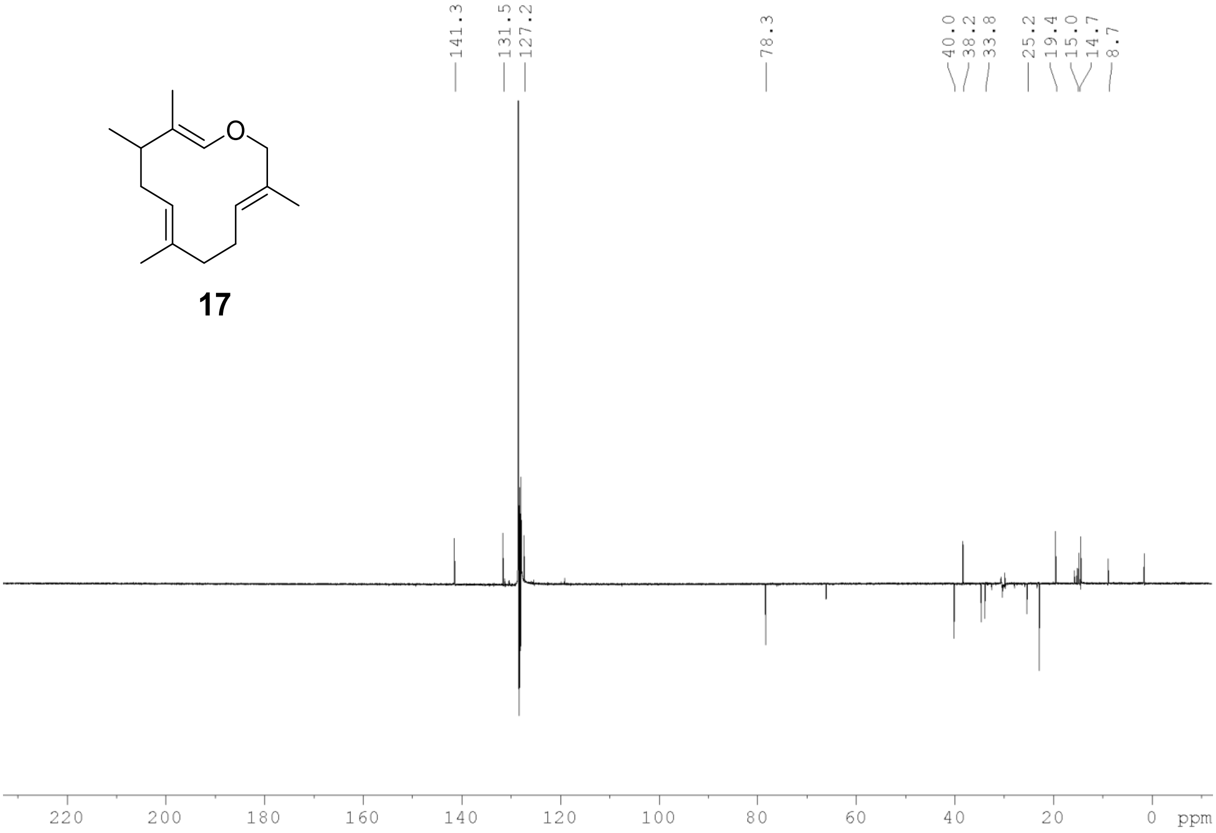

Figure S **86.** $^{13}C\{^{1}H\}$ DEPT135 NMR spectrum of compound **17** in C_6D_6.

Table S **24**. ^{1}H NMR signals and the corresponding ^{1}H-^{1}H COSY correlations for compound **17**. Signals with weak intensities are given in parentheses.

δ (^{1}H)/ppm	COSY correlations
5.35	1.54
4.89	2.22 – 2.15, 1.87 – 1.83, 1.54
4.79 – 4.77	2.10 – 2.03, 1.80 – 1.78, 1.39 – 1.38, 1.02
4.14	(4.89), 3.65, 1.87 – 1.83, 1.54
3.65	4.14, 1.54
2.22 – 2.15	4.89, 2.10 – 2.03, 1.95, 1.87 – 1.83
2.10 – 2.03	4.79 – 4.77, (2.22 – 2.03), 1.95, 1.80 – 1.78, 1.02
1.95	(2.22 – 2.15), 2.10 – 2.03, 1.87 – 1.83
1.87 – 1.83	2.22 – 2.15, 2.10 – 2.03, 1.95
1.80 – 1.78	4.79 – 4.77, 2.10 – 2.03, (1.39 – 1.38, 1.02)
1.54	5.35, 4.89, 1.87 – 1.83
1.39 – 1.38	4.79 – 4.77, 1.80 – 1.78
1.02	2.10 – 2.03, 1.80 – 1.78

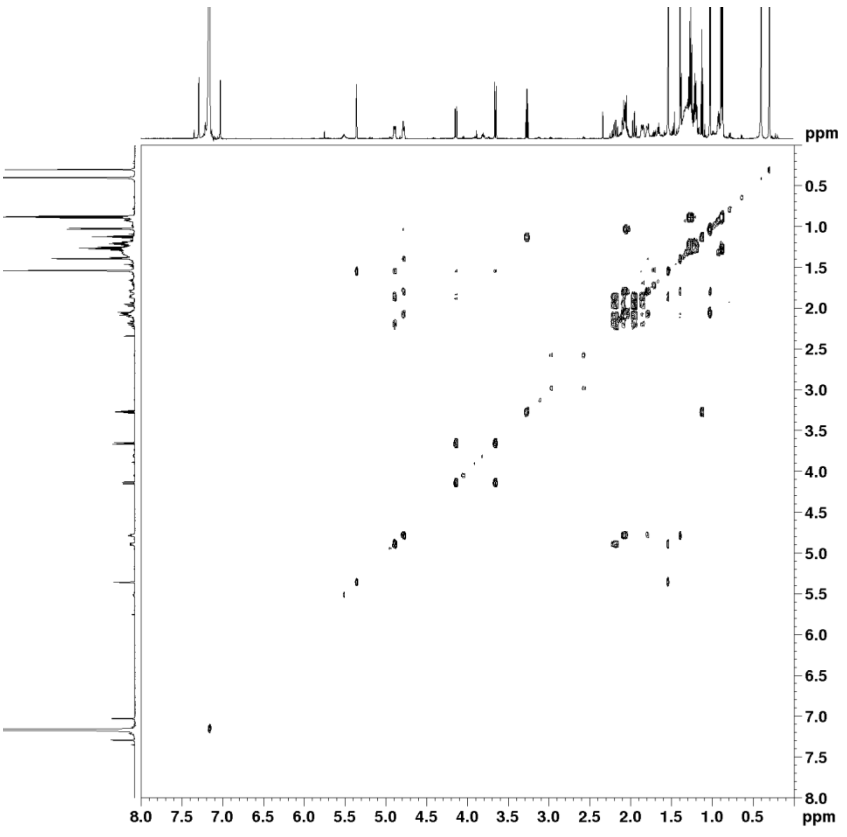

Figure S **87.** ^{1}H-^{1}H COSY NMR spectrum of compound **17** in C_6D_6.

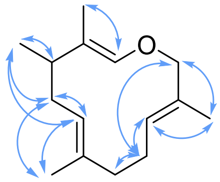

17

Figure S **88**. Key ^{1}H-^{1}H COSY NMR correlations of **17** as indicated by arrows.

Table S **25.** Selected correlations between ^{13}C NMR signals and neighbouring ^{1}H NMR signals as collected from the ^{1}H-^{13}C HMBC spectrum of compound **17**. Note: Ambiguous signals are marked by an “x”.

δ (^{1}H)/ppm	δ (^{13}C)/ppm
5.35	119.7, 78.2, 38.2, 8.7
4.89	-
4.79 – 4.77	15.0
4.14	141.3, 131.x, 14.7
3.65	141.3, 131.x, 14.7
2.22 – 2.15	131.x, 40.0
2.10 – 2.03	38.2
1.95	133.3, 127.2, 25.2, 14.7/15.0
1.87 – 1.83	40.0
1.80 – 1.78	-
1.54	141.3, 131.x, 119.7, 78.2, 38.2
1.39 – 1.38	133.3, 127.2, 40.0
1.02	119.7, 38.2, 33.8

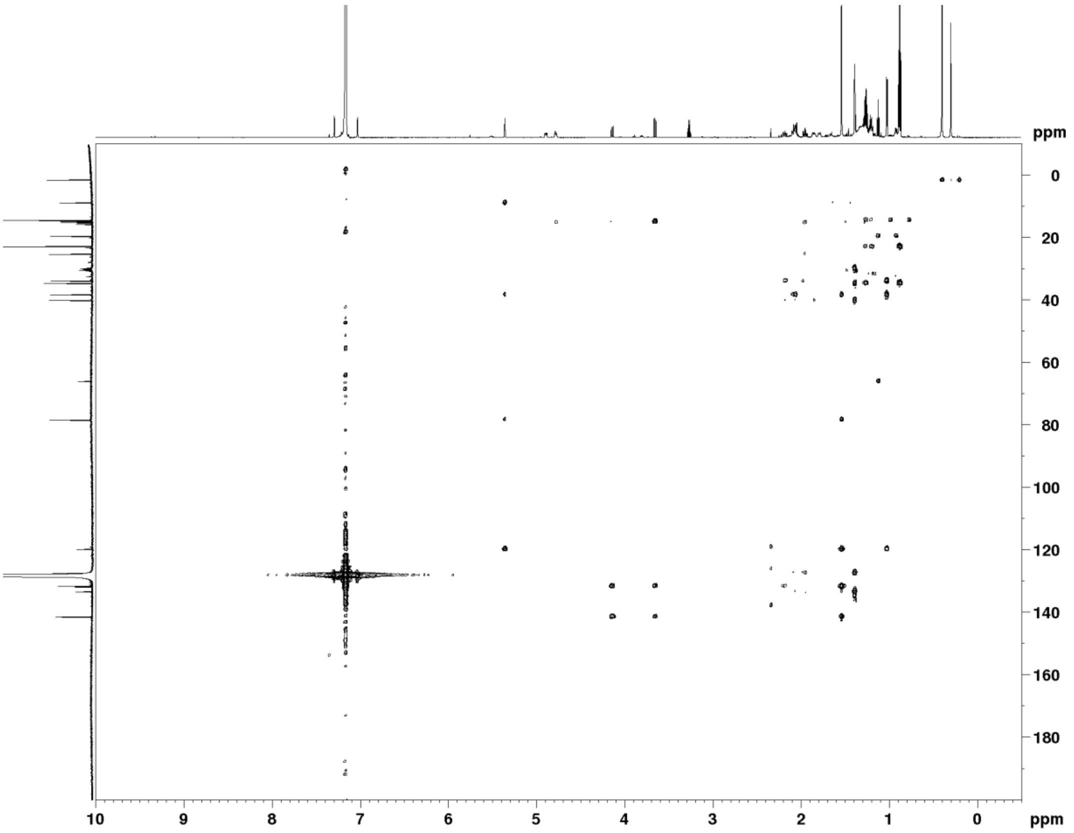

Figure S **89**. ^{1}H-^{13}C HMBC NMR spectrum of compound **17** in C_6D_6.

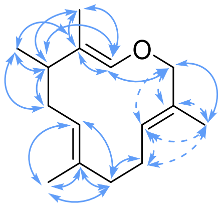

17

Figure S **90**. Key ^{1}H-^{13}C HMBC NMR correlations of **17** as indicated by arrows.

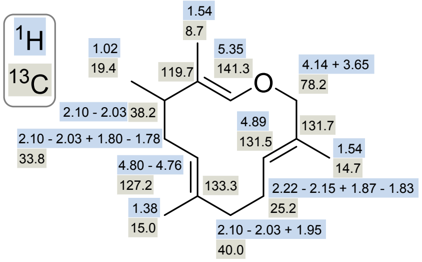

17

The NOESY spectra analysis shows no NOE signal for 5.35 and 1.54 which indicates a *trans* configurated double bond formed during the cyclization cascade.

Figure S **91**. Full assignment of NMR spectra for **X**.

1.5.9 Structure elucidation of 15

Table S **26**. ^{1}H NMR signals and their corresponding ^{13}C NMR signals for compound **15** as analysed with the support of ^{1}H-^{13}C HSQC experiments. The quaternary carbon atoms are listed at the bottom.

δ (^{1}H)/ppm	δ (^{13}C)/ppm	HSQC phase
5.63	143.8	CH/CH_3
5.02	124.3	CH/CH_3
4.91	131.0	CH/CH_3
4.79	119.9	CH/CH_3
3.91	76.9	CH_2
2.06 – 2.02	39.9, 25.2	CH_2, CH_2
1.89	42.0	CH_2
1.51	14.5	CH/CH_3
1.38	15.2	CH/CH_3
1.02	28.2	CH_2, CH_2
	134.2	C_q
	131.1	C_q
	35.0	C_q

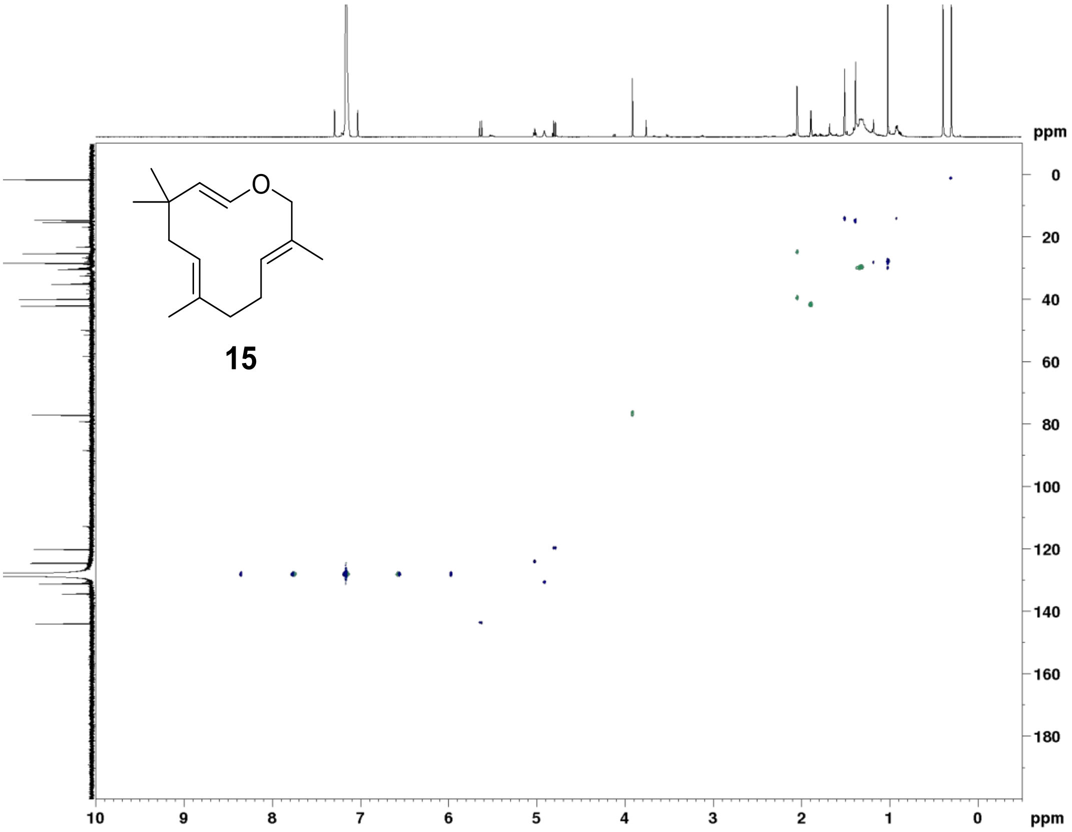

Figure S **92.** ^{1}H-^{13}C HSQC NMR spectrum of compound **15** in C_6D_6 (pos. phase = blue (CH/CH_3), neg. phase = green (CH_2)).

Table S **27**. ^{1}H NMR signals and the corresponding ^{1}H-^{1}H COSY correlations for compound **15**. Signals with weak intensities are given in parentheses.

δ (^{1}H)/ppm	COSY correlations
5.63	4.79
5.02	(2.06 – 2.02), 1.89, 1.38
4.91	2.06 – 2.02, (1.51)
4.79	5.63
3.91	4.91, 2.06 – 2.02
2.06 – 2.02	4.91, (3.91), 1.51
1.89	5.02, 2.06 – 2.02, 1.38
1.51	4.91, 2.06 – 2.02
1.38	5.02, 1.89
1.02	-

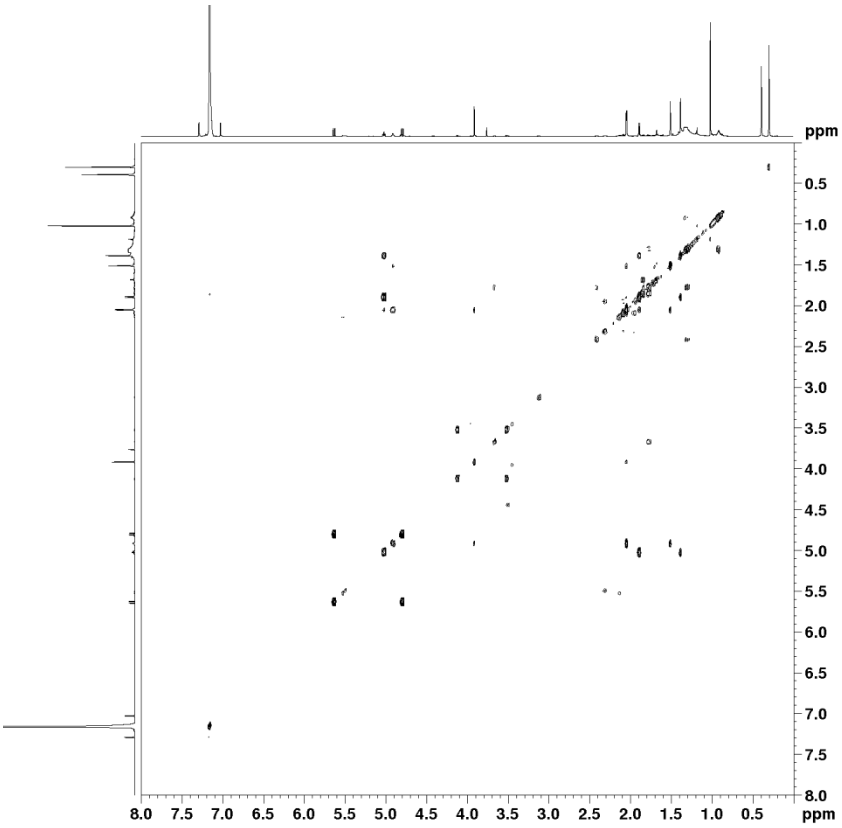

Figure S **93**. ^{1}H-^{1}H COSY NMR spectrum of compound **15** in C_6D_6.

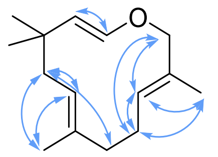

15

Figure S **94**. Key ^{1}H-^{1}H COSY NMR correlations of **15** as indicated by arrows.

Table S **28**. Selected correlations between ^{13}C NMR signals and neighbouring ^{1}H NMR signals as collected from the ^{1}H-^{13}C HMBC spectrum of compound **15**. Note: Ambiguous signals are marked by an "x".

δ (^{1}H)/ppm	δ (^{13}C)/ppm
5.63	119.9, 76.9
5.02	39.9, 15.2
4.91	-
4.79	143.8, 28.2
3.91	143.8, 131.x, 14.5
2.06 – 2.02	131.x, 124.3, 39.9, 25.2, 15.4
1.89	134.2, 124.3, 119.9, 35.0, 28.2
1.51	131.x, 76.9
1.38	134.2, 124.3, 39.9
1.02	119.9, 42.0, 35.0, 28.2

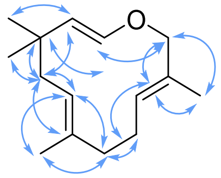

15

Figure S **95**. Key ^{1}H-^{13}C HMBC NMR correlations of **15** as indicated by arrows.

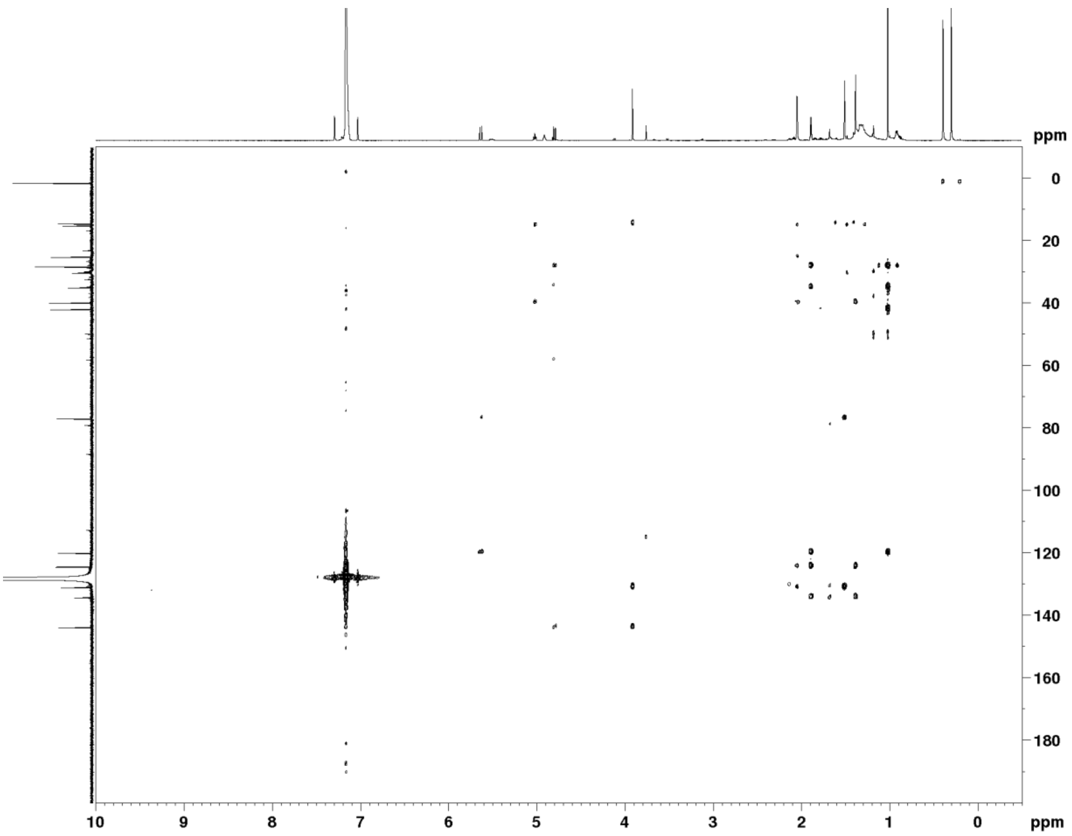

Figure S **96**. ^{1}H-^{13}C HMBC NMR spectrum of compound **15** in C_6D_6.

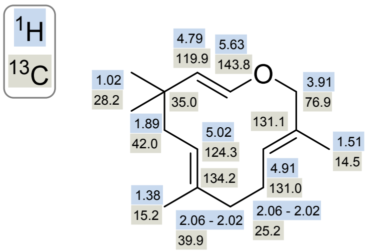

15

The NOESY spectra analysis shows no NOE signal for 5.63 and 4.79 which indicates a *trans* configurated double bond formed during the cyclization cascade.

Figure S **97**. Full assignment of NMR spectra for **15**.

1.5.10 Structure elucidation of 6

Table S **29**. ^{1}H NMR signals and their corresponding ^{13}C NMR signals for compound **6** as analysed with the support of ^{1}H-^{13}C HSQC experiments. The quaternary carbon atoms are listed at the bottom.

δ (^{1}H)/ppm	δ (^{13}C)/ppm	HSQC phase
5.72	145.5	CH/CH_3
5.54 – 5.52	122.8	CH/CH_3
5.49	127.6	CH/CH_3
5.18	111.5	CH_2
4.94	111.5	CH_2

δ (^{1}H)/ppm	δ (^{13}C)/ppm	HSQC phase
3.96	66.5	CH_2
3.85	76.1	CH_2
2.18 – 2.04	22.8	CH_2
1.70	14.1	CH/CH_3
1.60	25.7	CH/CH_3
1.52	18.0	CH/CH_3
1.50 – 1.41	42.3	CH_2
1.09	28.3	CH/CH_3
	135.3	C_q
	133.2	C_q
	72.9	C_q

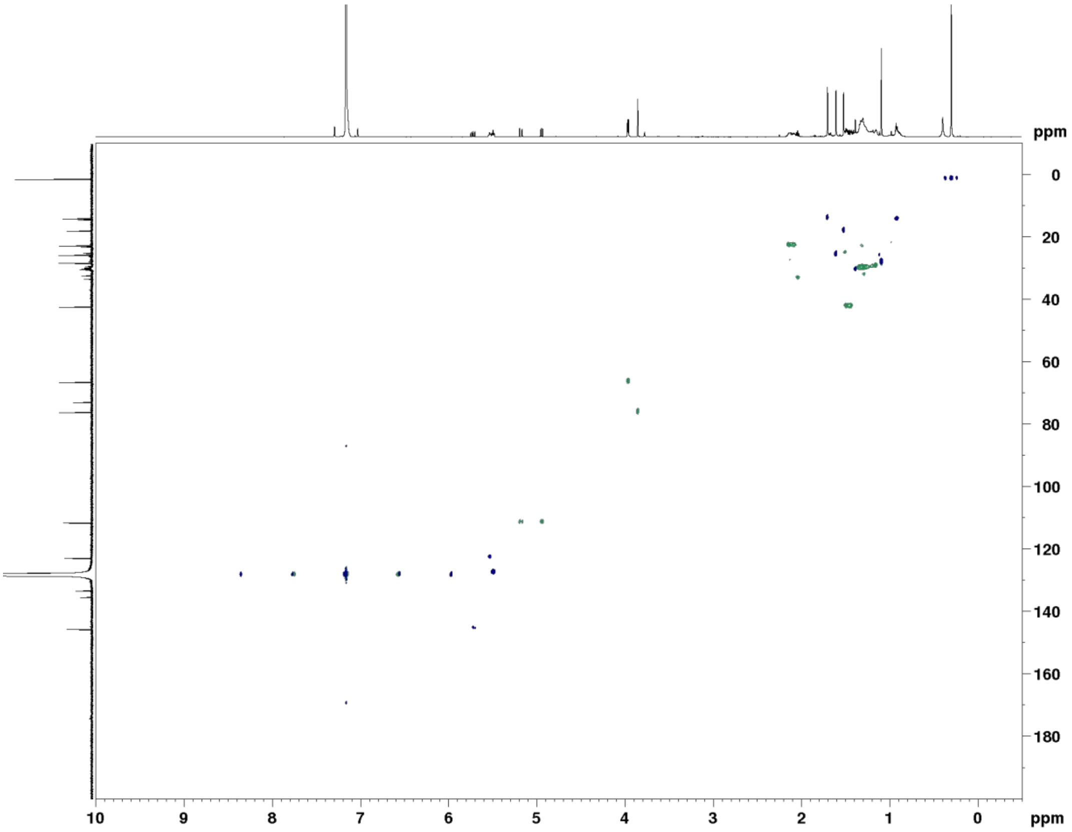

Figure S **98.** ^{1}H-^{13}C HSQC NMR spectrum of compound **6** in C_6D_6 (pos. phase = blue (CH/CH_3), neg. phase = green (CH_2)).

Table S **30**. ^{1}H NMR signals and the corresponding ^{1}H-^{1}H COSY correlations for compound **6**.

δ (^{1}H)/ppm	COSY correlations
5.72	5.18, 4.94
5.54 – 5.52	3.96, 1.60, 1.52
5.49	3.85, 2.18 – 2.04, 1.70
5.18	5.72, 4.94
4.94	5.72, 5.18
3.96	5.54 – 5.52, 1.60, 1.52
3.85	5.49, 1.70
2.18 – 2.04	5.49, 3.85, 1.50 – 1.41
1.70	5.49, 3.85
1.60	5.54 – 5.52, 3.96, 1.52
1.52	5.54 – 5.52, 3.96, 1.60
1.50 – 1.41	2.18 – 2.04
1.09	-

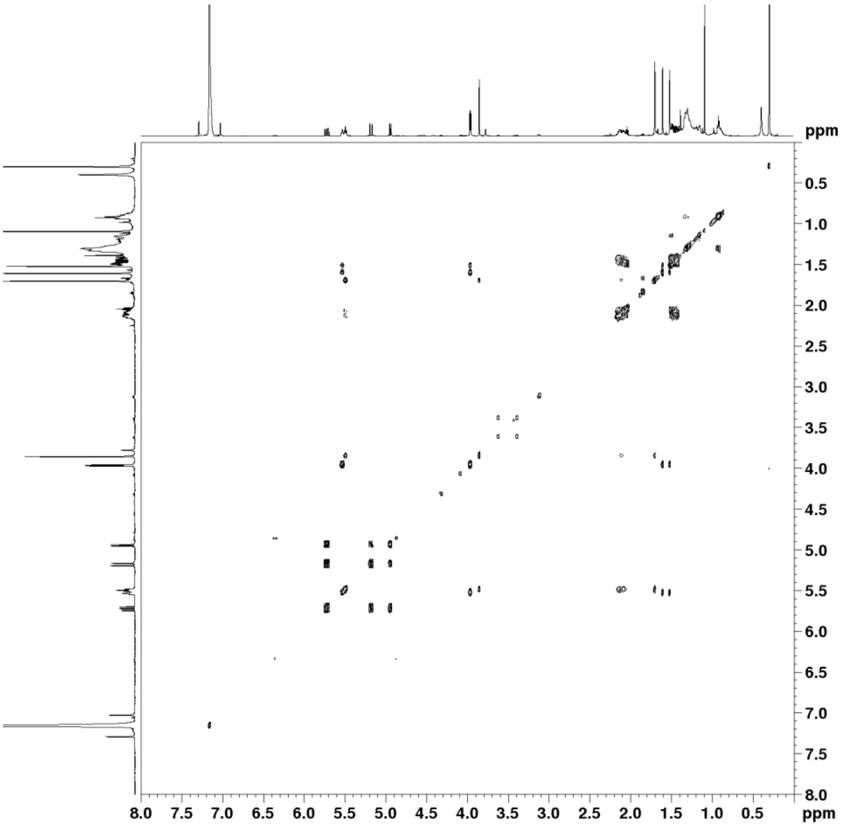

Figure S **99**. ^{1}H-^{1}H COSY NMR spectrum of compound **6** in C_6D_6.

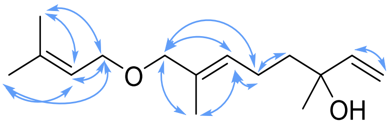

6

Figure S **100**. Key ^{1}H-^{1}H COSY NMR correlations of **6** as indicated by arrows.

Table S **31**. Selected correlations between ^{13}C NMR signals and neighbouring ^{1}H NMR signals as collected from the ^{1}H-^{13}C HMBC spectrum of compound **6**.

δ (^{1}H)/ppm	δ (^{13}C)/ppm
5.72	-
5.54 – 5.52	-
5.49	76.1, 14.1
5.18	72.9
4.94	72.9
3.96	135.3, 122.8, 76.1
3.85	133.2, 127.6, 66.5
2.18 – 2.04	-
1.70	133.2, 127.6, 76.1
1.60	135.3, 122.8, 18.0
1.52	135.3, 122.8, 25.7
1.50 – 1.41	-
1.09	145.5, 72.9, 42.3

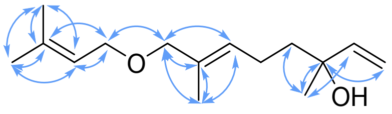

6

Figure S **101**. Key ^{1}H-^{13}C HMBC NMR correlations of **6** as indicated by arrows.

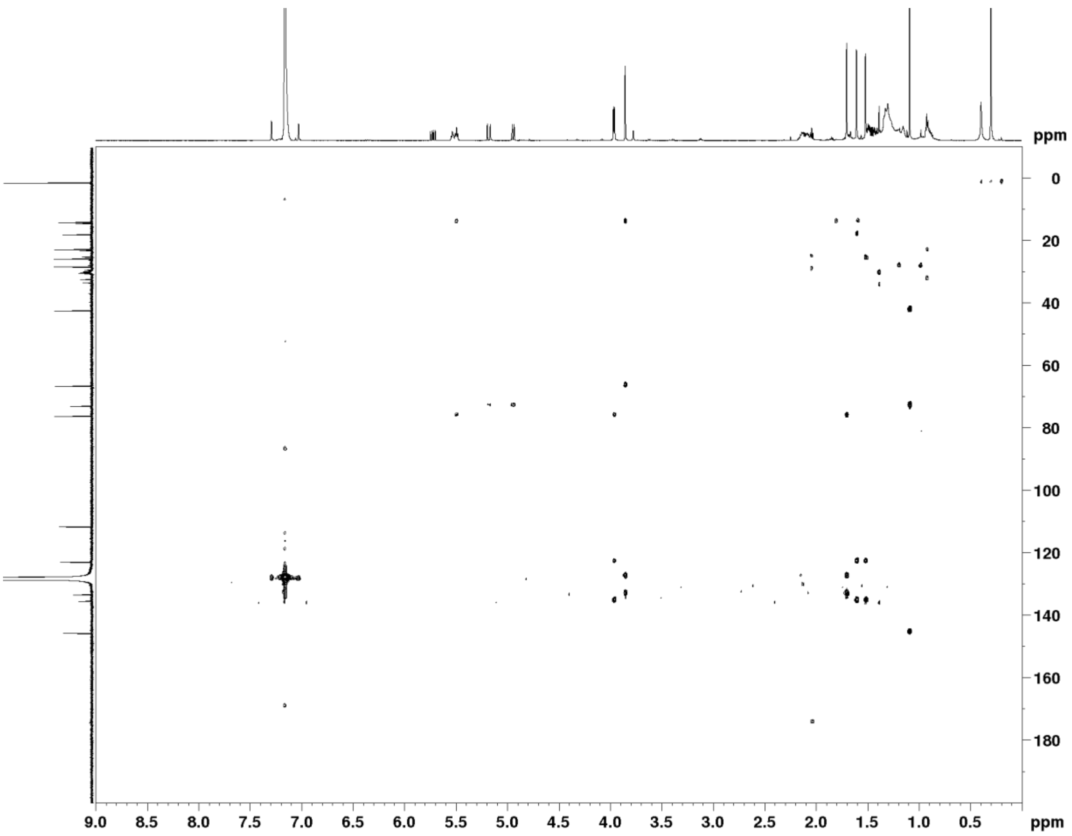

Figure S **102**. ^{1}H-^{13}C HMBC NMR spectrum of compound **6** in C_6D_6.

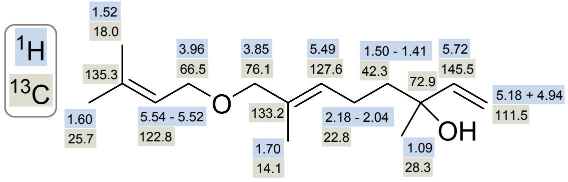

6

Figure S **103**. Full assignment of NMR spectra for **6**.

1.5.11 Structure elucidation of 29

Table S **32**. ^{1}H NMR signals and their corresponding ^{13}C NMR signals for compound **29** as analysed with the support of ^{1}H-^{13}C HSQC experiments. The quaternary carbon atoms are listed at the bottom.

δ (^{1}H)/ppm	δ (^{13}C)/ppm	DEPT135/HSQC phase
6.34	139.3	CH/CH_3
5.88	135.8	CH/CH_3
5.45 – 5.42	127.2	CH/CH_3
5.28	115.9	CH_2
5.17	113.2	CH_2
5.07 – 5.04	115.9	CH_2

δ (^{1}H)/ppm	δ (^{13}C)/ppm	DEPT135/HSQC phase
4.97 – 4.95	116.1, 113.2	CH_2, CH_2
3.81	70.5	CH_2
3.79	76.2	CH_2
2.21	31.5, 26.6	CH_2, CH_2
1.61	14.0	CH/CH_3
	146.2	C_q
	133.2	C_q

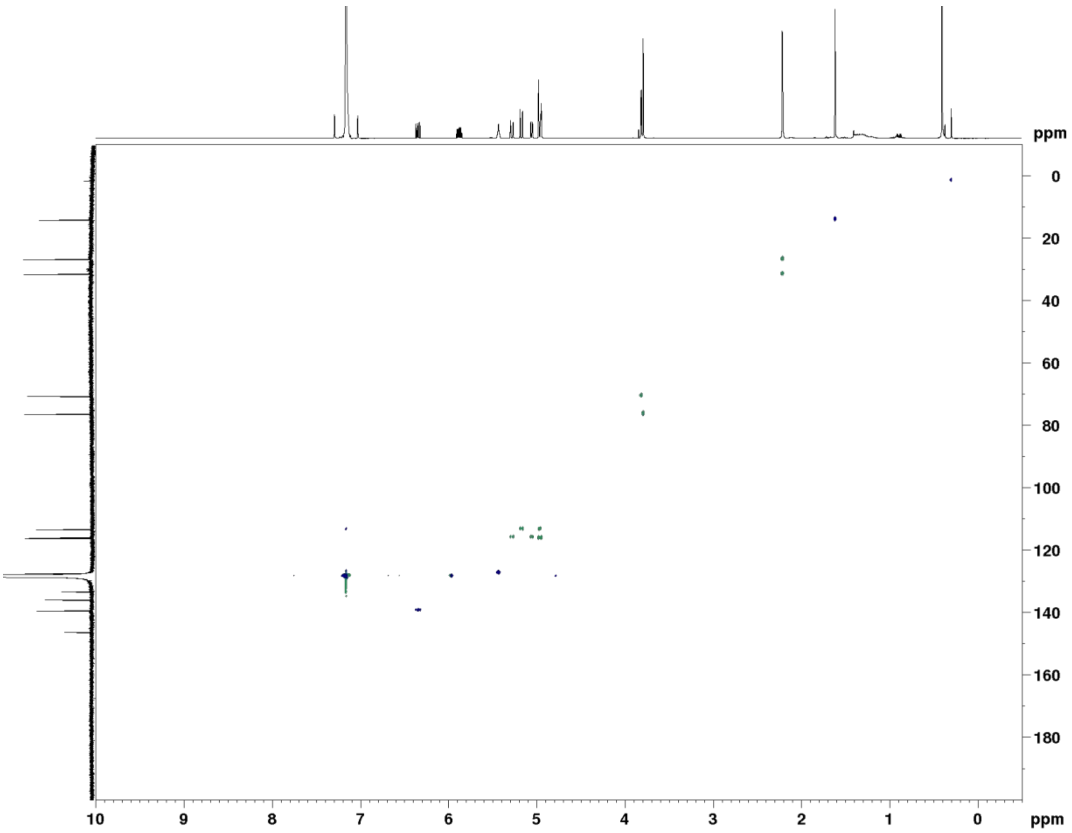

Figure S **104.** ^{1}H-^{13}C HSQC NMR spectrum of compound **29** in C_6D_6 (pos. phase = blue (CH/CH_3), neg. phase = green (CH_2)).

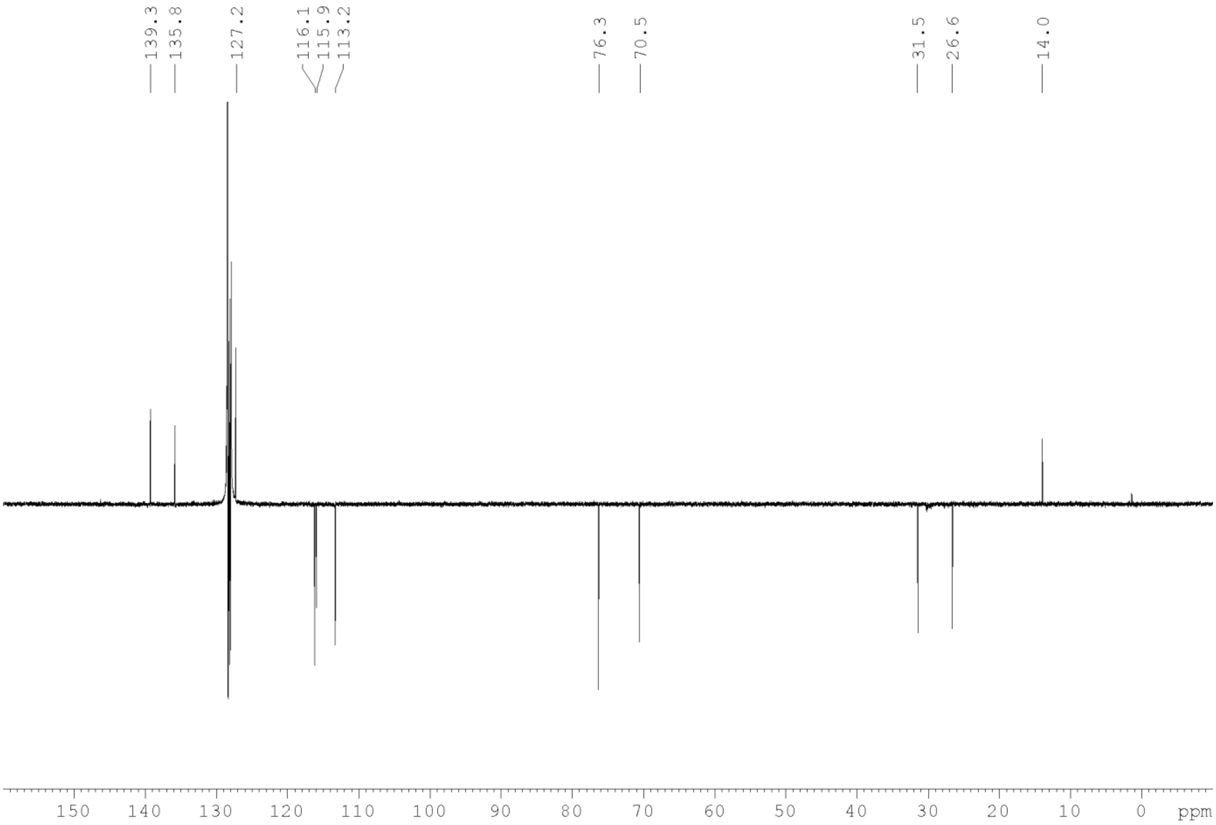

Figure S **105.** $^{13}C\{^{1}H\}$ DEPT135 NMR spectrum of compound **29** in C_6D_6.

Table S **33.** ^{1}H NMR signals and the corresponding ^{1}H-^{1}H COSY correlations for compound **29**.

δ (^{1}H)/ppm	COSY correlations
6.34	5.17, 4.97 – 4.95
5.88	5.28, 5.07 – 5.04, 3.81
5.45 – 5.42	3.79, 2.21, 1.61
5.28	5.88, 5.07 – 5.04, 3.81
5.17	6.34, 4.97 – 4.95,
5.07 – 5.04	5.88, 5.28, 3.81
4.97 – 4.95	6.34, 5.17, 2.21
3.81	5.88, 5.28, 5.07 – 5.04
3.79	5.45 – 5.42, 2.21, 1.61
2.21	5.45 – 5.42, 4.97 – 4.95, 3.79, 1.61
1.61	5.45 – 5.42, 3.79, 2.21

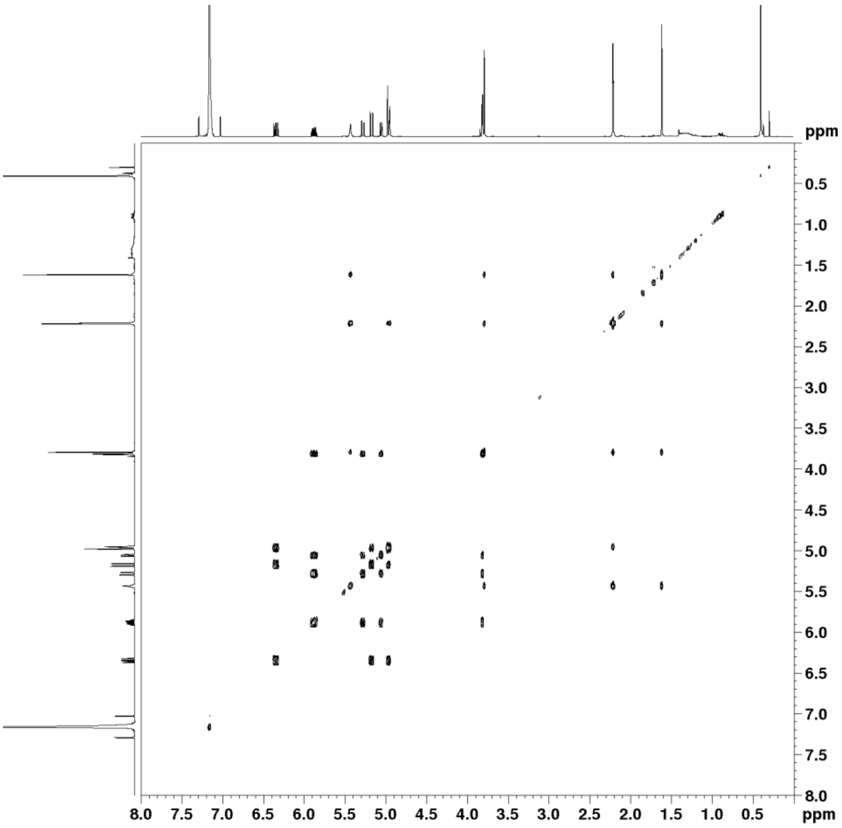

Figure S **106**. ^{1}H-^{1}H COSY NMR spectrum of compound **29** in C_6D_6.

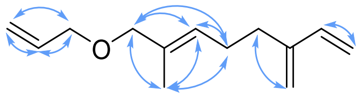

29

Figure S **107**. Key ^{1}H-^{1}H COSY NMR correlations of **29** as indicated by arrows.

Table S **34**. Selected correlations between ^{13}C NMR signals and neighbouring ^{1}H NMR signals as collected from the ^{1}H-^{13}C HMBC spectrum of compound **29**. Note: Signals with weak intensities are given in parentheses.

δ (^{1}H)/ppm	δ (^{13}C)/ppm
6.34	146.2, 116.1, 31.5
5.88	(70.5)
5.45 – 5.42	76.2, 26.6, 14.0
5.28	70.5
5.17	146.2, 139.3
5.07 – 5.04	70.5
4.97 – 4.95	146.2, 139.3, 31.5
3.81	135.3, 115.9, 76.2
3.79	133.2, 127.2, 70.5, 14.0

δ (^{1}H)/ppm	δ (^{13}C)/ppm
2.21	146.2, 139.3, 133.2, 127.2, 116.1
1.61	133.2, 127.2, 76.2

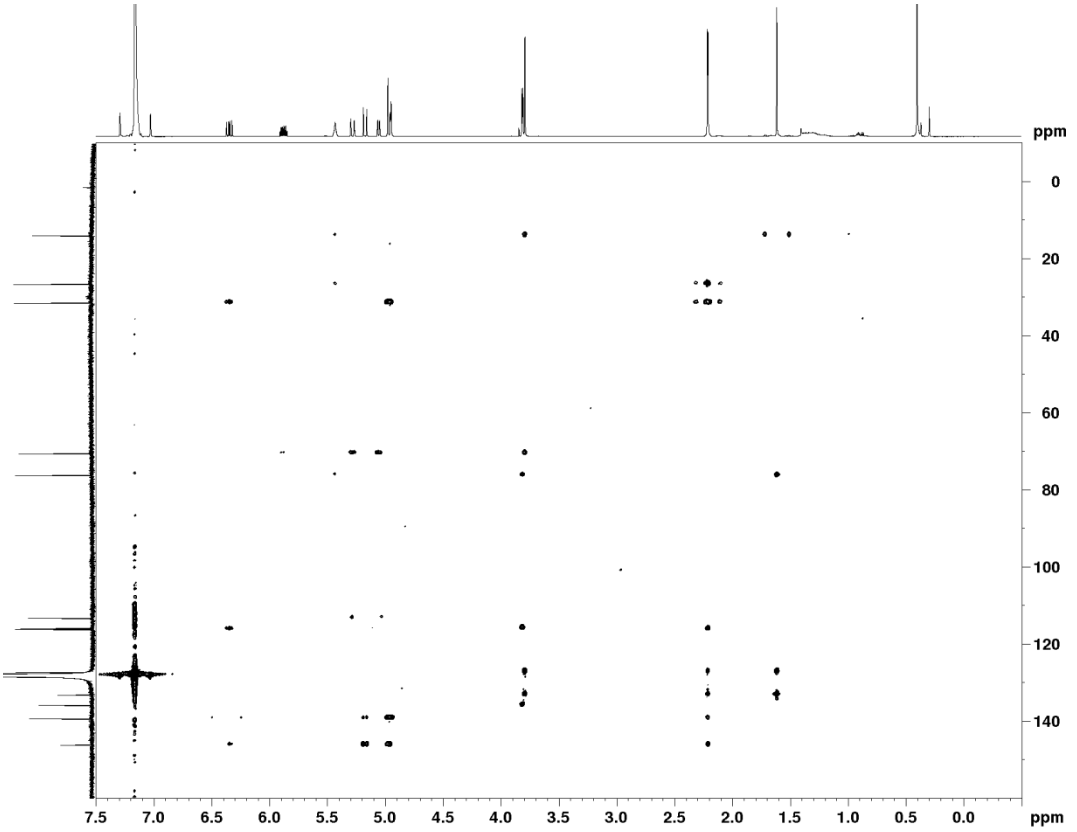

Figure S **108**. ^{1}H-^{13}C HMBC NMR spectrum of compound **29** in C_6D_6.

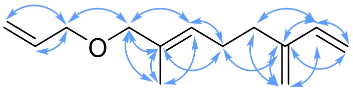

29

Figure S **109**. Key ^{1}H-^{13}C HMBC NMR correlations of **29** as indicated by arrows.

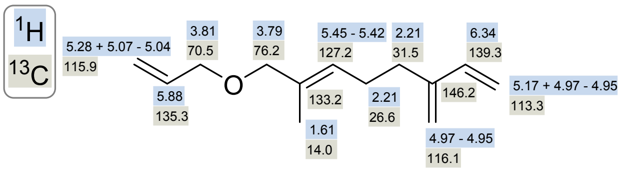

29

Figure S **110**. Full assignment of NMR spectra for **29**.

2 Copies of NMR Spectra

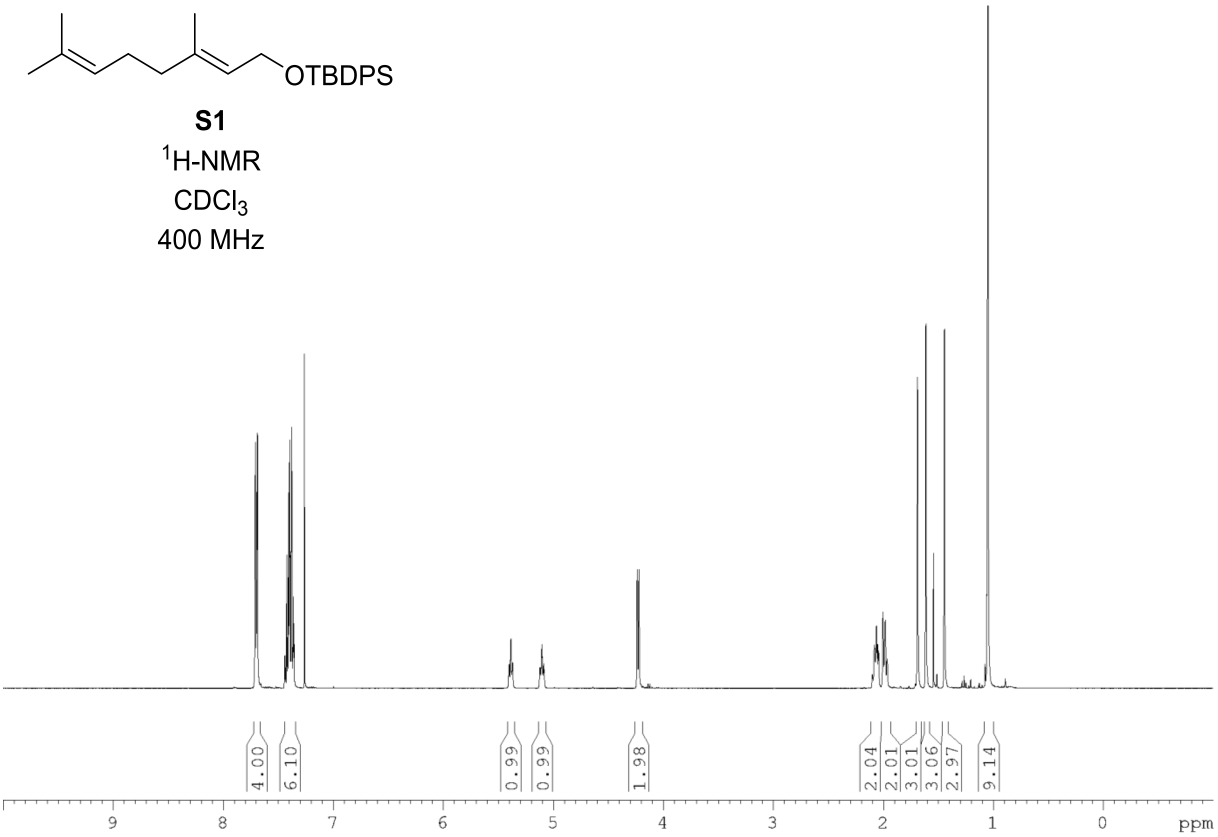

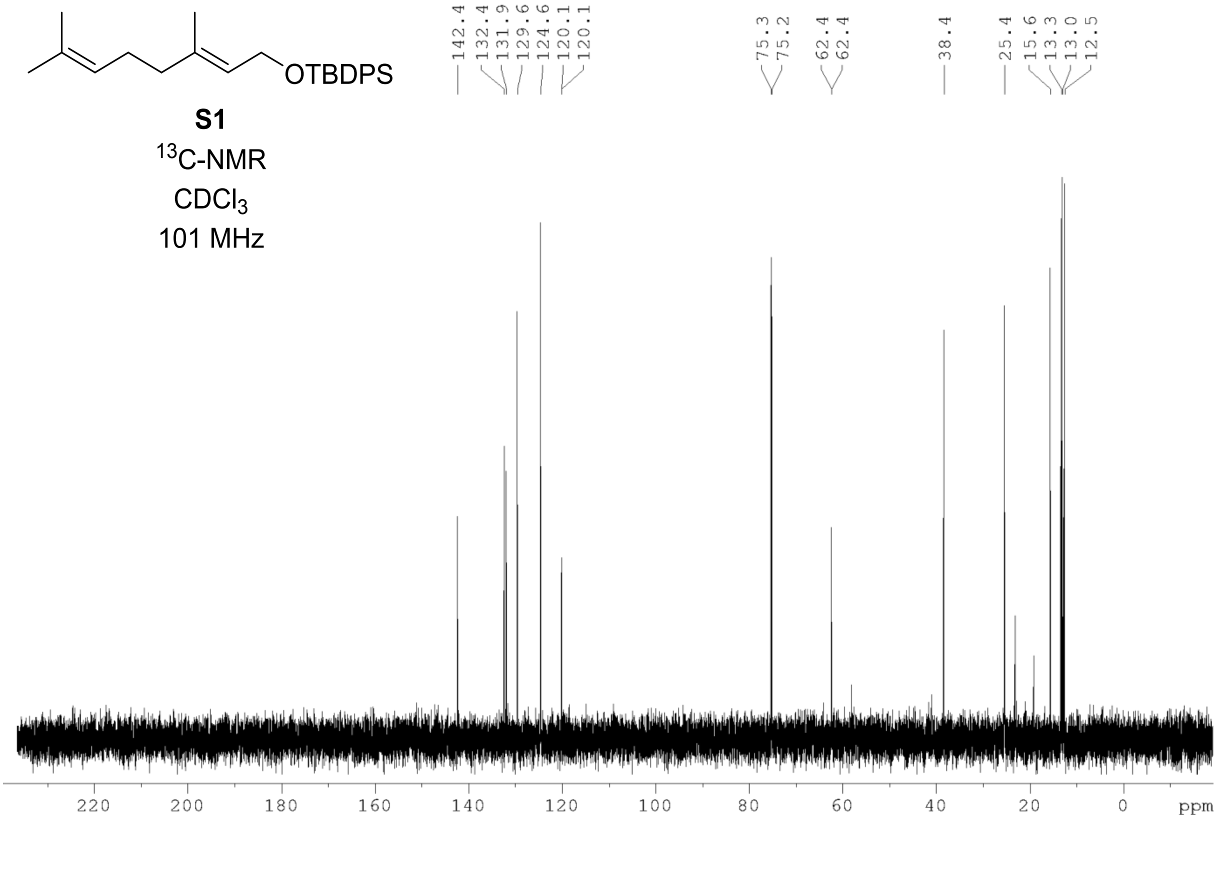

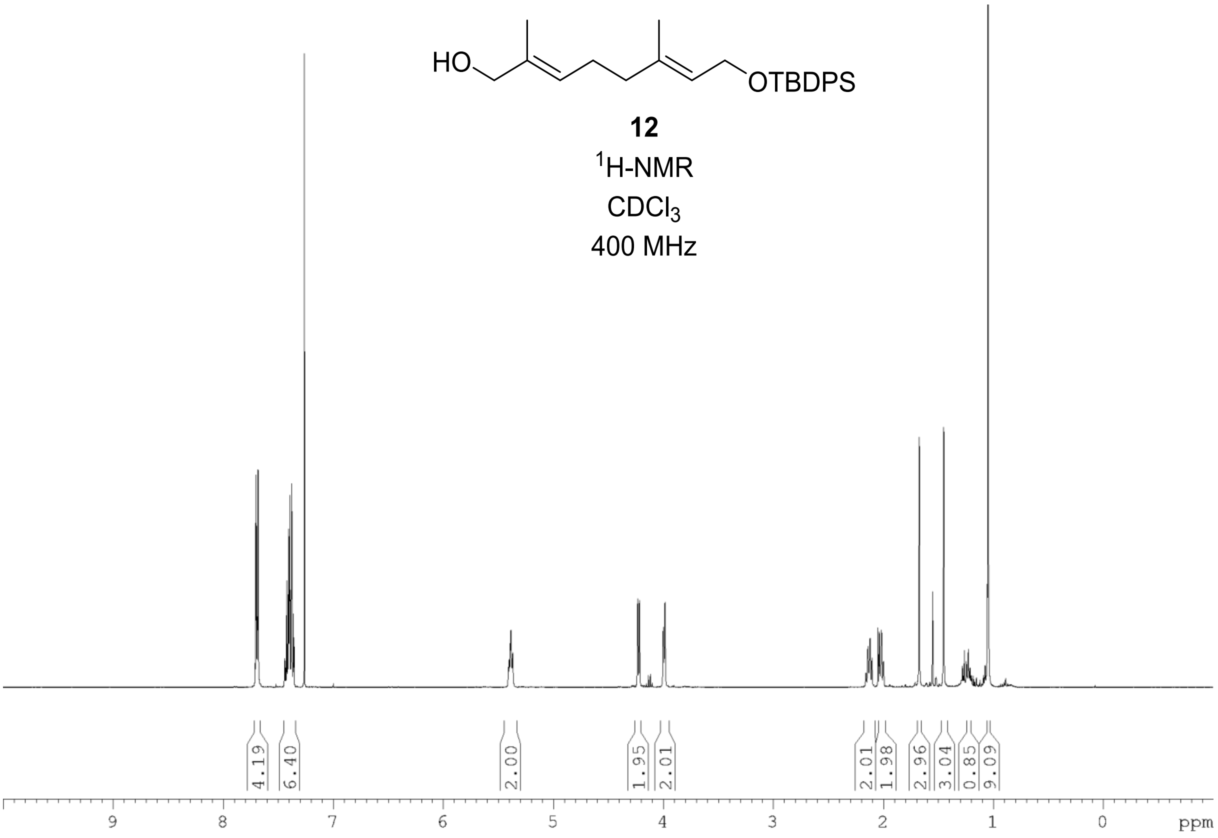
HO
OTBDPS
12
^{1}H-NMR
$CDCl_3$
400 MHz
4.19
6.40
2.00
1.95
2.01
2.01
1.98
2.96
3.04
0.85
9.09
9 8 7 6 5 4 3 2 1 0 ppm

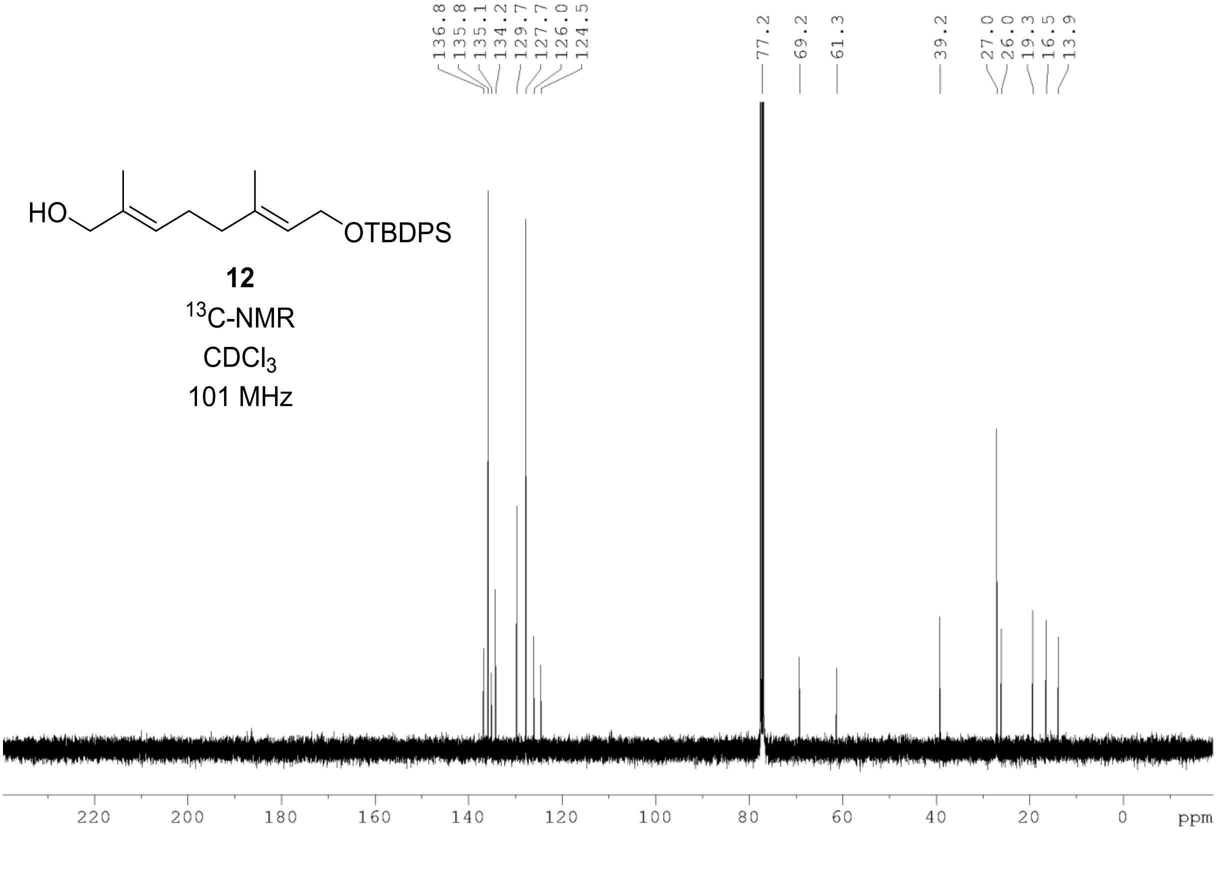
136.8
135.8
135.1
134.2
129.7
127.7
126.0
124.5
77.2
69.2
61.3
39.2
27.0
26.0
19.3
16.5
13.9
HO
OTBDPS
12
^{13}C-NMR
$CDCl_3$
101 MHz
220 200 180 160 140 120 100 80 60 40 20 0 ppm

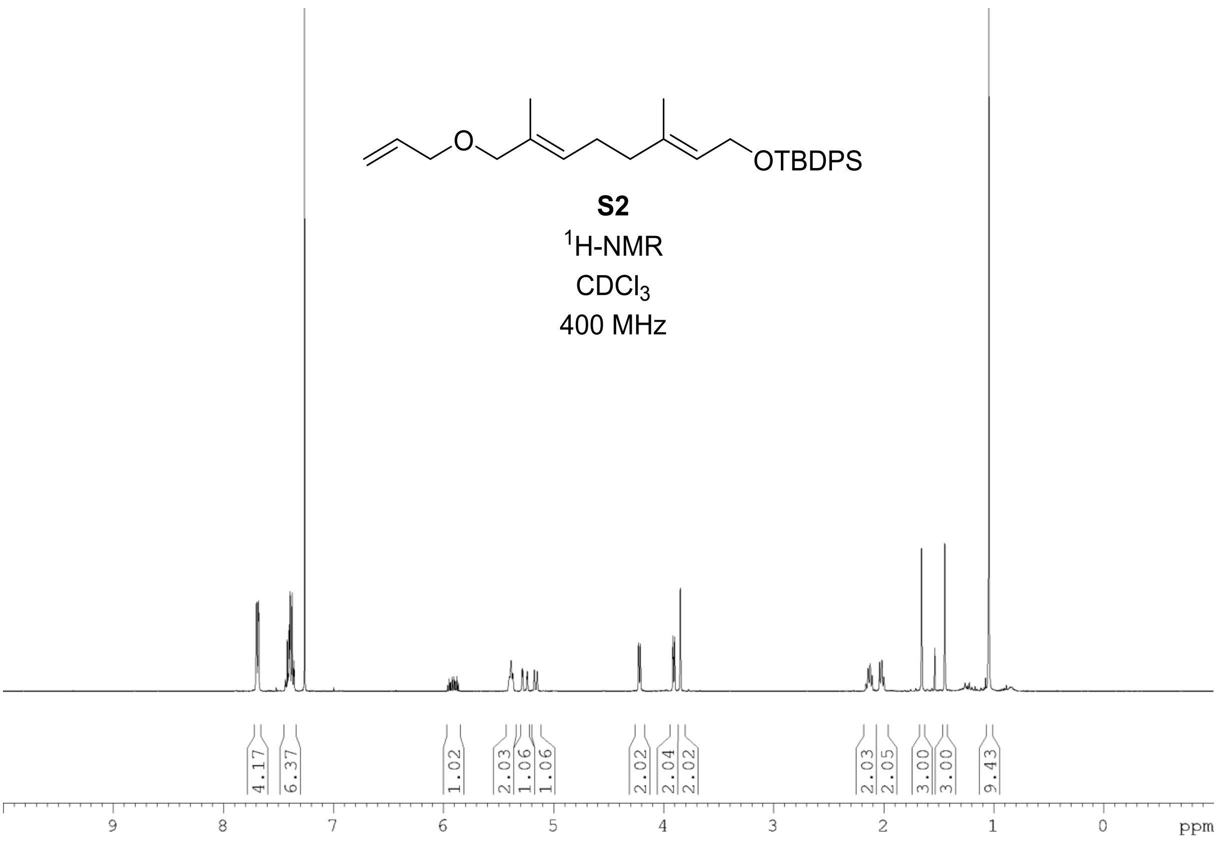
OTBDPS
S2
1H-NMR
CDCl3
400 MHz
4.17
6.37
1.02
2.03
1.06
1.06
2.02
2.04
2.02
2.03
2.05
3.00
3.00
9.43
9 8 7 6 5 4 3 2 1 0 ppm

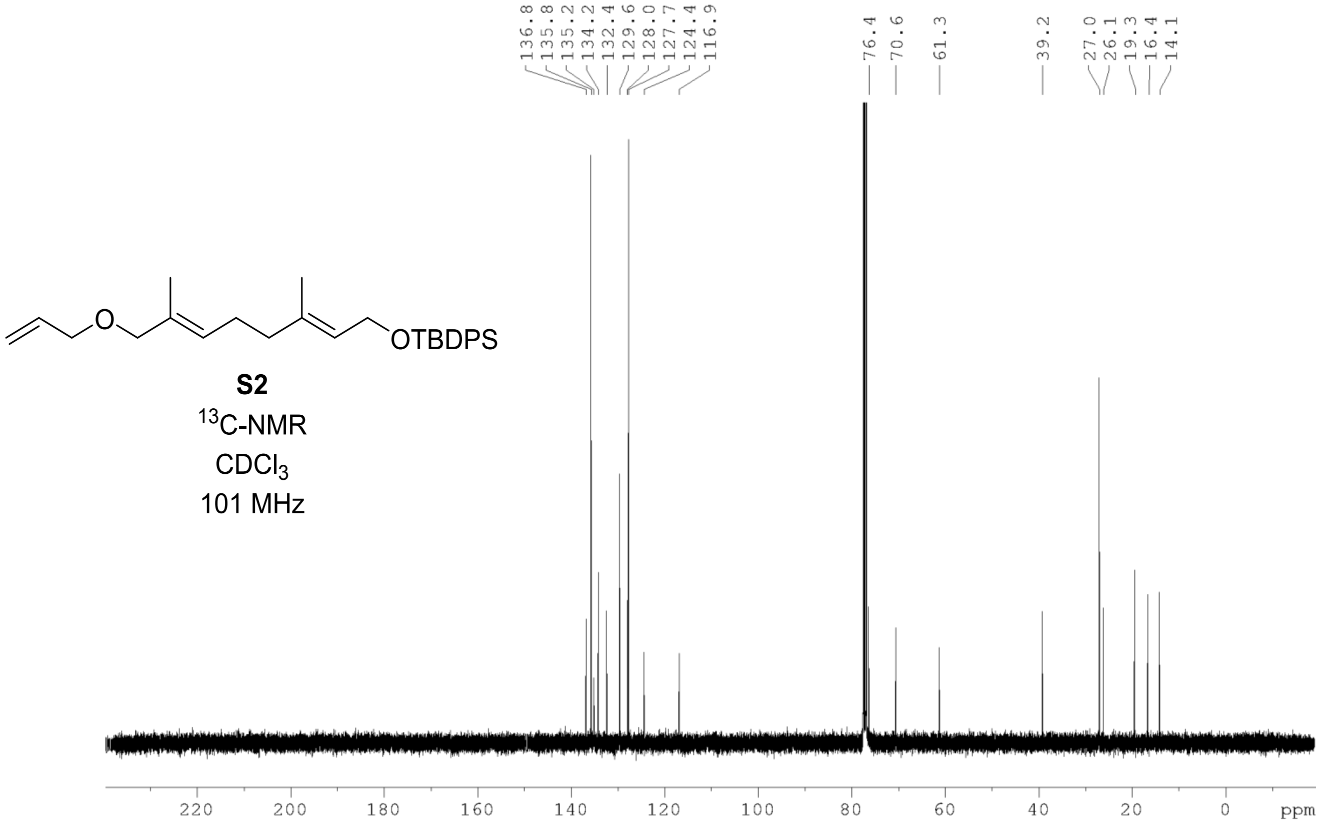
136.8
135.8
135.2
134.2
132.4
129.6
128.0
127.7
124.4
116.9
76.4
70.6
61.3
39.2
27.0
26.1
19.3
16.4
14.1
OTBDPS
S2
13C-NMR
CDCl3
101 MHz
220 200 180 160 140 120 100 80 60 40 20 0 ppm

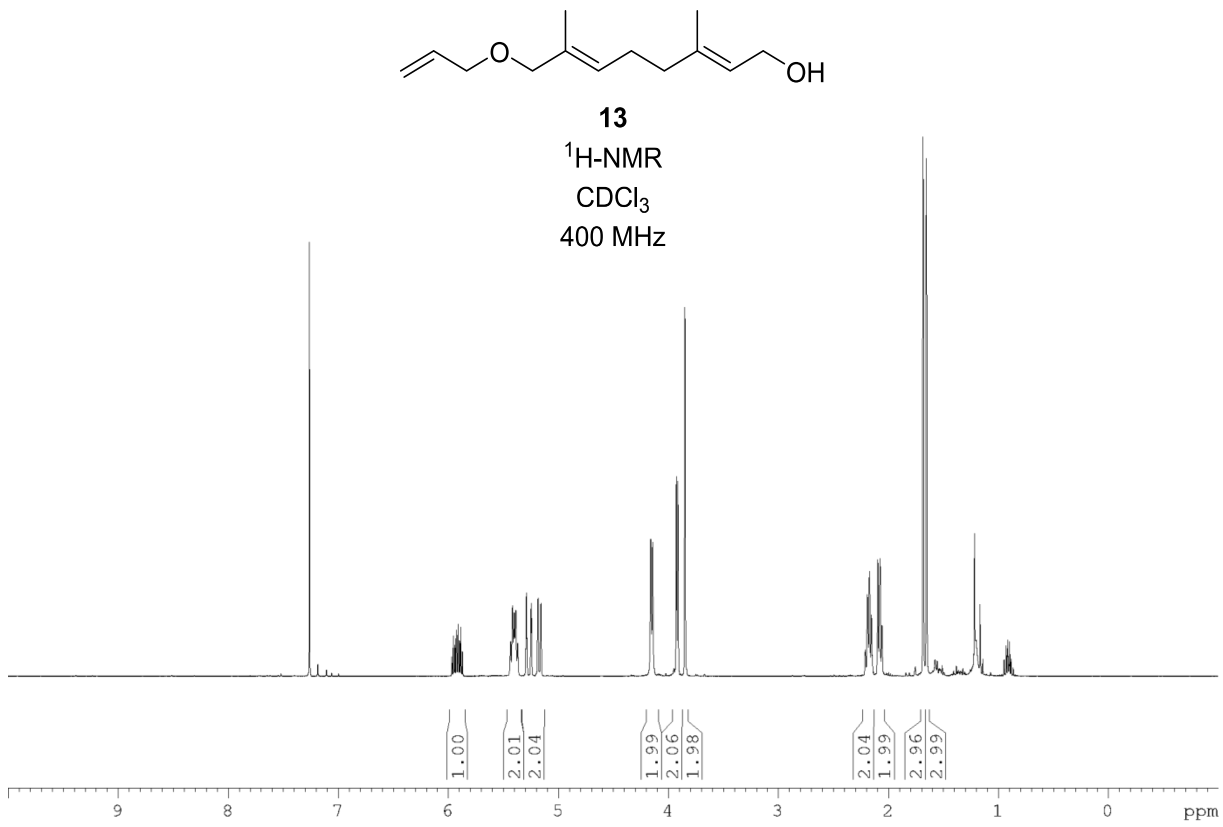
OH
O
13
1H-NMR
CDCl3
400 MHz
1.00
2.01
2.04
1.99
2.06
1.98
2.04
1.99
2.96
2.99
9
8
7
6
5
4
3
2
1
0
ppm

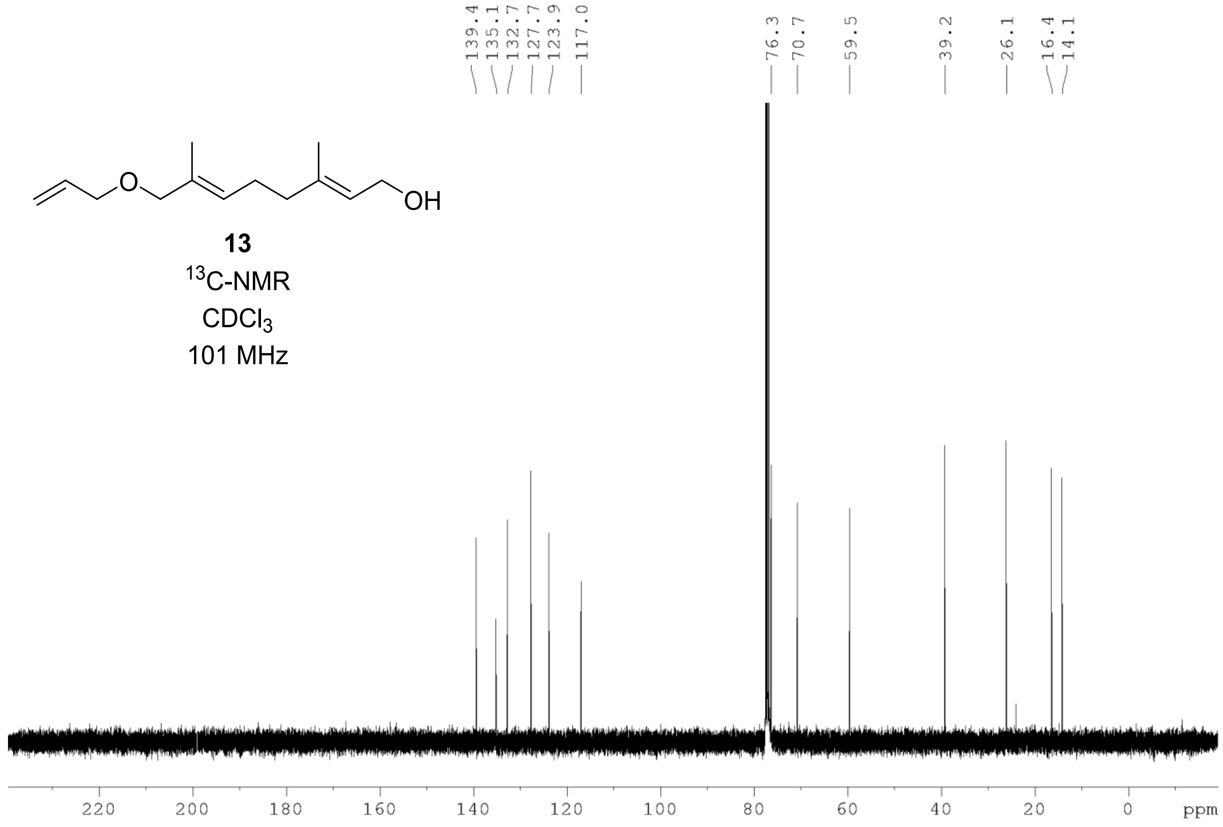
139.4
135.1
132.7
127.7
123.9
117.0
76.3
70.7
59.5
39.2
26.1
16.4
14.1
O
OH
13
13C-NMR
CDCl3
101 MHz
220
200
180
160
140
120
100
80
60
40
20
0
ppm

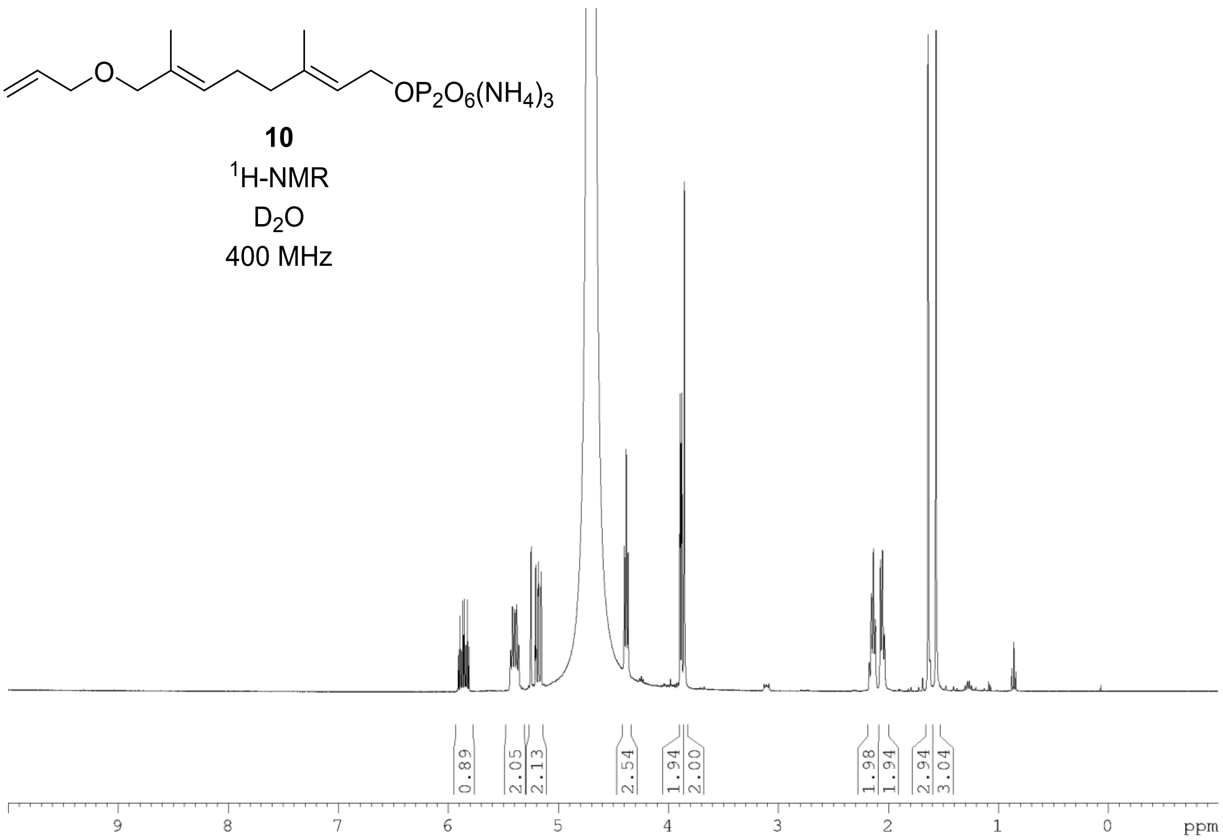
OP2O6(NH4)3
10
1H-NMR
D2O
400 MHz
0.89
2.05
2.13
2.54
1.94
2.00
1.98
1.94
2.94
3.04
9
8
7
6
5
4
3
2
1
0
ppm

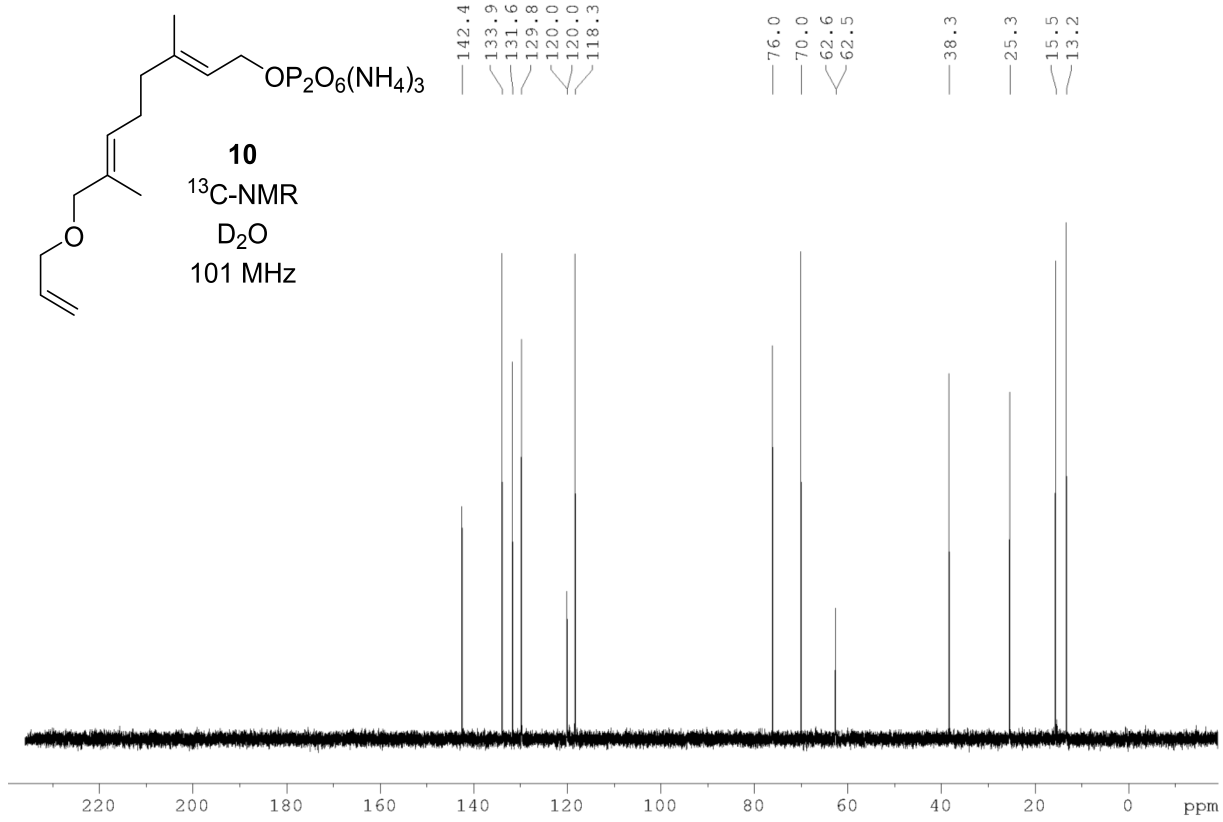
142.4
133.9
131.6
129.8
120.0
120.0
118.3
76.0
70.0
62.6
62.5
38.3
25.3
15.5
13.2
OP2O6(NH4)3
O
10
13C-NMR
D2O
101 MHz
220
200
180
160
140
120
100
80
60
40
20
0
ppm

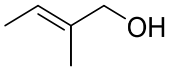

S3
^{1}H-NMR
$CDCl_3$
400 MHz

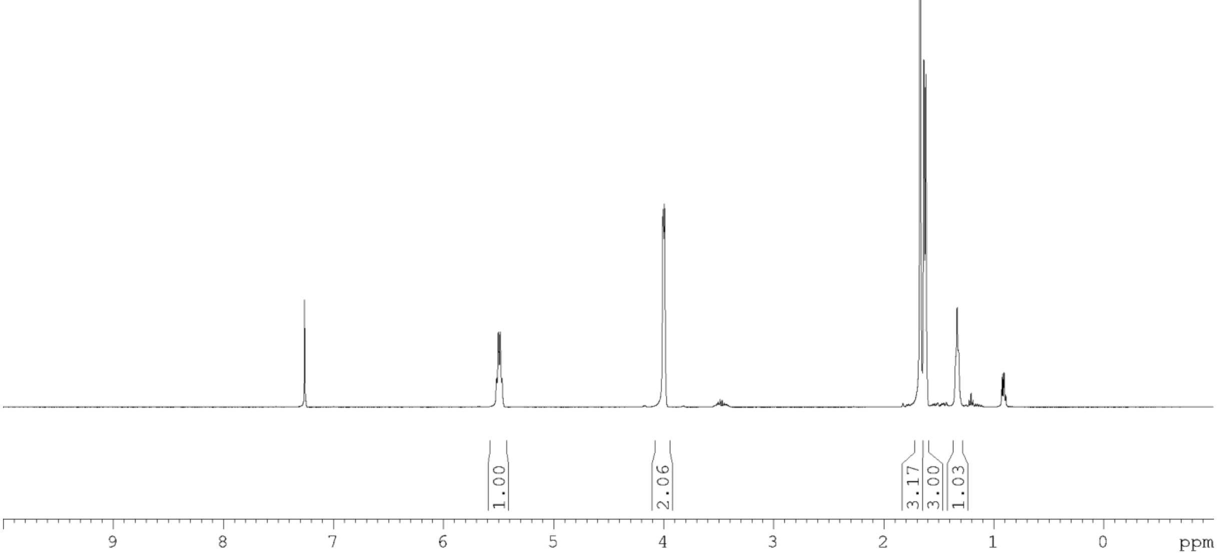

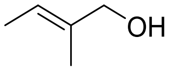

S3
^{13}C-NMR
$CDCl_3$
101 MHz

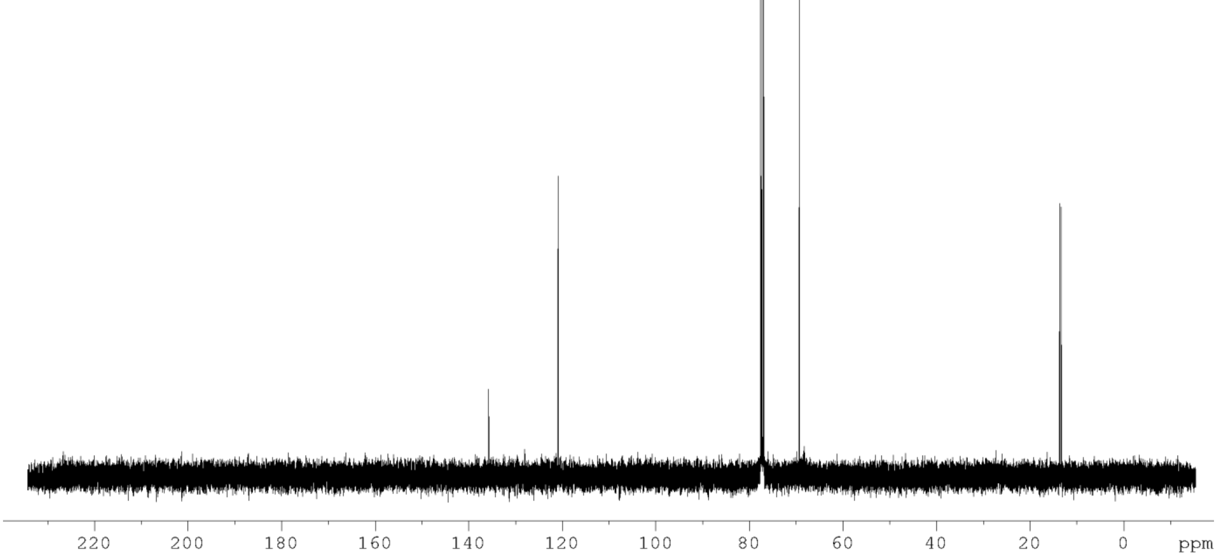

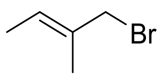

S4
^{1}H-NMR
$CDCl_3$
400 MHz

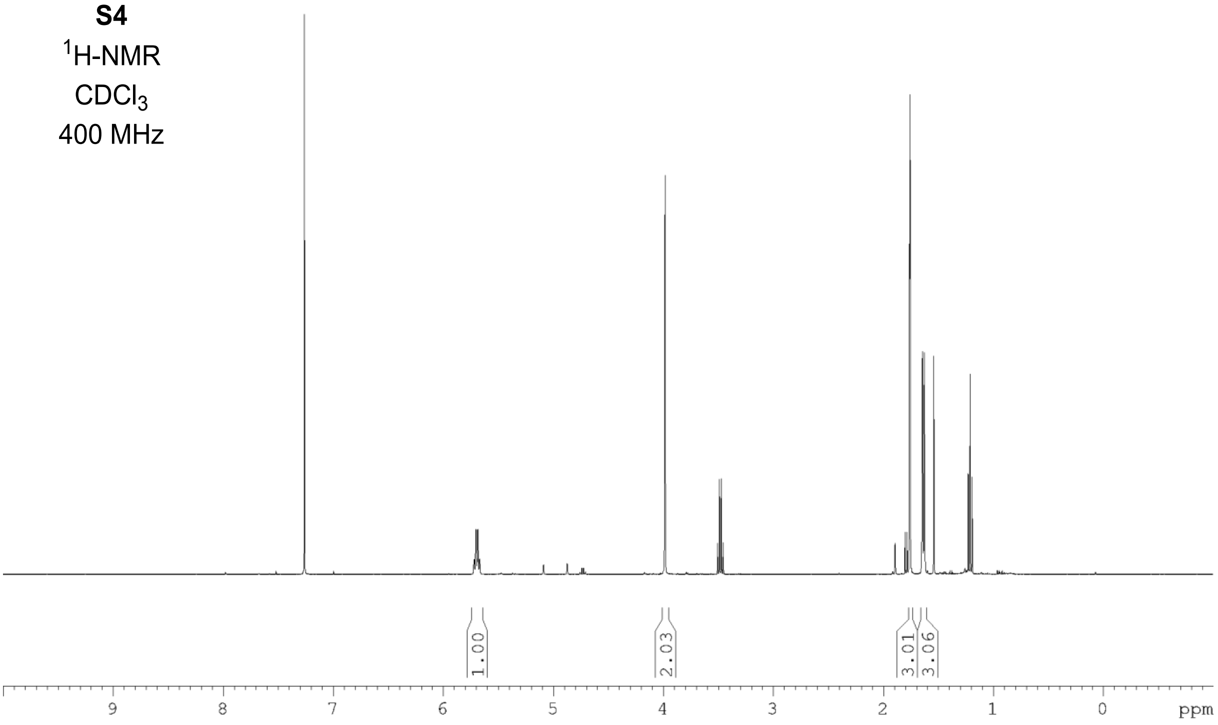

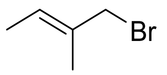

S4
^{13}C-NMR
$CDCl_3$
101 MHz

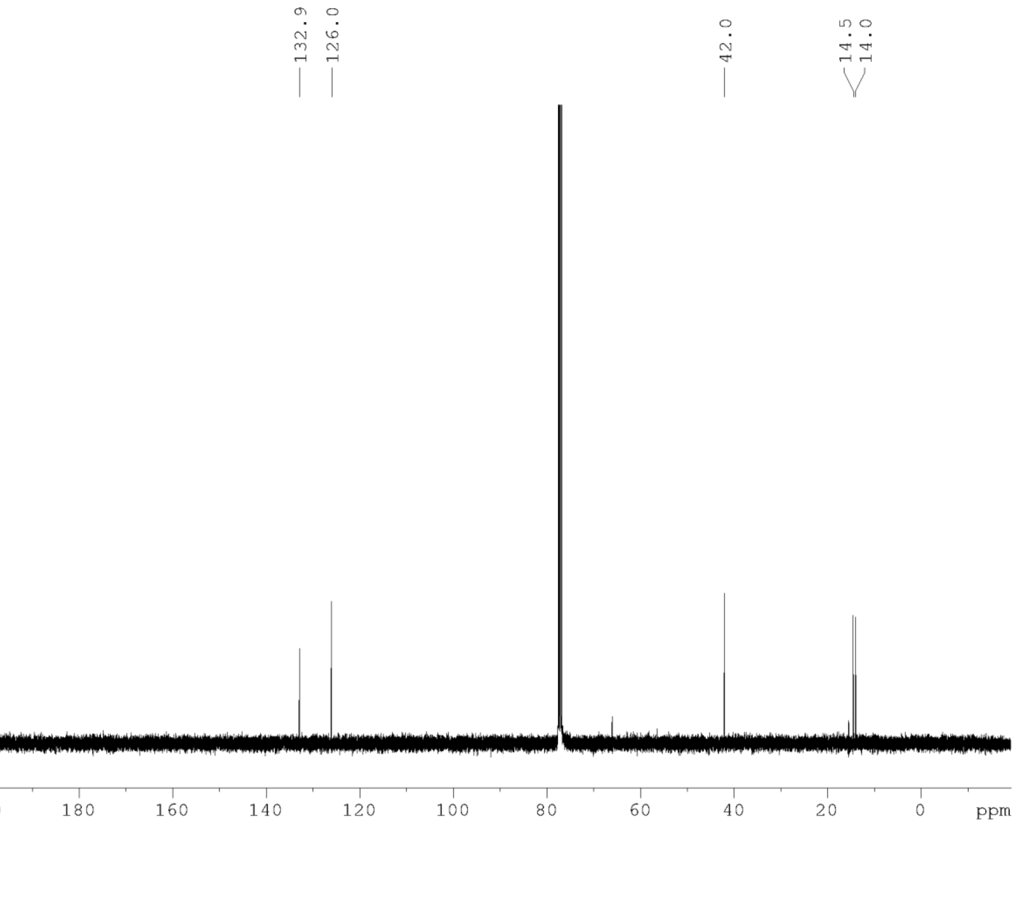

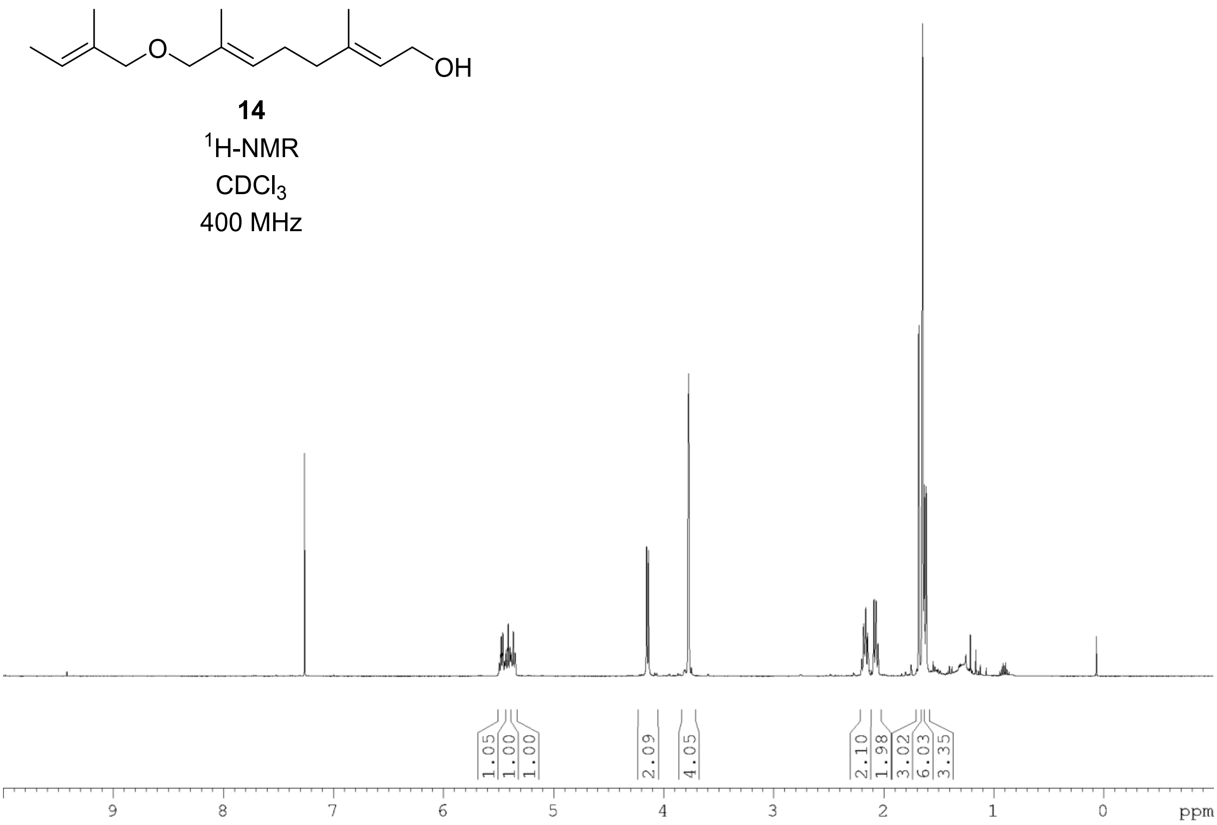
O
OH
14
^{1}H-NMR
$CDCl_3$
400 MHz
1.05
1.00
1.00
2.09
4.05
2.10
1.98
3.02
6.03
3.35
9 8 7 6 5 4 3 2 1 0 ppm

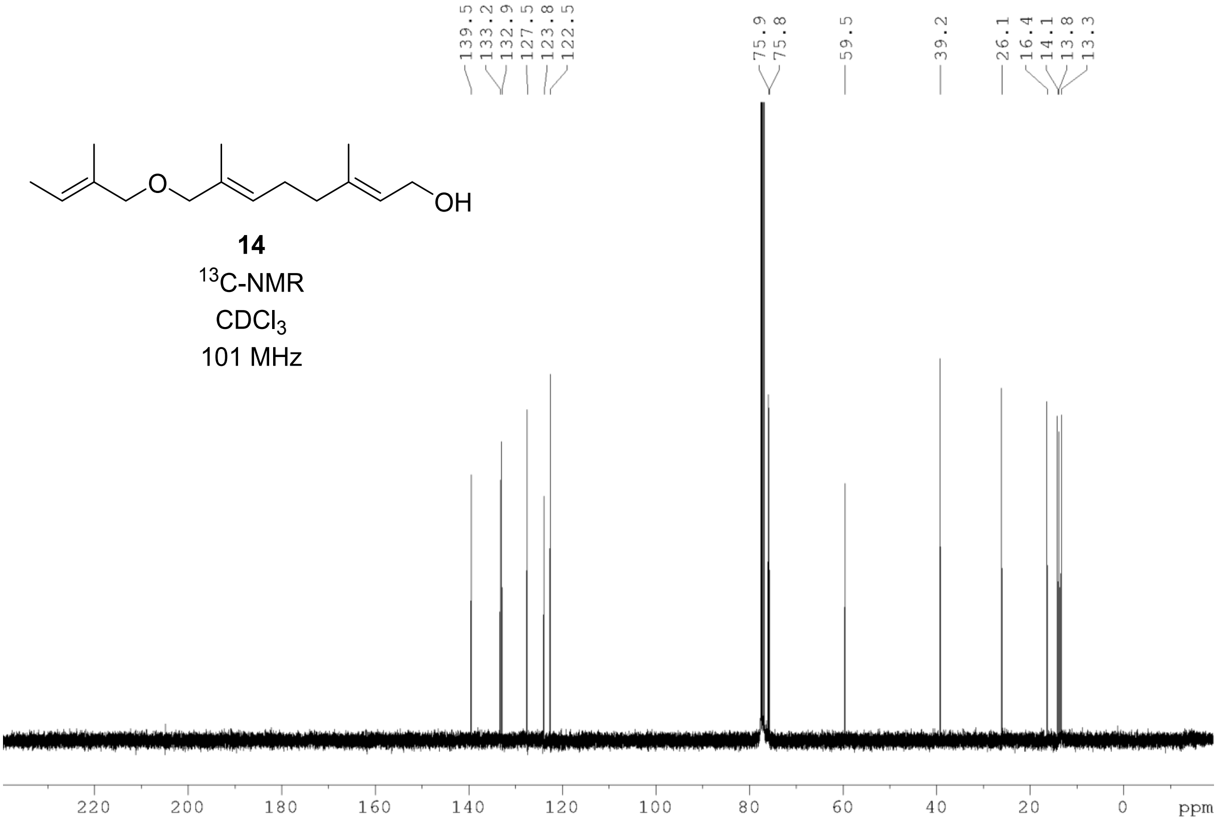
139.5
133.2
132.9
127.5
123.8
122.5
75.9
75.8
59.5
39.2
26.1
16.4
14.1
13.8
13.3
O
OH
14
^{13}C-NMR
$CDCl_3$
101 MHz
220 200 180 160 140 120 100 80 60 40 20 0 ppm

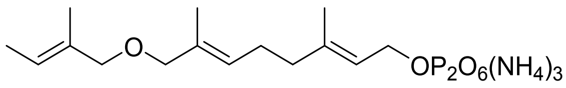

9
^{1}H-NMR
D_2O
400 MHz

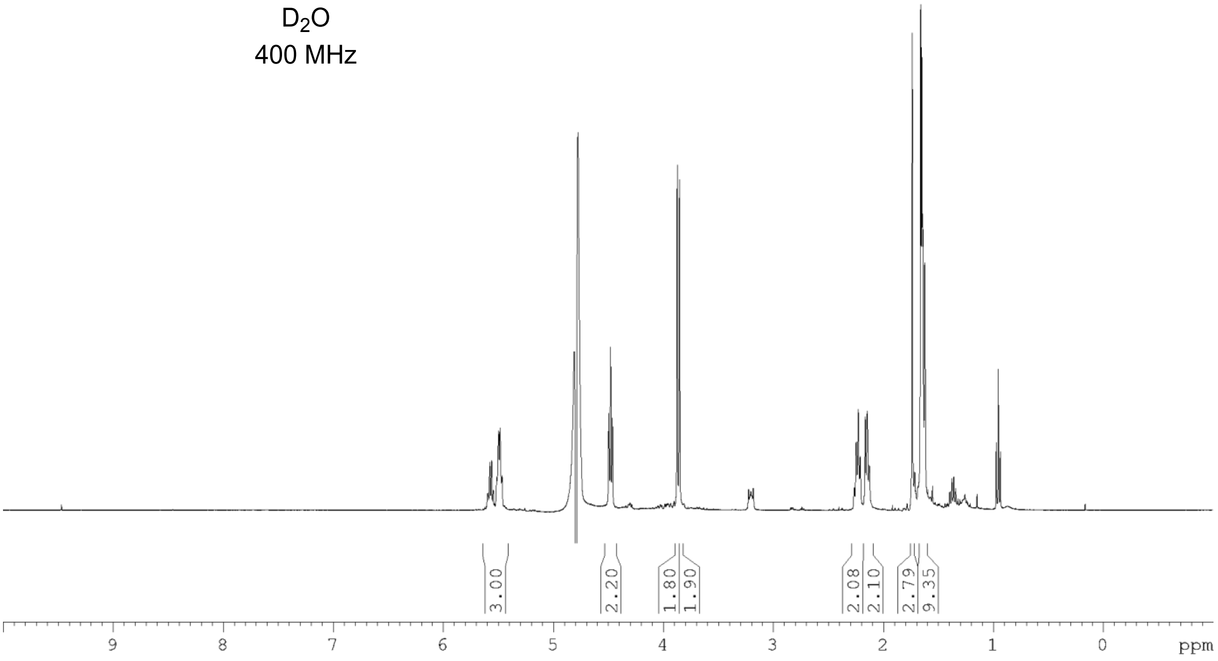

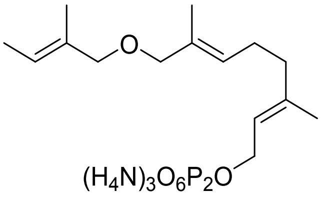

9
^{13}C-NMR
D_2O
151 MHz

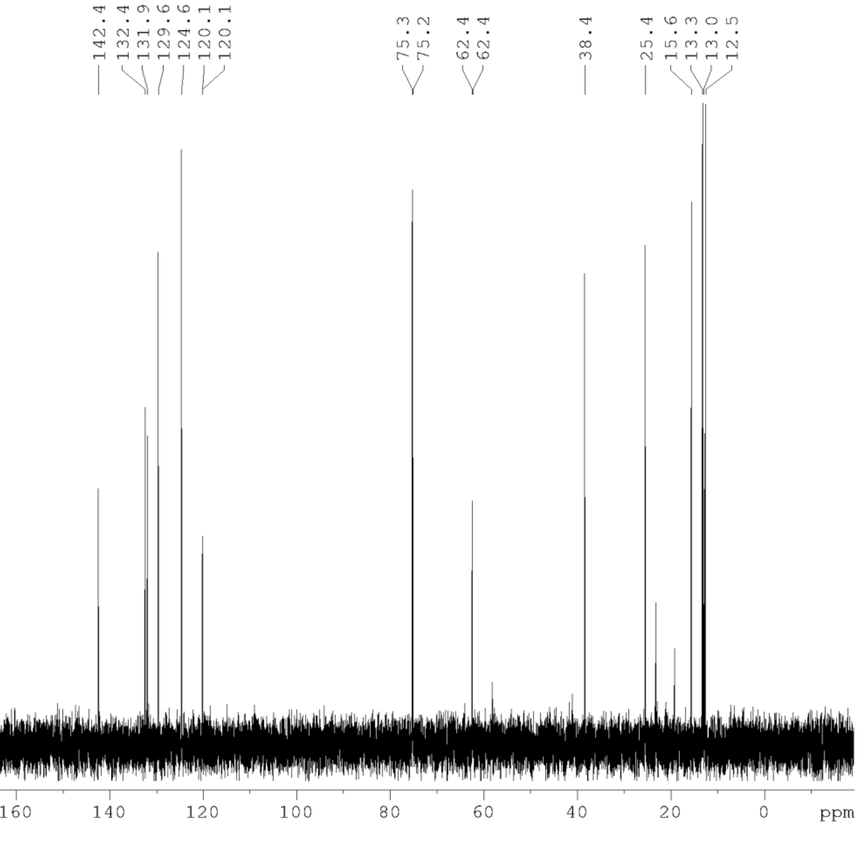

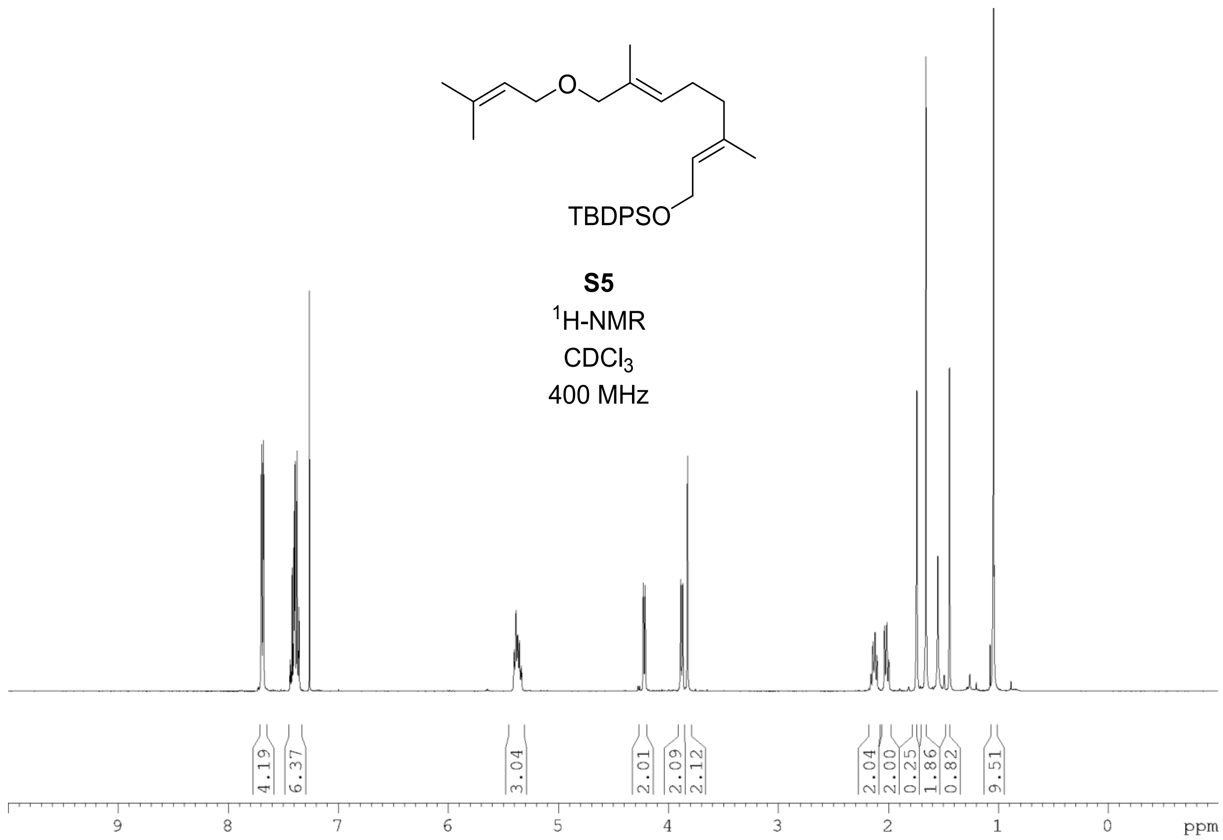
O
TBDPSO
S5
1H-NMR
CDCl3
400 MHz
4.19
6.37
3.04
2.01
2.09
2.12
2.04
2.00
0.25
1.86
0.82
9.51
9 8 7 6 5 4 3 2 1 0 ppm

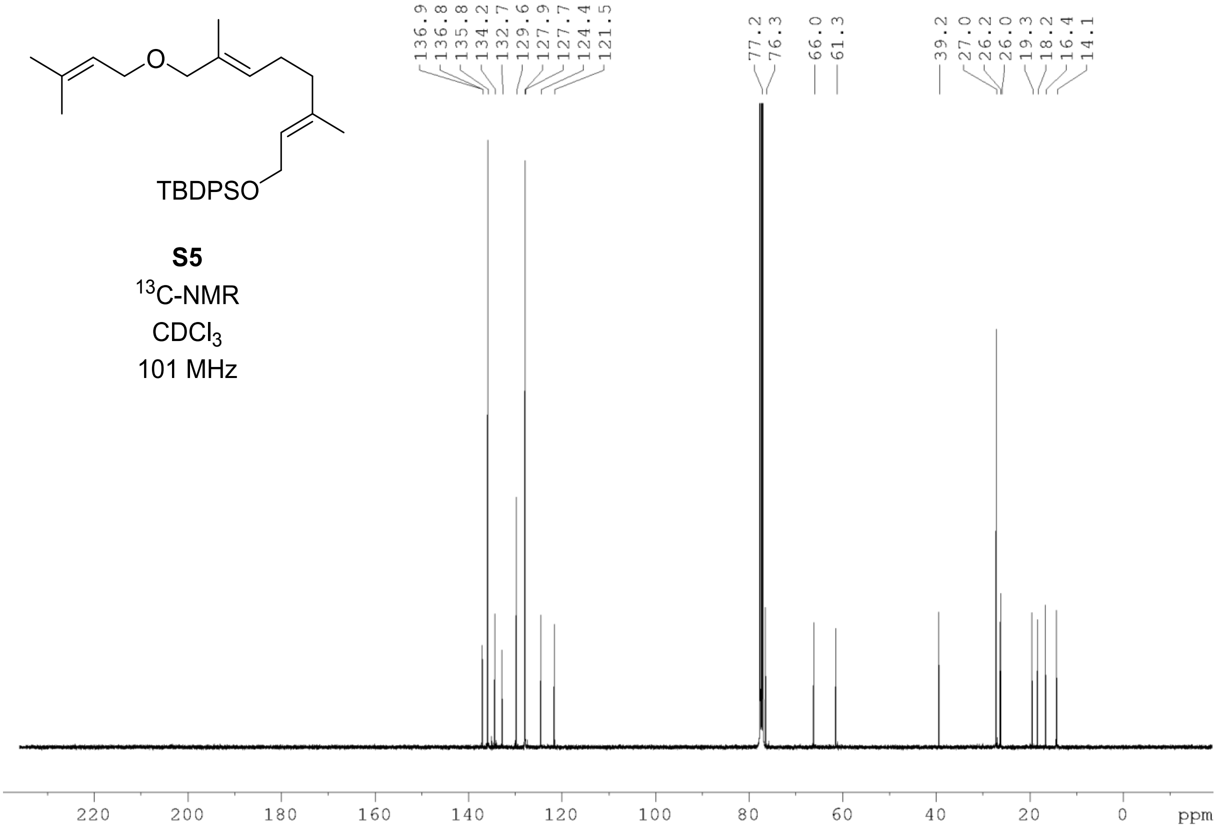
136.9
136.8
135.8
134.2
132.7
129.6
127.9
127.7
124.4
121.5
77.2
76.3
66.0
61.3
39.2
27.0
26.2
26.0
19.3
18.2
16.4
14.1
O
TBDPSO
S5
13C-NMR
CDCl3
101 MHz
220 200 180 160 140 120 100 80 60 40 20 0 ppm

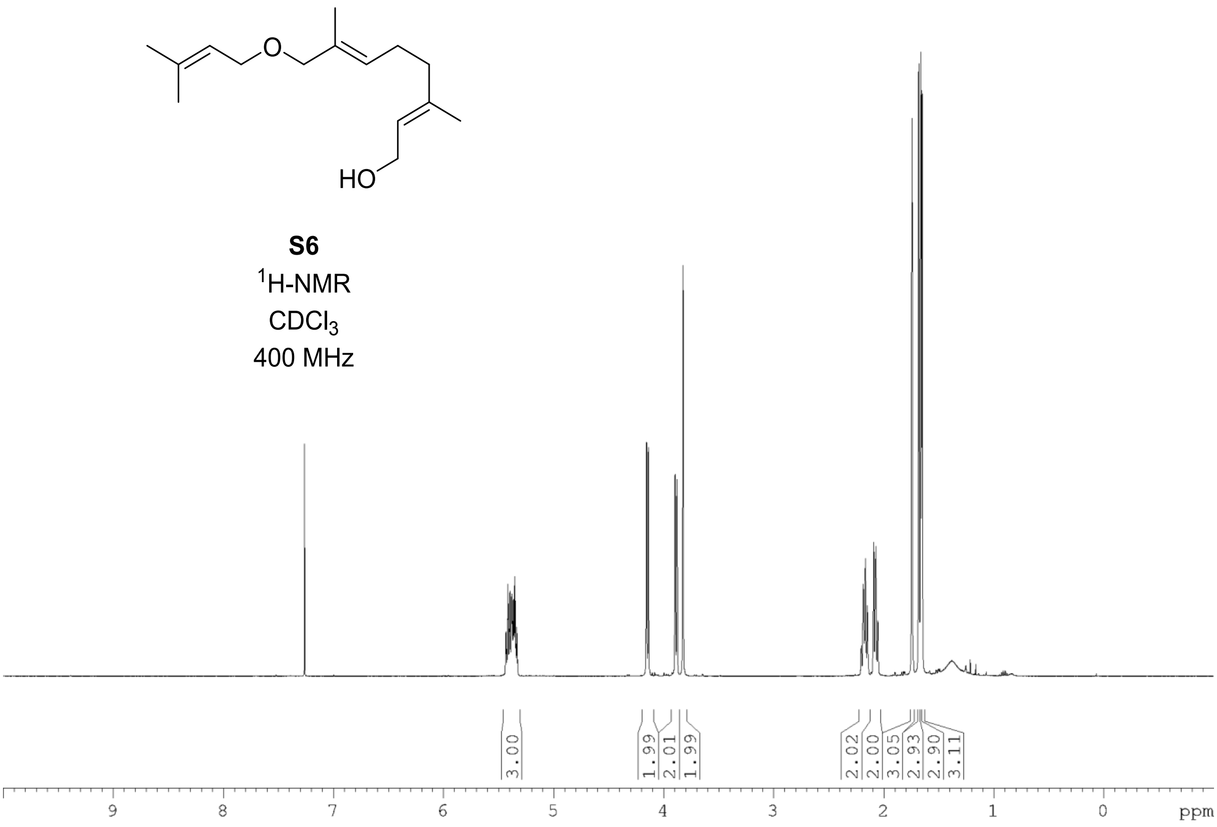
HO
O
S6
1H-NMR
CDCl3
400 MHz
3.00
1.99
2.01
1.99
2.02
2.00
3.05
2.93
2.90
3.11
9
8
7
6
5
4
3
2
1
0
ppm

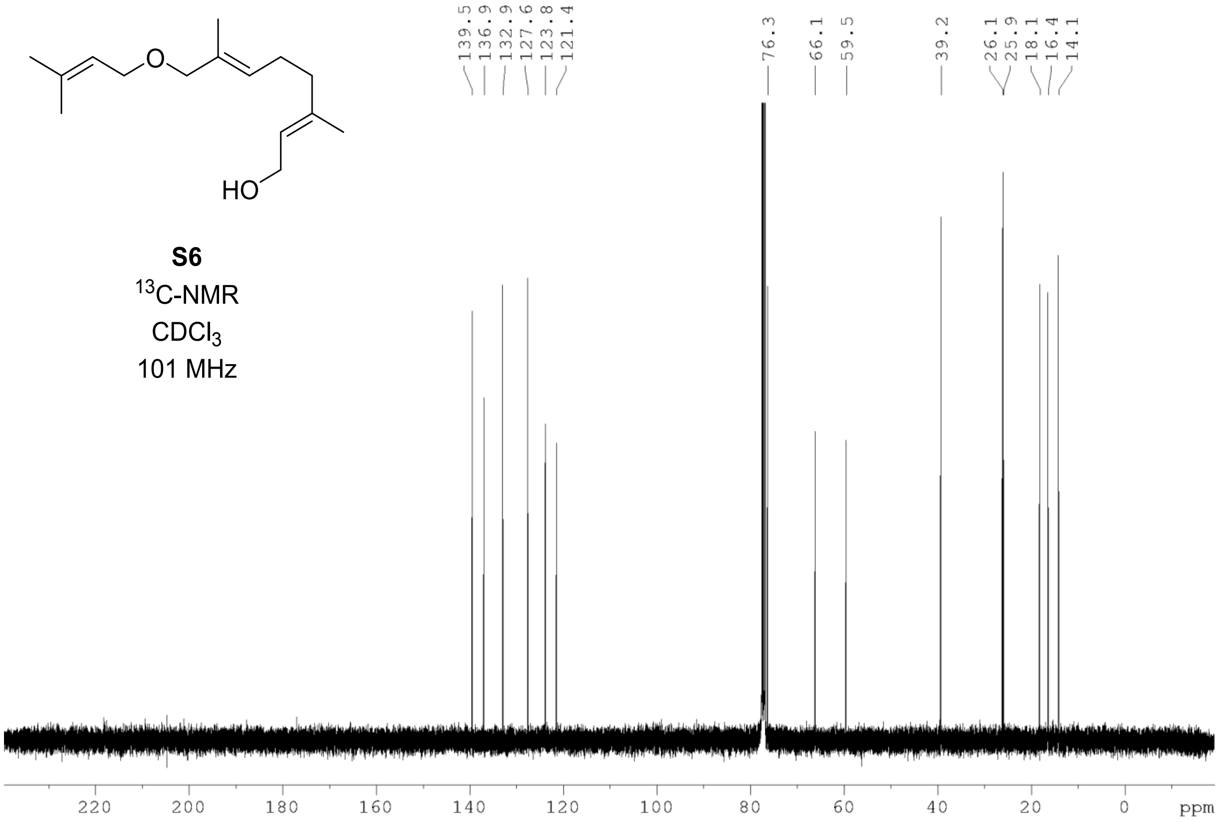
HO
O
S6
13C-NMR
CDCl3
101 MHz
139.5
136.9
132.9
127.6
123.8
121.4
76.3
66.1
59.5
39.2
26.1
25.9
18.1
16.4
14.1
220
200
180
160
140
120
100
80
60
40
20
0
ppm

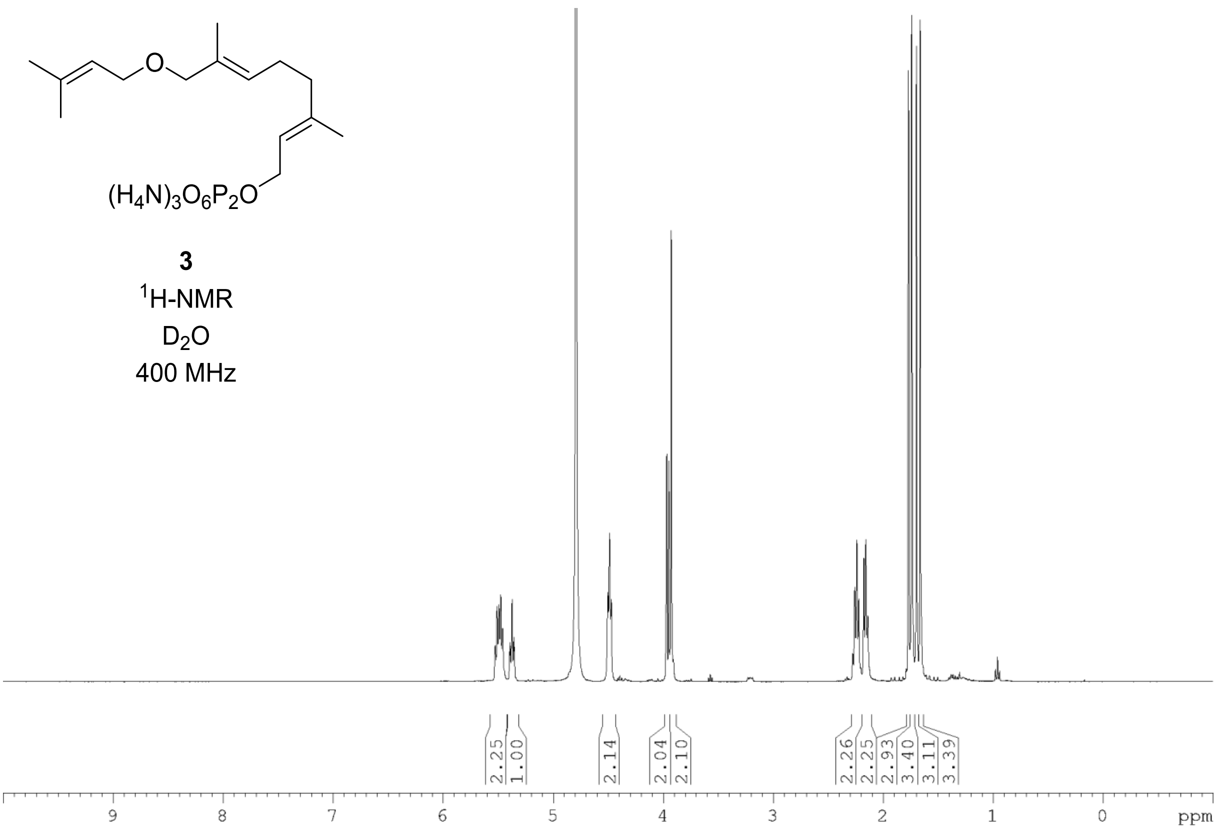

3
^{1}H-NMR
D_2O
400 MHz

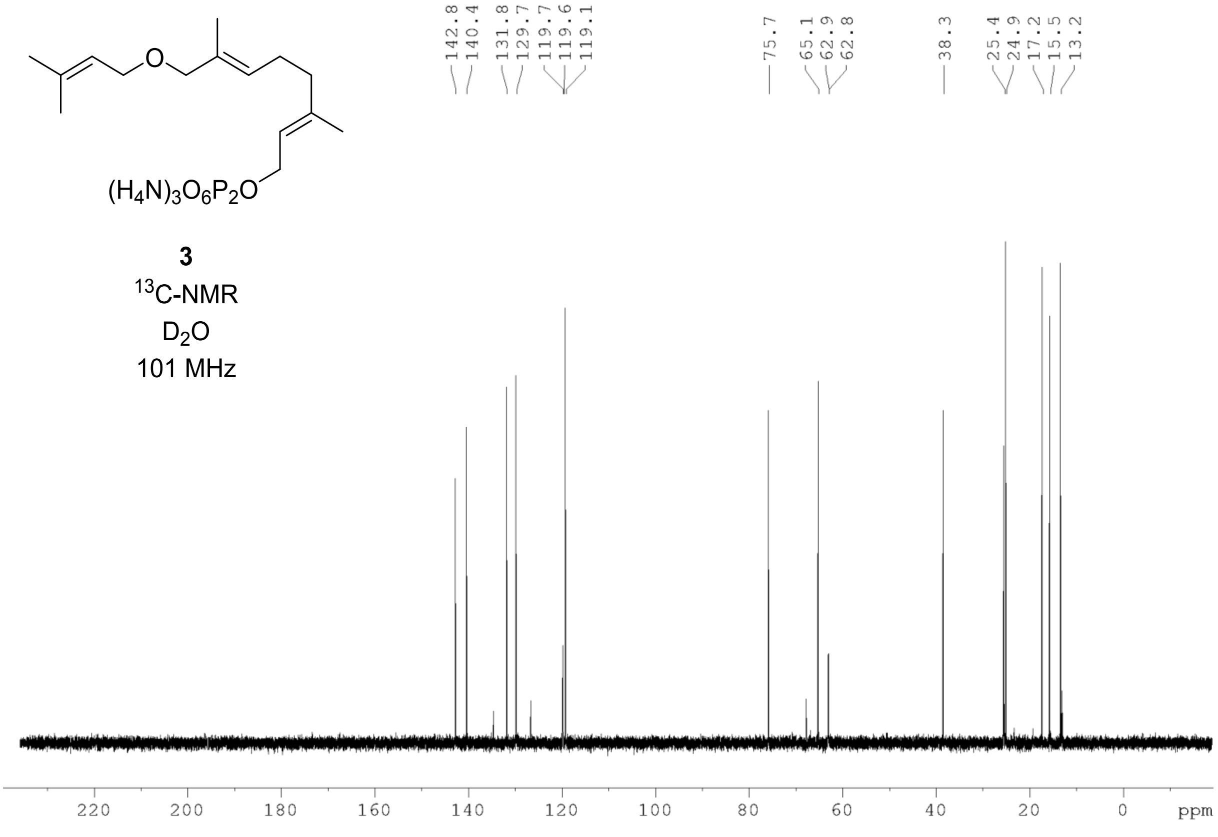

3
^{13}C-NMR
D_2O
101 MHz

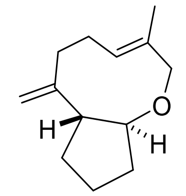

18
^{1}H-NMR
C_6D_6
600 MHz

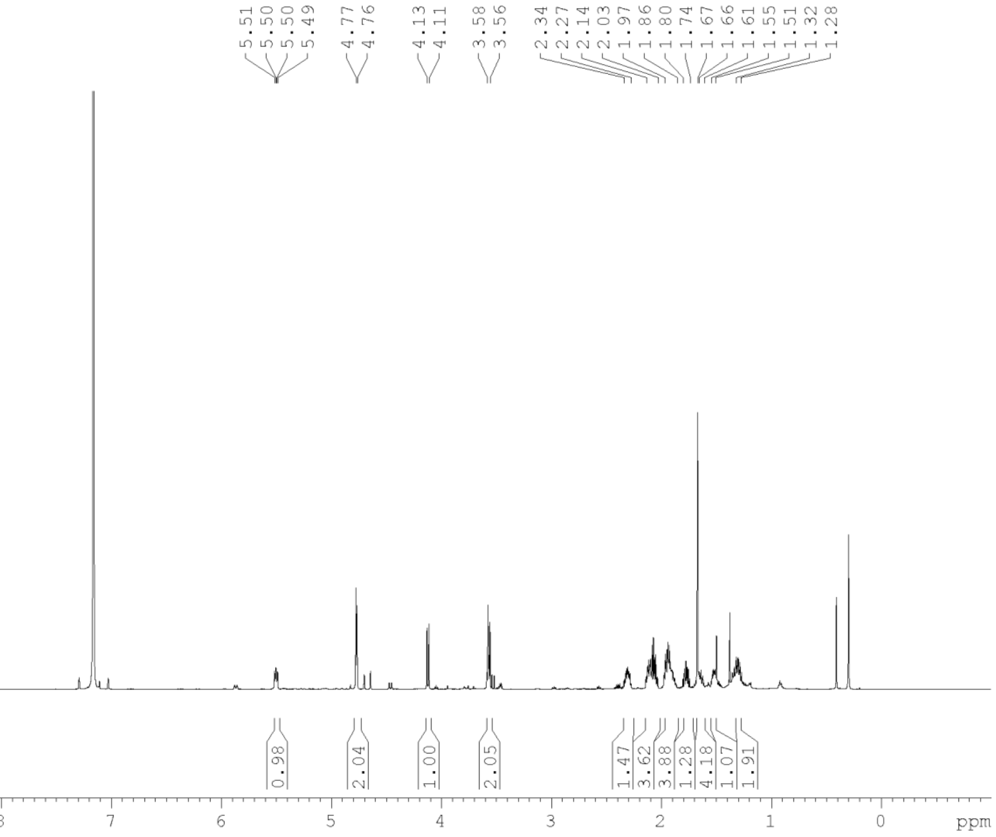

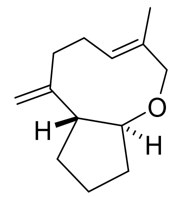

18
^{13}C-NMR
C_6D_6
151 MHz

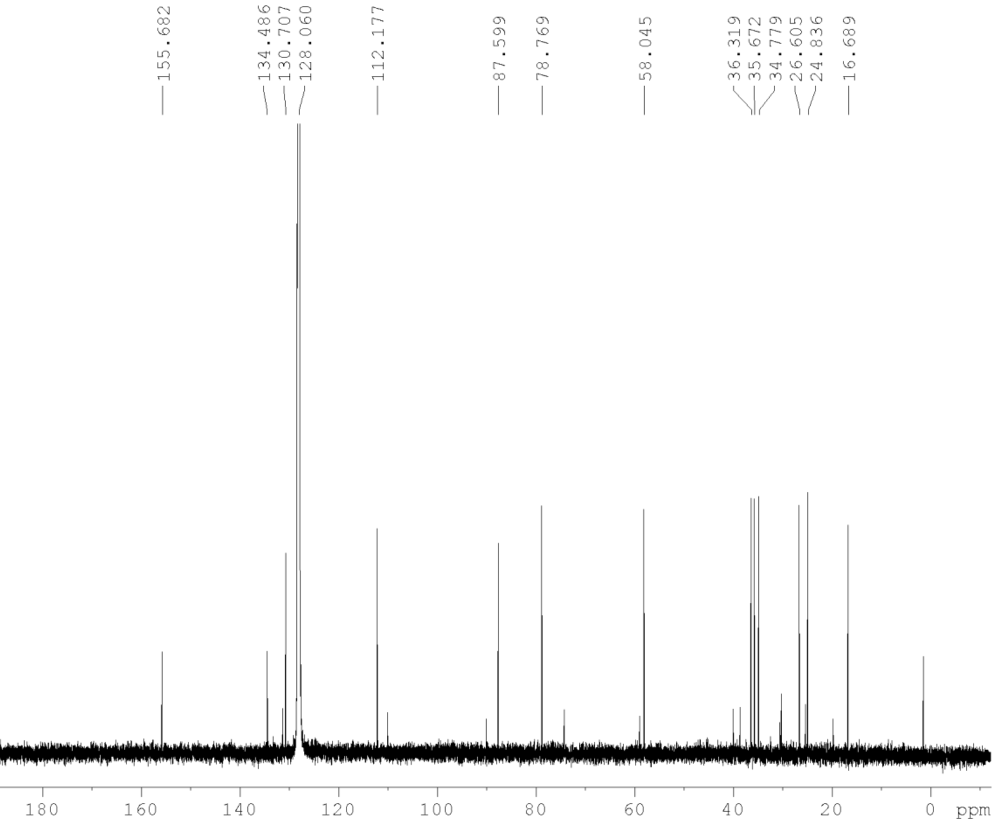

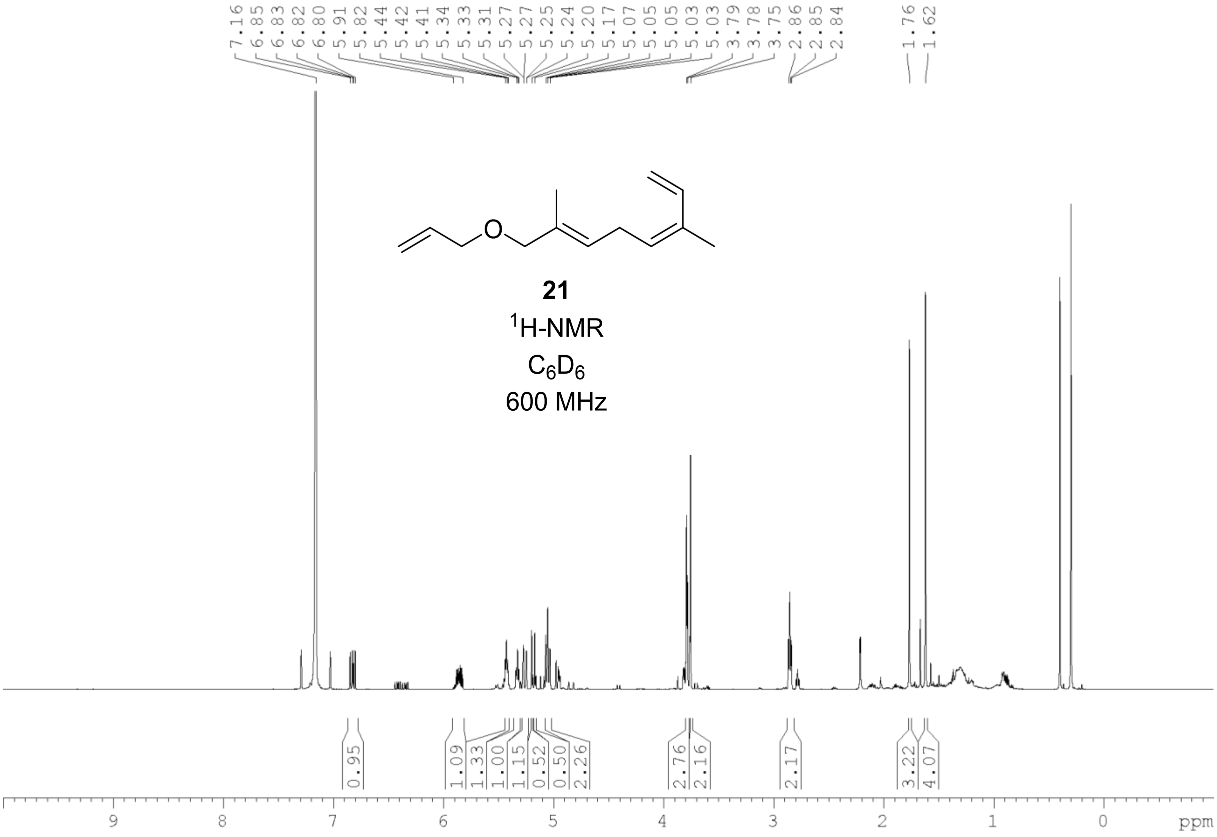
7.16
6.85
6.83
6.82
6.80
5.91
5.82
5.44
5.42
5.41
5.34
5.33
5.31
5.27
5.27
5.25
5.24
5.20
5.17
5.07
5.05
5.05
5.03
3.79
3.78
3.75
2.86
2.85
2.84
1.76
1.62
O
21
^{1}H-NMR
C_6D_6
600 MHz
0.95
1.09
1.33
1.00
1.15
0.52
0.50
2.26
2.76
2.16
2.17
3.22
4.07
9 8 7 6 5 4 3 2 1 0 ppm

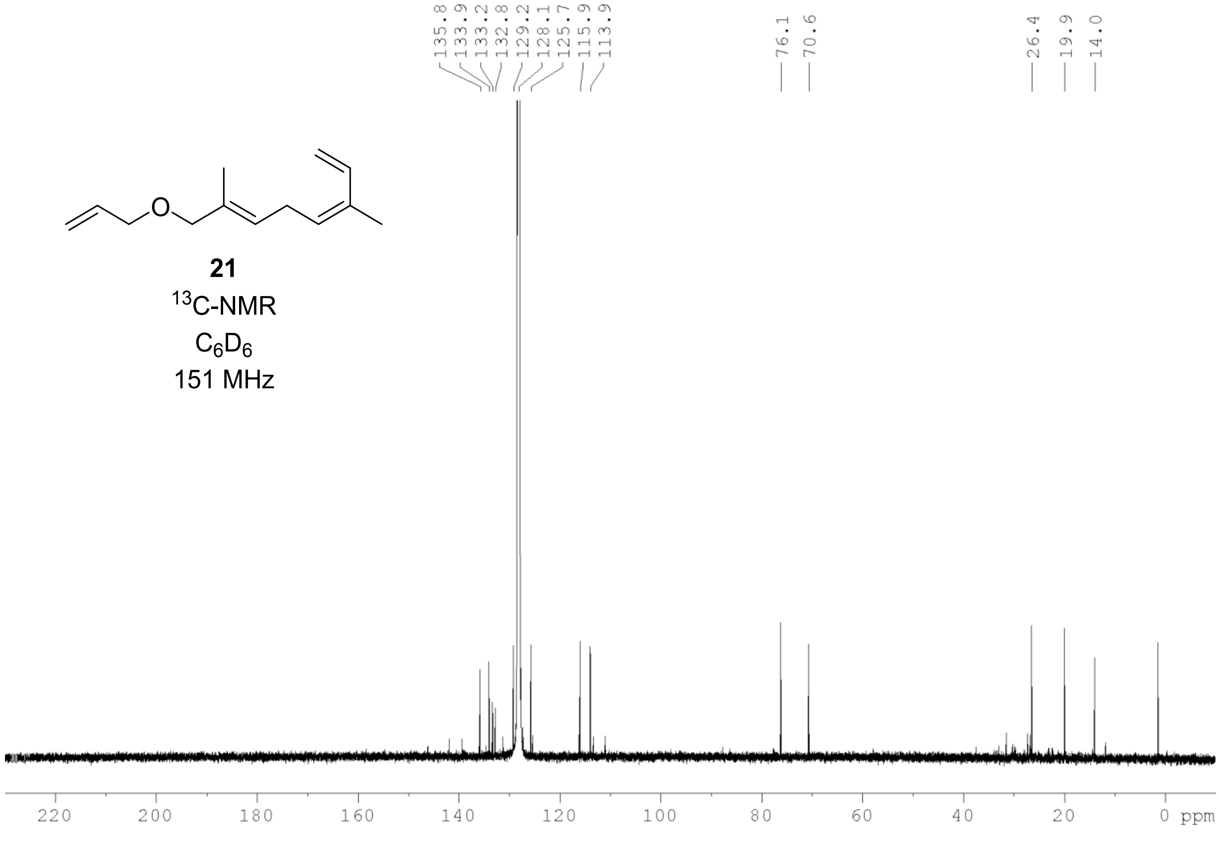
135.8
133.9
133.2
132.8
129.2
128.1
125.7
115.9
113.9
76.1
70.6
26.4
19.9
14.0
O
21
^{13}C-NMR
C_6D_6
151 MHz
220 200 180 160 140 120 100 80 60 40 20 0 ppm

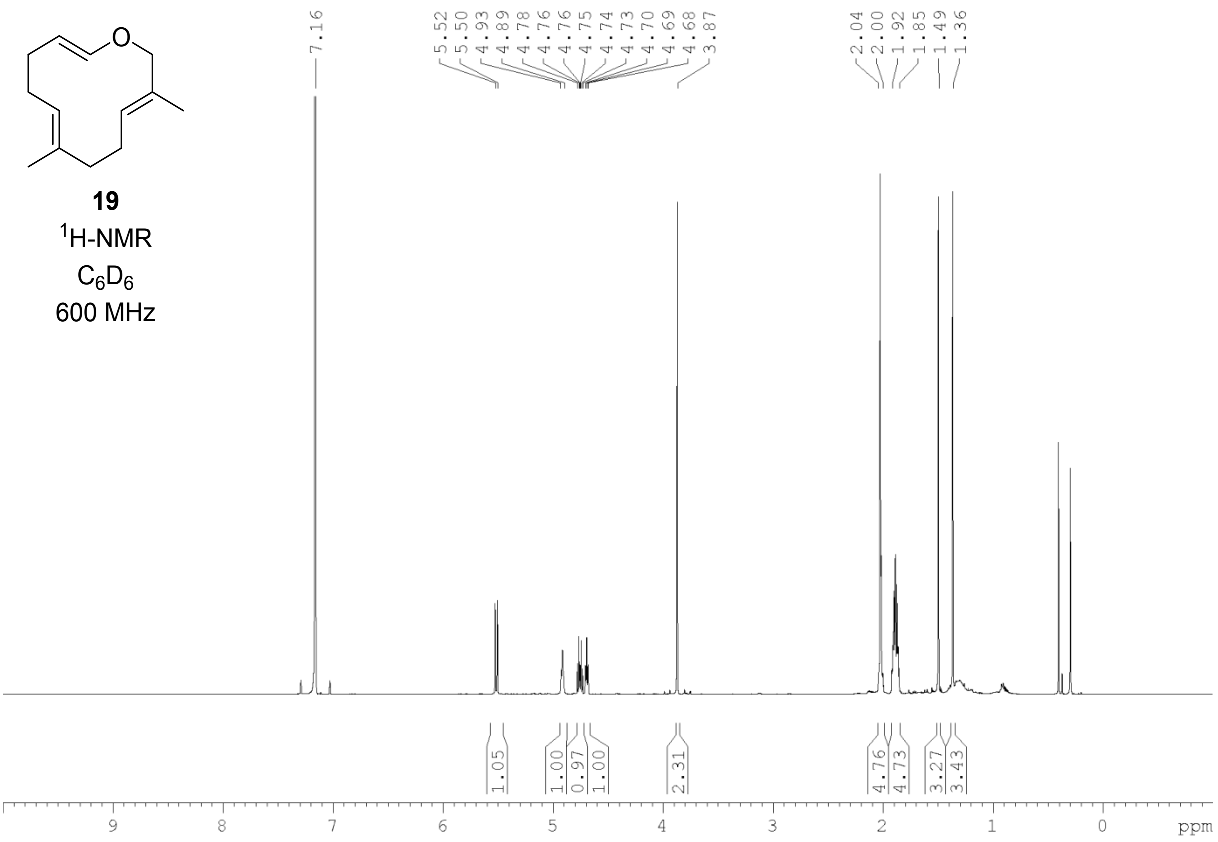

19
^{1}H-NMR
C_6D_6
600 MHz

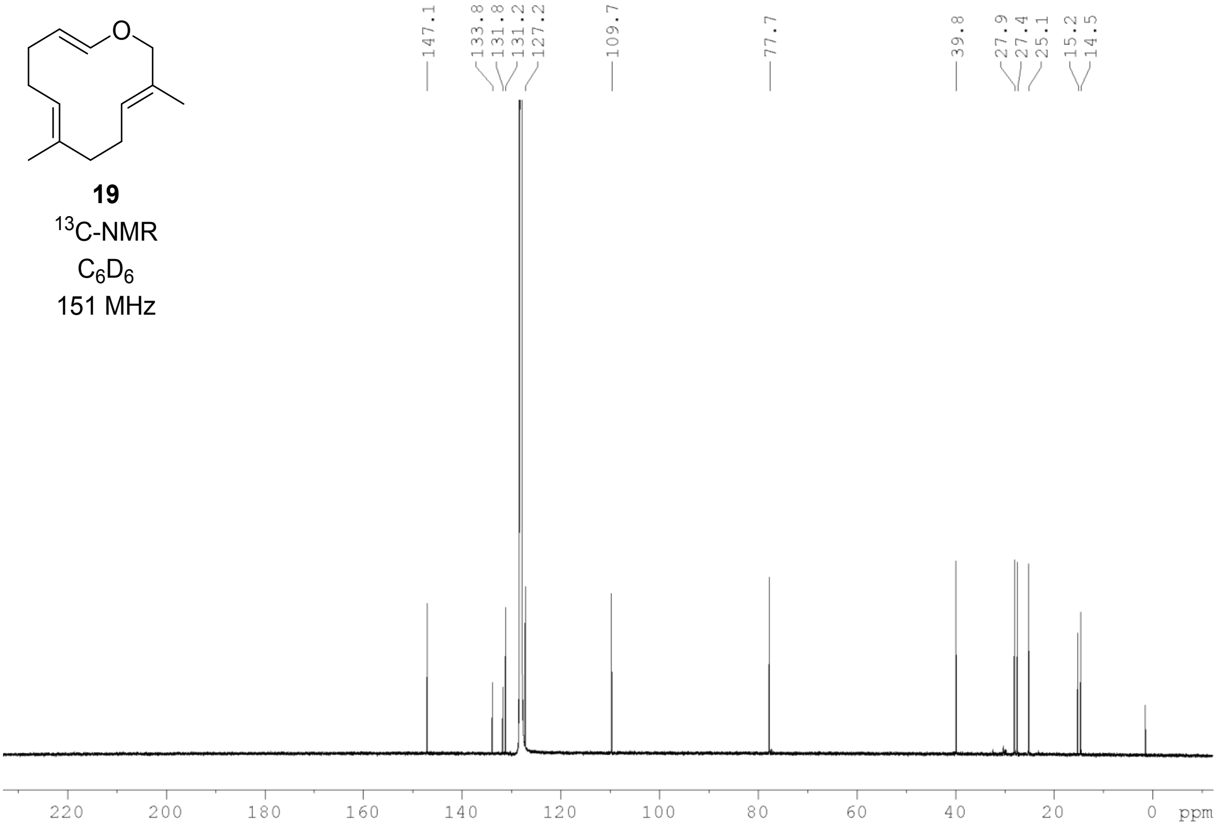

19
^{13}C-NMR
C_6D_6
151 MHz

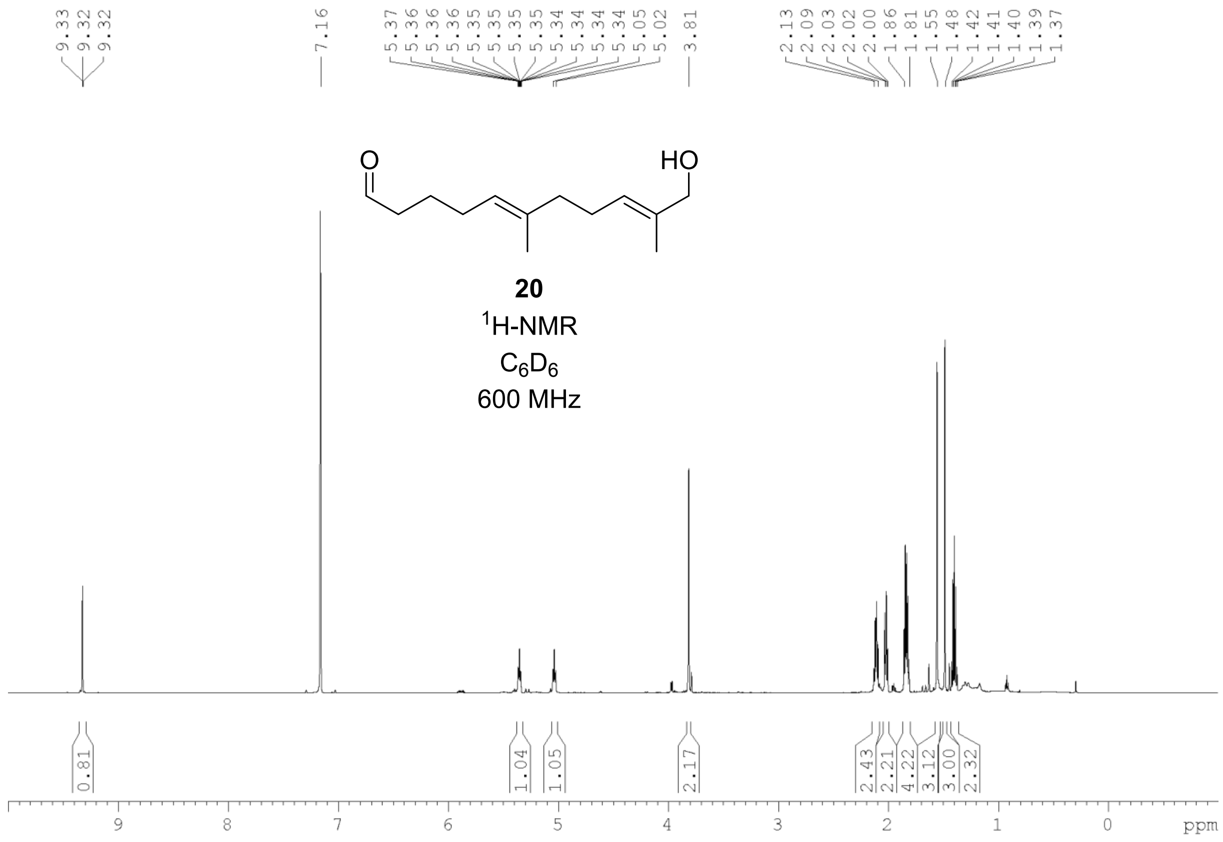
9.33
9.32
9.32
7.16
5.37
5.36
5.36
5.36
5.35
5.35
5.35
5.35
5.34
5.34
5.34
5.05
5.02
3.81
2.13
2.09
2.03
2.02
2.00
1.86
1.81
1.55
1.48
1.42
1.41
1.40
1.39
1.37
O
HO
20
1H-NMR
C6D6
600 MHz
0.81
1.04
1.05
2.17
2.43
2.21
4.22
3.12
3.00
2.32
9
8
7
6
5
4
3
2
1
0
ppm

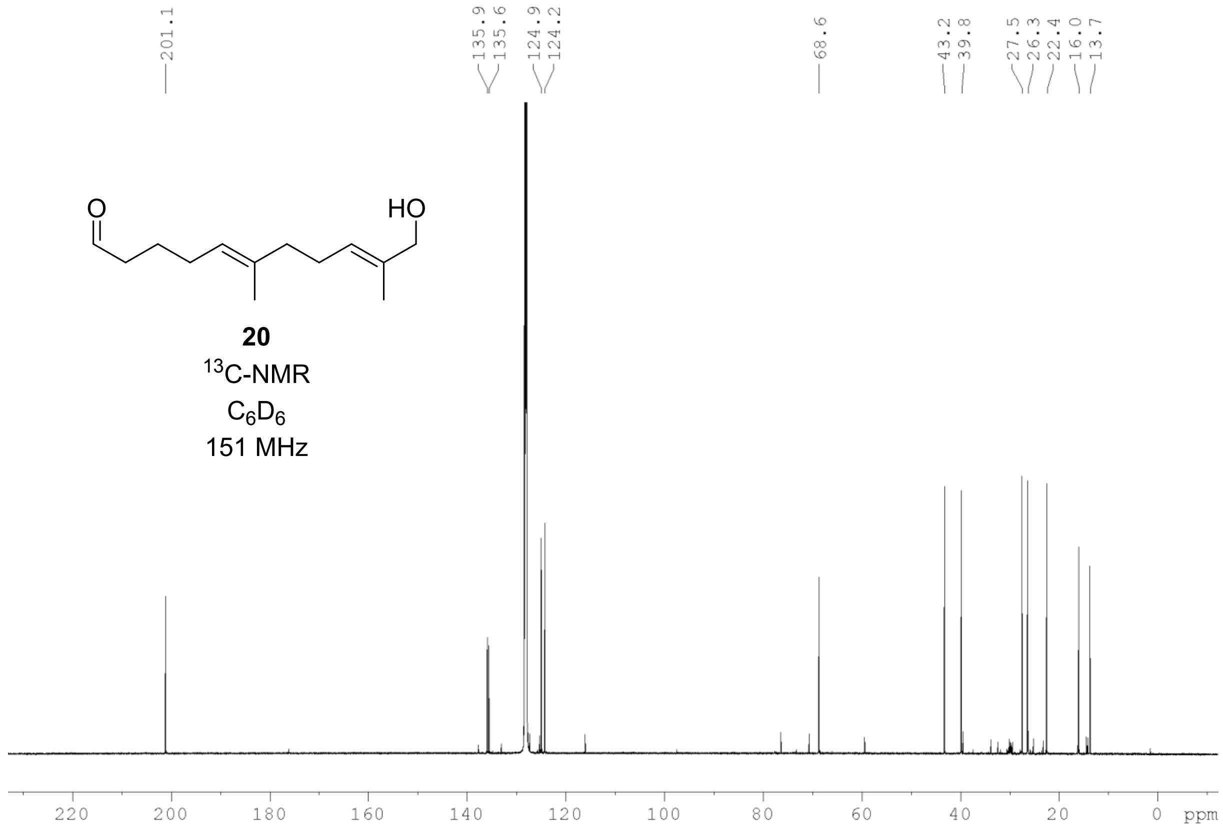
201.1
135.9
135.6
124.9
124.2
68.6
43.2
39.8
27.5
26.3
22.4
16.0
13.7
O
HO
20
13C-NMR
C6D6
151 MHz
220
200
180
160
140
120
100
80
60
40
20
0
ppm

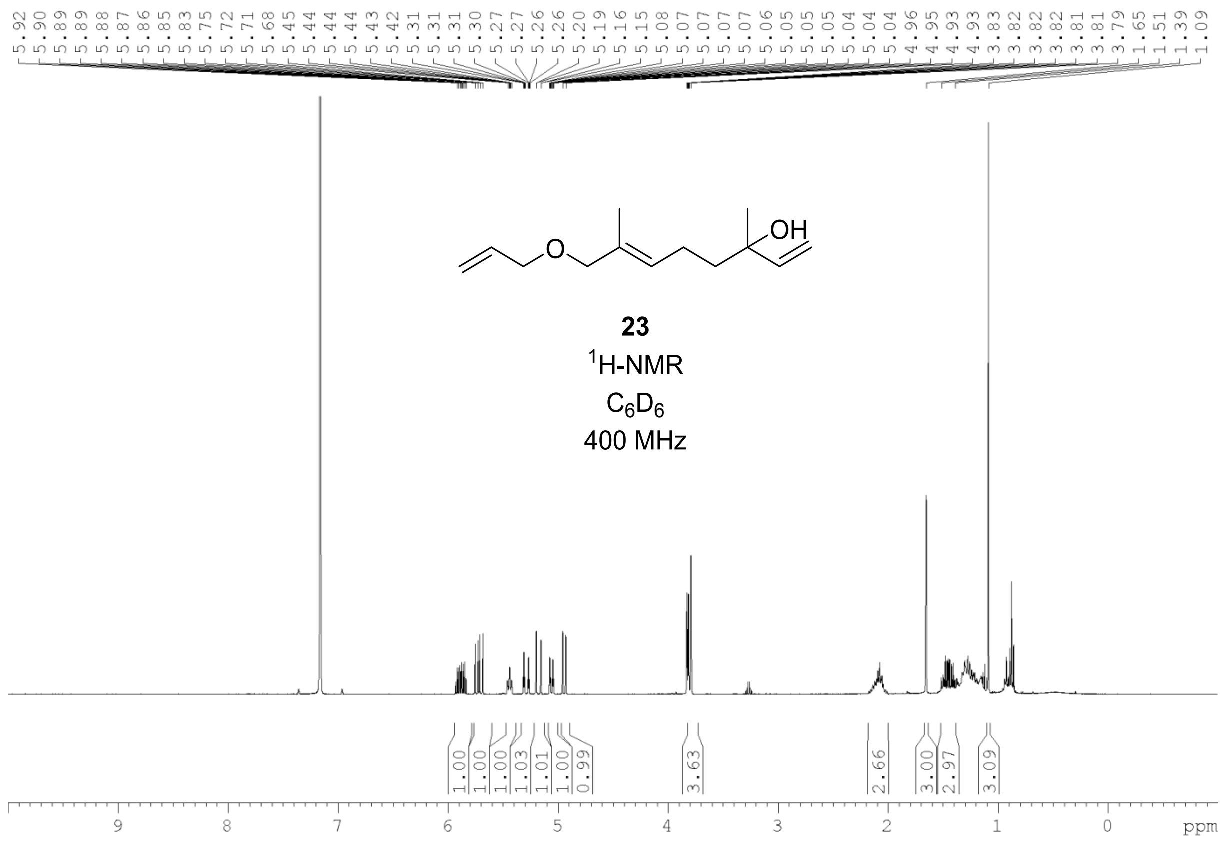
5.92 5.90 5.89 5.88 5.87 5.86 5.85 5.83 5.75 5.72 5.71 5.68 5.45 5.44 5.44 5.44 5.43 5.42 5.31 5.31 5.31 5.30 5.27 5.27 5.26 5.26 5.20 5.19 5.16 5.15 5.08 5.07 5.07 5.07 5.06 5.05 5.05 5.05 5.04 5.04 5.04 4.96 4.95 4.93 4.93 3.83 3.82 3.82 3.82 3.81 3.79 1.65 1.51 1.39 1.09
OH
O
23
^{1}H-NMR
C_6D_6
400 MHz
1.00 1.00 1.00 1.03 1.01 1.00 0.99 3.63 2.66 3.00 2.97 3.09
9 8 7 6 5 4 3 2 1 0 ppm

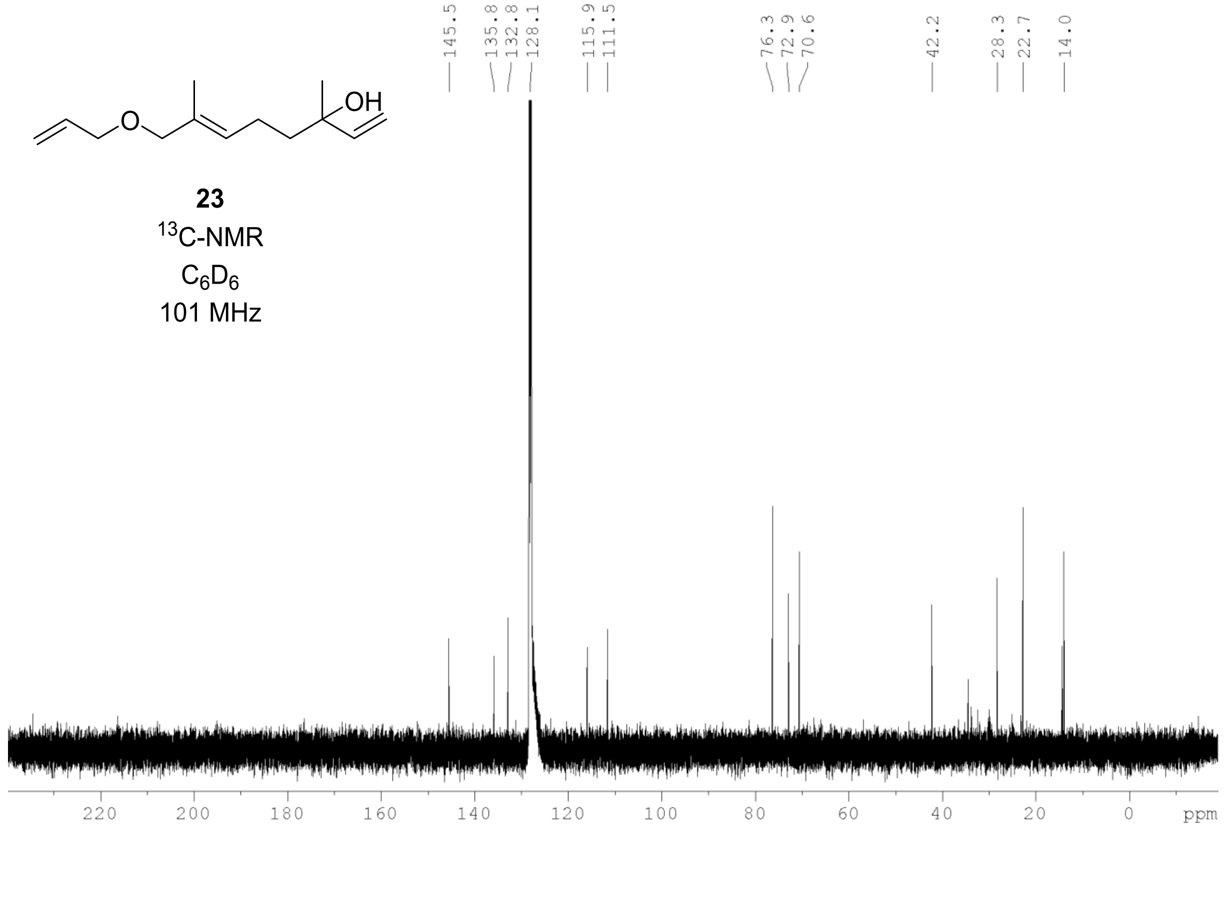
145.5 135.8 132.8 128.1 115.9 111.5 76.3 72.9 70.6 42.2 28.3 22.7 14.0
OH
O
23
^{13}C-NMR
C_6D_6
101 MHz
220 200 180 160 140 120 100 80 60 40 20 0 ppm

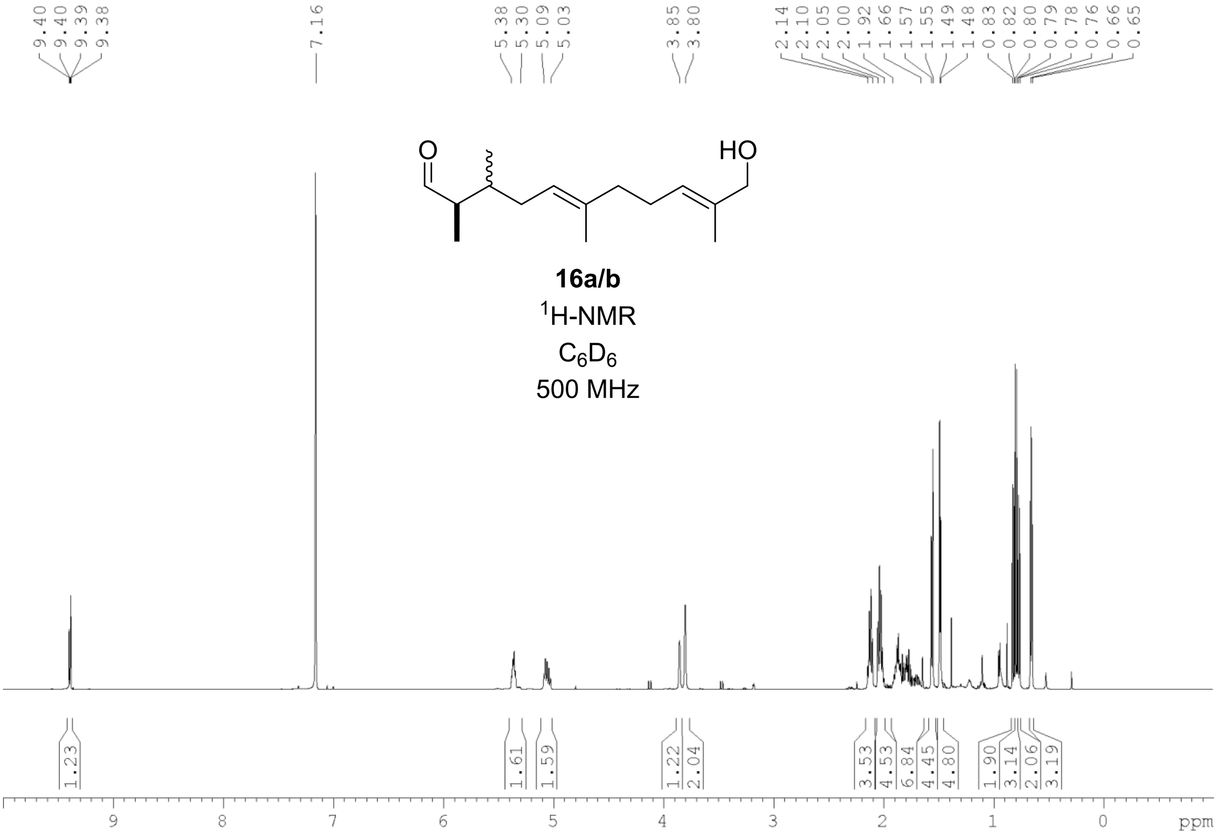
9.40 9.40 9.39 9.38
7.16
5.38 5.30 5.09 5.03
3.85 3.80
2.14 2.10 2.05 2.00 1.92 1.66 1.57 1.55 1.49 1.48 0.83 0.82 0.80 0.79 0.78 0.76 0.66 0.65
O
HO
16a/b
1H-NMR
C6D6
500 MHz
1.23 1.61 1.59 1.22 2.04 3.53 4.53 6.84 4.45 4.80 1.90 3.14 2.06 3.19
9 8 7 6 5 4 3 2 1 0 ppm

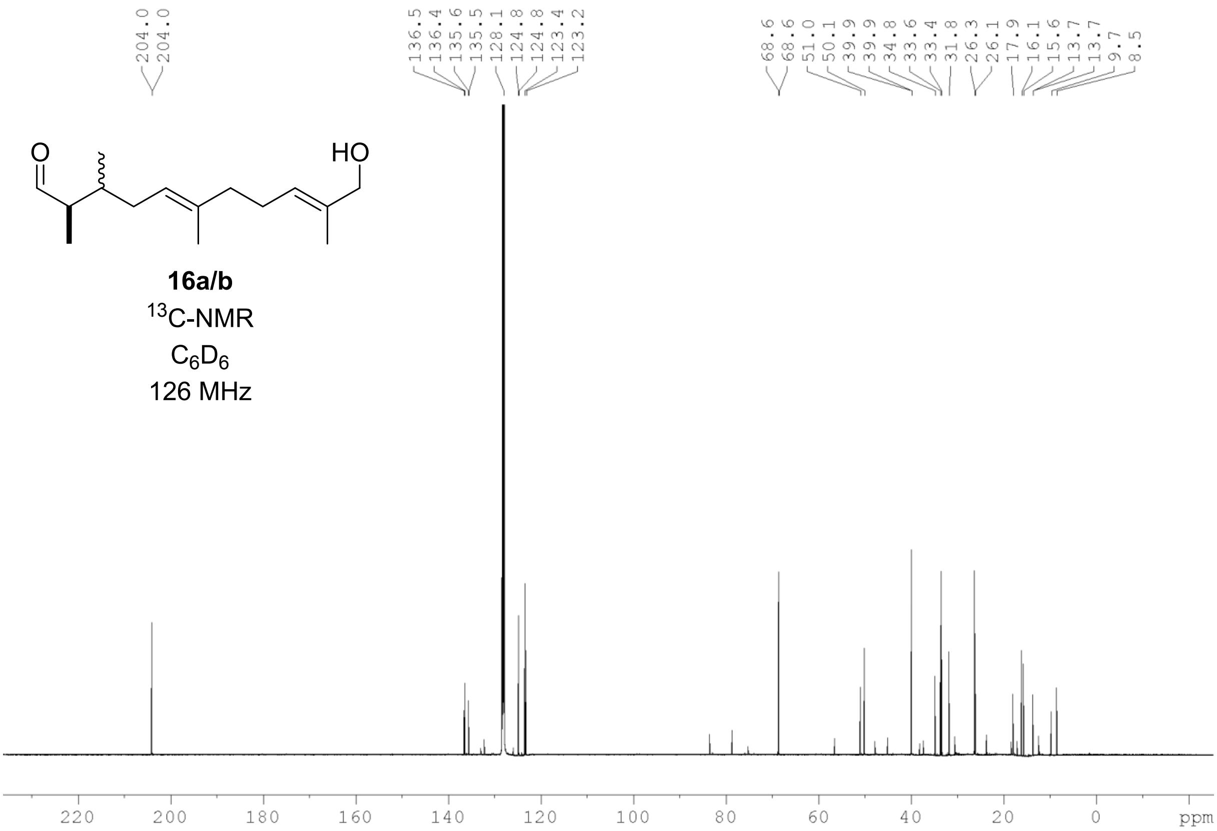
204.0 204.0
136.5 136.4 135.6 135.5 128.1 124.8 124.8 123.4 123.2
68.6 68.6 51.0 50.1 39.9 39.9 34.8 33.6 33.4 31.8 26.3 26.1 17.9 16.1 15.6 13.7 9.7 8.5
O
HO
16a/b
13C-NMR
C6D6
126 MHz
220 200 180 160 140 120 100 80 60 40 20 0 ppm

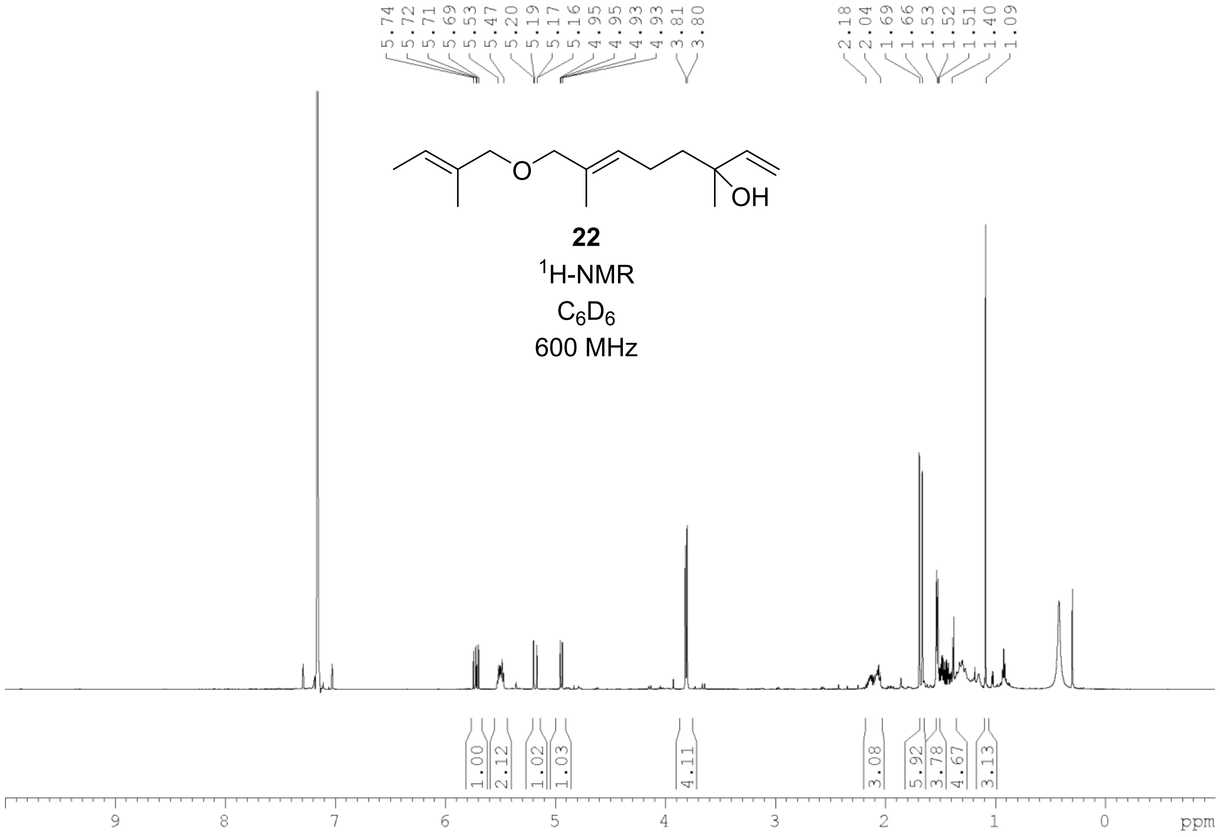
5.74 5.72 5.71 5.69 5.53 5.47 5.20 5.19 5.17 5.16 4.95 4.95 4.93 4.93 3.81 3.80
2.18 2.04 1.69 1.66 1.53 1.52 1.51 1.40 1.09
OH
22
^{1}H-NMR
C_6D_6
600 MHz
1.00 2.12 1.02 1.03 4.11 3.08 5.92 3.78 4.67 3.13
9 8 7 6 5 4 3 2 1 0 ppm

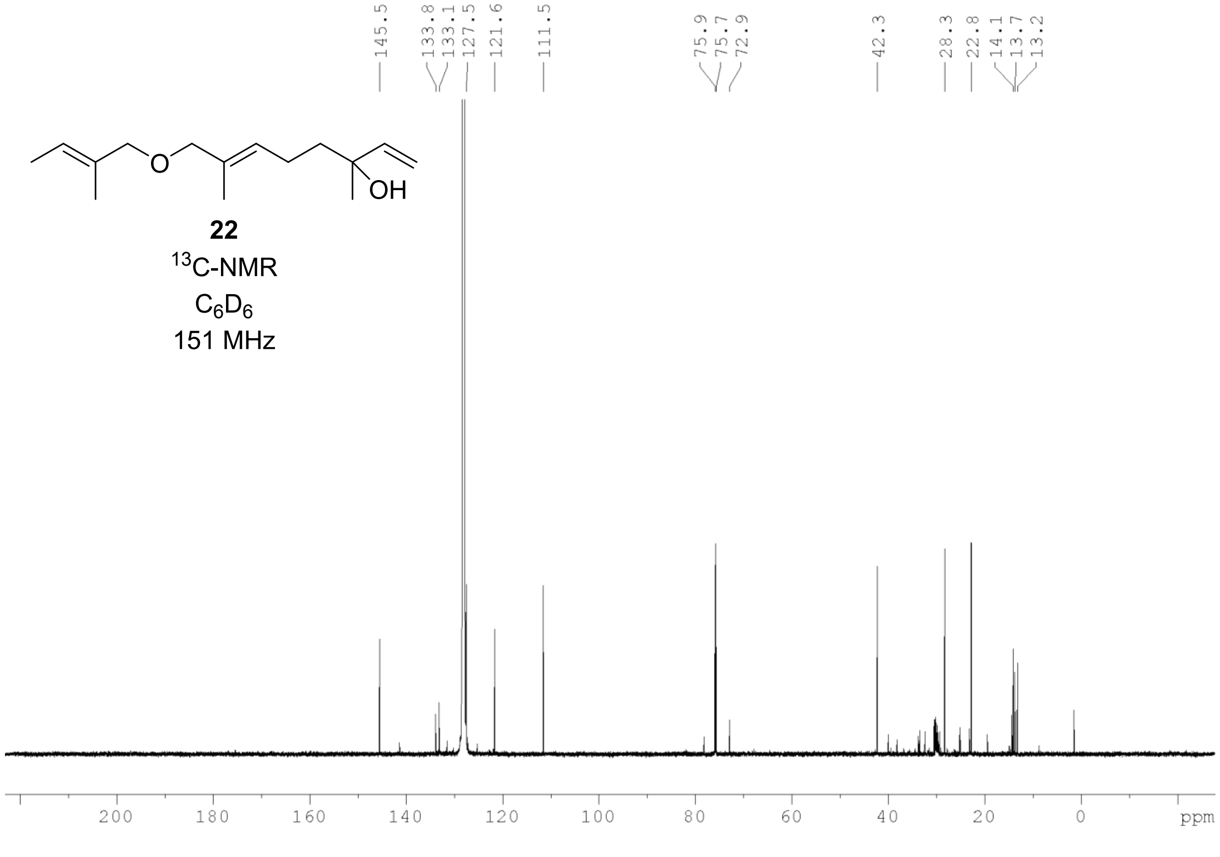
145.5 133.8 133.1 127.5 121.6 111.5 75.9 75.7 72.9 42.3 28.3 22.8 14.1 13.7 13.2
OH
22
^{13}C-NMR
C_6D_6
151 MHz
200 180 160 140 120 100 80 60 40 20 0 ppm

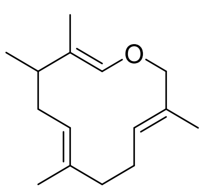

17
^{1}H-NMR
C_6D_6
600 MHz

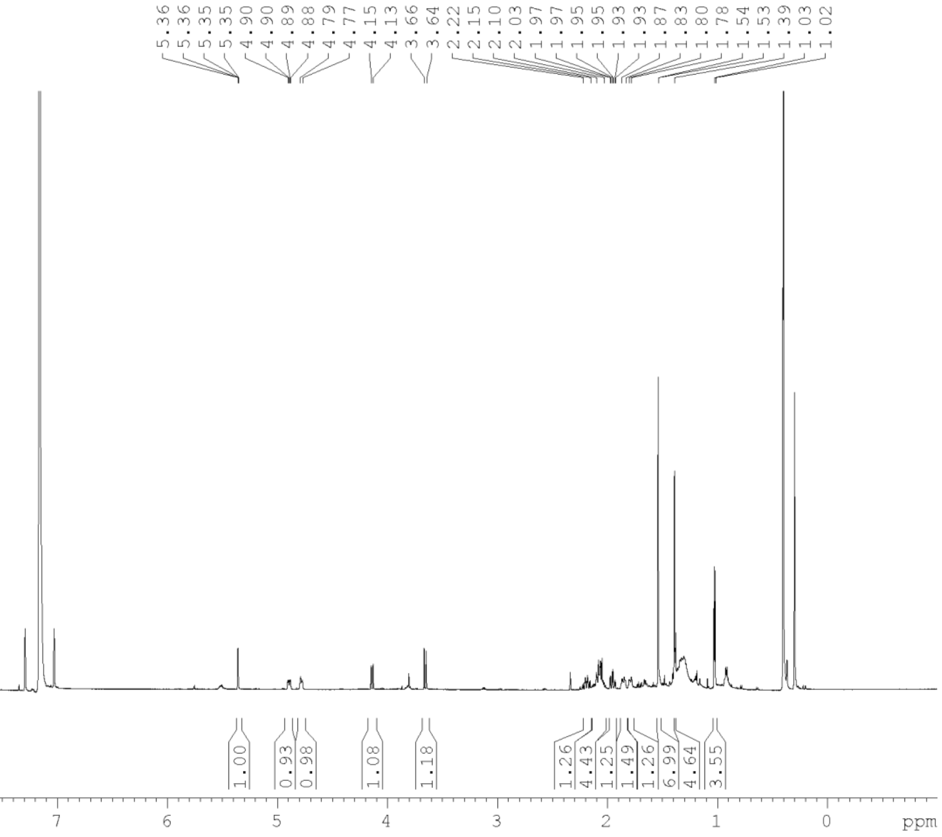

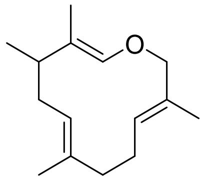

17
^{13}C-NMR
C_6D_6
151 MHz

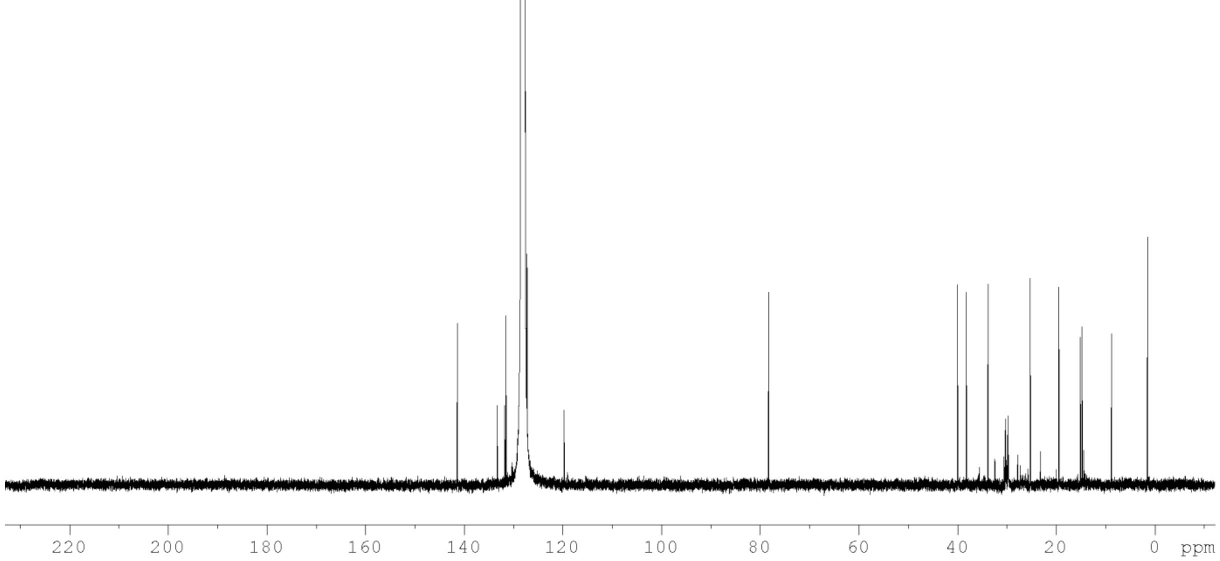

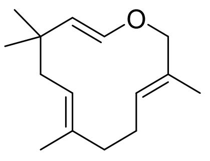

15
^{1}H-NMR
C_6D_6
600 MHz

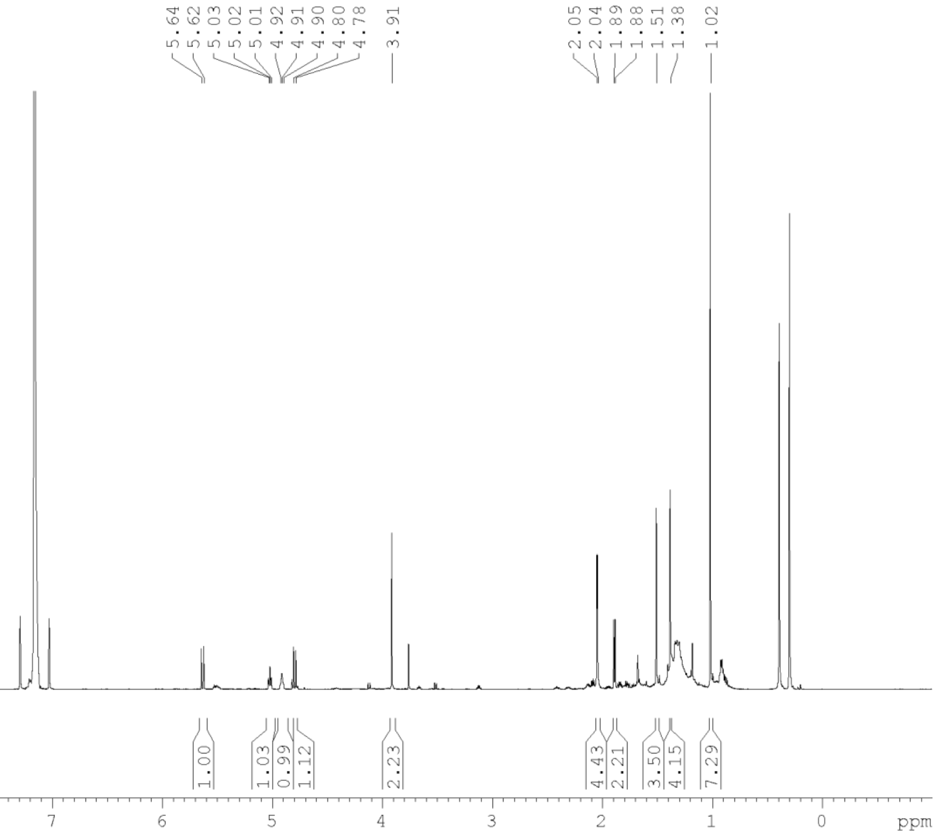

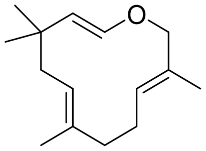

15
^{13}C-NMR
C_6D_6
151 MHz

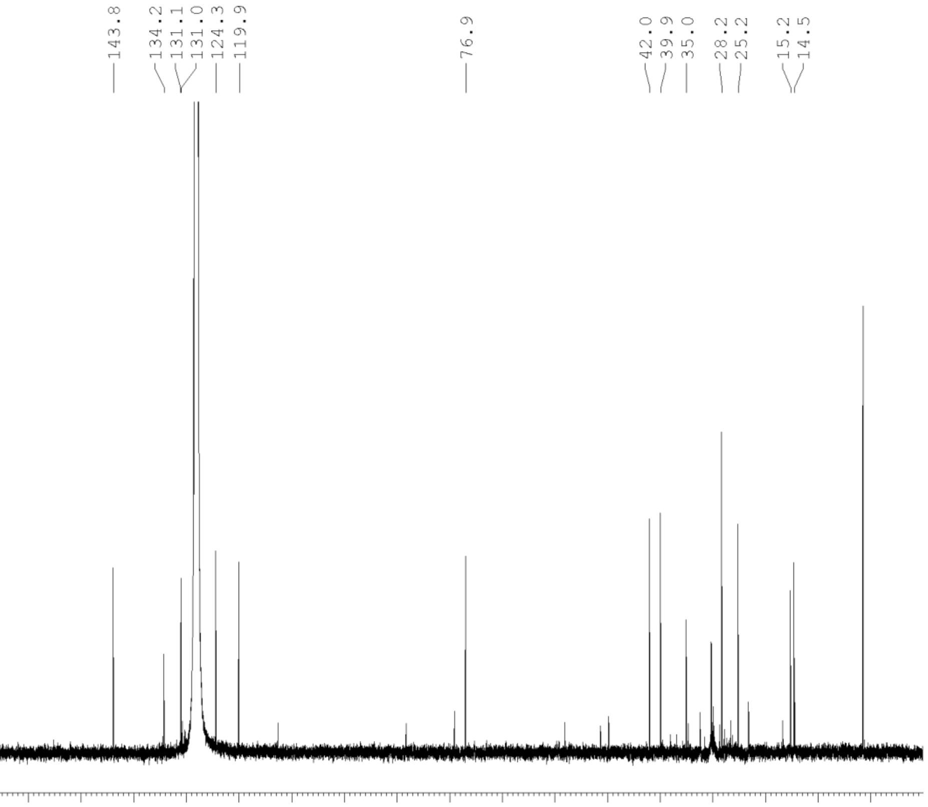

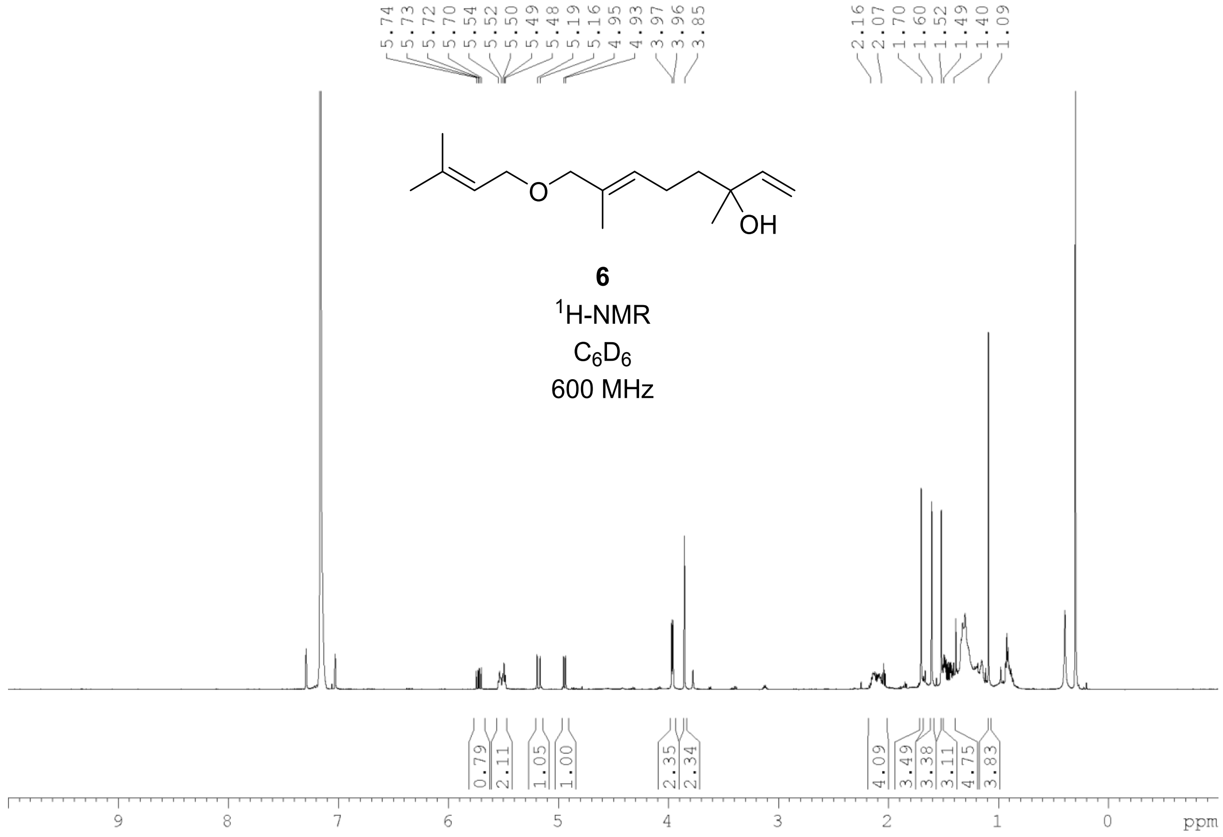
5.74
5.73
5.72
5.70
5.54
5.52
5.50
5.49
5.48
5.19
5.16
4.95
4.93
3.97
3.96
3.85
2.16
2.07
1.70
1.60
1.52
1.49
1.40
1.09
O
OH
6
1H-NMR
C6D6
600 MHz
0.79
2.11
1.05
1.00
2.35
2.34
4.09
3.49
3.38
3.11
4.75
3.83
9
8
7
6
5
4
3
2
1
0
ppm

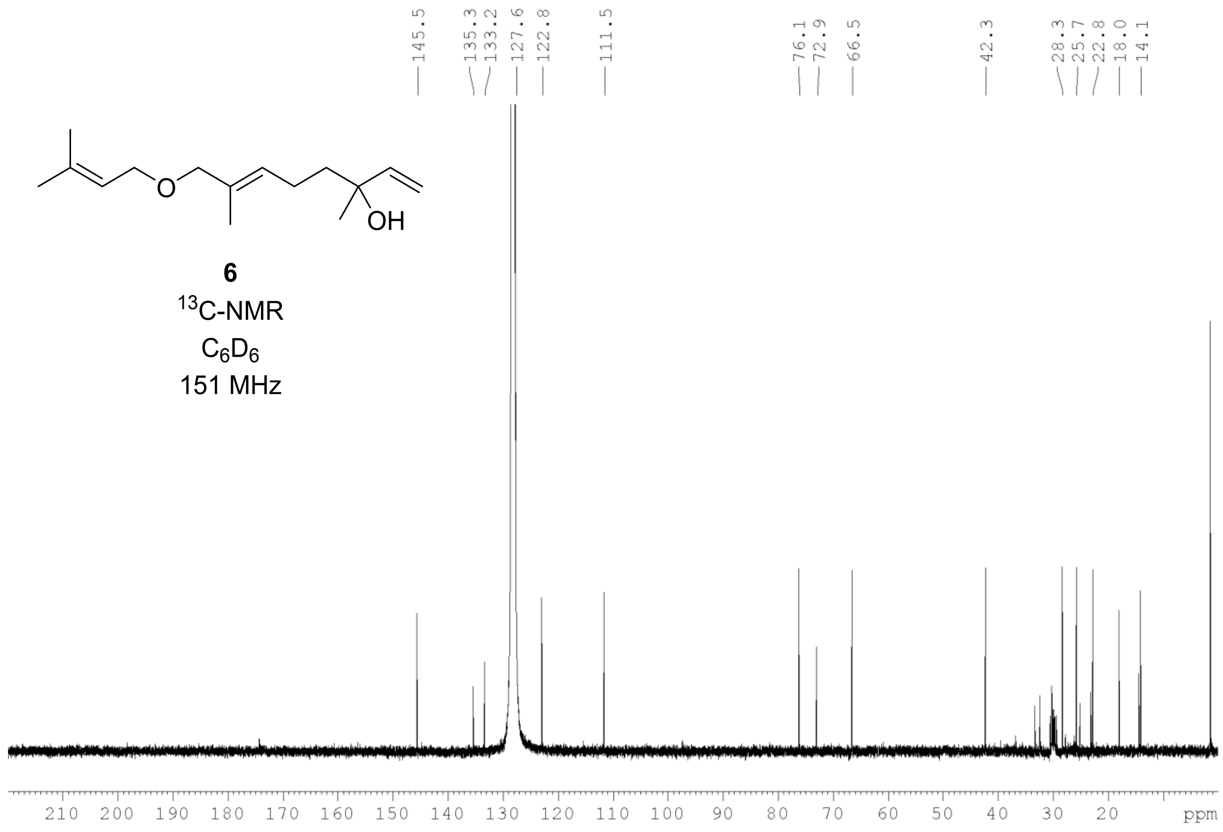
145.5
135.3
133.2
127.6
122.8
111.5
76.1
72.9
66.5
42.3
28.3
25.7
22.8
18.0
14.1
O
OH
6
13C-NMR
C6D6
151 MHz
210
200
190
180
170
160
150
140
130
120
110
100
90
80
70
60
50
40
30
20
ppm

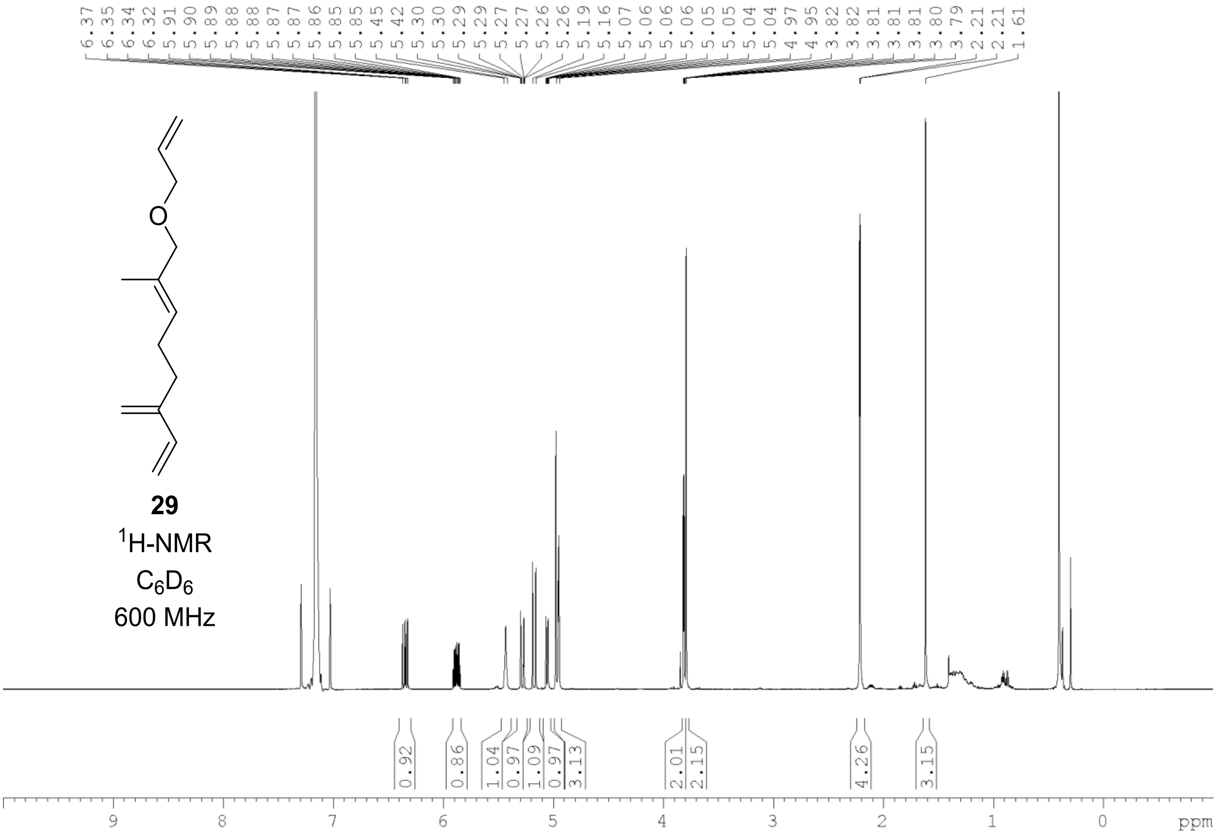
6.37 6.35 6.34 6.32 5.91 5.90 5.89 5.88 5.88 5.87 5.86 5.85 5.85 5.45 5.42 5.30 5.30 5.29 5.29 5.27 5.27 5.26 5.26 5.19 5.16 5.07 5.06 5.06 5.06 5.05 5.05 5.04 4.97 4.95 3.82 3.81 3.81 3.81 3.80 3.79 2.21 2.21 1.61
29
^{1}H-NMR
C_6D_6
600 MHz
0.92 0.86 1.04 0.97 1.09 0.97 3.13 2.01 2.15 4.26 3.15
9 8 7 6 5 4 3 2 1 0 ppm

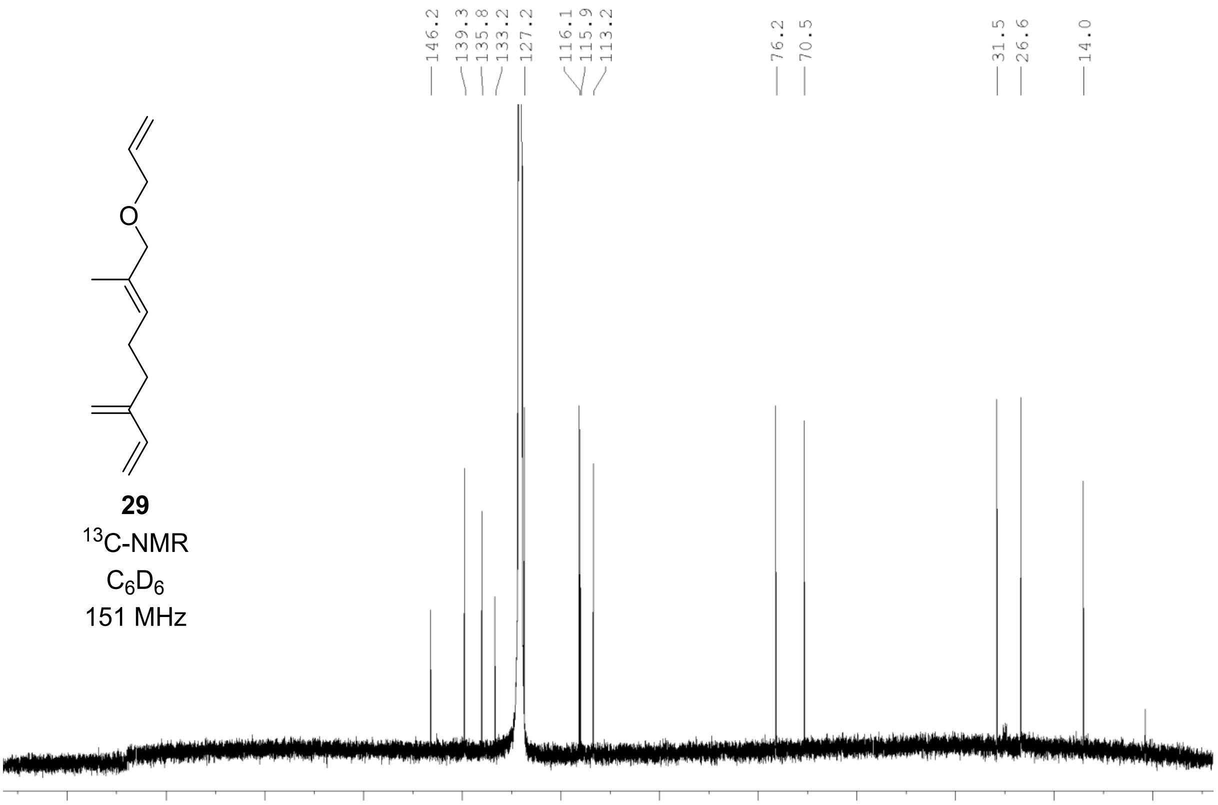
146.2 139.3 135.8 133.2 127.2 116.1 115.9 113.2 76.2 70.5 31.5 26.6 14.0
29
^{13}C-NMR
C_6D_6
151 MHz
220 200 180 160 140 120 100 80 60 40 20 0 ppm

3. References (supporting information)

(6a) Oberhauser, C.; Harms, V.; Seidel, K.; Schröder, B.; Ekramzadeh, K.; Beutel, S.; Winkler, S.; Lauterbach, L.; Dickschat, J. S.; Kirschning, A., Exploiting the Synthetic Potential of Sesquiterpene Cyclases for Generating Unnatural Terpenoids, *Angew. Chem. Int. Ed.* **2018**, *57*, 11802 – 11806.

(9) Johnson, R.; Bhat, W. W.; Sadre, R.; Miller, G. P.; Garcia, A. S.; Hamberger, B., Promiscuous terpene synthase from *Prunella vulgaris* highlight the importance of substrate and compartment switching in terpene synthase evolution, *New. Phytol.* **2019**, *223*, 323–335.

(13a) Cane, D. E.; Sohng, J. K.; Lamberson, C. R.; Rudnicki, S. M.; Wu, Z.; Lloyd, M. D.; Oliver, J. S.; Hubbard, B. R., Pentalenene Synthase. Purification, Molecular Cloning, Sequencing, and High-Level Expression in Escherichia coli of a Terpenoid Cyclase from Streptomyces UC5319, *Biochemistry* **1994**, *33*, 5846–5857.

(14b) Wawrzyn, G. T.; Quin, M. B.; Choudhary, S.; López-Gallego, F.; Schmidt-Dannert, C., Draft Genome of *Omphalotus olearius* Provides a Predictive Framework for Sesquiterpenoid Natural Product Biosynthesis in Basidiomycota, *Chem. Biol.* **2012**, *19*, 772-783.

(15) Wang, C. M.; Hopson, R.; Lin, X.; Cane, D. E., Biosynthesis of the Sesquiterpene Botrydial in Botrytis cinerea. Mechanism and Stereochemistry of the Enzymatic Formation of Presilphiperfolan-8β-ol, *J. Am. Chem. Soc.* **2009**, *131*, 8360–8361.

(19) Brown, H. C.; Imai, T., Organoboranes. 32. Homologation of Alkylboronic Esters with Methoxy(phenylthio)methyllithium: Regio- and Stereocontrolled Aldehyde Synthesis from Olefins via Hydroboration, *J. Am. Chem. Soc.* **1983**, *105*, 6285 – 6289.

(20) Nikolaiczyk, V.; Irwan, J.; Nguyen, T.; Fohrer, J.; Elbers, P.; Schrank, P.; Davari, M. D.; Kirschning, A., Rational reprogramming of the sesquiterpene synthase BcBOT2 yields new terpenes with presilphiperfolane skeleton, *Catal. Sci. Technol.* **2023**, *13*, 233.

(S1) Zhou, J.; Ogle, J. W.; Fan, Y.; Banphavichit(Bee), V.; Zhu, Z.; Burgess, K., Asymmetric Hydrogenation Routes to Deoxypolyketide Chirons, *Chem. Eur. J.* **2007**, *13*, 7162 – 7170.

(S2) Bonazzi, S.; Güttinger, S.; Zemp, I.; Kutay, U.; Gademann, K., Total Synthesis, Configuration, and Biological Evaluation of Anguinomycin C, *Angew. Chem. Int. Ed.* **2007**, *46*; 8707 – 8710.
